# Expanding the toolbox to develop IAP-based degraders of TEAD transcription factors

**DOI:** 10.1038/s42004-025-01871-x

**Published:** 2026-01-19

**Authors:** Nishma Gupta, Nicole Trainor, Mona Radwan, Stephanie Nguyen, Luke Duncan, Andrew X. Tang, Julia Beveridge, Natasha Silke, Jumana Yousef, Ceren Bilgilier, Johannes Wachter, Peter Greb, Zuzana Jandova, Ján Eliaš, Sara Kopf, Thomas Gerstberger, Peggy Stolt-Bergner, Nina Braun, Harald Weinstabl, Darryl B. McConnell, Federico Mauri, Isabelle S. Lucet, John Silke, Nicola E. A. Chessum, Michael J. Roy

**Affiliations:** 1 Inflammation Division, Walter and Eliza Hall Institute, Parkville, VIC Australia; 2 Department of Medical Biology, University of Melbourne, Parkville, VIC Australia; 3 ACRF Chemical Biology Division, Walter and Eliza Hall Institute, Parkville, VIC Australia; 4 Advanced Technology and Biology Division, Walter and Eliza Hall Institute, Parkville, VIC Australia; 5 Boehringer Ingelheim RCV GmbH & Co KG, Vienna, Austria; 6 Boehringer Ingelheim Pharma GmbH & Co KG, Biberach, Germany; 7 Present Address: Centre for Genetic Medicine, King Faisal Specialist Hospital & Research Centre, Riyadh, KSA Saudi Arabia; 8 Present Address: BioCurate Pty Ltd, Carlton, VIC Australia; 9 Present Address: Curie.Bio, Boston, MA USA; 10 Present Address: South Australian immunoGENomics Cancer Institute (SAiGENCI), Adelaide University, Adelaide, SA Australia

**Keywords:** Drug discovery and development, X-ray crystallography, Ubiquitin ligases, Small molecules, Structure-based drug design

## Abstract

The TEAD transcription factors (TEAD1-4) are critical effectors of the Hippo pathway, forming active nuclear complexes with transcriptional co-activators YAP/TAZ to regulate cell growth/apoptosis pathways and control fundamental processes such as organ size. Frequent dysregulation of the Hippo pathway in cancer and the presence of druggable binding sites on TEADs make them attractive targets for development of small molecule inhibitors and degraders. Here, we identify and mechanistically characterize three unique series of bifunctional degraders that target TEAD1 via a lipid pocket and recruit different members of the Inhibitor of Apoptosis proteins (IAPs) family to effect degradation of TEAD1. We provide a detailed toolkit for structural, biophysical and cellular profiling, including the development of a cellular target engagement assay for the lipid pocket of TEAD1 and an IAP/TEAD1 ternary complex formation assay. Our study therefore provides essential resources for detailed characterization of IAP-recruiting degraders and important tools and learnings for bifunctional degraders targeted to the lipid pocket of TEADs.

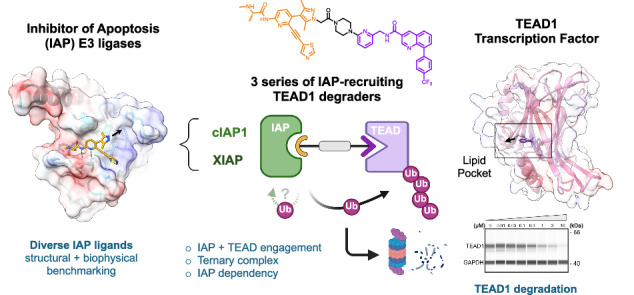

## Abstract (web summary)

TEAD transcription factors are critical effectors and druggable sites of the Hippo pathway in cancer, however, the development of small molecule inhibitors and degraders remains underexplored. Here, the authors identify and characterize bifunctional IAP-based degraders targeting TEAD1 *via* a lipid pocket and recruit different members of the inhibitor of apoptosis proteins (IAPs) family, offering a comprehensive toolkit for structural, biophysical and cellular profiling.

## Introduction

TEAD transcription factors are primary downstream effectors of the Hippo pathway, an evolutionarily conserved pathway regulating organ development and tissue homeostasis (including cell growth and apoptosis), that is one of the most frequently mutated pathways in human cancer^1^. In addition to genetic alterations in the pathway that occur in up to 10% of human cancers (including a variety of solid tumors such as lung, liver, breast, gastric, prostate and colorectal cancers), non-genetic dysregulation of the pathway also drives cancer phenotypes^1^. There are four human TEAD family paralogs (TEAD1–4), which share high sequence conservation, but differ in tissue- and development-specific expression^2^. Critically, all TEADs require co-factors to promote gene expression, including the two main transcriptional co-activators, yes-associated protein (YAP) and TAZ (transcriptional co-activator with PDZ binding motif)^2^.

Whilst TEADs are predominantly chromatin-localized in cells, in normal conditions, the transcriptional output of the pathway is regulated by a cascade of phosphorylation events triggered by upstream kinases that prevent the nuclear translocation of YAP/TAZ, leading to their sequestration or proteasomal degradation. This ultimately blocks TEAD transcriptional output, restricting cell growth. Conversely, inactivating mutations of these upstream kinases (such as the tumor suppressor protein Merlin, encoded by the NF2 gene) promote active nuclear TEAD/YAP/TAZ complexes and oncogenic transformation. NF2 mutations are present in several cancers, including mesothelioma^3^. The downstream role of TEADs as a convergence point in multiple pro-oncogenic signaling pathways make them attractive therapeutic targets^4^. Whereas YAP and TAZ are largely disordered, TEADs possess a structured DNA-binding domain and trans-activating YAP-binding domain (YBD), which has been a primary focus for recent development of small molecule inhibitors of the pathway^2,5^. Most of these inhibitors target a druggable conserved lipid pocket identified in the TEAD YBD (“P-site”) that recognizes palmitic acid, leading to TEAD auto-palmitoylation^6–9^. P-site inhibitors block TEAD auto-palmitoylation and, via an allosteric mechanism, can indirectly prevent cofactor binding and transcriptional output^5,8,10^. Multiple Phase I clinical trials using such P-site molecules have been initiated, primarily for advanced mesothelioma patients carrying Hippo pathway mutations (NF2), with VT3989 (NCT04665206) paving the way and showing that this class of TEAD inhibitors is clinically active^11^. More recently, a potent molecule able to inhibit TEAD/YAP-TAZ interaction directly has also been described and entered clinical development (IAG933 / NCT04857372)^12–14^.

We aimed to exploit a targeted protein degradation (TPD) strategy for TEADs by harnessing reversible P-site ligands. We envisioned that, relative to the allosteric mechanism of P-site inhibitors, TEAD degraders might more effectively block the full complement of TEAD/cofactor interactions^15^, and offer potential advantages in terms of potency, differentiated TEAD paralog selectivity and pharmacodynamic profile^16,17^. TPD can be achieved via the generation of heterobifunctional degrader molecules (also known as PROTACs), which recruit a target protein to a ubiquitin E3 ligase complex, forming a target-degrader-E3 ternary complex. This can then enable ubiquitination and rapid proteasomal degradation of the target protein. Only a small subset of the estimated 600 human E3 ligase complexes have been successfully utilized for TPD to date, and most prominent amongst these are Von Hippel Lindau Protein (VHL) and Cereblon (CRBN), each part of multi-subunit cullin RING E3 ligase complexes^18–20^.

Inhibitor of Apoptosis proteins (IAPs), a family of single-chain RING E3 ligases that regulate cell death and inflammatory signaling pathways, have also been explored as E3s for TPD. Potent ligands, collectively known as SMAC mimetics, that bind conserved BIR domains in the IAPs have been developed for various IAP family members, including cIAP1/2 and XIAP. These agents have been explored therapeutically in single-agent or combination studies in oncology, immuno-oncology and infectious disease^21–25^. IAP-harnessing protein degraders (referred to here as IPDs), also termed specific and non-genetic IAP-dependent protein erasers (SNIPERs)^26^, have also been successfully developed to degrade targets including nuclear receptors and kinases^16,27–32^. IPDs/SNIPERs have some distinctive features relative to VHL/CRBN TPDs, including: (i) potential to recruit multiple IAP family members and (ii) on-target auto-ubiquitination and degradation of the E3 ligase. Capacity to degrade a target via multiple different E3s offers a potential advantage in cancer to avoid resistance mechanisms driven by alterations to any one specific E3 ligase. IAP degradation, in the case of cIAP1/2-targeted IPDs and SMAC mimetics, typically occurs rapidly upon cIAP1/2 engagement and activation^33,34^, whilst for XIAP this is less common and target dependent^35^. Although conceptually, IAP auto-degradation might hamper potent degradation of the target, this characteristic of IPDs may also have advantages in particular cancers by co-targeting of cell death pathways regulated by IAPs^36,37^. For example, IAPs are reported to be overexpressed in malignant pleural mesotheliomas^38–40^, a cancer frequently characterized by mutations in the Hippo pathway^41^.

In this work, we report the development and screening of a series of IPDs to target TEAD1 for degradation via a ligand directed to the TEAD palmitoylation pocket (P-site). We identify three series of IAP-recruiting degraders that achieve partial degradation of TEAD1 with nanomolar-range DC_50_ and relative selectivity over TEAD2, 3, 4. Careful mechanistic investigation of IAP E3 dependency, including development of bespoke cellular TEAD engagement and TEAD1/IAP ternary complex formation assays, together point to both IAP-dependent and independent degradation mechanisms of TEAD1, with the first of the most potent two series recruiting primarily via cIAP1 and the second series via both cIAP1/XIAP. Our work therefore provides a robust framework and toolbox for developing and characterizing IAP-recruiting or TEAD-targeting degraders.

## Results

### IAP and TEAD binder characterization for library development

**Fig. 1:**
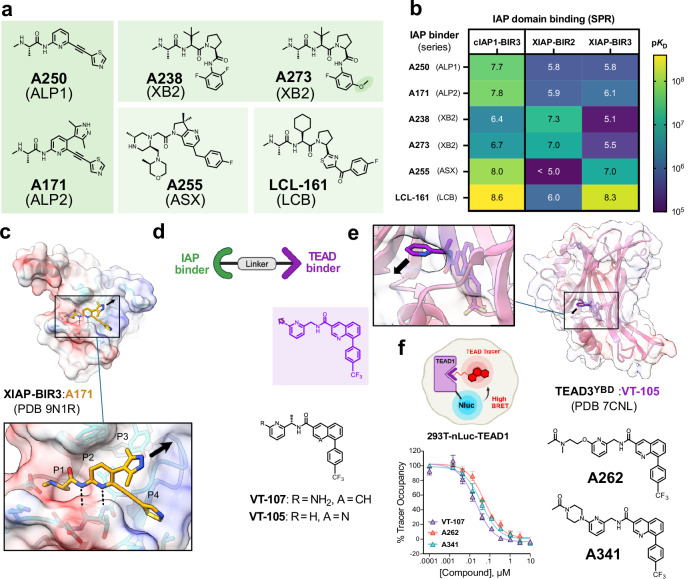
TEAD IPD screening library elements. **a** Chemical structures of IAP binders (ALP1 ligand **A250**, ALP2 ligand **A171**, XB2 ligand **A238**^27^, ASX ligand **A255**^47^ and LCB ligand **LCL-161**^48^). **b** SPR binding data for IAP binders to individual IAP BIR domains (cIAP1-BIR3, XIAP-BIR2^C202A, C213G^ and XIAP-BIR3). Affinity data is shown as a heat map where the color scale and values reflect the mean dissociation constant, *K*_D_, plotted as p*K*_D_, where p*K*_D_ = −log_10_*K*_D_). Fitted sensorgrams and aggregated biophysical binding data are provided in Supplementary Fig. 1e and Supplementary Data 1. IAP binding profiles are further supported by orthogonal binding data from competitive TR-FRET IAP binding assays shown in Supplementary Fig. 1c. **c** Crystal structure of XIAP BIR3 in complex with ALP2 IAP ligand **A171**. The selected exit vector is marked with a black arrow. **d** Chemical structures of TEAD1 binders **VT-107** and **VT-105**^10^. **e** Based on the crystal structure of **VT-105** in complex with the TEAD3 YAP-Binding Domain (YBD), TEAD3^YBD^: **VT-105**, we selected a solvent exposed position on the TEAD ligand (black arrow) as an exit vector for linker installation. **f** TEAD ligands based on **VT-105**/**VT-107** and incorporating exit vectors (**A262** and **A341**), were synthesized and profiled relative to **VT-107** in a NanoLuc-TEAD1 cellular target engagement assay for displacement of a fluorescent tracer **A472** from the TEAD1 P-site (Supplementary Fig. 1g, h). Fitted data represent mean ± SD from *n* = 3 biologically independent experiments.

As a starting point to TEAD IPD discovery, we developed an extended combinatorial library of TEAD IPDs (approx. 150 compounds) based upon SMAC mimetics known to bind cIAP1/2 or XIAP (Fig. 1a and Supplementary Fig. 1a–d). This library sampled a range of IAP exit vectors (Supplementary Fig. 1d) and predominantly flexible linkers of diverse length and chemical composition, joined to a TEAD binder targeting the P-site.

The core IAP ligand scaffolds used in our library were two structurally related in-house alkynyl pyridine (ALP) SMAC mimetics^42^ (ALP1, ALP2) (Fig. 1a, b and Supplementary Fig. 1c, d), selected based on their high-affinity and cIAP1 BIR3-focussed binding profile and overall properties towards degrader development (e.g., relatively low molecular weight and hydrogen bond donor count)^43^. To reveal the binding mode and suitable exit vectors for library generation, we solved the crystal structures of ALP1 ligand **A250** and ALP2 ligand **A171** bound to XIAP BIR3 (Supplementary Fig. 1d and Fig. 1c). To increase the diversity of our IAP-targeting library, we also included additional IAP ligands that have previously been utilized for TPD, which possess high affinity for XIAP. Although many studies of IAP-recruiting degraders lack systematic analysis of the contributions of individual IAPs to degradation^27,44^, there are reports implicating both cIAP1^28,29^ and XIAP^45,46^ as critical for degradation activity. We envisioned that the most effective E3 to recruit may be contextual to factors such as the target protein, cell-dependent IAP expression level and specificity of the IAP recruiting ligand. We thus structurally and biophysically benchmarked the individual IAP BIR binding of additional SMAC mimetics for library inclusion (Fig. 1b, c and Supplementary Fig. 1c–f). This included an IAP binder series we refer to as the XB2 series (**A238** and related methoxy derivative **A273**)^27^ with high affinity to XIAP BIR2 and cIAP1 BIR3, and two additional series with high affinity to XIAP BIR3 and cIAP1 BIR3 (referred to here as the ASX and LCB series, represented respectively by **A255**^47^ and **LCL-161**^48^, (Fig. 1b and Supplementary Fig. 1d). In the case of these ligands, our structural analysis revealed the critical importance of the P1 pocket for ligand binding, whilst regions of the ligands in the P3/P4 pockets were in general more solvent exposed (Supplementary Fig. 1d). This observation, as well as the relative synthetic amenity for installation of modifications in the P4 pocket for these ligands, led us to focus on a permissive exit vector in the IAP P4 pocket similar to that previously utilized for **LCL-161**-based IPDs^46^ (Supplementary Fig. 1d). This exit vector also echoes the trajectory of the SMAC protein (C-terminal to the IAP binding motif, IBM) when in complex with XIAP-BIR3 (Supplementary Fig. 1a). These additional IAP ligands were used to generate an analogous tailored library of IPDs of approximately 100 compounds. Binding to cIAP2 was not measured due to its high sequence homology to cIAP1 and typically much lower cellular expression compared with cIAP1^49^.

The TEAD binder selected was based on a simplified derivative of the reversible TEAD P-site ligands **VT-107** and **VT-105** (Fig. 1d)^10^. A solvent accessible exit vector was selected based on the TEAD3^YDB^ **VT-105** crystal structure (PDB: 7CNL, Fig. 1e) and used to generate acylated TEAD binders incorporating either aminoethyl or piperazine groups as points for linker attachment (**A262** and **A341**, Fig. 1f)^10^. To confirm retention of binding to the TEAD1 lipid pocket in cells, we developed a cellular nanoBRET target engagement assay utilizing nanoluciferase (NanoLuc) tagged TEAD1 and a custom fluorescent TEAD1 tracer (Fig. 1f and Supplementary Fig. 1g, h). This demonstrated that our modified TEAD binders incorporating exit vectors for IPD linker attachment retained potent binding to cellular TEAD1 (Supplementary Fig. 1h). We also assessed the effect of these ligands on TEAD1 stability in the NCI-H2052 mesothelioma cell line used for degradation screening, observing at higher concentrations (3–10 µM) a slight reduction in the levels of endogenous TEAD1 (averaged around 35% @ 10 µM for **A262** and **A341**) for the binders alone (Supplementary Fig. 1i).

### TEAD IPD library screening and hit identification

**Fig. 2:**
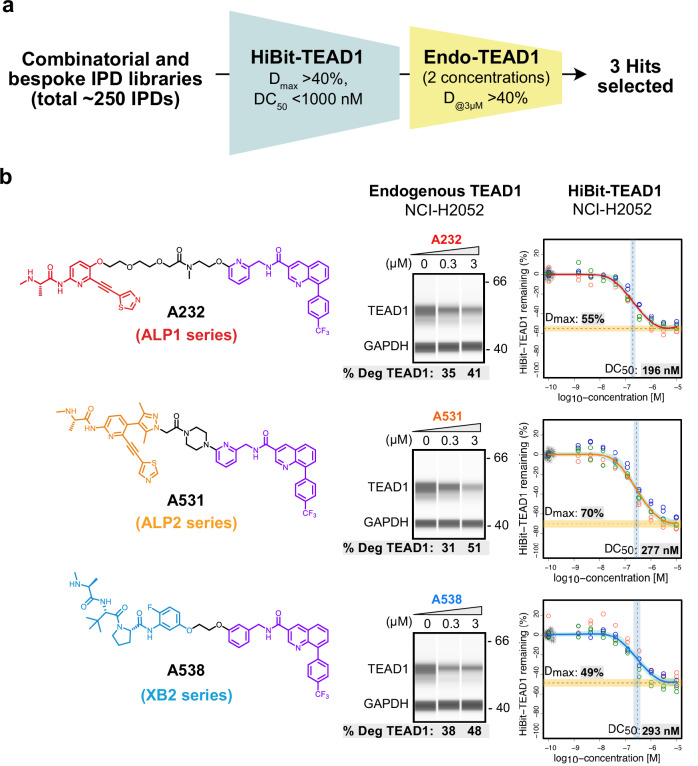
Screening of TEAD IPD library and hit identification. **a** IPD libraries were screened in a 20 h dose–response using a TEAD1 luciferase degradation assay (HiBiT-TEAD1, NCI-H2052 cells). Compounds with *D*_max_ > 40% and DC_50_ < 1000 nM were further selected for a follow-up 2 concentration screen for endogenous TEAD1 degradation (20 h treatment with 0.3 and 3 µM IPD, NCI-H2052 cells). Three IPDs that achieved >40% degradation of endogenous TEAD1 were selected. **b** Chemical structures of the three IPDs selected (ALP **A232**, ALP2 **A531**, and XB2 **A538**) representing different IAP binder series, linkers and exit vectors (left panel), and corresponding endogenous TEAD1 and HiBiT-TEAD1 degradation results (middle and right panel). For endogenous TEAD1, NCI-H2052 cells were treated with IPDs (0.3 or 3 µM) or DMSO control for 20 h and RIPA lysates generated. Following capillary western electrophoresis, these were probed with TEAD1 and GAPDH (loading control) antibodies. Shown is one representative capillary western image for each IPD out of *n* = 2 independent experiments performed. Uncropped blot images are available in Supplementary Data 1. Percentage endogenous TEAD1 degradation (represented as mean % values) was calculated relative to 100% value of DMSO controls. For HiBiT-TEAD1 degradation (screening assay), NCI-H2052 cells stably expressing HiBiT-TEAD1 were treated for 20 h with a dose–response of IPDs or DMSO control. Percentage TEAD1 remaining was plotted based on HiBiT luminescence normalized to CTG (HiBiT/CTG ratio) relative to vehicle control. Plotted data represent individual data points from *n* = 3 independent experiments. Degradation DC_50_ and *D*_max_ were fitted as described^92^.

To identify TEAD1 degraders, the full IPD screening library was initially profiled in a luminescent reporter degradation assay using HiBiT-TEAD1 (Fig. 2a). Hits (defined as maximal degradation, *D*_max_ > 40% and half-maximal degradation concentration, DC_50_ < 1000 nM) were chosen for follow-up profiling for endogenous TEAD1 degradation in a two-concentration screen (0.3 and 3 µM) in mesothelioma NCI-H2052 cells treated for 20 h (Supplementary Fig. 2). For analysis of endogenous protein degradation, a capillary based western assay (JESS, Simple Western^TM^) was optimized that could profile target proteins in cell lysates from as few as 1000 seeded cells. Compounds that gave higher degradation in both assays (>40% degradation of endogenous TEAD1 at 3 µM; *D*_max_ > 40% for HiBiT-TEAD1, Fig. 2b) were chosen for full dose response profiling of endogenous TEAD1 degradation. From these screening results three promising IPD hits were selected for follow-up: two based on the in-house ALP series IAP binder, ALP1 **A232** (41% TEAD1 degradation following 3 µM treatment), ALP2 **A531** (51% TEAD1 degradation following 3 µM treatment) and one based on the XB2 series IAP binder, XB2 **A538** (48% TEAD1 degradation following 3 µM treatment) (Fig. 2b and Supplementary Fig. 2). Comparison of TEAD1 degradation profiles at 3 µM concentration in both HiBiT and endogenous TEAD1 assay formats clearly showed a consistent clustering of higher TEAD1 degradation for the three identified hit IPDs as compared to TEAD binders **VT-107, A262** and **A341** (Supplementary Fig. 2). Despite more modest TEAD1 degradation, ALP1 **A232** was included as it shared a similar exit vector and overall linker length to ALP2 **A531**. Comparing endogenous degradation for the hits at 0.3 µM, the best efficacy was observed for XB2 **A538** (TEAD1 *D*_max_ of 38%), relative to ALP2 **A531** (TEAD1 *D*_max_ of 31%) and ALP1 **A232** (TEAD1 *D*_max_ of 35%, Fig. 2b). Overall, ALP2 **A531** and XB2 **A538** showed slightly higher endogenous TEAD1 degradation, and we focused on these for follow-up profiling.

### IPD hits induce modest endogenous TEAD1 degradation

**Fig. 3:**
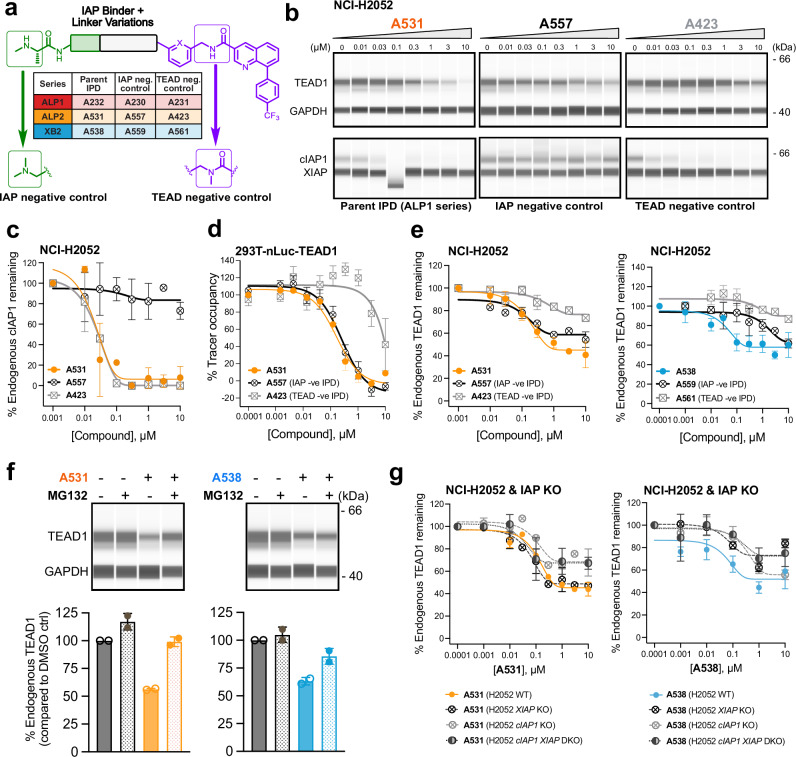
Endogenous TEAD1 degradation profiling of IPD hits and negative controls. **a** Molecular matched pair IAP- or TEAD-negative controls were generated by modification of the IAP BIR binding N-methylalanine group with N,N-dimethylglycine or TEAD binding central amide -NH methylation (refer Supplementary Fig. 3a for full chemical structures of IPD negative controls). **b** Profiling of endogenous TEAD1 and cIAP1 degradation using capillary western electrophoresis (20 h treatment, dose titration and DMSO, NCI-H2052 cells). % Endogenous TEAD1 and cIAP1 degradation was quantified relative to DMSO samples, and dose response curves (as in **c**, **e**, **g**) fitted using one-phase decay model to calculate *D*_max_ and DC_50_ values. **c** Endogenous cIAP1 auto-degradation curves in NCI-H2052 cells for ALP2 **A531** (orange line) and matched IAP and TEAD negative control IPDs (black and gray lines, respectively). **d** Profiling of IPDs in cellular TEAD1 target engagement assay. Dose–response NanoBRET signal was measured for displacement of a fluorescent TEAD tracer from NanoLuc-TEAD1 (HEK293T cells) following treatment with ALP2 **A531** (orange line) and matched IAP- and TEAD- negative IPD controls (**A557**, **A423**; black and gray lines respectively) and percentage tracer displacement plotted relative to a vehicle control. Data represent mean ± SD of *n* = 3 independent experiments. **e** Endogenous TEAD1 degradation curves for ALP2 A531 (left, orange line) and XB2 **A538** (right, blue line) and matched IAP and TEAD negative IPD controls (black and gray lines respectively). **f** Proteasome dependency analysis for ALP2 **A531** (orange) and XB2 **A538** (blue). Top panel shows western blot analysis of NCI-H2052 cells treated for 16 h with DMSO, 3 µM compound ±5 µM MG132. Bottom panel shows bar graph of % endogenous TEAD1 degradation relative to DMSO-treated cells. **g** IAP dependency of ALP hit **A531** (orange) and XB2 hit **A538** (blue) in isogenic WT and IAP KO NCI-H2052 lines. Plot shows endogenous TEAD1 degradation after 20 h compound treatment (5 concentrations with ten-fold serial dilutions starting from 10 µM and DMSO vehicle control) assessed in NCI-H2052 wildtype cells (orange/blue line), *cIAP1* KO (gray dashed line), *XIAP* KO (black dashed line) and *cIAP1/XIAP* DKO (black dotted line) cell lines. All data points for endogenous degradation curves represent mean ± SD of *n* = 2 biologically independent experiments, except *n* = 5 for **A531** and *n* = 3 for **A538** in (**e**) and *n* = 3 for (**g**). All uncropped blot images are available in Supplementary Data 1.

To validate TEAD- and IAP-binding dependency of our IPDs (Fig. 3a), we generated closely matched negative control pairs with small modifications designed to negate either IAP or TEAD binding. The IAP binding moieties are SMAC mimetics and can therefore promote auto-degradation of cIAP1 (Fig. 3b and Supplementary Fig. 1b)^33,34^. To generate IAP binder negative controls, we modified the basic N-methyl alanine motif common to all hit series that mimics the conserved amino-terminal alanine residue in the IAP-binding motifs of proteins, such as SMAC. This motif is critical for interaction with the acidic P1 pocket of all IAP BIR domains (Fig. 1c and Supplementary Fig. 1a, d). We predicted that replacement with an isobaric N, N-dimethylglycine moiety would disrupt these P1 interactions (Fig. 3a), and as expected, this modification completely prevented cellular cIAP1 auto-degradation by IAP negative control (ALP **A557**, Fig. 3b, c). The TEAD binder negative control was designed based on structural modeling (Supplementary Fig. 3b), by amide methylation of the TEAD binder (Fig. 3a). Evaluation of matched IPDs in the cellular target engagement assay confirmed that this modification led to >20-fold loss in TEAD1 binding (ALP **A423**, Fig. 3d). As expected, the TEAD binding negative control ALP **A423** promoted cIAP1 auto-degradation to similar levels as the IPD hits, confirming that cellular uptake and cIAP1 binding were unaffected by this modification (Fig. 3b, c and Table 1). Although XIAP has been reported to be co-degraded with the target protein by some SNIPERs^46^, we observed no effect on XIAP levels with any of our compounds (Fig. 3b). Similar cIAP1 auto-degradation and absence of XIAP degradation was also observed for XB2 **A538** and its matched IAP binding and TEAD binding negative controls **A559** and **A561**, respectively (Supplementary Fig. 3c and Table 1).

**Table 1:** Degradation of endogenous TEAD1, TEAD4 and cIAP1 with IPD hits and negative controls

Compound	Description	NCI-H2052 (TEAD1)		NCI-H2052 (TEAD4)		NCI-H2052 (cIAP1)		NCI-H226 (TEAD1)	
		DC_50_ (nM)	*D*_max_ (%)	DC_50_ (nM)	*D*_max_ (%)	DC_50_ (nM)	*D*_max_ (%)	DC_50_ (nM)	*D*_max_ (%)
**VT-107**	TEAD binder	3	21	-	-	-	-	-	-
**A262**	TEAD binder	800	36	-	-	-	-	-	-
**A341**	TEAD binder	2600	38	-	-	-	-	-	-
**A232** **A230** **A231**	ALP1 IPD hit ALP1 IAP neg. control ALP1 TEAD neg. control	14 970 4100	32 45 40	- - -	-	270 240 530	88 36 95	- - -	- - -
**A531** **A557** **A423**	ALP2 IPD hit ALP2 IAP neg. control ALP2 TEAD neg. control	170* 140 560	55* 41 23	1100 - -	33 - -	20 150 23	94 17 96	77 370 930	53 42 29
**A538** **A559** **A561**	XB2 IPD hit XB2 IAP neg. control XB2 TEAD neg. control	44^#^ 1500 630	42^#^ 37 10	510 - -	9 - -	39 920 360	100 7 97	76 780 1100	38 27 17

Endogenous degradation post 20 h compound treatment (10–0.001 µM) assessed using capillary-based western assay. Data are mean of *n* = 2 biologically independent experiments except for *(*n* = 5) and #(*n* = 3).

- = Not tested.

Cellular degradation experiments were undertaken for the three series of IPD hits (ALP **A232**, ALP2 **A531**, and XB2 **A538**) and matched negative controls to confirm endogenous TEAD1 degradation potency (DC_50_) and efficacy (*D*_max_). Overall, although the ALP2 IPD **A531** was consistently the most efficacious (highest maximal TEAD1 degradation), the XB2 IPD **A538** exhibited the clearest window of IAP dependency. NCI-H2052 cells were treated with three-fold serial dilutions from 10 to 0.01 µM of compounds or DMSO controls for 20 h, and assayed for endogenous TEAD1 and cIAP1/XIAP levels by capillary western analysis. In NCI-H2052 cells, the ALP2 IPD **A531** degraded endogenous TEAD1 with a *D*_max_ of 55% and DC_50_ of 170 nM. Relative to this, the matched IAP binding negative control **A557** showed a modest reduction in efficacy (*D*_max_), yet retained a similar DC_50_ (TEAD1 *D*_max_ 41%, DC_50_ 140 nM) (Fig. 3b, e and Table 1). The corresponding TEAD binding negative control, **A423**, had a more pronounced loss in TEAD1 degradation, with a 2–3 fold weaker *D*_max_ and DC_50_ (TEAD1 *D*_max_ 23%, DC_50_ 560 nM) (Fig. 3b, e). Comparing the ALP2 series and XB2 series IPD hits, the XB2 **A538** degrader was the more potent with a TEAD1 DC_50_ of 44 nM (Fig. 3e, Supplementary Fig. 3c and Table 1), but had a weaker *D*_max_ of 42%. However, the IAP binding negative control of this compound, XB2 **A559**, showed a marked loss in potency, with the DC_50_ right-shifted approximately 30-fold to 1500 nM (Fig. 3e). Relative to the XB2 hit **A538**, the matched TEAD negative control, XB2 **A561**, was also 14-fold less potent and 4-fold less efficacious (**A561:** TEAD1 *D*_max_ 10%, DC_50_ 630 nM). Lastly, dose response profiling of the ALP1 IPD hit **A232** and comparison with the matched IAP- and TEAD- negative control IPDs **A230** and **A231** (Supplementary Fig. 3a, d and Table 1) indicated this hit had overall a shallower degradation profile (ALP1 IPD hit **A232**: TEAD1 *D*_max_ 32% and DC_50_ 14 nM) and so was deprioritized for detailed characterization relative to the other two hits. For both ALP2 **A531** and XB2 **A538** degraders, a similar endogenous TEAD1 degradation profile was confirmed in another mesothelioma cell line NCI-H226, with TEAD1 *D*_max_/DC_50_ values of 53%/77 nM and 38%/76 nM, respectively, while IAP and TEAD binding negative controls were less effective at promoting TEAD1 degradation (Supplementary Fig. 3e and Table 1).

Overall, degradation profiling of the initial hits from each IPD series and matched negative controls (negating either IAP- or TEAD-binding) suggested that the observed TEAD1 degradation required TEAD binding and was only partially dependent on IAP engagement. The IAP-independent degradation activity may potentially be explained by the capacity of ligands designed for target inhibition to “supercharge” degradation through other mechanisms alongside direct proximity-induced degradation, a phenomenon which is increasingly recognized^50^.

### IPD hits degrade nuclear TEAD1 in a proteasome-dependent and partially IAP-dependent manner

To demonstrate that IPD-induced loss of endogenous TEAD1 was due to proteasome activity, we tested our IPDs in the presence of the proteasome inhibitor MG132. ALP2 **A531** mediated TEAD1 degradation (44% @ 3 µM, Fig. 3f) was completely abolished, and near 100% recovery of TEAD1 was observed with XB2 **A538** treatment in the presence of 5 µM of MG132 (Fig. 3f). These data confirm that the observed degradation of TEAD1 by all our IPD hits is proteasome dependent.

Since our degraders could function via one or more IAPs (Supplementary Fig. 1c), we next sought to delineate the IAP dependency of our compounds by profiling them in isogenic IAP-deficient cell lines. Since the predominant IAP family members expressed in the NCI-H2052 mesothelioma cell line used for screening are cIAP1 and XIAP, we generated *cIAP1* (*BIRC2*) and *XIAP* KO NCI-H2052 cells using CRISPR Cas9 (Supplementary Fig. 3f). The polyclonal *cIAP1* KO cell line did not express detectable levels of cIAP1 (Supplementary Fig. 3f). For XIAP, we used a CRISPR gRNA with a binding site that precedes the XIAP RING domain sequence, such that the resulting polyclonal *XIAP* KO line (Supplementary Fig. 3f) expressed, at low level, a truncated catalytically ‘dead’ form of XIAP, lacking the C-terminal RING domain required for ubiquitination. Single cell clones were generated from the polyclonal KO lines and confirmed by western analysis (right panel Supplementary Fig. 3f). The *cIAP1* KO was validated by confirming activation of the non-canonical NF-κB pathway^33,34^, that results in increased expression of NFκB2 p100, and processing to p52, compared to wild type cells (Supplementary Fig. 3g) A *cIAP1*/*XIAP* double KO line was generated in the same way from a validated *cIAP1* KO single clone (Supplementary Fig. 3h).

Endogenous TEAD1 degradation was then compared in H2052 wild-type (WT) and IAP KO cell lines. For ALP2 **A531**, a *D*_max_ of 55% was observed in NCI-H2052 WT cells that was reduced to 34% in H2052 *cIAP1* KO (Fig. 3g and Supplementary Fig. 3i, j). Consistent with the much tighter binding of the ALP series to cIAP1 and weaker binding to XIAP (Supplementary Fig. 1c), we observed that degradation of endogenous TEAD1 was not affected by loss of XIAP (D_max_ 49% in *XIAP* KO compared to 55% in WT cells) (Fig. 3g and Supplementary Fig. 3i, j). Consistent with this observation, the level of TEAD1 degradation for ALP2 **A531** was also similar in both *cIAP1* single KO and *cIAP1*/*XIAP* double KO cell lines (*D*_max_ of 34% and 33% respectively, Fig. 3g). Overall, this data suggested a cIAP1 dependency for ALP2 **A531**. In contrast, and consistent with the tight binding biophysically of the XB2 binder to both XIAP and cIAP1 (XIAP BIR2 and cIAP1 BIR3; Supplementary Fig. 1c), XB2 **A538** showed an apparent dependency on both cIAP1 and XIAP, with approximately 2-fold reduction in *D*_max_ of *XIAP* KO and 5-fold reduction in DC_50_ for *cIAP1* KO (WT: TEAD1 *D*_max_ 48%, DC_50_ 64 nM; *XIAP* KO: *D*_max_ 27%, DC_50_ 61 nM; *cIAP1* KO: *D*_max_ 44%, DC_50_ 340 nM, (Fig. 3g and Supplementary Fig. 3i, j). Although loss of cIAP1 reduced potency, there was no marked difference in degradation *D*_max_ between *XIAP* KO and *cIAP1*/*XIAP* DKO (*cIAP1/XIAP* DKO: *D*_max_ 28%, DC_50_ 220 nM) cell lines, suggesting that XIAP may be the principal E3 affecting *D*_max_ for XB2 **A538**. Quality control of our double KO’s revealed that *cIAP1 XIAP* double KO cells gradually lost *XIAP* KO status over time (Supplementary Fig. 3k). This suggests that loss of both these IAPs is deleterious for cells and is consistent with knockout studies in mice showing that loss of XIAP and cIAP1 results in embryonic lethality^49^, but this phenomenon limited more extensive testing of our compounds in double KO cells.

**Fig. 4:**
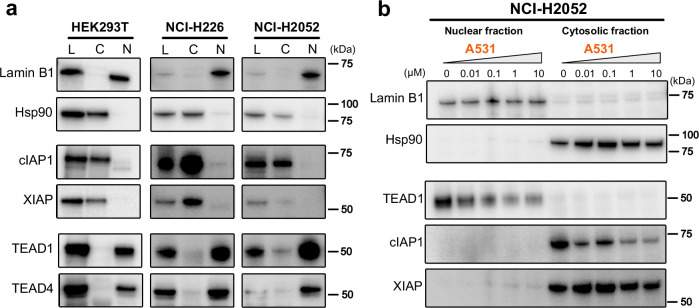
Localization of E3 ligases and POI in HEK293T and mesothelioma model cell lines. **a** Immunoblots of equivalent total cell lysate (L), cytosolic fraction (C) and nuclear fraction (N) from indicated cell lines were probed for nuclear marker (Lamin B1), cytosolic marker (Hsp90), E3 ligases (cIAP1 and XIAP) and target proteins (TEAD1 and TEAD4). **b** Subcellular profiling of endogenous TEAD1 and cIAP1 degradation. NCI-H2052 cells were treated with dose titration of ALP hit **A531** (four concentrations with 10-fold serial dilutions from 10 µM and DMSO vehicle control), nuclear and cytosolic fractions purified, and equivalent amounts run on Western blot. Antibodies against nuclear marker (Lamin B1), cytosolic marker (Hsp90), target protein (TEAD1) and E3 ligases (cIAP1 and XIAP) were used for probing the blots. All subcellular fraction experiments are performed as a single biological experiment (*n* = 1), but have at least *n* = 2 biologically independent experiments overall for TEAD1 and cIAP1/XIAP localization in NCI-H2052 cells (**a**, **b**). All uncropped blot images are available in Supplementary Data 1.

As our IPDs achieved incomplete TEAD1 degradation, we next examined the possible influence of the subcellular localization of either the target or IAP E3 ligase. It has been reported that relative target and E3 subcellular localization may in certain cases affect degrader efficacy; although CRBN and VHL (both typically predominantly cytoplasmic) can efficiently degrade nuclear proteins, including native substrates and PROTAC targets^51^. We probed endogenous TEAD1, TEAD4, cIAP1 and XIAP in purified nuclear and cytosolic fractions of HEK293T, NCI-H226 and NCI-H2052 cells. cIAP1 and XIAP were both localized to the Hsp90-containing cytosolic fraction, while, as anticipated, TEAD1 and TEAD4 localized to the Lamin B1-containing nuclear fractions in all three cell lines (Fig. 4a). Since our IPDs appeared to have better efficacy in the HiBiT assays, we also tested the localization of HiBiT-tagged TEAD1 and TEAD4 in the respective NCI-H2052 cell lines. These tagged and over-expressed TEADs localized in the nuclear fraction like the endogenous proteins (Supplementary Fig. 4a, b). We next examined the degradation profile of TEAD1 and cIAP1 localized in either purified cytosolic or nuclear fractions. NCI-H2052 cells were treated with ALP2 **A531** for 20 h followed by nuclear-cytosol purification (Fig. 4b). As before, TEAD1 was primarily nuclear localized and IAPs were cytosolic. A concentration-dependent decrease in endogenous TEAD1 was observed in nuclear fractions, while cIAP1 auto-degradation was observed in cytosolic fractions, indicating target degradation was not prevented by differential localization of TEAD and IAP E3 ligases (Fig. 4b).

In sum, our data therefore suggest that a component of the observable TEAD1 degradation for our IPDs occurs via a proteasome-and IAP-dependent mechanism, whilst the remainder occurs via a proteasome-dependent destabilization/degradation process that is IAP independent but requires direct TEAD binding, potentially by supercharging an endogenous degradation circuit^50^.

### Rigid linker variation on the IPD ALP2 series did not increase TEAD1 degradation

**Fig. 5:**
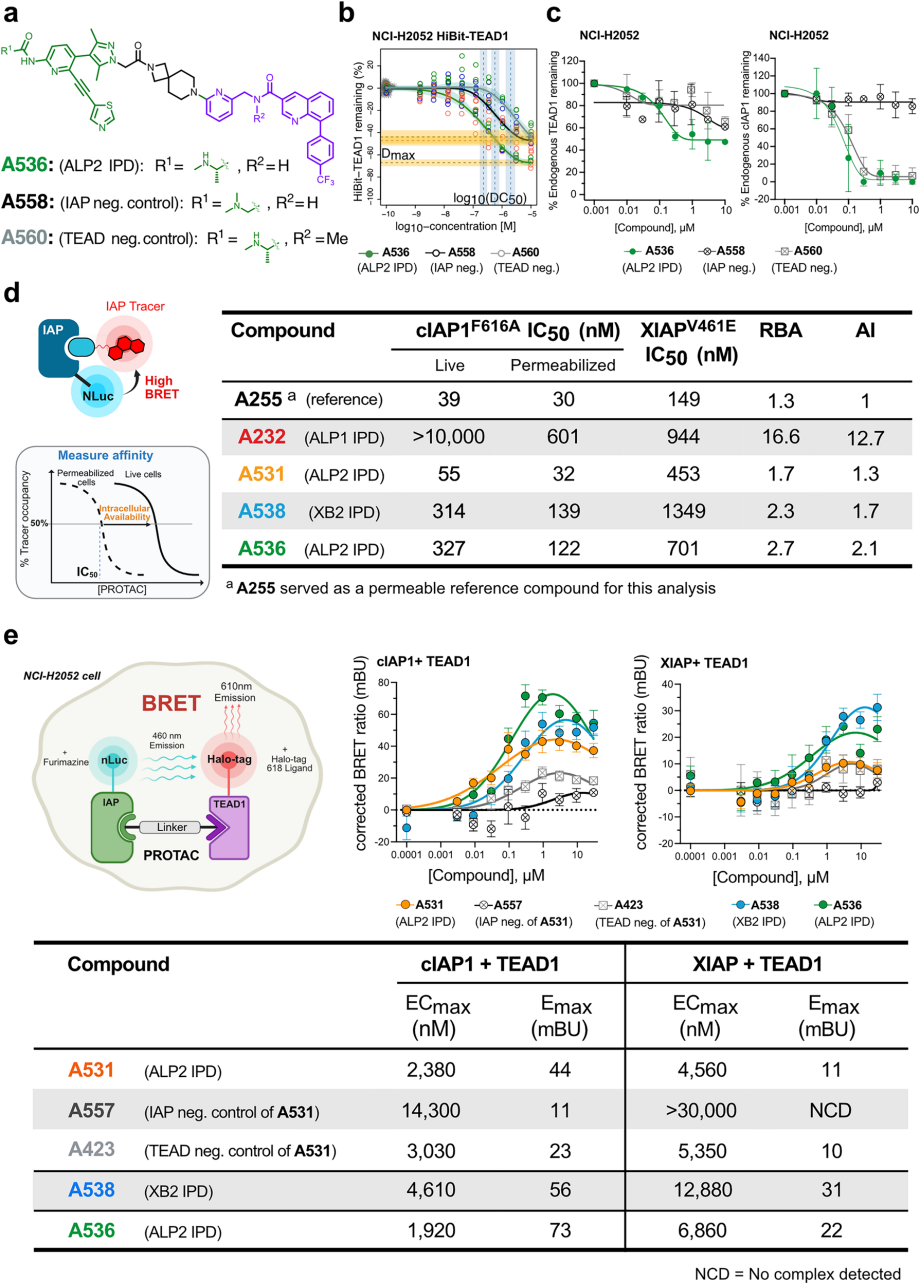
Profiling of rigid linker variation of ALP2 series and assessing IAP engagement, permeability and ternary complex formation by IPD hits. **a** Chemical structure of ALP2 series IPD **A536** incorporating a spirocyclic linker and matched IAP- and TEAD- negative controls (**A558** and **A560**, respectively). **b** Profiling of degradation of HiBiT-TEAD1 (NCI-H2052 cells) following 20 h dose–response treatment with ALP2 IPD (**A536**, green line) or matched IAP negative control (**A558**, black line) or TEAD1 negative control (**A560**, gray line) IPDs. HiBiT signal was normalized to CTG reading, and the HiBiT/CTG ratio was compared to a vehicle control to plot percentage of TEAD1 remaining. Plotted data represent individual data points from three independent biological experiments. Degradation DC_50_ and *D*_max_ were fitted as described^92^. **c** Capillary-based western profiling of endogenous TEAD1 degradation (left panel) and cIAP1 auto-degradation (right panel) in NCI-H2052 cells (dose response, 20 h) by ALP2 IPD **A536**, its matched IAP negative control, **A558** and TEAD negative control, **A560** (color scheme as in **b**). Uncropped blot images are available in Supplementary Data 1. Dose response curves are represented with each concentration denoting mean $\pm$ SD of *n* = 2 biologically independent experiments. **d** IAP cellular target engagement. A cellular IAP target engagement assay was developed based on the displacement of a fluorescent IAP tracer **B678** from NanoLuc-tagged cIAP1_184–618_^F616A^ or XIAP_124–497_^V461E^ (HEK293T cells) and nanoBRET signal measured for IAP binders or IPDs treated in dose–response (left panel, refer Supplementary Fig. 5d and Supplementary Information Synthetic Chemistry methods for synthesis of **B678**). Percentage tracer occupancy (based on nanoBRET signal, normalized to DMSO vehicle) was measured for live cells (cIAP1 and XIAP) or cells permeabilized by pre-treatment with digitonin (cIAP1 only). For cIAP1, a cellular Availability Index (AI) was determined by first comparing the fitted IC_50_ values in live and permeabilized modes to obtain a Relative intracellular availability (RBA) value, then normalizing this to ASX series IAP ligand **A255**, selected as a cell-permeable control compound with high affinity to BIR3 of cIAP1 and XIAP^47^. cIAP1 was used for AI determination as most compounds tested have potent cIAP1 binding. Larger AI values represent lower intracellular availability relative to the permeable control **A255**. Right panel: tabulated IC_50_, RBA and AI values for ASX series IAP binder reference **A255** and IPDs ALP1 **A232**, ALP2 **A531**, XB2 **A538** and ALP2 **A536**. **e** Cellular ternary complex formation. Cellular IAP/IPD/TEAD1 ternary complex formation was measured by treating NCI-H2052 cells co-expressing NanoLuc-tagged cIAP1_184–618_^F616A^/Halo-TEAD1 or NanoLuc-tagged XIAP_124–497_^V461E^/Halo-TEAD1 with a dose response of IPDs (ALP1 IPD **A531** and corresponding IAP or TEAD negative controls **A557** and **A423**, or XB2 IPD **A538**, or ALP2 IPD **A536**), alongside NanoGlo Substrate and HaloTag 618 ligand. Measured nanoBRET signals relative to background were fitted to a Gaussian distribution model to calculate *E*max and EC_max_ values for ternary complex formation with TEAD1 and either cIAP1 or XIAP. Data represent mean ± SD for *n* = 3 biologically independent experiments.

One strategy used to enhance the potency and selectivity of degraders involves linker rigidification that can help stabilize a productive POI-degrader-E3 ligase ternary complex^52–54^. Whilst our most potent IPD, ALP2 **A531**, already possessed a relatively short and rigid linker, we wanted to explore whether subtle alterations in rigid linker geometry might improve the maximal TEAD1 degradation achieved for this IPD. We therefore developed a spirocyclic IPD, ALP2 **A536** (Fig. 5a). Despite achieving similar degradation potency, the spirocyclic linker ALP2 **A536** did not, unfortunately, demonstrate a clear increase in HiBiT-TEAD1 or endogenous TEAD1 degradation *D*_max_ (HiBiT-TEAD1 *D*_max_ 67%, DC_50_ 233 nM; endogenous TEAD1 *D*_max_ 51%, DC_50_ 110 nM) compared to ALP2 **A531** (Fig. 5b, c). The IAP- or TEAD-binding negative control matched pairs of ALP2 **A536** also showed a similar window of IAP- and TEAD-dependent activity to **A531** (ALP2 **A558**: TEAD1 *D*_max_ 41%, DC_50_ 2400 nM and ALP2 **A560**: TEAD1 *D*_max_ 20%, DC_50_ 11 nM respectively, Figs. 5c, 3b, c and Supplementary Fig. 5a, b), and degradation was markedly reduced upon proteasome inhibition (Supplementary Fig. 5c). cIAP1 auto-degradation was not affected by this new linker (Fig. 5c and Supplementary Fig. 5a, b), with both ALP2 **A536** and the TEAD negative control ALP2 **A560** showing identical levels of cIAP1 degradation while the IAP negative control ALP2 **A558** left cIAP1 levels untouched (Fig. 5c).

### IPD hits are cell permeable and can form ternary complexes with IAPs and TEAD1

To better understand the plateau in maximal cellular TEAD1 degradation for our IPD series, we next sought to directly evaluate cellular IAP engagement, the cellular permeability of our IPDs and their ability to promote ternary complex formation with TEAD1. To do this, we established an IAP cellular nanoBRET assay based on NanoLuc-tagged IAPs (cIAP1^F616A^, XIAP^V461E^) and competitive displacement of a fluorescently tagged IAP tracer with high affinity for the BIR3 domains of both cIAP1 and XIAP. A similar IAP nanoBRET assay has been employed by others to examine cellular engagement of SMAC mimetics across the IAP family^55^. In our approach, the RING-domain point mutation in each IAP renders them monomeric and E3-deficient, thereby preventing IPD induced degradation of either the IAP or target^56–58^. IPDs were profiled in live cells or following permeabilization using digitonin, to determine an IC_50_ for cIAP1 or XIAP BIR3 engagement and an intracellular availability index (AI) (Fig. 5d), being the fold difference in cellular availability for cIAP1 engagement between permeabilized and live cells relative to the permeable ASX series IAP ligand **A255** (Supplementary Fig. 1d)^47^. This revealed that, relative to the weaker ALP1 **A232** with a flexible PEG-based linker, the ALP2 and XB2 IPDs with shorter, more rigid linkers (ALP2 **A531**, XB2 **A538** and ALP2 **A536**) showed better cell permeability and cIAP1 and XIAP engagement (Fig. 5d and Supplementary Fig. 5d), which was also consistent with the endogenous cIAP1 auto-degradation results (Fig. 3b, c for **A531**; Supplementary Fig. 3c for **A538** and Fig. 5c for **A536**). We next developed a cellular nanoBRET ternary complex assay by co-expressing Halo-tagged TEAD1 with NanoLuc-tagged cIAP1 or XIAP (Fig. 5e) and measuring nanoBRET signal in the presence of IPDs. This confirmed formation of a cellular ternary complex between cIAP1 and TEAD1 for all IPDs at concentrations consistent with observed degradation potency, whereas ternary complex formation was reduced for IAP- and TEAD-negative controls (Fig. 5e), consistent with loss of IAP and TEAD binding (Supplementary Fig. 5d, e). Notably, based on comparison of *E*_max_ and EC_max_, the ALP2 series IPDs **A536** and **A531** preferentially formed cIAP1-IPD-TEAD1 complexes, whilst the XB2 series IPD **A538** efficiently formed TEAD1 ternary complexes with both XIAP and cIAP1. This was consistent with the binding profile of the parent IAP binders and also the observed dependency on cIAP1 and XIAP for each series (Figs. 1b, 3g and Supplementary Fig. 1c).

### IAP-based degraders are selective for TEAD1 degradation

**Fig. 6:**
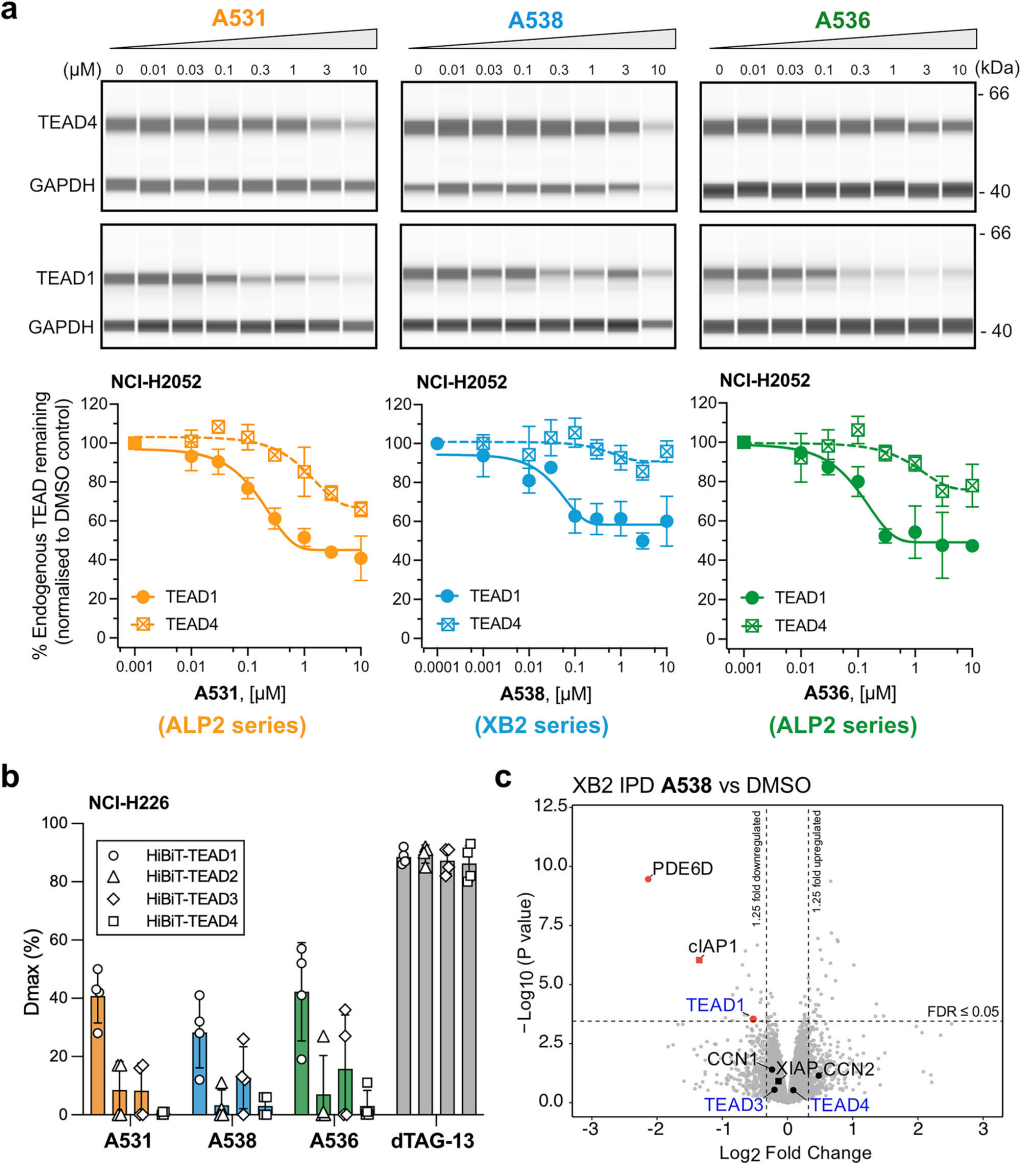
Specificity of endogenous TEAD degradation by IPDs. **a** Endogenous TEAD1 and TEAD4 degradation profiling in NCI-H2052 cells with 20 h treatment with ALP2 IPD **A531**, XB2 IPD **A538** and ALP2 IPD **A536**. With compound concentrations represented in increasing concentration from DMSO to 10 µM, Top panel shows representative capillary western blot of TEAD4 and loading control GAPDH; middle panel, blot of TEAD1 and GAPDH and bottom panel shows degradation dose response curves with each data point representing mean ± SD of *n* = 2 biologically independent experiments, except for *n* = 5 for **A531** (TEAD1) and *n* = 3 for **A538** (TEAD1). Uncropped blot images are available in Supplementary Data 1. **b** HiBiT assay measuring *D*_max_ of ALP2 IPD **A531**, XB2 IPD **A538** and ALP2 IPD **A536** after 18 h treatment of HiBiT-TEAD1–4 NCI-H226 transgenic cell lines, normalized to CTG viability assay. Compound dTAG-13 (heterobifunctional degrader of the FKBP12^F36V^ sequence incorporated in the TEAD1–4 transgenic constructs)^97^ was used as positive control. Data are representing mean ± SD of *n* = 4 biologically independent experiments, each with two technical replicates. **c** Global proteomic analysis of XB2 hit **A538** specificity in NCI-H2052 cells, treated with compound **A538** (0.5 µM, 16 h) or DMSO (*n* = 5 biological replicates). Volcano plots show relative protein abundance (log2 fold change) vs significance (−log10 *p*-value) of quantified proteins. Proteins significantly altered lie above the horizontal dashed line (adjusted *p*-value “or” FDR ≤ 0.05) and beyond vertical cut off lines (left, 1.25 times downregulated; right, 1.25 times upregulated in **A538** treated cells). A complete protein list is provided in Supplementary Data 2.

Our IPDs utilize a binder derived from pan-TEAD inhibitor **VT-107** as the TEAD ligand^10^. Palmitoylation of TEAD is critical for its stability, and **VT-107** inhibits palmitoylation of endogenous TEAD1 and TEAD3, but also potently blocks palmitoylation of TEAD4^10^. However, several studies have shown that bifunctional degraders can demonstrate unexpected specificity compared with their target protein ligands, often due to the structural constraints required to form an effective ternary complex^17,59–61^. To assess paralog specificity of endogenous TEAD degradation by our compounds, we compared degradation of TEAD1 and TEAD4, for which suitably specific antibodies are available. The TEAD1 and TEAD4 antibodies were each validated in NCI-H2052 *TEAD1* KO and HEK293T *TEAD4* KO cells, respectively (Supplementary Fig. 6a, b). Interestingly all three IPDs: ALP2 **A531**, XB2 **A538** and ALP2 **A536**, showed TEAD1 specificity with reduced TEAD4 degradation profiles giving a *D*_max_/DC_50_ of 33%/1100 nM (TEAD1 55%/170 nM), 9%/510 nM (TEAD1 42%/44 nM) and 24%/990 nM (TEAD1 51%/110 nM) respectively (Fig. 6a, Table 1 and Supplementary Fig. 5b).

Given the lack of suitable antibodies against TEAD2 and TEAD3 and considering the fluctuations in endogenous TEAD1–4 expression across different cell lines, we then generated HiBiT-TEAD1–4 transgene insertions in the NCI-H226 cell line and used it to measure the selectivity of our compounds on all TEAD paralogs (Fig. 6b and Supplementary Fig. 6c, *D*_max_ and DC_50_ values in Supplementary Data 1). These assays confirm the relative TEAD1 specificity of the IPDs ALP2 **A531**, XB2 **A538** and ALP2** A536**, displaying minimal activity for TEAD2 and TEAD4 and, for **A538** and **A536**, weak degradation activity for TEAD3 (*D*_max_ = 13% and 16%, respectively). Analysis of matched IAP and TEAD negative control compounds also confirmed TEAD1 specificity, albeit at lower degradation levels compared to the respective IPD hits (Supplementary Fig. 6c), complementing the endogenous degradation data. Overall, whilst these degraders all utilize a similar TEAD ligand (derived from a reportedly pan-TEAD inhibitor), they preferentially degrade TEAD1, with subtle variations in TEAD specificity between each IPD potentially reflecting the constraints of ternary complex formation.

To assess the effect of our TEAD IPDs on global protein abundance, we treated NCI-H2052 cells for 16 h with our most potent hit XB2 **A538** and compared it with DMSO (Fig. 6c), matched IPD and TEAD negative controls **A559** and **A561**, respectively (Supplementary Fig. 6d) and analysed lysates by LC-MS-based proteomics (data in Supplementary Data 2). Whole cell proteomics analysis confirmed TEAD1 specificity over TEAD3 and TEAD4 in all three comparisons (TEAD2 was undetected). TEAD1 was one of the significantly downregulated proteins in XB2 hit **A538** treatment (Fold change −1.4; adjusted *p* value/FDR = 0.045) when compared to DMSO treated cells (Fig. 6c). In **A538** treatment compared to matched IAP- and TEAD-negative control IPDs, TEAD1 also remained significantly downregulated (fold change values of −1.3 in both sets; adjusted *p* value/FDR = 0.042 and 0.044 respectively).

cIAP1 was one of the most significantly downregulated proteins upon **A538** treatment, whether relative to either treatment with DMSO (Fig. 6c), or IAP negative control **A559** (left plot in Supplementary Fig. 6d). This confirmed IAP engagement and auto-degradation induced by IPD **A538**, while also validating the lack of IAP engagement in the IAP negative control. As expected, cIAP1 downregulation was absent when analyzing **A538** treatment relative to the matched TEAD-negative control **A561** (right plot in Supplementary Fig. 6d), each of which can bind cIAP1 equivalently. PDE6D (retinal rod rhodopsin-sensitive cGMP 3′,5′-cyclic phosphodiesterase subunit delta), previously reported as an off-target protein degraded with PTK2^62^ and LRRK2 PROTACs^63^, was the most significantly downregulated protein in **A538** treatment compared to DMSO. Incidentally, it was also the second most downregulated protein when compared to matched IAP-negative control treatment, but not the TEAD-negative control treatment (Supplementary Fig. 6d). This supports a previous observation for TEAD-directed degraders that PDE6D is a potential off target for TEAD lipid binding pocket compounds^64^, now validated with a TEAD negative control in our study. H3-4 (Histone H3.4) was the most downregulated protein in **A538** treatment when compared to IAP-negative and TEAD-negative control treatment. H3K4 methylation is important for activation of Hippo target genes^65,66^, and downregulation of H3-4 could putatively indicate a secondary effect of TEAD degradation. Alternatively, this could be a change induced by the antiproliferative effects induced by TEAD inhibition in this specific cell line and linked to the remodeling of the chromatin landscape following alterations of the cell cycle. Overall, the presence of other downregulated proteins, at least some of which are putative TEAD1 targets (complete list in Supplementary Data 3, 4 and 5), may reflect secondary effects of pathway downregulation. Given the target protein TEAD1 is a transcription factor, and our kinetics data indicated XB2 IPD **A538** to be a slow degrader (Supplementary Fig. 6f), we do expect a secondary effect on the cellular proteome due to longer treatment time (16 h) used in this analysis. Generally, shorter treatment times are preferred in PROTAC global proteomic analysis (5–8 h) to avoid detection of indirect effects of target protein degradation on the cellular proteome. Amongst TEAD canonical targets altered were CCN1 and CCN2 (encoded by *CYR61* and *CTGF* genes, respectively). Notably, CCN1 downregulation upon IPD **A538** treatment reached significance only when compared to matched TEAD-negative control (Fold change −1.3; adjusted *p* value 0.025), not the DMSO treatment (Fig. 6c and Supplementary Fig. 6d), highlighting the critical role of an appropriate negative control in evaluating PROTAC specificity. In addition, four significantly downregulated proteins were commonly identified across all three comparisons (See ‘Common downregulated proteins’ in Supplementary Data 2), including the target protein TEAD1. Notably, the other three proteins were VGLL3 (vestigial like family member 3, a known co-factor of TEAD proteins^67^), LOX (Lysyl oxidase, YAP/TAZ/TEAD transcriptional target^68^ influencing extracellular matrix stability), CAVIN2 (Cavin protein 2, functionally dependent on isoform CAVIN1, a known YAP/TAZ-TEAD target gene essential for caveolae formation^69^), all functionally related to TEAD1 or the Hippo pathway. This indicates common biological signatures associated with TEAD1 downregulation in IPD **A538** treatment, regardless of the control used (DMSO or E3 ligase IAP or target TEAD binding controls). Overall, despite the limitations of the global proteomics data for **A538**, the observed TEAD paralog selectivity profile correlates with that of the HiBiT TEAD1–4 data, supporting the degradation specificity of this compound for TEAD1 relative to other TEAD paralogs.

### Functional downstream analysis of IPD hits on cell proliferation and Hippo pathway

**Fig. 7:**
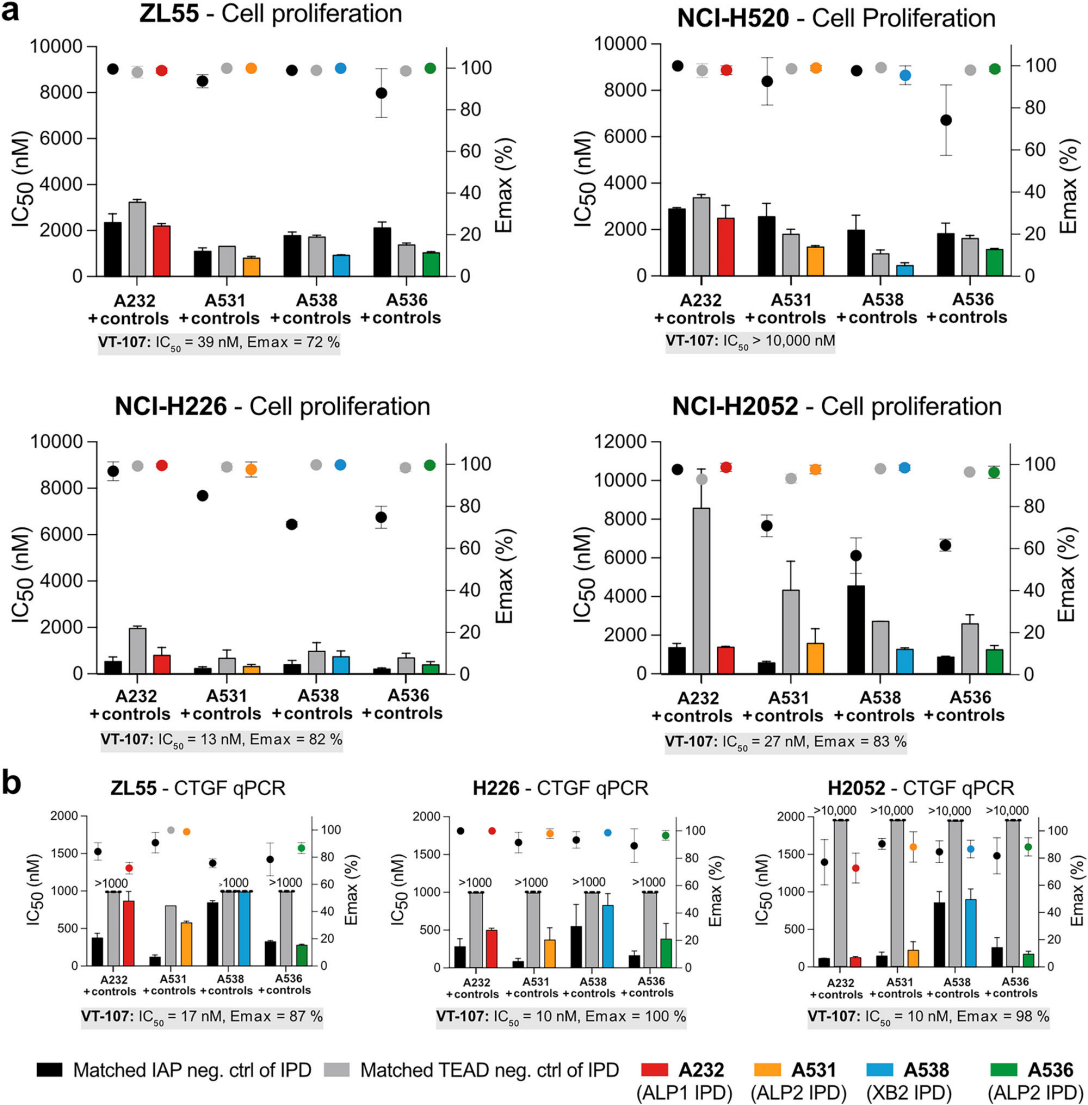
Analysis of TEAD-based IAP degrader activity on proliferation and transcription. **a** Viability of Hippo pathway-dependent mesothelioma cells ZL55, NCI-H226, NCI-H2052 and Hippo pathway-independent NCI-H520 cells was assessed post dose titrations of indicated IPDs for 7 days. IC_50_ (plotted as bars) and maximum inhibition % *E*max (plotted as dot symbols) values were calculated from dose response curves (see Supplementary Data 1) for indicated cell lines. Data represent mean ± SD of *n* = 4 biologically independent experiments. **b** Effect of IPDs on transcript levels of TEAD-dependent genes. qPCR analysis of *CTGF* expression level (top panel) from indicated cell lines treated with 5 concentration points (1:10 dilution factor from 10 µM and DMSO) for 48 h. IC_50_ and *E*_max_ values were calculated as mean ± SD of *n* = 3 biologically independent experiments (*n* = 2 in the case of ZL55 cells). IC_50_ values marked >1000 and >10000 nM indicate the upper concentration limit for dose response fitting, as detailed in “Materials and Methods”.

To assess the effects of TEAD1 degradation induced by our IPDs, we analyzed cellular proliferation in human mesothelioma cell lines ZL55, NCI-H226 and NCI-H2052, that are dependent on TEAD activity, as compared to a Hippo pathway independent cell line, NCI-H520. All four cell lines were treated with dose titrations of the IPDs ALP1 **A232**, ALP2 **A531**, XB2 **A538**, ALP2 **A536** and the matched IAP- and TEAD-negative control compounds, and cell proliferation analyzed by CellTiter Glo assay after 144 h treatment. As expected, the TEAD inhibitor **VT-107** showed activity in the NF2-deficient ZL55 line (ZL55 IC_50_ = 39 nM) and not in the Hippo pathway-independent NCI-H520 line (NCI-H520 IC_50_ > 10,000 nM) (Fig. 7a). In the ZL55 cell line, the IPDs ALP2 **A531**, XB2 **A538** and ALP2 **A536** had a weaker overall antiproliferative effect than the TEAD inhibitor **VT-107** (refer IC_50_ values in Fig. 7a and Table 2), consistent with their profiling as partial TEAD1 degraders, whereas **VT-107** is proposed to target efficiently all TEAD paralogs^10^. Similarly, ALP2 **A531**, XB2 **A538** and ALP2 **A536** have weaker antiproliferative activity compared to the TEAD inhibitor **VT-107** in the NCI-H2052 and NCI-H226 cell lines, although the IC_50_ values are overall more potent in NCI-H226 than in ZL55 cells, reflecting a higher degree of dependency of this cell line on the transcriptional output of the Hippo pathway. Moreover, the fact that matched IAP negative controls have largely comparable efficacy to the IPDs suggests that the antiproliferative effect of the compounds derives largely from allosteric modulation of TEAD activity rather than protein degradation activity. This is also confirmed by the analysis of the Hippo pathway modulation, which was assessed by qPCR of the TEAD target gene *CTGF*, which was expected to reduce in response to IPD-dependent TEAD1 degradation. Again, all four IPDs and their matched negative controls were used for dose treating Hippo pathway dependent lines ZL55, NCI-H226 and NCI-H2052 for 48 h. In all three cell lines ALP2 **A531**, XB2 **A538** and ALP2 **A536** induced a weaker modulation of *CTGF* expression levels compared to the TEAD inhibitor **VT-107**, and the effects were comparable to the respective IAP negative controls, ALP2 **A557**, XB2 **A559** and ALP2 **A558** (Fig. 7b and Table 3). Conversely, the pathway modulation effect was strongly suppressed for the corresponding TEAD-negative control compounds, ALP2 **A423**, XB2 **A561** and ALP2 **A560**, pointing to a TEAD-dependent and at least partially IAP-independent effect. Overall, the data suggest that most of the effects of the IPDs on cell proliferation and pathway inhibition result from inhibition of TEAD activity rather than TEAD degradation, and more potent compounds are likely needed to harness the full potential of TPD-mediated pathway inhibition. The weaker effects of IPDs compared to the parent TEAD binder **VT-107** can likely be explained by a combination of modest loss in binary TEAD affinity and cell permeability (Supplementary Figs. 1h and 5e), reduced target accessibility, and primarily TEAD1-restricted degradation selectivity (Fig. 6b), as compared to the broader targeting of all TEAD paralogs described for **VT-107**^10^.

**Table 2:** Cellular proliferation in mesothelioma and non-mesothelioma cells lines with IPD hits and negative controls

Compound	Description	ZL55		NCI-H226		NCI-H2052		NCI-H520	
		IC_50_ (nM)	*E*_max_ (%)	IC_50_ (nM)	*E*_max_ (%)	IC_50_ (nM)	*E*_max_ (%)	IC_50_ (nM)	*E*_max_ (%)
**VT-107**	TEAD binder	39	72	13	82	27	83	>1000	N.V.
**A232** **A230** **A231**	ALP1 IPD hit ALP1 IAP neg. control ALP1 TEAD neg. control	2194 2344 3225	99 100 98	798 531 1955	100 97 99	1375 1353 8561	99 98 93	2491 2888 3370	98 100 98
**A531** **A557** **A423**	ALP2 IPD hit ALP2 IAP neg. control ALP2 TEAD neg. control	798 1096 1312	100 94 100	310 227 670	98 85 99	1573 563 4325	98 71 93	1243 2555 1793	99 93 99
**A538** **A559** **A561**	XB2 IPD hit XB2 IAP neg. control XB2 TEAD neg. control	925 1777 1709	100 99 99	733 398 967	100 72 100	1267 4545 2711	99 57 98	443 1969 955	96 98 99
**A536** **A558** **A560**	ALP2 IPD hit ALP2 IAP neg. control ALP2 TEAD neg. control	1026 2111 1373	100 88 99	386 207 690	100 75 98	1250 868 2589	96 62 97	1137 1821 1609	98 74 98

Cell viability post 144 h compound treatment (10–0.001 µM) assessed using Cell Titer-Glo assay. Data are means of *n* = 4 biologically independent experiments for all cell lines except NCI-H2052 (*n* = 3). Decimal values are rounded to nearest whole number.

*N.V.* no value.

**Table 3:** Effect on *CTGF* gene transcription in response to TEAD IPDs and negative controls

Compound	Description	ZL55		NCI-H226		NCI-H2052	
		IC_50_ (nM)	*E*_max_ (%)	IC_50_ (nM)	*E*_max_ (%)	IC_50_ (nM)	*E*_max_ (%)
**VT-107**	TEAD binder	17	87	10	100	10	98
**A232** **A230** **A231**	ALP1 IPD hit ALP1 IAP neg. control ALP1 TEAD neg. control	866 375 >1000	72 84 N.V.	497 281 1211	100 100 N.V.	125 111 >10000	73 77 N.V.
**A531** **A557** **A423**	ALP2 IPD hit ALP2 IAP neg. control ALP2 TEAD neg. control	576 120 801	99 91 N.V.	369 85 8862	98 92 N.V.	225 152 >10000	87 91 N.V.
**A538** **A559** **A561**	XB2 IPD hit XB2 IAP neg. control XB2 TEAD neg. control	>1000 844 >1000	N.V. 76 N.V.	826 547 >1000	99 93 N.V.	875 824 >10000	89 86 N.V.
**A536** **A558** **A560**	ALP2 IPD hit ALP2 IAP neg. control ALP2 TEAD neg. control	278 324 >1000	87 78 N.V.	380 162 2612	93 90 N.V.	170 256 >10000	88 82 N.V.

qPCR of *CTGF* gene was done to assess transcript levels post 48 h compound treatment. (10–0.001 µM, 5 points with 10-fold serial dilution). Data are means of *n *= 3 biologically independent experiments for NCI-H226 and NCI-H2052 and *n *= 2 for ZL55. Decimal values are rounded to nearest whole number.

*N.V.* no value.

## Discussion

In this study, we developed IAP-recruiting degraders (IPDs) targeting the palmitoylation pocket of TEAD1, harnessing a variety of IAP ligands with differing capacity to recruit the E3 ligases cIAP1/2 or XIAP. We identified IPDs with nanomolar range degradation DC_50_ for TEAD1 and relative selectivity for endogenous TEAD1 over TEAD4. However, despite screening a large number of IPDs generated from a panel of IAP binders and exploring both different IAP exit vectors and different linker compositions, these IPDs remained partial degraders of TEAD, with a plateau in the achievable endogenous TEAD1 *D*_max_ at around 40–60%. With the aim of developing optimized degraders, we performed additional detailed studies to better understand the IAP-based activity of these IPD hits and the reason for this plateau.

Poor compound cellular permeability is a major impediment to cellular activity, in particular for relatively large chemical molecules such as bifunctional degraders. Additionally, few cellular assays exist for measuring and comparing TEAD P-site ligand binding. We therefore developed cellular nanoBRET assays for cIAP1, XIAP and TEAD1 that allow assessment of cellular permeability and target engagement. We also report the development of an IAP-IPD-TEAD1 ternary complex formation assay. Together, these approaches showed that our IPDs ALP2 **A531**, XB2 **A538** and ALP2 **A536** had acceptable cellular uptake comparable to permeable control **A255** and access to their targets in cells (approximately 1.3–2.1-fold lower uptake based on AI value relative to **A255**, Fig. 5d), while the ternary complex assay confirmed that they engage both TEAD1 and IAPs simultaneously. We also validate robust negative control IPD matched pairs and show that these have either substantial (>20x) or complete loss of binding for TEAD- and IAP-negative controls, respectively.

A unique feature of IAPs relative to most E3 ligases hitherto harnessed for TPD is the ability to recruit *via* one or more E3 ligases, notably cIAP1 and XIAP. It is mechanistically well established that a corollary of cIAP1 engagement, principally via the BIR3 domain, is the activation of its ubiquitin ligase function that results in its autoubiquitination and proteasomal degradation. In comparison, XIAP is typically not activated in the same way and is therefore not degraded upon ligand binding to its BIR2 or BIR3 domains. IPD-induced destruction of cIAP1 potentially imposes a limit on the function of an IPD, and we therefore deliberately explored IPDs, which, in addition to cIAP1, could recruit XIAP via either BIR2 or BIR3. Analysis of IAP binding, ternary complex formation and TEAD degradation upon knockout of individual IAP members revealed a consistent picture whereby ALP2 IPDs preferentially degrade TEAD1 by recruiting cIAP1, whilst the XB2 series IPD **A538** harnesses both cIAP1 and XIAP activity. Amongst our hits, ALP2 **A531** and **A536** had more rigid linkers and displayed enhanced ternary complex formation. In this case, stabilization of the ternary complex did not appear to noticeably enhance IAP-dependent degradation of TEAD. Interestingly, it has been observed by others developing cIAP1-recruiting degraders of Bruton’s tyrosine kinase that identification of degraders with increased ternary complex stability did not necessarily correlate with increased degradation efficiency, although the contribution of other IAP family members was not assessed^28^. It has also previously been reported for IAP recruiting BRD4-degraders that XIAP is required and can be co-degraded alongside the target^46^. Yet, XIAP levels have also been reported to be transcriptionally affected by BRD4 inhibition, which may confound such effects^70^. In our studies for TEAD1, we observed no significant degradation of XIAP for either ALP or XB2 compounds, suggesting that simultaneous loss of XIAP by IPDs may be target dependent.

To understand the cellular plateau we observed in IPD-induced TEAD1 degradation, *D*_max_, we also considered TEAD turnover rate. A plateau in *D*_max_ might occur if a target is naturally continuously synthesized but rapidly degraded. In such cases, a fast degrader is typically required to overcome the fast resynthesis rate and achieve a high *D*_max_^71^. Our data support that TEAD1 is a relatively long-lived protein (half-life >8 h) (Supplementary Fig. 6e), whilst our kinetic profiling of the rate of TEAD1 degradation by our IPDs indicates they are slow degraders, with depletion of HiBiT-tagged TEAD1 occurring over a period of multiple hours (Supplementary Fig. 6f). Our cellular data confirm that our TEAD IPDs profiled can recruit via cIAP1 and suggest a dependency on cIAP1 for maximal TEAD1 degradation. However, as we demonstrate, these molecules also simultaneously promote cIAP1 auto-degradation, which we believe together contribute to the observed plateau in *D*_max_. Notwithstanding this, highly potent IAP-recruiting degraders of RIPK2 have been developed based upon ligands with high cIAP1 affinity^16,27^. This likely in part reflects target-specific differences. Although RIPK2 also has a long half-life (typical half-life >50 h primary immune cells)^72,73^ and slow rate of resynthesis following degrader treatment^16^, RIPK2 is also known to be a natural substrate for XIAP ubiquitination^74^, and additionally, inhibitor-induced RIPK2 degradation has recently been reported to be associated with both RIPK2 multimerization and the E3 ligase activity of cIAP1 and XIAP^50^. As IAPs function as active dimers, an intriguing possibility is that multimeric proteins might represent advantageous targets for IAP-recruiting degraders. In the case of TEADs, whilst our data suggest that our IPDs can engage both IAPs (predominantly cytosolic) and TEAD1 (predominantly nuclear), it also remains possible that differential localization may hinder efficient degradation. Further studies (and validated E3 recruiters) would be required to delineate whether a predominantly nuclear-localized E3 ligase might offer enhanced capacity for TEAD1 degradation.

Recently, TEAD degraders targeting the P-site via recruitment of the CRL4^CRBN^ E3 ubiquitin ligase complex have been described^64,75,76^ with reported selectivity to degrade TEAD1/3^75^ or TEAD2^76^ paralogs. Another recent publication^77^ reports a potent CRBN-based PROTAC that utilizes a YAP-TEAD protein-protein interaction inhibitor and demonstrates in vitro near pan TEAD degrader activity against TEAD1, 3, 4 (TEAD2 expression was too low, and the antibody described has been discontinued) and in vivo degradation of TEAD1. The current lack of specific antibodies for all four TEAD paralogs complicates the study of endogenous degradation selectivity. As a result, epitope tagging strategies, or in some cases even pan-TEAD antibodies^76^, have typically been used to monitor TEAD degradation. However, epitope-tagged transgenes have some limitations—most notably, the potential for the tag to affect protein stability, or the expression level of the tagged protein to alter degradation rates—necessitating careful validation^71^. Additionally, even highly similar protein paralogs can vary significantly in degrader-induced degradation selectivity and kinetics, as illustrated for paralogs of the BET bromodomain family^61,78^. In our study, both HiBiT-tagged TEAD1 and endogenous TEAD1 assays identified the same potential hits. However, we observed that degradation was consistently stronger in HiBiT assays. For example, for the lead ALP series compounds **A531** and **A536**, we consistently observed around 70% degradation for HiBiT-TEAD1, compared to 50–55% for endogenous TEAD1 across isogenic cell lines. These findings underscore the importance of also benchmarking with endogenous protein profiling to accurately assess degrader activity.

To evaluate TEAD paralog degradation selectivity, we therefore utilized complementary strategies employing, where possible, both tagging and endogenous protein analysis. Using the NCI-H226 cell line carrying the insertion of a HiBiT-FKBP12^F36V^-TEAD transgene for each of the TEAD1–4 paralogs, we could assess the activity of the degraders against each of them, while also measuring the degradation of the endogenous TEAD1 protein. Despite using a highly similar TEAD binder based on the **VT-107** compound, originally described as having broader TEAD-binding activity^10^, all of our degraders displayed preferential activity against TEAD1. This is most likely linked to the efficiency of ternary complex formation leading to proficient degradation of the target, and could depend on small differences in the accessibility of the PROTAC molecules bound to the palmitoylation pocket of the different TEAD paralogs. The TEAD1 specificity of one of our IPDs (XB2 **A538**) was also validated in a global proteomic analysis comparing it with DMSO and matched IAP- and TEAD-negative control IPDs. TEAD1 was significantly downregulated (at >1.25 fold) in all three comparisons over TEAD3 and TEAD4 (TEAD2 undetected in MS). In addition, multiple significantly downregulated proteins observed were indicative of potential off-targets (PDE6D) or secondary effects linking to Hippo pathway modulation post downregulation of TEAD1. The extent of pathway modulation and the resulting antiproliferative effects depend on the capacity of targeting multiple TEAD paralogs and inhibiting their function to a sufficient extent. TEAD1 targeting is critical since it is a major tumor driver, as highlighted by DEPMAP analysis^79–81^, and a restricted specificity profile could have a positive impact in terms of anticipated tolerability. Conversely, to avoid compensation mechanisms by other TEAD paralogs, or in general to increase efficacy, targeting additional paralogs would be beneficial. Therefore, designing IPDs with higher TEAD1 lipid binding pocket affinity or broader paralog specificity, along with faster degradation kinetics would likely show better efficacy in the downregulation of the Hippo pathway and significant antiproliferative effects across different mesothelioma cell lines and beyond.

Our work also suggests some valuable observations for TEAD degraders targeting the P-site. Interestingly, somewhat similar to published data for other reported TEAD degraders utilizing the P-site^64,75,76^, TEAD degradation appeared incomplete, with a fraction of TEADs remaining detectable even at the highest degrader concentrations. While it is beyond the present study to evaluate in detail, it is possible there may be a reservoir of TEAD where the P-site is poorly accessible for ligand/IPD binding, which may have broader relevance for degraders targeting this site^64,75,76^. For our P-site directed IPDs, our data showed that TEAD1 degradation is completely proteasome dependent, yet robust matched IAP-negative controls as well as IAP knockout experiments suggest that a component of the observed degradation is independent of IAPs. Our data suggest that while inhibitors of the TEAD1 palmitoylation pocket cause modest degradation of TEAD1, the degradation can be promoted to a greater extent using bifunctional degraders.

In summary, our study provides some important learnings and an assay toolbox for future development of TEAD degraders targeting the palmitoylation pocket, as well as for IAP-recruiting degraders for other targets beyond TEAD.

## Methods

### Chemical synthesis

Chemical synthesis is described in the Supplementary Information.

### Protein expression

#### Recombinant production of IAP BIR domains

BIR3 domains of cIAP1 and XIAP. The BIR3 domain of cIAP1 (UniProt Q804E2, residues 266–344 for TR-FRET assays) and XIAP (UniProt P98170, residues 241–361 for TR-FRET assays and residues 249–354 for crystallography) were cloned into a pGEX-6P3 expression vector containing a 3C protease cleavable N-terminal GST-tag for affinity purification (gift of Catherine Day lab, University of Otago). For crystallography, the BIR3 domain of cIAP1 (UniProt Q804E2, residues 260–352) was cloned into a pGEX expression vector containing a TEV protease cleavage GST-tag. Proteins were recombinantly expressed in *Escherichia coli* C41 (DE3) cells, whereby cultures were grown at 37 °C until an optical density of 1.0 was reached, then cooled to 16 °C before inducing protein expression with 0.5 mM IPTG at 16 °C for 16 h. Cells were harvested by centrifugation and stored at −80 °C.

Cells were thawed and resuspended in GST-lysis buffer (50 mM HEPES pH 7.5, 500 mM NaCl, 10 mM DTT, 5% glycerol) supplemented with lysozyme (10 mg), DNAse-I (0.5 mg) and 1 tablet of c0mplete^TM^ EDTA-free Protease Inhibitor Cocktail (Roche) and incubated at 24 °C for 10 min under constant agitation. Cells were lysed on ice by sonication (40% amplitude for 10 s and 10 s on ice, for 2 min). Cell debris was pelleted by centrifugation at 40,000 × *g* at 4 °C for 45 min, and supernatant was filtered through a 0.45 mm syringe filter before loading onto a gravity flow column packed with glutathione resin (GenScript), equilibrated with GST-lysis buffer, for affinity purification. GST-tagged protein was eluted from the resin with GST-lysis buffer supplemented with 5 mM reduced L-glutathione (Sigma-Aldrich). GST-IAP-BIR3 proteins were further purified by SEC (HiLoad Superdex 75 pg column, Cytiva) equilibrated in SEC Buffer (20 mM HEPES pH 7.5, 150 mM NaCl, 0.5 mM TCEP). Purified protein was concentrated using an Amicon Ultra-15 Centrifugal Filter Unit (10 kDa MWCO, Millipore) to 2.8 mg/mL (GST-cIAP1-BIR3) and 16 mg/mL (GST-XIAP-BIR3), flash frozen in liquid nitrogen and stored at −80 °C for assays. For crystallography, GST-tagged 3C Protease or His-tagged TEV protease was added to GST-XIAP-BIR3 or GST-cIAP1-BIR3, respectively, in a 1:10 molar ratio to cleave off the GST-tag, then GST removed by passing over glutathione resin and further purified by SEC as previously described.

BIR2 domain of XIAP. The BIR2 domain of XIAP (UniProt P98170, residues 152–236) was cloned into a modified pCold IV (Takara Bio) expression vector containing 3C protease cleavable N-terminal His and GST tags and TEV cleavable AviTag sequence. Using this vector as a template, site-directed mutagenesis was performed using Phusion polymerase to introduce point mutations, C202A and C213G, to improve protein behavior^82^. Both WT and mutant XIAP-BIR2 proteins were recombinantly expressed in *Escherichia coli* BL21 (DE3) cells, whereby cultures were grown at 37 °C until an optical density of 1.0 was reached, then cooled to 16 °C before inducing protein expression with 0.5 mM IPTG at 16 °C for 16 h. Cells were harvested by centrifugation and stored at −80 °C.

Cells were thawed and resuspended in His Buffer A (50 mM HEPES pH 7.5, 500 mM NaCl, 10 mM imidazole, 2 mM beta-mercaptoethanol) supplemented with lysozyme (10 mg), DNAse-I (0.5 mg) and 1 tablet of c0mplete^TM^ EDTA-free Protease Inhibitor Cocktail (Roche) and incubated at 24 °C for 10 min under constant agitation. Cells were lysed on ice by sonication (40% amplitude for 10 sec and 10 s on ice, for 2 m). Cell debris was pelleted by centrifugation at 40,000 × *g* at 4 °C for 45 m and supernatant was filtered through a 0.45 mm syringe filter before loading onto a gravity flow column packed with C0mplete His-Tag Purification Resin (Roche), equilibrated with His Buffer A, for affinity purification. A step-wise wash of the resin was performed with increasing concentrations of imidazole (25, 50, and 100 mM). His-tagged protein was eluted from the resin with His buffer A supplemented with 250 mM imidazole.

WT His-GST-XIAP-BIR2 protein was further purified by anion exchange chromatography (MonoQ 5/50 GL, Cytiva) equilibrated in Anion Exchange Buffer A (20 mM HEPES pH 7.5, 0.5 mM TCEP) and eluted across a gradient of 0–500 mM NaCl. Purified protein was concentrated using an Amicon Ultra-15 Centrifugal Filter Unit (10 kDa MWCO) to 0.71 mg/mL, flash frozen in liquid nitrogen and stored at −80 °C for TR-FRET assays. The mutant XIAP-BIR2(C202A,C213G) protein was incubated with His-tagged TEV protease in a 1:10 molar ratio to remove all tags (His, GST, AviTag). The His-tagged TEV protease and cleaved tags were removed by passing over nickel resin as previously described. Then, XIAP-BIR2 (C202A, C213G) was further purified by size exclusion chromatography (HiLoad Superdex 75 pg column, Cytiva). Purified protein was concentrated using an Amicon Ultra-15 centrifugal unit (3 kDa MWCO, Millipore) to 9.9 mg/mL, flash frozen in liquid nitrogen and stored at −80 °C for SPR assays.

### Biophysical IAP binding assays

#### Competitive IAP binding assay by time-resolved förster resonance energy transfer (TR-FRET)

TR-FRET assays used to assess IAP binding were carried out in white 384-shallow well ProxiPlates (Revvity, 6008289). All TR-FRET assays were conducted in triplicate in buffer containing 50 mM Tris pH 7.4, 150 mM NaCl, 0.05% Tween-20, 0.1% BSA, 1 mM DTT. A 10-point 5-fold serial dilution of compounds were dispensed using an Echo® 555 Liquid Handler (LabCyte) from 10 mM DMSO stocks (final compound concentration range 10 µM–5 nM, plus DMSO-only and background no-protein control wells). For TR-FRET assays for cIAP1-BIR3 and XIAP-BIR3, detection reagents were used at a final concentration of 3 nM LANCE Europium-labeled streptavidin (Revvity, AD0062) and 5 nM LANCE Ultra ULight Anti-GST antibody (Revvity, TRF0104) utilized a biotinylated SMAC peptide tracer (H-AVPIAQKSE-Lys(Biotin)-NH_2_, Mimotopes) (final concentrations 3.3 and 10 nM, respectively) and GST-tagged protein (final concentrations 0.37 nM of GST-cIAP1-BIR3 and GST-XIAP-BIR3, respectively). The TR-FRET assay for XIAP-BIR2 utilized a custom tracer (**A191**, Supplementary Information Synthetic Chemistry) (final concentration 45 nM) and WT His-GST-AviTag-XIAP-BIR2 (final concentration 45 nM) and 12 nM LANCE Europium-labeled streptavidin (Revvity, AD0062) and 45 nM LANCE Ultra ULight Anti-GST antibody (Revvity, TRF0104). After the addition of all reagents, plates were incubated at room temperature (RT) for 1 h, and the FRET signal was measured with a CLARIOstar^Plus^ plate reader (BMG Labtech) (EX TR excitation filter at 337 nm, LP TR dichroic mirror, 665-10 and 620-10 emission filters). The percentage of maximum signal generated by the tested compounds was calculated according to the following equation:

$${{\mathrm{Percentage}}}\,{{\mathrm{of}}}\,{{\mathrm{Maximum}}}\,{{\mathrm{Signal}}}=100\,\times \,\left(\frac{T-{\mu }_{{{\rm{L}}}}}{{\mu }_{{{\rm{H}}}}-{\mu }_{{{\rm{L}}}}}\right)$$

Where $T=$ TR-FRET signal of the wells containing compounds, ${\mu }_{{{\rm{L}}}}=$ mean TR-FRET signal from the background control wells and ${\mu }_{{{\rm{H}}}}=$ mean TR-FRET signal from the 0% inhibition DMSO-only control wells. The data were plotted on GraphPad Prism 9.5.1 and fit to a 4-parameter logistic curve to determine the half-maximal inhibitory concentration (IC_50_).

#### Direct IAP binding assay by surface plasmon resonance (SPR)

Relevant IAP BIR domains (each 100 µM) were biotinylated using EZ-Link NHS-PEG4-biotin (Thermo Fisher Scientific, A39259) at a 1:1 molar ratio for 1 h at RT in 20 mM HEPES, 150 mM NaCl, 1 mM TCEP. Excess biotin was removed by passing over a Zeba Spin Desalting column (7K MWCO, 0.5 mL, Thermo Fisher Scientific) into fresh buffer, according to manufacturer’s specifications, then proteins were snap frozen in N_2_ (liq.). All SPR experiments were performed using a Biacore 8K+ instrument (Cytiva) at 20 °C, using a Biotin CAPture kit, Series S (Cytiva) or SA chip Series S (Cytiva) according to manufacturer’s specifications. The SPR running buffer consisted of 20 mM HEPES pH 7.4, 150 mM NaCl, 0.005% (v/v) Tween-P20, supplemented with 2% (v/v) DMSO. Compounds (2 µL volume, from 10 mM stocks in DMSO) were acoustically dispensed as serial concentration series into 384-well polypropylene microplates (Greiner, 781280) using a Echo 650 Series Liquid Handler (Beckman Coulter), then resuspended in SPR running buffer using a Multidrop Combi Reagent Dispenser (Thermo Fisher Scientific) and sealed plates briefly centrifuged (1 min, 500 × *g*) (final 2% (v/v) DMSO in 100 µL sample volume per well). Briefly, following chip regeneration and capture of Biotin CAPture reagent to all channels, biotinylated IAP domains (50 nM in running buffer) were captured on the active flow cell to a final surface density of approximately 500–700RU. Biocytin (50 nM in running buffer) was captured on the reference flow cell. Binding experiments for cIAP1-BIR3 were performed in single cycle kinetic (SCK) format (flow rate 70 µL/min, 60 s contact time, 600 s dissociation time), consisting of a blank cycle of running buffer, then a compound series (5-point, 5-fold serial dilution, 500–0.8 nM), followed by regeneration/recapture. Binding experiments for XIAP-BIR3 and XIAP-BIR2 (res 152–236, C202A, C213G) in general were performed in Multicycle format without regeneration (flow rate 30 µL/min, 60 s contact time, 600 s dissociation time, consisting of three buffer blanks followed by the compound series (9-point, 3-fold serial dilution, 10 µM–1.5 nM). Binding experiments for IAP binders for XIAP-BIR2 (res 152–236, C202A, C213G) were performed in the same way, but using a SA chip and capture the IAP domain to the active flow cell to a final density of approximately 1500 RU and no surface regeneration between runs. All runs included solvent correction (6 pt, running buffer with 0.5–4% (v/v) DMSO) and a wash step between injections (50% (v/v) DMSO). Biacore Insight Evaluation Software (Cytiva, version 3.0.12.15655) was used to fit doubly-referenced sensorgrams to a 1:1 binding model (kinetic fit for SCK data, steady state or kinetic fit as appropriate for multicycle data).

### Structure determination by X-ray crystallography

#### Crystallization

Crystals of XIAP-BIR3 were grown by sitting drop vapor diffusion by mixing a 1:1 ratio of XIAP-BIR3 protein (10 mg/mL) to well solution containing 0.05–0.2 M HEPES pH 8.0, and 3.0–3.4 M NaCl, then incubated at 20 °C. Crystals of cIAP1-BIR3 were grown by sitting drop vapor diffusion by first preparing a complex with low affinity fragment, **L118** (final concentration of 13 mg/mL cIAP1-BIR3, 1 mM **L118**, 2% DMSO), then mixing a 1:1 ratio of complex to well solution containing 2.8–3 M NaCl and 0.2 M Bis-Tris pH 5.5 and incubating at 20 °C. Full-sized, rod-shaped crystals of XIAP-BIR3 and cIAP1-BIR3 formed within 1 week. IAP binders of interest were soaked into XIAP-BIR3 and cIAP1-BIR3 crystals. Compound stocks (10 mM) were diluted in a 1:10 molar ratio with reservoir solution, then added to existing drops containing crystals to give a final concentration of 0.2 mM compound and 2% DMSO and incubated at 20 °C for 16 h.

#### Data collection and processing

Single crystals were mounted, cryo-protected in 20% ethylene glycol (for XIAP-BIR3), then cryo-cooled in liquid nitrogen. Diffraction data were collected at a wavelength of 0.954 Å at 100 K using the MX2 beamline at the Australian Synchrotron, part of ANSTO, and made use of the Australian Cancer Research Foundation detector^83^. Diffraction data were collected using an oscillation angle of 0.1°, yielding 3600 frames per data set. Data were integrated using XDS^84^, converted to an mtz format using POINTLESS, then scaled and merged using AIMLESS^85,86^. Ligand restraints for each compound were generated using ELBOW^87^. Statistics from data processing and refinement are summarized in Supplementary Table 1.

#### Structure refinement

Pre-existing structures of human XIAP-BIR3 (PDB: 3CLX) and cIAP1-BIR3 (PDB: 4EB9) were used as search models to solve the phase problem using molecular replacement, facilitated by PHASER MR^88^ from the CCP4 program suite^86^. Iterative cycles of manual rebuilding were completed using COOT^89^, followed by model refinement using PHENIX.REFINE^90^. Model validation was completed using MOLPROBITY^91^. The statistics of model refinement for all solved structures of ligand-bound XIAP-BIR3 and cIAP1-BIR3 are summarized in Supplementary Table 1. The atomic coordinates and structure factors of XIAP-BIR3:**A171**, XIAP-BIR3:**A250** and cIAP1-BIR3:**A273** have been deposited to the Protein Data Bank with the following accession codes: 9N1R, 9N21 and 9N23, respectively.

### Cell lines and cell culture

The human cell lines NCI-H226, NCI-H2052, NCI-H520 and HEK293T cells were obtained from American Type Culture Collection (ATCC). The human mesothelioma cell line ZL55 was provided by CellBank Australia. NCI-H226, NCI-H2052 and NCI-H520 were maintained in RPMI-1640 Medium supplemented with 10% fetal bovine serum (FBS, Sigma, F9423) and 2 mM Glutamine. ZL55 was maintained in Dulbecco’s modified Eagle’s medium/F-12 1:1 (DMEM/F-12; Thermo Fisher Scientific, 11320082) supplemented with 15% FBS and 2 mM Glutamine. HEK293T cells were cultured in Dulbecco’s modified Eagle’s medium (DMEM; Thermo Fisher Scientific, 11966025) supplemented with 8% FBS. All media were supplemented with 100 U/mL penicillin, and 100 µg/mL streptomycin (Fisher Scientific, 15070063) and cells were kept at 37 °C humidified chambers with a 5% CO_2_ incubator except for HEK293T cells, which were kept at 10% CO_2_. All cell lines used were routinely screened for mycoplasma contamination in a PCR-based assay and found negative.

### Constructs and transfection

To generate the Hibit-TEAD1 cell line, NCI-H2052 cells were transduced with a lentivirus expressing the MSCV-HiBiT-FKBP12-F36V-TEAD1-PGK-Puro-IRES-GFP construct (plasmid synthesized and sequenced at GenScript). Briefly, 5 × 10^6^ Platinum-GP cells (Cell Biolabs, Inc., RV-103; lot: 101113,8) were seeded in 10 mL DMEM medium (Lonza, BE12-604F) + 10% FCS Tet System approved (Clontech, 631101) in 10 cm cell culture dishes (BD, 353003). After overnight incubation at 37 °C and 5% CO_2_ the medium was removed, and 5 mL fresh medium were added. Two transfection mixes were prepared: (1) 440 µL Opti-MEM medium (Gibco, 51985-026) + 60 µL Lipofectamine LTX (Invitrogen, 15338-100); (2) 476 µL Opti-MEM + 12 µL Plus Reagent (Invitrogen, 15338-100) + 9 µL of the plasmid encoding the expression construct for packaging (MSCV-HiBiT-FKBP12-F36V-TEAD1-PGK-Puro-IRES-GFP; 1 mg/mL concentration) and 3 µL of VSV-Gene Plasmid (1 mg/mL). Both reaction mixes were incubated at RT for 5 min. After mixing all reagents and incubating at RT for 20 min, 1000 µL of this mix were added to the 10 cm dish containing the cells. The following day the medium was removed, and 10 mL of fresh medium (DMEM + 10% FCS Tet System approved) were added. One day later 0.3 × 10^6^ cells of the target cell line NCI-H2052 (ATCC original lot#58033333) were seeded in 2 mL medium (RPMI, Gibco, A1049101; +10% FCS, Gibco, 26140-079) in 6 well plates. These cells were incubated overnight at 37 °C 5% CO_2_. The following day the viral supernatant from Platinum-GP cells was collected using a 20 mL Syringe (Injekt Luer-Lock Solo, Braun, 4606736V) and a sterile 0.45 µm filter (Costar, 431220). After removing the medium from NCI-H2052 cells, 1 mL of the collected and filtered viral supernatant was added to the target cells. Polybrene (Santa Cruz Biotechnology, sc-134220) was added to a final concentration of 8 µg/mL. Cells were incubated again for 3 days before puromycin selection was applied. Puromycin (Sigma, P9629) was added to transduced and non-transduced cells at a concentration of 2.5 µg/mL. Selection was finished as soon as all non-transduced cells were dead. Transduced cells were cultured using medium with puromycin addition.

We then proceeded to the selection of single clones carrying the insertion by seeding the parental NCI-H2052_MSCV_HiBiT_FKBP12-F36V-TEAD1_PGK_IRES-GFP cells in 96-well plates at a density of 0.7 cells/well in 200 µL RPMI + 10% FCS per well. These cells were incubated at 37 °C 5% CO_2_. After 14 days 75 µL fresh medium was added to each well. Colonies derived from in total 24 single cell clones were transferred into 6-well plates after additional 9 days of culturing and cultured independently. Upon reaching confluency, they were used for testing the HiBiT signal and selecting the final single cell clone.

For testing, 3000 cells of each clone were seeded in 40 µL of medium per well in white 384-well microplates(OptiPlate-384, Revvity, 6007290). The 384-well plates were incubated overnight before the HiBiT signal was measured by adding 20 µL of Nano-Glo® HiBiT Lytic Detection System (Promega, N3030) prepared according to the manufacturer’s protocol, and incubated at RT for 5–10 min before reading the signal using an EnVision® 2105 multimode plate reader (PerkinElmer) or a comparable plate reader. A clone with an intermediate low signal was selected as final clone and was used for all experiments.

For cellular degradation kinetics, NCI-H2052^HiBiT-TEAD1^ cells were transiently transfected in 15 cm^2^ petri dish with the LgBiT expression vector (Promega, N2681), using Effectene (Qiagen, 301427) following the manufacturer’s protocol. Cells were allowed to express the LgBiT protein for 20 h at 37 °C and 5% CO_2_ before compound treatment.

For IAP cellular BRET measurements, the cDNAs encoding fragment including BIR2, BIR3, and RING domains of either cIAP1 (UniProt Q804E2, residues 184-618) or XIAP (UniProt P98170, residues 124–497) in frame with N-terminal NanoLuc fusion were synthesized by GenScript (Singapore). Mutation in the RING domain of cIAP1 (F616A)^34^ and XIAP (V461E)^35^ were included to abrogate E3 ligase activity and block the auto-degradation of IAPs in response to compounds tested. For TEAD cellular BRET measurements, N-terminal NanoLuc-TEAD1 fusion were encoded in pF TRE3G rtTA puro expression vector including 7-residue linker (GGSGGGS) between the tag and full-length TEAD1(UniProt P28347). The constructs were cloned into the doxycycline-inducible, puromycin selectable vector, pF TRE3G rtTA puro. For nanoBRET ternary complex assay, the HaloTag-TEAD1 fusion vector was generated via amplifying the HaloTag coding sequence from pFTRE3G N-Halo EGFP rtTAAd puro vector using the following primer sets: forward (5′-CCTTAATTAAGAGGCCCTTTCGTC-3′) and reverse (5′-CCTGTACACGCCGGAAATCTCGAGC-3′). The amplified fragment was then inserted into pF TRE3G NEGFP GS hs TEAD1 via digestion with PacI and BsrGI. Lentiviruses were generated in HEK293T cells^33^ before infection of target cells and selection/maintenance in 5 μg/mL puromycin. Lentiviral constructs were induced with 0.2 μg/mL doxycycline.

### Cellular degradation assay

Single clone of NCI-H2052 stably expressing HiBiT-tagged TEAD1 were seeded at density of 3000 cells per well of a white 384-well tissue culture (TC)-treated microplates (OptiPlate-384, Revvity, 6007689) in RPMI-1640 Medium supplemented with 10% fetal bovine serum (FBS, Sigma, F9423) and 2 mM Glutamine, then incubated overnight at 37 °C, 5% CO_2_. Test compounds were titrated at nine logarithmic dose series (from 10 to 0.0001 µM, 3-fold dilution) from a 10 mM stock solution in 100% DMSO, with 40 nL transferred per well using Echo 650 Series Liquid Handler (Beckman Coulter). After 20 h incubation, HiBiT levels were detected using the Nano-Glo® HiBiT lytic detection system (Promega, N3040) following manufacturer’s instructions. Briefly, 20 µL of Nano-Glo HiBiT Lytic Detection Reagent was added directly to the cells and incubated for 10 min on an orbital shaker (60 rpm) before recording luminescence on CLARIOstar^Plus^ plate reader (BMG Labtech) with 0.5 s integration time. Cellular ATP content was measured using CellTiter Glo 2.0 assay (Promega, G9242) as a checking assay in parallel to the HiBiT assay to assess potential cytotoxicity side effects of test compounds. For this purpose, 15 µL of CellTiter Glo 2.0 reagent (Promega, G924C) were added to each well, and the plate was incubated for 10 min before measuring luminescence signal on CLARIOstar^Plus^ plate reader (BMG Labtech). HiBiT signal was normalized to CTG reading, and the HiBiT/CTG ratio was compared to DMSO-treated well to determine % of TEAD1 remaining. The compound dose response curves were fitted using Bayesian Gaussian Processes (GP) model^92^ and the DC_50_ and the maximum effect *D*_max_ were estimated from the fitted curves.

### Cellular IAP engagement and cell permeability assays

NanoBRET experiments were performed in white 384-well plates (OptiPlate-384, Revvity, 6007689). Stable, doxycycline-inducible HEK293T cell lines expressing NanoLuc-tagged cIAP1_184–618_^F616A^ or XIAP_124–497_^V461E^ were plated at a density of 2500 cells per well in 40 μL of Opti-MEM without phenol red (Life Technologies) containing 1% FBS and 0.2 μg/mL doxycycline and incubated overnight at 37 °C, 10% CO_2_. IAP Tracer was prepared first at a stock concentration of 100X in DMSO, after which the 100X stock was diluted to a working concentration of 10X in Tracer dilution buffer (6.25 mM HEPES, 15.63% PEG-400, pH 7.5). To determine the optimal tracer concentration, we first generated the dose–response curves of the IAP tracer (cIAP1/XIAP BIR3 tracer: **B678**) by adding the tracer to the cells in a 9-point, 3-fold dilution series starting at a final concentration of 10 μM in the presence or absence of digitonin as permeabilizing agent (50 μg/ mL). As a control, the same experiment was repeated in the presence of an excess of unlabeled ligand (10 μM **A255**) as a competitive inhibitor. Plates were incubated at 37 °C, 10% CO_2_ for 1 h. A 10 μL NanoGlo Substrate (1/50 dilution in Opti-MEM, Promega, N1120) was transferred per well and BRET signals were collected on a CLARIOstar^Plus^ plate reader (BMG Labtech) equipped with a 460/80-nm bandpass (BP) filter for donor emission and a 590/60-nm BP filter for acceptor emission. BRET ratio were calculated as the ratio of the acceptor emission value to the donor emission value and expressed in milliBRET units by multiplying by 1000. Background correction was performed by subtracting no tracer mBRET ratio from these values then fitted to the hyperbolic dose–response equation for binding to a single site available in GraphPad Prism (GraphPad, v. 10.3.1). The tracer equilibrium dissociation constant (*K*_D_) values were obtained in both cell lines (230 nM (live)/24 nM (permeabilized) for cIAP1 and 250 nM (live)/7 nM (permeabilized) for XIAP). Based on the robustness of assays and signal-to-noise ratio, we selected 250 and 25 nM as optimal tracer concentrations for live and permeabilized assays, respectively.

Compounds’ binding affinities for cIAP1/XIAP were measured in live and permeabilized cells. Two hours after compound addition via Echo 650 Series Liquid Handler (Beckman Coulter), the IAP tracer was added to the cells at final concentrations of 25 nM for permeabilized-mode or 250 nM for live-mode. For the permeabilized-mode NanoBRET assay, digitonin at a final concentration of 50 μg/mL was added at the time of tracer addition. Plates were then incubated for another hour before BRET measurement. Fractional tracer occupancy (%) was calculated by dividing the background-corrected mBRET in the presence of the test compound and tracer by that of the 100% BRET control (tracer + DMSO). To determine the test compound concentration that yielded a half-maximal response (IC_50_), tracer occupancy (%) values from three biological replicates (*n* = 2) were plotted as a function of test compound concentration, and the data were fitted to the [inhibitor] vs. response (three parameters) equation available in GraphPad Prism (GraphPad, v. 10.3.1). The relative intracellular availability (RBA) of the test compounds is estimated using the equation,

$${{\mathrm{RBA}}}=\,\frac{{IC}_{50}^{\,\,\,\,\,\,\,{live}-{mode}}}{{IC}_{50}^{\,\,\,\,\,\,\,{permeabilised}-{mode}}}$$

where IC_50_ under permeabilized cell conditions is a proxy for intrinsic affinity of the interaction. The RBA for the permeable control compound (**A255**)^47^ is used to calibrate the assay behavior, and the RBA values for all compounds are then normalized to that of the permeable control compound to establish an AI, where AI values greater than 1 suggest reduced availability compared to the control, and vice versa.

### NanoBRET in-cell TEAD1 engagement assay

To evaluate In-cell TEAD engagement, experiments were conducted as described above for the nanoBRET IAP engagement assay, except that stable, doxycycline-inducible HEK293T cell lines that express NanoLuc-tagged TEAD1 were used. The assay was conducted in live cells. For the TEAD tracer dose–response experiment, 10 μM **A262** was used as the unlabeled ligand. To assess the binding affinities of test compounds, the TEAD tracer was used at a final concentration of 20 nM, as determined from the tracer dose–response curve and incubated for 2 h before BRET measurements. The acceptor emission was collected with 610/60-nm long pass (LP) filter.

### Cellular ternary complex assay

Stable, doxycycline-inducible NCI-H2052 cell lines co-expressing NanoLuc-tagged cIAP1_184–618_^F616A^/Halo-TEAD1 or NanoLuc-tagged XIAP_124–497_^V461E^/Halo-TEAD1were seeded into white 384-well microplates (Revvity, 6007689) in Opti-MEM, No Phenol Red containing 4% FBS and 0.2 μg/mL doxycycline with or without HaloTag® NanoBRET® 618 Ligand (Promega, N1662) and incubated at 37 °C, 5% CO_2_ for assay the following day_._ A 9-points, 1:3 compound dose–response titration starting from 30 μM was directly added to appropriate wells using Echo 650 Series Liquid Handler (Beckman Coulter), and then assay plates were incubated at 37 °C, 5% CO_2_. After 4 h of compound treatment, plates were equilibrated to RT for 15 min, followed by the addition of 25 μL NanoGlo Substrate (1/168 dilution in Opti-MEM, Promega, N2584). The contents were mixed by shaking the plate for 30 s before measuring donor and acceptor signals on CLARIOstar^Plus^ plate reader (BMG Labtech). Dual-filtered luminescence was collected with a 450/50 nm BP filter (donor, NanoLuc-cIAP1 or NanoLuc-XIAP) and a 610-nm LP filter (acceptor, HaloTag NanoBRET ligand) using 1.0-s integration time. The raw data is checked for outliers, and outliers are removed based on visual examination. Background corrected (cells without HaloTag 618 ligand) nanoBRET ratios were calculated and plotted against compound concentration on a log_10_ scale using GraphPad Prism (GraphPad, v. 10.3.1). Data were fitted using Gaussian distribution model to calculate EC_max_(IPD concentration yielding maximum signal) and *E*_max_ (maximum signal) values.

### Cell proliferation assay

NCI-H226, NCI-H2052, ZL55 and NCI-H520 cells were counted, and the viability was assessed with the use of ViCell XR cell counter (Beckman Coulter Life Sciences). For compound testing, 200 cells in 50 µL per well were seeded in white opaque 384-well microplates (OptiPlate-384, Revvity, 6007290), and the plates were incubated at 37 °C, 5% CO_2_ overnight to ensure that cells are adherent prior to treatment. A t0 plate was prepared as well following the same protocol. Twenty four hours later, the compounds were added from DMSO stock solution to the cells using D300 Digital Dispenser (Tecan Life Sciences) (max. final DMSO concentration was set to 0.1%). Cells were treated with the compounds in duplicates, and a dilution series of 3.16-fold was introduced, within a concentration range of 1 nM–10 µM. As negative control, 0.1% DMSO was added to the cells. At the timepoint of treatment, the t0 plate was measured for qualitative evaluation of cell proliferation, while the effect of compounds on proliferation was assessed 144 h after treatment. For this purpose, 20 µL of CellTiter Glo 2.0 reagent (Promega, G924C) were added to each well, and the plate was incubated for 10 min on a plate shaker (Titramax 101, Heidolph) at RT to stabilize the luminescence signal. Subsequently, the luminescence signal was measured using an EnSpire plate reader (PerkinElmer).

### qPCR analysis

For the qPCR assays, we seeded 20.000 cells in 100 µL per well in 96-well plates (Corning, CLS 3585), and the plates were incubated at 37 °C, 5% CO_2_ overnight to ensure cell adhesion. 24 h later, cells were treated with the compounds in duplicates. We used a total of 5 concentration points with 1:10 dilution factor up to 10 µM, and compounds were added to cells from their DMSO stock using a D300 Digital Dispenser. While DMSO was normalized in all wells to highest volume in the plate, 0.3% DMSO was added to cells as negative control. Subsequently, plates were transferred to incubator and kept at 37 °C, 5% CO_2_. The cells were lysed 48 h after treatment using the FastLane Cell RT-PCR QuantiTect Multiplex RT-PCR Kit (Qiagen, 216513). We slightly modified the protocol compared to manufacturer’s recommendations: the media was removed from the wells and cells were washed with 80 µL FCW Buffer once for couple of seconds. After the removal of FCW Buffer, 30 µL cell processing mix (composed of 28.2 µL FCPL Buffer and 1.8 µL gDNA Wipeout Buffer from the kit) was added to cells and incubated for 5 min at RT. Subsequently, the cells were transferred into a storage plate (Eppendorf, 0030128575), which was sealed and heated up to 75 °C for 5 min in a cycling machine (BioRad, T100 Thermal cycler). Then the ready-to-use lysates were stored at −80 °C until use in qPCR assays. The reaction mix for qPCR assays was prepared in 96-well MicroAmp Fast Optical Reaction Plates (ThermoFisher Scientific, 4346906) as follows: for each sample we mixed 10 µL QuantiTect Multiplex RT-PCR master mix, 0.33 µL from each of two primers (for housekeeping and target gene), 0.2 µL QuantiTect Multiplex RT mix and 3 µL of cell lysate, and added water up to 20 µL. Taqman probes detecting *B2M* (beta-2 microglobulin) (Thermo Fisher Scientific, Hs00187842_m1) as reference gene, and *CTGF* (Thermo Fisher Scientific, Hs00170014_m1) as target downstream gene were used. The PCR reaction included a preliminary reverse transcription step at 50 °C for 20 min, followed by denaturation at 95 °C for 15 min, and 40 cycles at 94 °C for 45 s and 60 °C for another 45 s. For the analysis of qPCR results, we used the classic delta-delta Ct method, using the formula: 2^−ΔΔCt^. In summary, first, the difference in Ct levels of the target gene *CTGF* and the housekeeping gene *B2M* was determined (ΔCt). Then, ΔCt values of treated samples were normalized to the ΔCt of the control (i.e., only DMSO-treatment) sample (ΔΔCt). After that, the relative fold change in gene expression level was calculated (2^−ΔΔCt^). Calculations and graphs were made using an in-house developed software (MegaLab).

### TEAD1 stability cycloheximide experiment

In order to generate lysates for WES analysis, 20.000 cells per well were seeded in 100 µL culture medium in 96-well plates (Corning, CLS 3585), and the plates were incubated at 37 °C, 5% CO_2_, overnight for cell attachment. The following day, cells were treated in duplicates at different time points with 100 µg/mL Cycloheximide (CHX) (Sigma, C4859) or the same amount of DMSO for comparison. After 8, 4, 2, 1 or 0 h of treatment time, cell lysates were collected. To this aim, the supernatant was discarded, cells were washed with 100 µL PBS per well, before 35 µL of RIPA Buffer (Sigma, R0278) + HALT protease/phosphatase inhibitor cocktail (Thermo Fisher Scientific, 78446) per well was added to each well of the assay plate. Plates with lysate samples were incubated at 4 °C for 15 min and then transferred to −80 °C. After samples were frozen completely, they were thawed again at 4 °C. Samples were transferred to a 96well V-Bottom plate (Corning, 3894) for centrifugation at 4 °C for 20 min at full speed. After this centrifugation step, the supernatant was transferred to a storage plate (Eppendorf, 0030128575), which was sealed using an adherent foil and stored at −80 °C until further usage of the samples.

For analysis of TEAD1 protein level, the frozen samples were thawed at 4 °C and analyzed using a WES Kit with 12–230 kDa Separation Module (Bio-Techne, SM-W001) together with Anti-Rabbit Detection Module (Bio-Techne, DM-001), anti TEAD1 antibody at a dilution of 1:50 (Cell Signaling Technology, 12292S) and anti GAPDH antibody at a dilution at 1:500 (Abcam, ab9485). 4.8 µL of lysate samples and 1.2 µL of 5x FL Standard Master Mix (part of SM-W001) were combined before incubating at 95 °C for 5 min. Four microliters of the prepared samples as well as other reagents were added to the WES plate according to the manufacturer’s protocol and the run was started using the default settings. Data analysis was performed using the Compass for Simple Western Software (ProteinSimple; Version 6.1.0) and Microsoft Excel (Microsoft, v 16.9.2). The mean of chemiluminescence signals for TEAD1 protein was normalized using the signal of GAPDH within the same capillary and was then compared to the DMSO-treated (no CHX) samples of the respective Cycloheximide treatment time.

### CRISPR KO generation

sgRNAs targeting human genes were available cloned in lentiviral vector as bacterial glycerol stocks at WEHI from Sanger Arrayed Whole Genome Lentiviral CRISPR Library (Sigma-Aldrich/Merck, HSANGERG). sgRNA sequences are as follows: TEAD1 (s1: 5′-ACATTCAGGTTCTTGCCAGAAGG-3′, s2: 5′-TGGCCGGGAATGATTCAAACAGG-3′), TEAD4 (s1: 5′-CCTTTCTCTCAGCAAACCTATGC-3′, s2: 5′-CCAGCCTCCGCTGCCTCTGCCAG-3′), cIAP1(s1: 5′-CCTGGAGAAAGTTCTTCAGAAGA-3’, s2: 5’-ACCTGTGGTTAAATCTGCCTTGG-3’) & XIAP (s1: 5’-CAAATATCTGTTAGAACAGAAGG-3′, s2: 5′-GCAGCTAAGGCGCCTGCAAGAGG-3′). Plasmids were purified for two optimized gRNA clones per gene and correct sequences confirmed (using primer 5′-GGCACTGCGTGCGCCAATTC-3′). The gRNA vector (1 µg) along with packaging pMD2.G VSVg (0.4 µg) and envelope vector pCMV-dR8.2 (1 µg) were used to generate lentiviruses in HEK293T cells using Effectene transfection kit (Qiagen, 301425). Lentiviral supernatants for gRNA were filtered with 0.45 µM filter and stored at -80C with polybrene (5 µg/mL) until use.

Cas9 mCherry lentivirus were also generated using similar transfection method as above using pFUCas9Cherry plasmid (gift of Marco Herold lab, WEHI).

NCI-H226 and NCI-H2052 cells were first transduced with Cas9 mCherry lentivirus and sorted to (BD FACSDiva 8.0.1) 90% purity using the mCherry marker. H226 and H2052 Cas9 mCherry stable lines were further transduced with respective gRNA lentivirus (TEAD1, cIAP1 or XIAP), followed by selection with puromycin. Generation of polyclonal KO was tested by Western blot. For the generation of single cells KO clones of *cIAP1* and *XIAP*, single cells were sorted from polyclonal KO lines into 96-well plates, and after 4 weeks of expansion, about 30 clones were screened for KO status. Cells that were either *cIAP1* or *XIAP* KO were then transfected with gRNA XIAP and cIAP1 lentivirus, respectively. After selection with puromycin, cells were confirmed of their double KO status by western blot.

### Cell lysis and immunoblotting

For western blot, cells were lysed on ice for 45 min in complete RIPA lysis buffer containing 1% Triton X-100, 150 mM NaCl, 1% Sodium deoxycholate, 0.1% SDS, 50 mM Tris (pH = 7.5) supplemented with Benzonase (Millipore, US170746-3) and 1X phosphatase (PhosSTOP, Roche, 4906837001) and protease inhibitors (complete EDTA free, Roche, 11873580001). Following centrifugation at 13,000 rpm for 10 min at 4 °C, supernatants were used as lysate. Lysates were loaded on NuPAGE Novex 4–12% Bis Tris gels (Invitrogen, NP0335BOX) and transferred to PVDF membrane (iBlot 2 transfer stacks, Invitrogen, IB24002) using iBlot 2 gel transfer device (Invitrogen). Membranes were blocked in 3% BSA (Sigma, A3294) and probed with primary antibodies overnight at 4 °C. Primary antibodies used were: rat anti-cIAP1 (1:500, Enzo, ALX-803-335), mouse anti-XIAP(1:1000, MBL life science, M044-3), rabbit anti-TEAD1 (1:1000, Cell Signaling Technology, 12292S), rabbit anti-Pan-TEAD (1:1000, Cell Signaling Technology, 13295S), mouse anti-TEAD4 (1:1000, Abcam, ab58310), mouse anti-GAPDH (1:10,000 Sigma, G8795), mouse anti-Hi-Bit (1:1000, Promega, N7200), rat anti-Hsp90 (1:1000, Enzo life science, DI-SPA835), rabbit anti-Lamin B1 (1:1000, Cell Signaling Technology, 12586) and rabbit anti-NFkb2 p100/p52 (1:1000, Cell Signaling Technology, 4882). Following 3 washes (10 min each) with PBS-0.1% Tween20 (PBS-T), membranes were incubated with secondary antibodies for 45’ at RT followed by 3 washes with PBS-T. Secondary antibodies used were: goat anti-Rat IgG HRP (1:5000, Southern Biotech, 3010-05), goat Anti-Rabbit IgG HRP (1:5000, Southern Biotech, 4010-05), goat anti-Mouse HRP (1:5000, Southern Biotech, 1010-05). The membranes were developed using Immobilon Forte Western HRP substrate (Merck Millipore, WBLUF0500) and imaged on Chemidoc MP Imaging system (Bio-Rad, 2.4.0.03).

### Cytoplasmic and nuclear fractionation

Cytoplasmic and nuclear fractions were purified from HEK293T, NCI-H2052, NCI-H2052 HiBiT-TEAD1 and NCI-H2052 HiBiT-TEAD4 cells using nuclear extraction kit (Abcam, ab113474) according to the manufacturer’s instructions, with some modifications. Briefly, 1 million cells were resuspended in 100 µL of 1X pre-extraction buffer and incubated on ice for 10 min. The suspension was then vortexed vigorously for 10 s and spun down at 12000 rpm for 1 min. The ensuing supernatant was used as “cytosolic fraction”. The “nuclear” pellet was further washed with 1X pre-extraction buffer by centrifuging at 12000 rpm for 30 s, followed by solubilization in 25 µL of 1X Laemmli buffer at 95 °C for 5 min. For Western blot analysis, equivalent cytosolic fractions and whole cell lysates were also solubilized in 1X Laemmli buffer at 95 °C for 5 min and analyzed along with nuclear fractions for cytosolic /nuclear markers and proteins of interest.

### Endogenous protein degradation assay

Levels of endogenous TEAD1, TEAD4, cIAP1, XIAP and GAPDH for dose response experiments were assessed by capillary electrophoresis method (JESS Simple Western System, ProteinSimple/Bio-Techne). 5000 cells were seeded in round bottom 96 well plates (Falcon, 353077) 24 h prior to compound treatment. Cells were treated for specified time points with compounds stocks in DMSO sequentially diluted 3-fold from 10 µM to 0.001 µM and DMSO. Post treatment, the media was discarded, and cells were washed with cold PBS two times. Pelleted cells in plate were lysed in 35 µL of complete RIPA lysis buffer (described in cell lysis protocol) on ice for 45 min. The plate was then centrifuged at 3900 rpm for 20 min at 4 °C to clarify the whole cell lysate. The supernatant was then used to quantify endogenous protein levels. Sample preparation method provided by ProteinSimple was used. Briefly, 1–2 µg equivalent protein was diluted in manufacturer provided fluorescent 5X master mix. Samples were diluted with 0.1X sample buffer as needed. Samples were then denatured at 95 °C for 5 min. Denatured samples were then run on in 12–230 kDa capillaries (Bio-Techne, SM-FL004) to detect endogenous target proteins.

Protein levels were calculated using Compass for Simple Western software (ProteinSimple; Version 6.1.0) as the area under the curve of each target protein normalized to the area under the curve of loading control GAPDH in each capillary. Ensuing values were then normalized to DMSO values and represented as % protein remaining values. These values were plotted in GraphPad Prism (Version 10.2.0) and the dose response curves were fitted using the one-phase decay model. *D*_max_ (%) and DC_50_ (nM) values were calculated using the parameters in the following calculations as 100-Plateau and 1000*Half-life, respectively.

### HiBiT-TEAD1 live cell kinetic degradation

NCI-H2052^HiBiT-TEAD1^ cells transiently expressing LgBiT protein were plated into a white 384-well plate (Revvity, 6007689) at density of 5000 cells per well in FluoroBrite™ DMEM supplemented with 10% FCS and incubated overnight at 37 °C, 5% CO_2_. The Nano-Glo® Vivazine substrate (Promega, N2581) was added and incubated for 1 h prior readout for substrate activation. After incubation, compounds were added, and the plate was sealed with BreathEasy plate seal (Sigma Aldrich, Z380059) and continuously measured every 30 min for 20 h on CLARIOstar^Plus^ plate reader (BMG Labtech). Untreated baseline measurements were subtracted from the measurements to normalize the data and plotted using GraphPad Prism.

### HiBiT-TEAD1–4 degradation assay with normalization to CTG

Cell lines for the HiBiT assay were generated by inserting in the NCI-H226 cell line a transgene expressing the HiBiT-FKBP12-F36V-TEAD1–4 insert under control of a MSCV promoter using standard lentiviral transduction methods. The cells were cultured using RPMI 1640 (PAN-Biotech, P04-18047) plus NEAA (Gibco, 11140-035), 1 mM Sodium pyruvate (Gibco, 11360-039), 4.5 g/L Glucose (GlutaMAX, Gibco, 35050-038), 10 mM HEPES and 1.5 g/L NaHCO3 and FCS (Gibco, 26140-079), plus Puromycin at 2 µg/ml concentration (Sigma, P9620) and grown at 37 °C, 5% CO_2_.

#### HiBiT assay

5000 cells per well were seeded in white 384-well microplates in two sets (OptiPlate-384, Revvity, 6007290) in 40 µL assay medium (growth medium: RPMI1640 with supplements; no puromycin). Next day, a 1:20 predilution of each compound was prepared (stock concentration 10 mM) diluting it in DMSO, and compounds aliquots were added to the cells using the digital dispenser D300e (D300e Digital Dispenser, Tecan) using a concentration range from 10 µM to 0.3 nM with dilution steps of 1:3. Negative control samples were treated with DMSO only and all samples were normalized to the same DMSO volume as well (0.25% maximum DMSO). Duplicates were measured for each condition and the edge wells of each plate were not used for treatment. After compounds addition, the cells were incubated again at 37 °C 5% CO_2_ for 18 h, following which one plate was measured using the NanoGlo HiBiT Lytic Detection System (Promega, N3040) and another using the CellTiter-Glo 2.0 Luminescent Cell Viability Assay (Promega, G9243) according to manufacture instructions.

Briefly, LgBit Protein (1:100) and the Nano-Glo HiBiT Lytic Substrate (1:50) were diluted in HiBiT Lytic Buffer. 20 µL of the prepared NanoGlo mix was added one plate, and 20 µL of CellTiter Glo 2.0 reagent added to another. Both plates were incubated for 10 min on a plate shaker at RT protected from light (IKA MTS 4 MTP Microplate Shaker). Subsequently, the luminescence signal of both plates was measured using a plate reader (EnVision® 2105 multimode plate reader, PerkinElmer). The luminescence signal was measured in accordance with the settings defined in the Ultrasensitive luminescence protocol for 384 plate formats (distance determination: 0.1, measurement time [s]: 0.1, glow (CT2) correction factor [%]: 0). Prior to normalizing the data, the raw data was checked for outliers that were removed from the fitted curve calculation.

For each condition, raw data from the plate measured using the NanoGlo HiBiT Lytic system were normalized against the data from the other plate, which was measured using the CellTiter Glo 2.0 reagent, to account for HiBiT signal variations due to changes in cell number. The average of the negative control samples (DMSO treatment only) was calculated and used to normalize all compound-treated samples. The normalized raw data were fitted to a four-parameter logistic regression model (variable slope) by a customized software in accordance to the formula: Y = Bottom + (Top-Bottom)/(1 + 10^((LogIC50-X)* Slope)).

### Proteomics and mass spectrometry

#### Proteomics sample preparation

NCI-H2052 cells (0.25 × 10^6^ cells) were seeded in a six-well plate. Post 24 h seeding, cells were treated with 0.5 µM of XB2 **A538**, IAP negative control **A559**, TEAD negative control **A561** and DMSO control for 16 h (*n* = 5 biological replicates each). Cells were washed thrice with cold PBS, and pellets were snap frozen.

When ready for extraction, frozen samples were immediately lysed in 50 μL of preheated (95 °C) lysis buffer (2.5% SDS in 100 mM Tris-HCl, pH 8.5). DNA was hydrolyzed with the addition of 5 μL of 10% TFA (Thermo Fisher Scientific) and lysates were neutralized by the addition of 16 μl 1 M Tris-HCl pH 8.5. The protein concentrations of the samples were determined using Pierce^TM^ BCA Protein Assay Kit following manufacturers’ instructions. Protein lysates (20 μg protein per replicate) were transferred to 0.5 mL LoBind Deep Well plate (Eppendorf) and prepared for mass spectrometry analysis using the USP3 protocol^93^. Briefly, samples were subjected to simultaneous reduction and alkylation with a final concentration of 10 mM Tris (2-carboxyethyl) phosphine (TCEP) and 40 mM 2-chloracetamide followed by heating at 95 °C for 10 min. Prewashed magnetic PureCube Carboxy agarose beads (20 μl, Cube Biotech) were added to all the samples along with acetonitrile (ACN, 70% v/v final concentration) and incubated at room temperature for 20 min. Samples were placed on a magnetic rack and supernatants were discarded, and beads were washed twice with 70% ethanol and once with neat ACN. ACN was completely evaporated from the tubes using a CentriVap (Labconco) before the addition of digestion buffer (50 mM Tris-HCl, pH 8) containing 0.4 μg each of Lys-C (Wako, 129–02541) and SOLu-Trypsin enzymes (Sigma-Aldrich, EMS0004). Trypsin-LysC on-bead digestion was performed with agitation (400 rpm) for 1 h at 37 °C on a ThermoMixer C (Eppendorf). Following digestion, the samples were transferred to pre-equilibrated C18 StageTips (AttractSPE Tips C18, product number: C18.T3.200.96 AFF, Affinisep) for sample clean-up. The eluates were lyophilized before being reconstituted in 100 μL 0.1% FA/2% ACN, ready for mass spectrometry analysis.

#### Mass spectrometry

Reconstituted peptides were analysed on an Orbitrap Astral™ (Thermo Scientific) that is interfaced with a Neo Vanquish liquid chromatography system (Thermo Scientific). A volume of 0.2 µL was loaded onto a C18 fused silica column (inner diameter 75 µm, OD 360 µm × 15 cm length, 1.6 µm C18 beads) packed into an emitter tip (IonOpticks) using pressure-controlled loading with a maximum pressure of 1500 bar. The HPLC was interfaced to the Orbitrap Astral™ using an Easy nLC source and electrosprayed directly into the mass spectrometer. Peptides were loaded onto the column with the following analytical gradient: buffer A (0.1% FA) and 4% buffer B (80% ACN, 0.1% FA) followed by an increase of buffer B to 34% for 20 min, and 100% for 3 min at a flow rate of 400 nL/min.

A data-independent acquisition MS method was used in which one full scan (380–980 m/z, *R*  =  240,000) at a target of 5 × 10^6^ ions was first performed, followed by 200 windows with a resolution of 80,000 (at m/z 524) where precursor ions were fragmented with higher-energy collisional dissociation (collision energy 25%) and analysed with an AGC target of 8 × 10^4^ ions and a maximum injection time of 3 ms in profile mode using positive polarity.

#### Data analysis

Thermo raw files were processed by directDIA™ library-free search using Spectronaut software version 20.0^94^. Files were searched against reviewed sequences from the UniProt Human proteome database (downloaded August 2024) with the following default settings: trypsin specificity, peptide length of 7–52 residues, cysteine carbamidomethylation as a fixed modification, variable modifications set to n-terminal protein acetylation and oxidation of methionine, the maximum number of missed cleavages at 2, and filtering outputs set at a PSM, peptide, and protein FDR cutoff of < 1%.

#### Statistical analysis

Data processing and analysis of the Spectronaut output were performed using R software (v. 4.4.2). Only proteins identified using proteotypic peptides were retained for downstream analysis. To ensure data quality, protein groups that were present in at least 50% of replicates within at least one experimental condition were included for further analysis, resulting in a final set of 8937 proteins. Protein intensity values were log2-transformed to meet the assumptions of downstream statistical tests. Normalization was performed using the cyclic loess method implemented in the limma R package (v. 3.62.2).

Principal Component Analysis (PCA) was conducted to reduce the dimensionality of the data and identify potential outliers. Differential expression analysis was carried out using the limma, with empirical Bayes moderation to enhance statistical power. Proteins were considered significantly differentially expressed if they met a false discovery rate (FDR) threshold of ≤ 5% following Benjamini–Hochberg (BH) correction and had an absolute log2 fold change ≥ 1.25. Data visualization was performed using the ggplot2 R package. The mass spectrometry proteomics data have been deposited to the ProteomeXchange Consortium via the PRIDE partner repository^95^ with the dataset identifier PXD068528.

TEAD target genes (Supplementary Data 3, 4 and 5) were annotated with information from the public resource *Harmonizome* (version 3.0; https://maayanlab.cloud/Harmonizome/). We used the JASPAR^96^ predicted transcription factor TEAD1 gene target dataset (https://maayanlab.cloud/Harmonizome/dataset/JASPAR+Predicted+Transcription+Factor+Targets), that lists 1541 target genes of TEAD1, based on curated and non-redundant TF binding profiles.

### Statistical analysis

Statistical analysis and error estimates are detailed in the figure and table legends and methods.

### Reporting summary

Further information on research design is available in the Nature Portfolio Reporting Summary linked to this article.

## Supplementary information

Supplementary Information

Description of Additional Supplementary Files

Supplementary Data 1

Supplementary Data 2

Supplementary Data 3

Supplementary Data 4

Supplementary Data 5

Life Sciences Reporting Summary

## Data Availability

Additional data supporting this study are available within the Supplementary Information and Supplementary Data 1–5. Coordinates and structure factors for the X-ray crystal structures have been deposited in the PDB with accession codes 9N1R (XIAP-BIR3:**A171**), 9N21 (XIAP-BIR3:**A250**) and 9N23 (cIAP1-BIR3:**A273**). The mass spectrometry proteomics data have been deposited to the ProteomeXchange Consortium via the PRIDE partner repository with the dataset identifier PXD068528.
