# Supplementary material for: Expanding the toolbox to develop IAP-based degraders of TEAD transcription factors
Source: Commun Chem. 2026 Jan 19;9:69. doi: 10.1038/s42004-025-01871-x (PMC12873239; doi:10.1038/s42004-025-01871-x)
Supplement: Supplementary file 1 — Supplementary Information [file 42004_2025_1871_MOESM1_ESM.pdf]

Supplementary Information:

Expanding the toolbox to develop IAP-based degraders of TEAD transcription factors

Nishma Gupta[1,4‡], Nicole Trainor[2,4‡], Mona Radwan[1,4,5], Stephanie Nguyen[2,4], Luke Duncan[2,4], Andrew X. Tang[2,4], Julia Beveridge[2,4,8], Natasha Silke[1,4], Jumana Yousef[3,4], Ceren Bilgilier[6], Johannes Wachter[6], Peter Greb[6], Zuzana Jandova[6], Ján Eliaš[6], Sara Kopf[6], Thomas Gerstberger[6], Peggy Stolt-Bergner[7], Nina Braun[6], Harald Weinstabl[6], Darryl B. McConnell[6,9], Federico Mauri[6#], Isabelle S. Lucet[2,4#], John Silke[1,4#], Nicola E. A. Chessum[6#*], Michael J. Roy[2,4,10#*]

[1]Inflammation Division, Walter and Eliza Hall Institute, Parkville, VIC, Australia. [2]ACRF Chemical Biology Division, Walter and Eliza Hall Institute, Parkville, VIC, Australia. [3]Advanced Technology and Biology Division, Walter and Eliza Hall Institute, Parkville, VIC, Australia. [4]Department of Medical Biology, University of Melbourne, Parkville, VIC, Australia. [5]Present address: Centre for Genetic Medicine, King Faisal Specialist Hospital & Research Centre, Riyadh, KSA. [6]Boehringer Ingelheim RCV GmbH & Co KG, Vienna, Austria. [7]Boehringer Ingelheim Pharma GmbH & Co KG, Biberach, Germany. [8]Present address: BioCurate Pty Ltd, Carlton, VIC, Australia. [9]Present address: Curie.Bio, Boston, MA, USA. [10]Present address: South Australian immunoGENomics Cancer Institute (SAiGENCI), Adelaide University, Adelaide, SA, Australia. [‡]These authors contributed equally: Nishma Gupta, Nicole Trainor. [#]These authors jointly supervised this work: Federico Mauri, Isabelle S. Lucet, John Silke, Nicola E. A. Chessum, Michael J. Roy.

*Correspondence: nicola.chessum@boehringer-ingelheim.com; michael.roy@adelaide.edu.au.

Table of Contents

Supplementary Figures 3
  Supplementary Figure 1 4
  Supplementary Figure 2 9
  Supplementary Figure 3 10
  Supplementary Figure 4 13
  Supplementary Figure 5 14
  Supplementary Figure 6 18
Supplementary Tables 20
  Supplementary Table 1: Crystallographic data collection and refinement statistics. 20
Synthetic Chemistry 21
  General Methods 21
    Analytical MS Methods and Instrumentation 21

Preparative Purification Methods and Instrumentation: 21
General Synthetic Procedures 1-2 22
General procedure 1: Boc deprotection with TFA 22
General procedure 2: Amide coupling 22
Synthesis of IAP Binders 23
Synthesis of **A250** 23
Synthesis of **A171** 24
Synthesis of **A238** 27
Synthesis of **A273** 29
Synthesis of **L118** (low affinity IAP ligand for cIAP1 crystallization) 32
Synthesis of TEAD Binders 34
Synthesis of **A262** 34
Synthesis of **A341** 37
Synthesis of TEAD IPDs and negative controls 39
Synthesis of **A531** 39
Synthesis of **A557** (**A531** IAP -ve control) 42
Synthesis of **A423** (**A531** TEAD -ve control) 44
Synthesis of **A538** 48
Synthesis of **A559** (**A538** IAP -ve control) 52
Synthesis of **A561** (**A538** TEAD -ve control) 55
Synthesis of **A536** 57
Synthesis of **A558** (**A536** IAP -ve control) 60
Synthesis of **A560** (**A536** TEAD -ve control) 61
Synthesis of **A232** 64
Synthesis of **A230** (**A232** IAP negative control) 68
Synthesis of **A231** (**A232** TEAD negative control) 70
Synthesis of Tracers 73
Synthesis of **A191** (Biotinylated XB2 TR-FRET Tracer) 73
Synthesis of **B678** (IAP nanoBRET Tracer) 75
Synthesis of **A472** (TEAD nanoBRET Tracer) 78
Spectra and Analytical Data 81
Abbreviations 140
Supplementary References 142

Supplementary Figures

Supplementary Figure 1

a

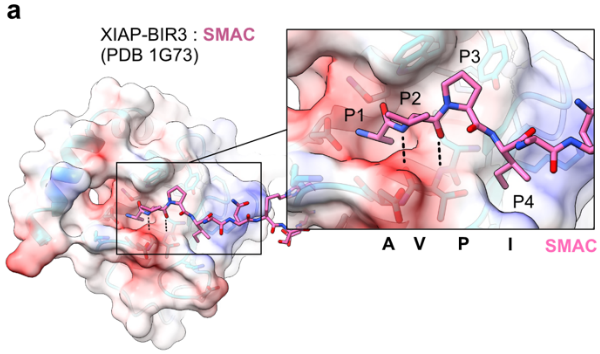

b

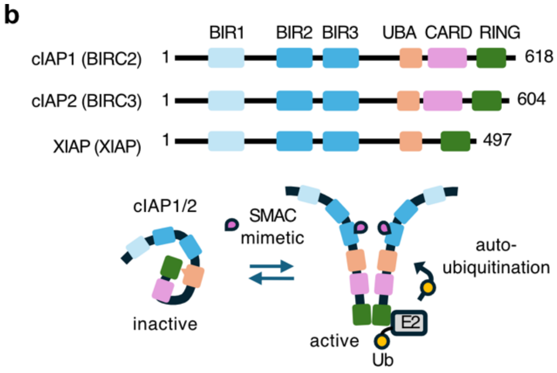

c

IAP domain binding (TR-FRET)

IAP binder (series)	cIAP1-BIR3	XIAP-BIR2	XIAP-BIR3
**A250** (ALP1)	6.6	5.5	6.1
**A171** (ALP2)	8.1	5.3	5.9
**A238** (XB2)	6.8	6.9	< 5.0
**A273** (XB2)	7.0	6.6	5.3
**A255** (ASX)	8.5	< 5.0	7.5

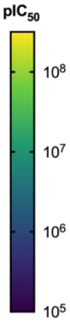

d

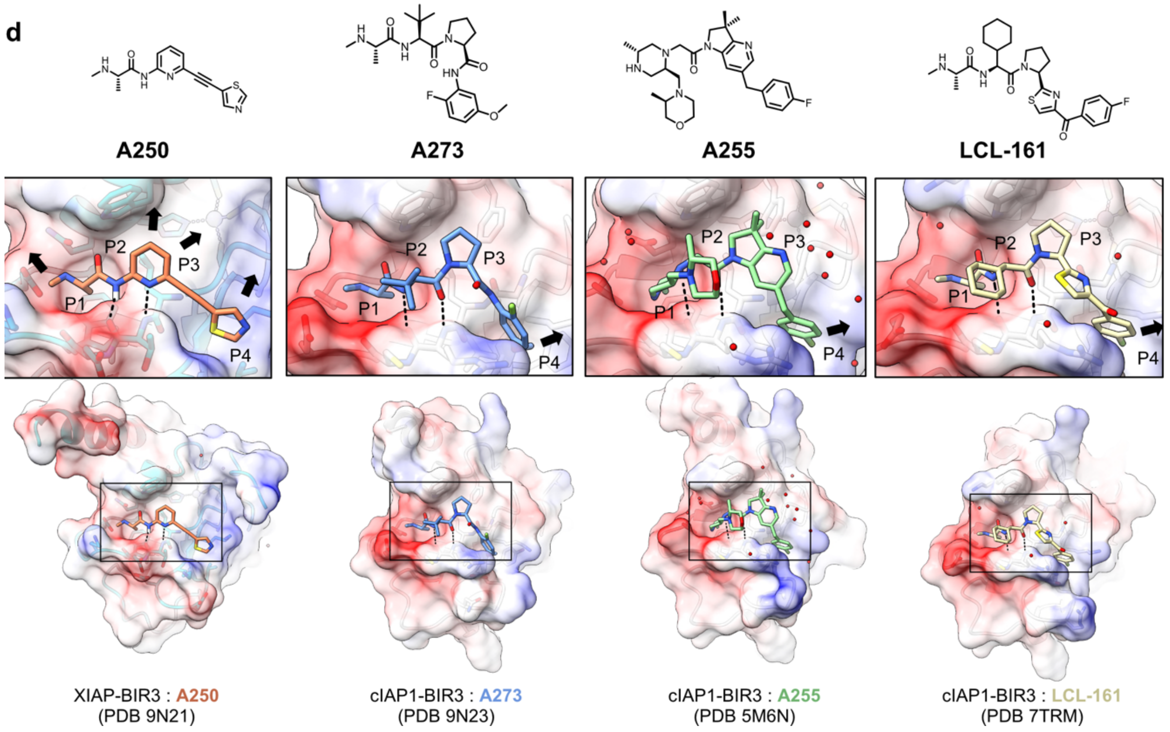

Supplementary Figure 1 (continued).

e IAP Binding – SPR – Fitted Sensorgrams and Binding Data

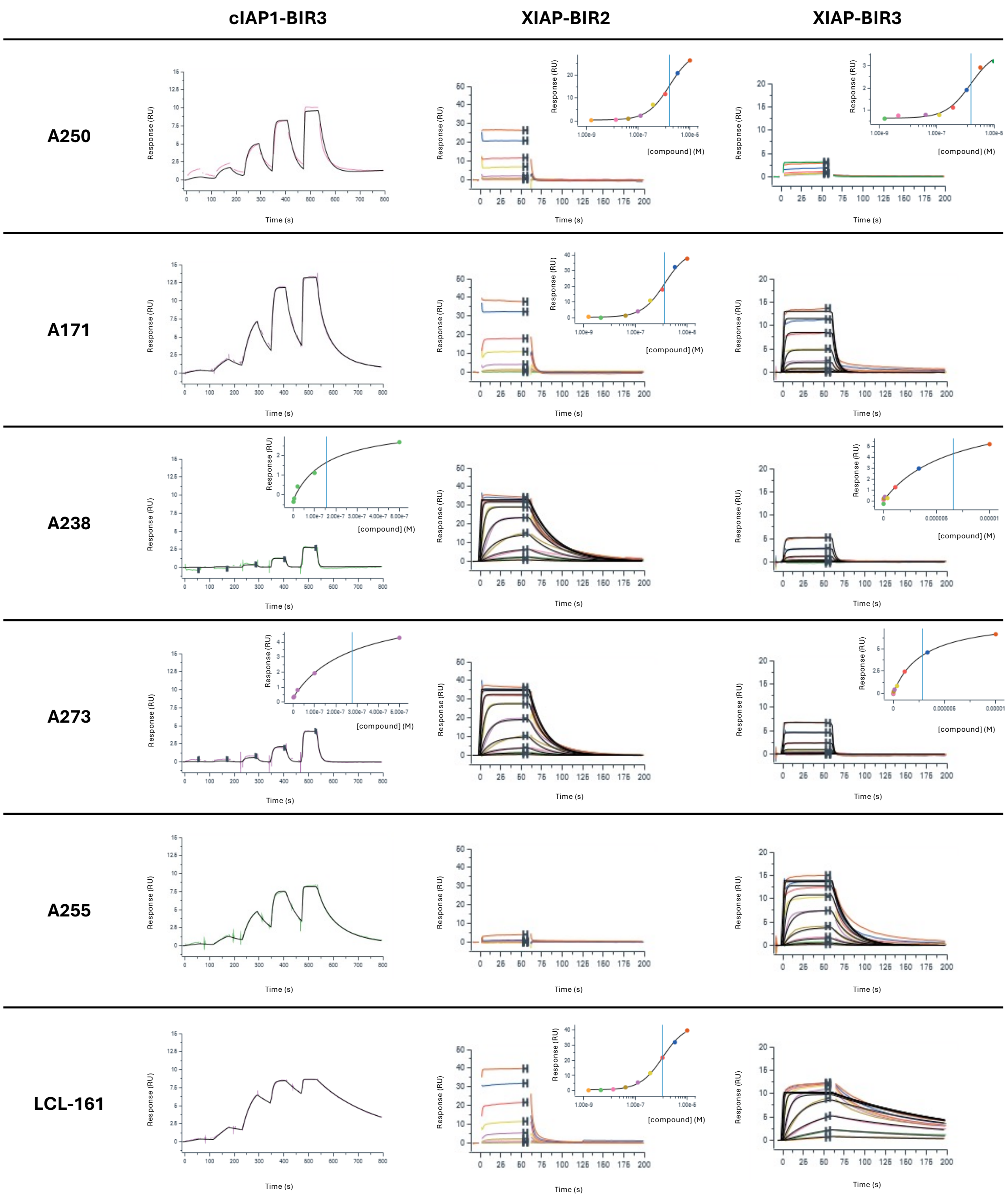

Compound	cIAP1-BIR3				XIAP-BIR2				XIAP-BIR3			
	SPR K_D (nM)				SPR K_D (nM)				SPR K_D (nM)			
	Mean	± SD	n	pIC_{50}	Mean	± SD	n	pIC_{50}	Mean	± SD	n	pIC_{50}
**A250**	19	-	1	7.7	1501	± 158	3	5.8	1427	± 161	2	5.8
**A171**	17	± 2	3	7.8	1260	± 89	3	5.9	726	± 35	3	6.1
**A238**	372	± 226	3	6.4	47	± 6	3	7.3	7232	± 1427	3	5.1
**A273**	209	± 59	3	6.7	101	± 9	3	7.0	2902	± 152	3	5.5
**A255**	10.4	± 0.7	3	8.0	> 10,000	-	3	< 5.0	99	± 11	3	7.0
**LCL-161**	2.8	± 0.1	3	8.6	1079	± 80	3	6.0	5.5	± 1.1	3	8.3

Supplementary Figure 1 (continued).

f IAP Binding – TR-FRET – Fitted Binding Data

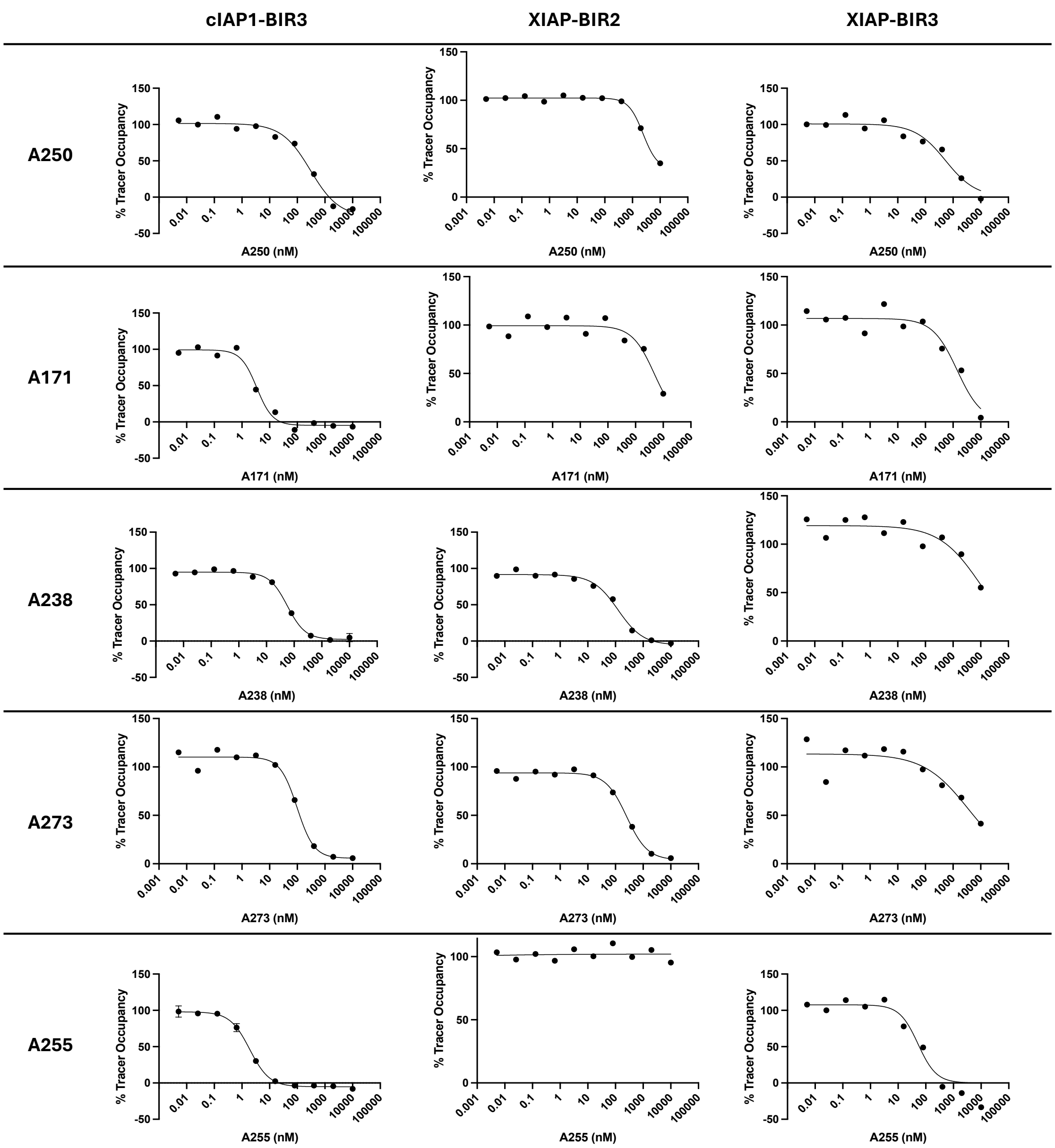

Compound	cIAP1-BIR3				XIAP-BIR2				XIAP-BIR3			
	TR-FRET IC_{50} (nM)				TR-FRET IC_{50} (nM)				TR-FRET IC_{50} (nM)			
	Mean	± SD	n	pIC_{50}	Mean	± SD	n	pIC_{50}	Mean	± SD	n	pIC_{50}
**A250**	241	± 79	2	6.6	3529	± 1055	3	5.5	789	± 361	2	6.1
**A171**	7	± 5	2	8.1	4778	-	1	5.3	1378	± 40	2	5.9
**A238**	157	± 101	3	6.8	118	± 15	3	6.9	>10,000	-	2	< 5.0
**A273**	98	± 1	2	7.0	250	± 34	3	6.6	4897	± 1927	2	5.3
**A255**	3.5	± 0.8	3	8.5	> 10,000	-	1	< 5.0	35	± 25	2	7.5

Supplementary Figure 1 (continued).

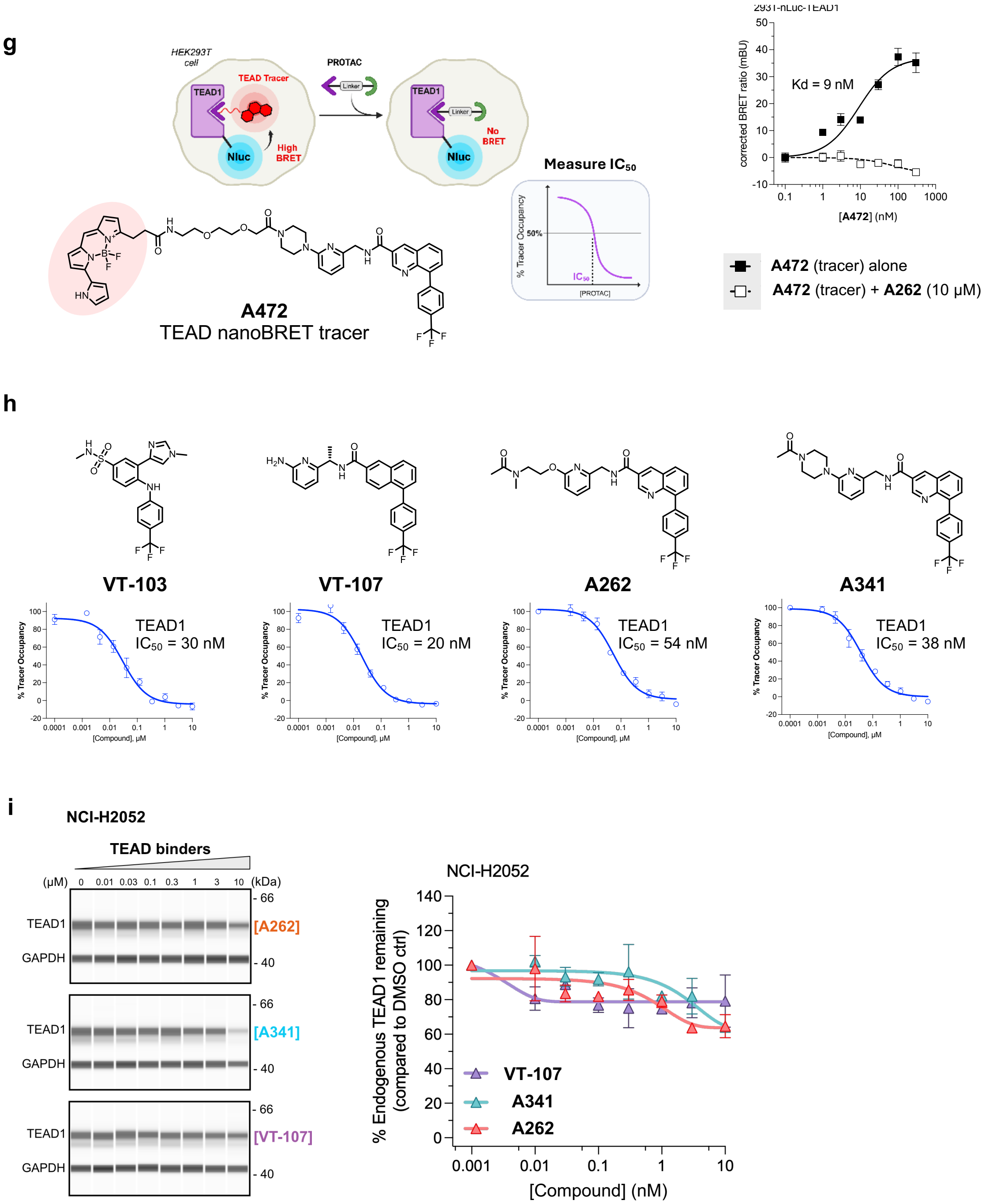

Supplementary Figure 1. Structural and binding benchmarking of IAP and TEAD binders and exit vectors. a Structural analysis of the published XIAP-BIR3/SMAC crystal structure (PDB 1G73)[1], highlighting the N-terminal 4 amino acid IAP-binding motif (IBM) of the SMAC protein (AVPI, pink) that engage corresponding pockets P1-P4 of XIAP-BIR3. XIAP is shown in electrostatic surface representation (red = negative electrostatic potential, blue = positive electrostatic potential). Renderings of all crystal structures were prepared in ChimeraX (v 1.4)[2]. **b** Domain structure of human cIAP1, cIAP2 and XIAP, showing positions of Baculoviral Repeat (BIR) domains 1-3, UBiquitin-Associated (UBA) domain, CAspase

Recruitment Domain (CARD) and the Really Interesting New Gene (RING) domain, which must be dimerized for E3 ligase activity. Model of cIAP1/2 activation upon binding of a SMAC mimetic (or bifunctional degrader recruiting a target), shifting the equilibrium from a compact inactive monomer to an active RING dimer, able to bind the ubiquitin-conjugated E2 and effect cIAP1/2 autoubiquitination (or target ubiquitination)[3, 4, 5]. In contrast, XIAP is a constitutive dimer and SMAC mimetic binding does not typically promote auto-ubiquitination[6, 7]. **c** Heat map of binding profiles of IAP ligands to individual recombinant BIR domains of cIAP1-BIR3, XIAP-BIR2^{C202A,C213G} and XIAP-BIR3 as measured using a competitive Time Resolved Fluorescence Resonance Energy Transfer (TR-FRET) binding assay. Values and color scale reflect half-maximal inhibitory constants (mean IC_{50}, reported as pIC_{50}, where $pIC_{50} = -\log_{10}IC_{50}$) as measured using TR-FRET assays based on competitive displacement of a biotinylated tracer from the relevant IAP domain. cIAP1-BIR3 and XIAP-BIR3 used a biotinylated SMAC peptide tracer, H-AVPIAQKSE-Lys(Biotin)-NH_2 (Mimotopes); for XIAP-BIR2 a custom biotinylated tracer **A191** was developed (Supplementary Information Synthetic Chemistry), based on XB2 ligand **A238**, which has high affinity for XIAP-BIR2. Summarized SPR and TR-FRET binding data and representative sensorgrams/fitted curves are shown in panels **e**, **f** and Supplementary Data 1. **d** Comparison of crystal structures of representative 'SMAC mimetic' IAP ligands bound to BIR3 domain of cIAP1 or XIAP, depicted as described in (**a**): XIAP-BIR3:**A250** (PDB 9N21, this work, ALP1 series), cIAP1-BIR3:**A273** (PDB 9N23, this work, XB2 series), cIAP1-BIR3:**A255** (PDB 5M6N)[8], cIAP1-BIR3:**LCL-161** (PDB 7TRM)[9]. Highlighted are features common to all ligands, including engagement of the critical basic group into the acidic P1 pocket, key hydrogen bonding interactions to the main chain backbone of IAP BIR domains, and locations of exit vectors selected to be represented in the TEAD IPD library (black arrows). **e** Representative SPR fitted sensorgrams and binding data for direct binding of compounds to individual IAP BIR domains. Dissociation constants (K_D) were determined from 1:1 kinetic fitting (black line), or steady state fitting (for examples with additional dose/response curve shown inset); tabulated values are mean ± SD for the stated number of independent experimental repeats (n). Additional repeats are shown in Supplementary Data 1. **f** Representative TR-FRET fitted binding data for competitive binding of compounds to individual IAP BIR domains; tabulated Half-maximal Inhibitory Constants (IC_{50}) values are mean ± SD for the stated number of independent experimental repeats (n). Additional repeats are shown in Supplementary Data 1. **g** A TEAD1 cellular target engagement (nanoBRET) assay was developed to measure engagement of ligands to the lipidic pocket (P-site) of TEAD1, based on expression of NanoLuc-TEAD1 in HEK293T cells and competitive displacement of fluorescent tracer **A472** (left panel, refer methods). As part of assay validation, direct titration of tracer **A472** yielded a measurable increase in nanoBRET signal with an estimated dissociation constant (K_D) of 9 nM, whilst pre-treatment with a saturating concentration of a competitor TEAD P-site ligand (**A262**, 10 µM) completely blocked nanoBRET signal (right panel). **h** Profiling of TEAD P-site ligands in the TEAD1 cellular target engagement assay confirmed that ligands **A262** and **A341** (which each incorporated an exit vector for degrader linker attachment) retained similar TEAD1 binding relative to literature molecules **VT-107** or **VT-103** and therefore were deemed suitable binders to include as part of constructing the TEAD1 IPD library. **i** TEAD binders **VT-107**, **A262** and **A341** were also evaluated in the screening cell line NCI-H2052 for their effect on endogenous TEAD1. NCI-H2052 cells were treated for 20 h with TEAD binders (7 concentration dose-response titration with three-fold serial dilution starting from 10 µM and DMSO vehicle control). Resulting cell lysates (RIPA buffer) were subjected to capillary western electrophoresis (left panel) and analysed for endogenous TEAD1 degradation with GAPDH as loading control and dose response curves (right panel) fitted using one phase decay model to calculate D_{max} and DC_{50} values.

Supplementary Figure 2

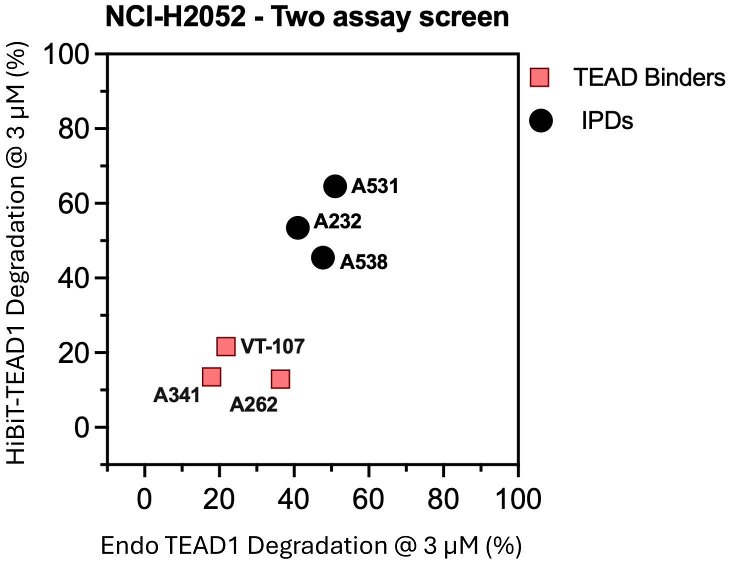

Supplementary Figure 2. **Comparison of TEAD1 degradation for IPD hits and TEAD binders for luciferase and endogenous screening formats.** Plotted are relative level of observed TEAD1 degradation using the luciferase (HiBiT-TEAD1) or endogenous TEAD1 degradation assay formats used in library screening. Values represent mean observed % TEAD1 degradation for TEAD IPD hits selected from degradation screening (**A232, A531 and A538;** black circles; n=2 and n=3 biologically independent experiments for endo and HiBiT assay respectively) or for TEAD binders (**A262**, **A341** and **VT107**; red squares; n=2 and n=1 biologically independent experiments for endo and HiBiT assay respectively). All compounds were tested at 3 µM concentration for 20 h in NCI-H2052 cells.

Supplementary Figure 3

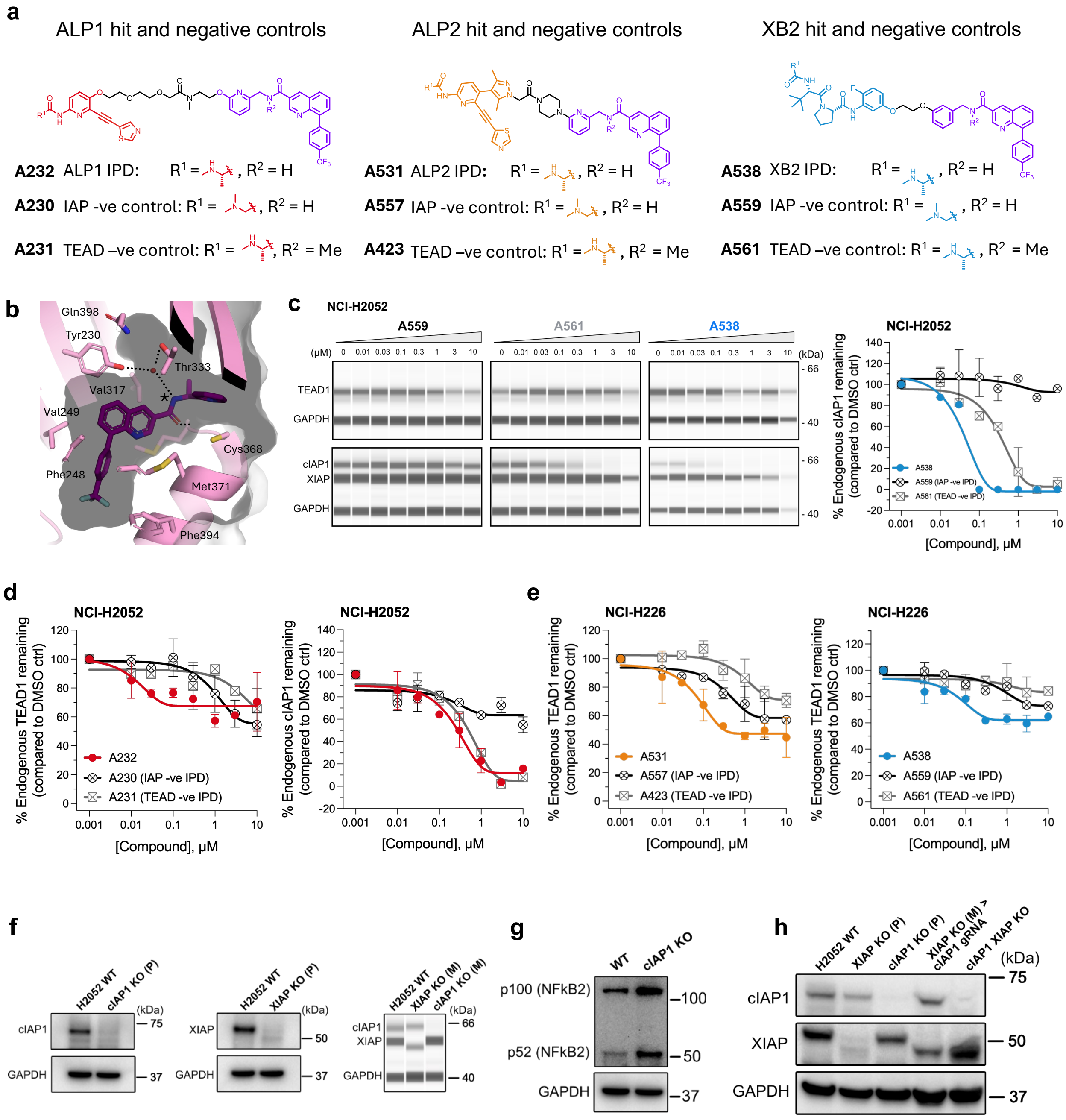

i

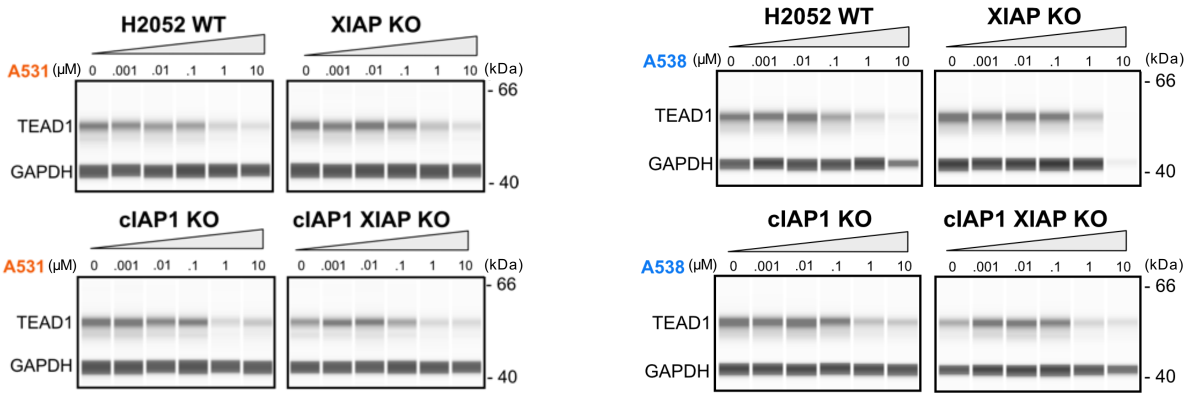

j

Cell line	ALP2 A531 (TEAD1)		XB2 A538 (TEAD1)	
	DC_{50} (nM)	D_{max} (%)	DC_{50} (nM)	D_{max} (%)
NCI-H2052 WT	120	55	64	48
NCI-H2052 *XIAP* KO	83	49	61	27
NCI-H2052 *cIAP1* KO	90	34	340	44
NCI-H2052 *cIAP1 XIAP* KO	39	33	220	28

Endogenous degradation post 20 hours compound (dose response) treatment was assessed using capillary based western assay for TEAD1. Data is mean of n=2 biological replicates.

k

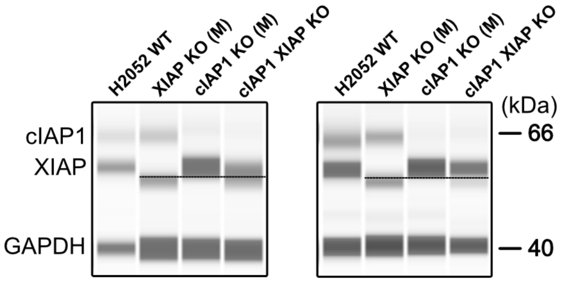

Supplementary Figure 3. IPD hit negative controls and detailed TEAD1 degradation profiling. a Chemical structures of TEAD IPD hits ALP1 **A232**, ALP2 **A531** and XB2 **A538** and corresponding IAP and TEAD negative control molecular matched pairs. **b Design of TEAD binder negative control.** Depiction of crystal structure of TEAD3^{YBD}:**VT-105** complex (PDB 7CNL; TEAD3 pink, **VT-105** purple) showing the central TEAD binder amide (marked *; amide present in **VT-105**, **VT-107** and related binders) and surface of the TEAD lipid pocket shown in light grey. The TEAD binder amide -NH that projects into a small pocket bounded by Val317, Tyr230, Thr333 of TEAD3, with a water-mediated interaction with Thr333 and the TEAD binder amide -NH, as well as weak hydrogen bond formed between the TEAD binder amide carbonyl and the backbone amide -NH of Cys368 of TEAD3. We predicted that replacement of the TEAD binder -NH with a methyl would disrupt this key water-mediated interaction, and likely alter the bound conformation of the TEAD ligand in the lipid pocket, including disturbing coplanarity of the TEAD binder amide and quinoline ring and the hydrogen bond formed with the backbone of Cys368.

c-e Indicated cell lines were treated with dose titration (7 concentrations with three-fold serial dilutions starting from 10 uM and DMSO control) of relevant compounds for 20 h. Further generated RIPA lysates were subjected to capillary western electrophoresis (JESS, Simple Western^{TM}) and analysed for endogenous protein levels (GAPDH as loading control) and dose response curves plotted. All data was acquired with n=2 biologically independent experiments. Represented in **c** Capillary western images for effect on endogenous TEAD1 (top left panel) and cIAP1 / XIAP levels (Bottom left panel) and on right cIAP1 autodegradation curves for XB2 hit **A538** and its matched IAP negative control, **A559** and TEAD negative control, **A561** in NCI-H2052 cells. **d** Dose response curves of ALP hit **A232** and its matched IAP negative control, **A230** and TEAD negative control, **A231** in NCI-H2052 cells for Endogenous TEAD1 degradation (left) and cIAP1 autodegradation (right). **e** Endogenous TEAD1 degradation curves in NCI-H226 cells for ALP hit **A531** and XB2 hit **A538** and their matched IAP and TEAD binding negative controls. **f Generation of IAP polyclonal and monoclonal CRISPR Cas9 KO.** Western blot depicting cIAP1 and XIAP levels in NCI-H2052 cells and NCI-H2052 Cas9 cells transduced with gRNA targeting cIAP1 (left panel) and XIAP (middle panel), with GAPDH as loading control. Right panel is capillary western images of RIPA lysates from NCI-H2052 wild type (WT), *cIAP1* monoclonal KO, *XIAP* monoclonal KO, probed with cIAP1 and XIAP antibodies along with GAPDH as loading control. **g Functional validation of *cIAP1* KO.** Western blot images showing NFkb2 p100, p52 and loading control GAPDH levels in NCI-H2052 wild type (WT) and *cIAP1* polyclonal KO cells. **h Generation of IAP double KO.** Western blot images showing cIAP1, XIAP and loading control GAPDH levels in NCI-H2052 wild type (WT), *XIAP* polyclonal KO, *cIAP1* polyclonal KO, *XIAP* monoclonal KO transduced with cIAP1 gRNA (no double KO) and *cIAP1* monoclonal KO transduced with XIAP gRNA (*cIAP1 XIAP* double KO). **i** Endogenous TEAD1 degradation after 20-hour compound treatment (5 concentrations with ten-fold serial dilutions starting from 10 uM and DMSO vehicle control) assessed in NCI-H2052 wildtype cells, *cIAP1* monoclonal KO, *XIAP* monoclonal KO and *cIAP1 XIAP* DKO cell lines and representative capillary western images shown for ALP hit **A531** and XB2 hit **A538. j** Table shows endogenous degradation values (D_{max} and DC_{50}) for TEAD1 by ALP hit **A531** and XB2 hit **A538** in NCI-H2052 wild type cells, *XIAP* monoclonal KO, *cIAP1* monoclonal KO and *cIAP1 XIAP* DKO cell lines. **K Effect of subculturing on double IAP KO phenotype.** RIPA lysates of double IAP KO (last lane), after three passages (left panel) and five passages (right panel) from generation were probed in capillary western analysis for XIAP and cIAP1 levels and compared with multiple passaged NCI-H2052 WT, *cIAP1* monoclonal KO, *XIAP* monoclonal KO cells. Dashed line represents the upper limit of the band for the mutant truncated XIAP. Any signal above this band indicates co-presence of cells containing WT XIAP that are outgrowing cells containing mutated XIAP (susceptible to cell death) in double IAP KO cell cultures. All KO western blots have at least n=2 biological replicates.

Supplementary Figure 4

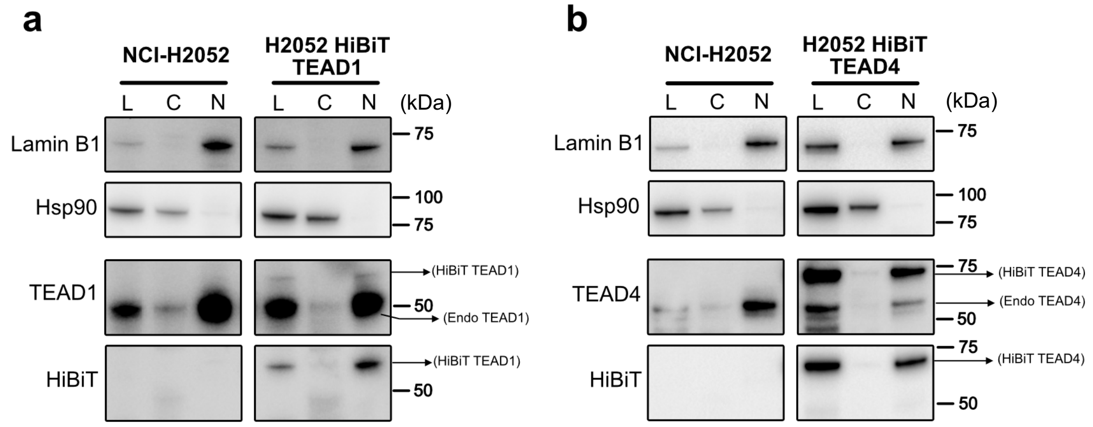

Supplementary Figure 4. Localization of POI TEAD in HiBiT tagged lines. Immunoblots of equivalent total cell lysate (L), cytosolic fraction (C) and nuclear fraction (N) were probed for nuclear marker (Lamin B1), cytosolic marker (Hsp90) target proteins (TEAD1 / TEAD4) and HiBiT tag in NCI-H2052 and NCI-H2052 HiBiT tagged TEAD1 cell line (left panel) and NCI-H2052 HiBiT tagged TEAD4 cell line (right panel). Arrows indicate the endogenous and HiBiT tagged versions of TEAD1 or TEAD4.

Supplementary Figure 5

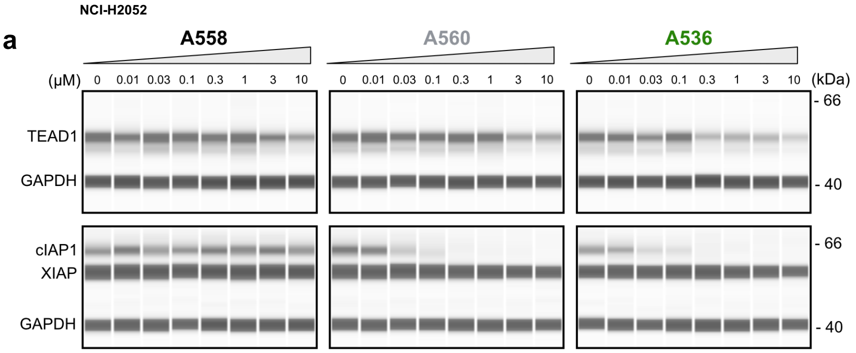

b

Compound	Description	NCI-H2052 (TEAD1)		NCI-H2052 (TEAD4)		NCI-H2052 (cIAP1)	
		DC_{50} (nM)	D_{max} (%)	DC_{50} (nM)	D_{max} (%)	DC_{50} (nM)	D_{max} (%)
**A536**	ALP IPD hit	110	51	990	24	56	98
**A558**	IAP neg ctl	2400	41	-	-	5	10
**A560**	TEAD neg ctl	11	20	-	-	99	94

Endogenous degradation post 20 hours compound (dose response) treatment was assessed using capillary based western assay. Data is mean of n=2 biological replicates.

- = Not tested

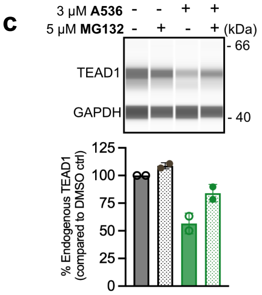

Supplementary Figure 5 (continued)

d

IAP cellular target engagement

A255

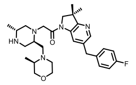

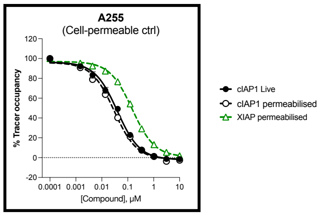

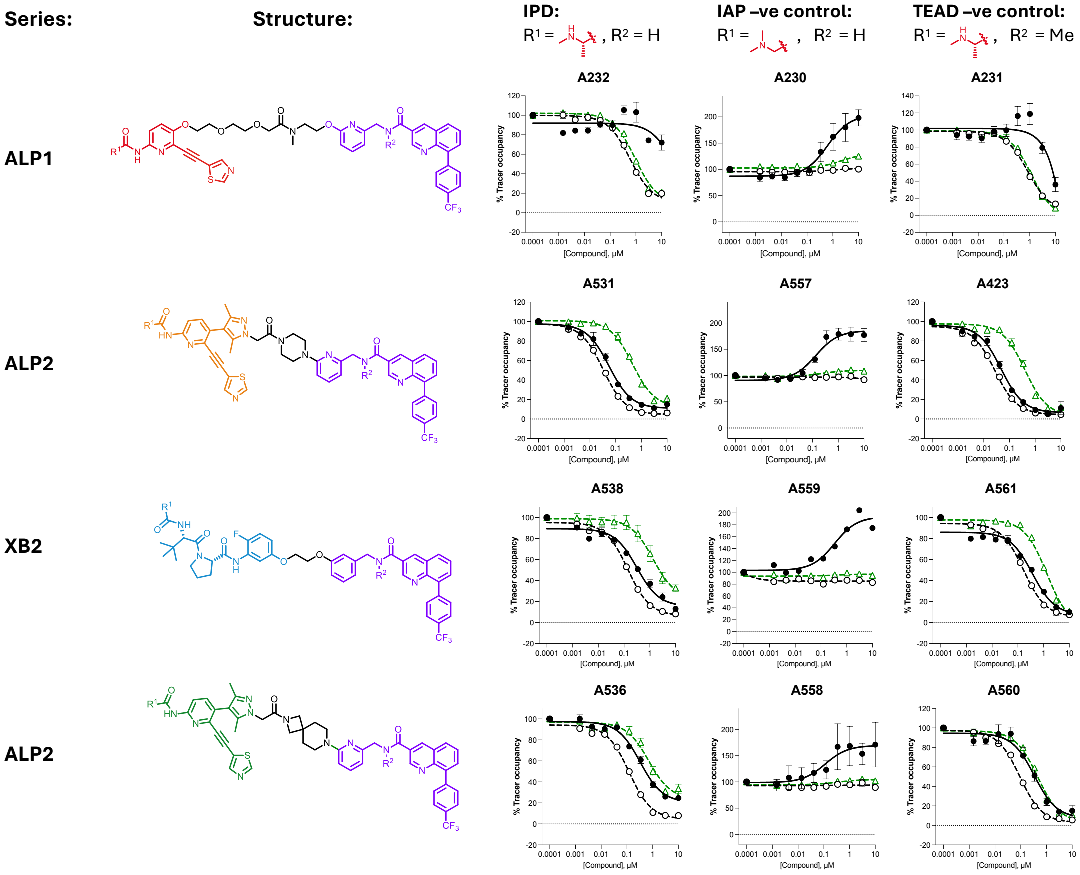

IAP binding in cellular target engagement NanoBRET assay.

Compound	cIAP1^{F616A} IC$_{50}$ (nM)		XIAP^{V461E} IC50 (nM)	RBA	IA
	Live	Permeabilised	Permeabilised		
**A255**	39	30	149	1.3	1
**A232**	>10,000	601	944	16.7	12.7
**A230**	NB	NB	NB	NA	NA
**A231**	>10,000	851	1196	11.8	9.0
**A531**	55	32	453	1.7	1.3
**A557**	NB	NB	NB	NA	NA
**A423**	48	27	403	1.8	1.3
**A538**	314	139	1349	2.3	1.7
**A559**	NB	NB	NB	NA	NA
**A561**	369	167	1342	2.2	1.7
**A536**	327	122	701	2.7	2.1
**A558**	NB	NB	NB	NA	NA
**A560**	339	92	451	3.7	2.8

Supplementary Figure 5 (continued)

e TEAD cellular target engagement

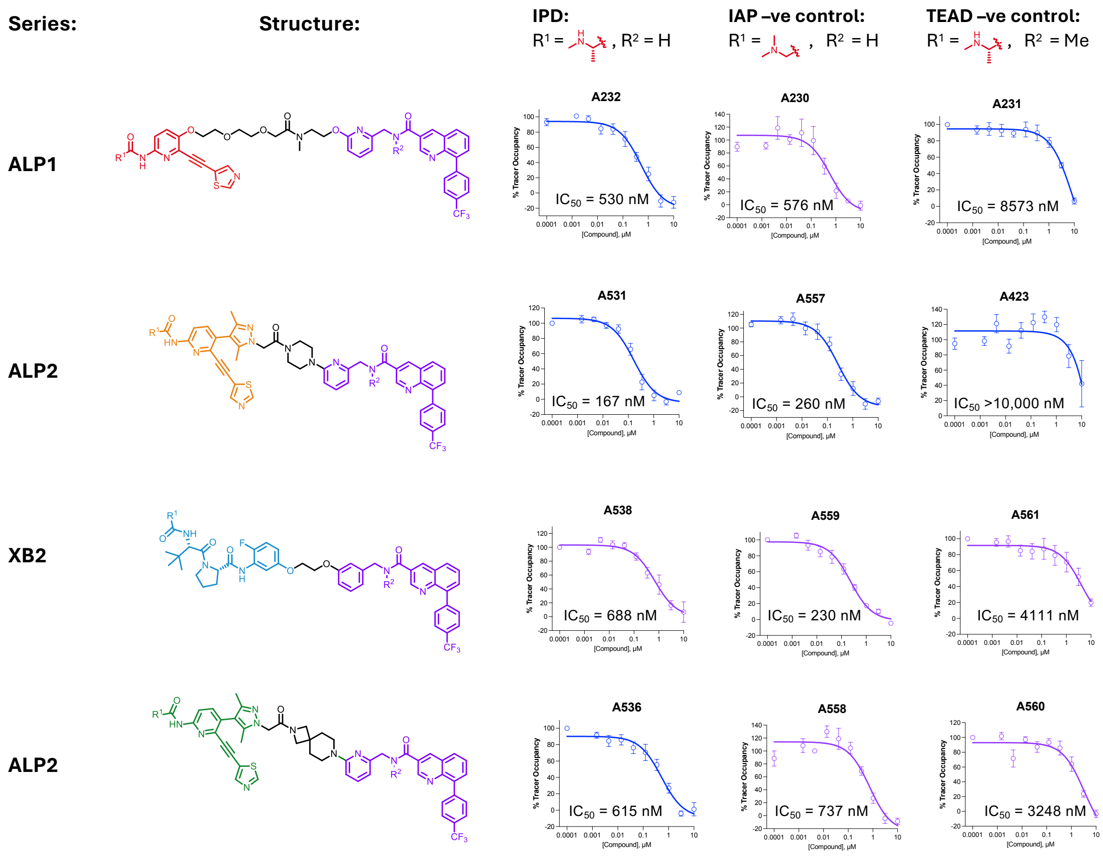

Supplementary Figure 5. a Cellular degradation profiling of A536 and matched negative controls. NCI-H2052 cells were treated with ALP2 spirocyclic linker IPD **A536** and its matched IAP- and TEAD-negative control IPDs (**A558** and **A560** respectively) at various doses for 20 h. RIPA lysates were generated and subjected to capillary western electrophoresis (JESS, Simple Western) to analyse endogenous TEAD-1 (top panel) cIAP1 / XIAP expression levels (bottom panel) along with GAPDH as loading control. **b** Table shows endogenous degradation values (D_{max} and DC_{50}) for TEAD1, TEAD4 and cIAP1 by ALP2 **A536** and corresponding IAP and TEAD negative controls in the NCI-H2052 cell line. **c** Top panel shows representative western blot analysis of NCI-H2052 cells treated for 16 h with DMSO, 5 uM proteasome inhibitor MG132 and 3 uM compound **A536** in absence or presence of 5uM MG132. Bottom panel shows bar graph of % endogenous TEAD1 degradation relative to DMSO treated cells. Error bars represent mean $\pm$ SD of n=2 biologically independent experiments. **d** A cellular IAP target engagement assay was developed based on displacement of a fluorescent IAP tracer **B678** from NanoLuc-tagged $cIAP1_{184\text{-}618}^{F616A}$ or $XIAP_{124\text{-}497}^{V461E}$ (HEK293T cells) and nanoBRET signal measured following treated in dose-response with IAP binders, IPDs or matched IPD IAP- or TEAD- negative controls (top panel; Fig. 5d). Percentage tracer occupancy (based on nanoBRET signal, normalised to DMSO vehicle) was measured for live cells (cIAP1 and XIAP) or cells permeabilised by pre-treatment with digitonin (cIAP1 only). Plotted data represent mean ± SD for n=3 biologically independent experiments. For cIAP1, a cellular Availability Index (AI) was determined by first comparing the fitted IC_{50} values in live and permeabilised modes to obtain a Relative intracellular availability (RBA) value, then normalizing this to ASX series IAP ligand **A255**, selected as a cell-permeable control compound with high affinity to BIR3 of cIAP1 and XIAP. cIAP1 was used for AI determination as most compounds tested have potent cIAP1 binding. Larger AI values represent lower intracellular availability relative to the permeable control **A255**. Bottom panel: tabulated IC_{50}, RBA and AI values for ASX series IAP binder reference **A255**, IPDs and matched negative control IPDs. **e** IPDs and matched IAP- and TEAD- negative control IPDs were profiled relative in the NanoLuc-TEAD1 cellular target engagement assay for displacement of a fluorescent tracer **A472** from the TEAD1 P-site (refer Fig.1f) and fitted to determine and cellular IC_{50}. Fitted data represent mean ± SD from n=3 biologically independent experiments.

Supplementary Figure 6

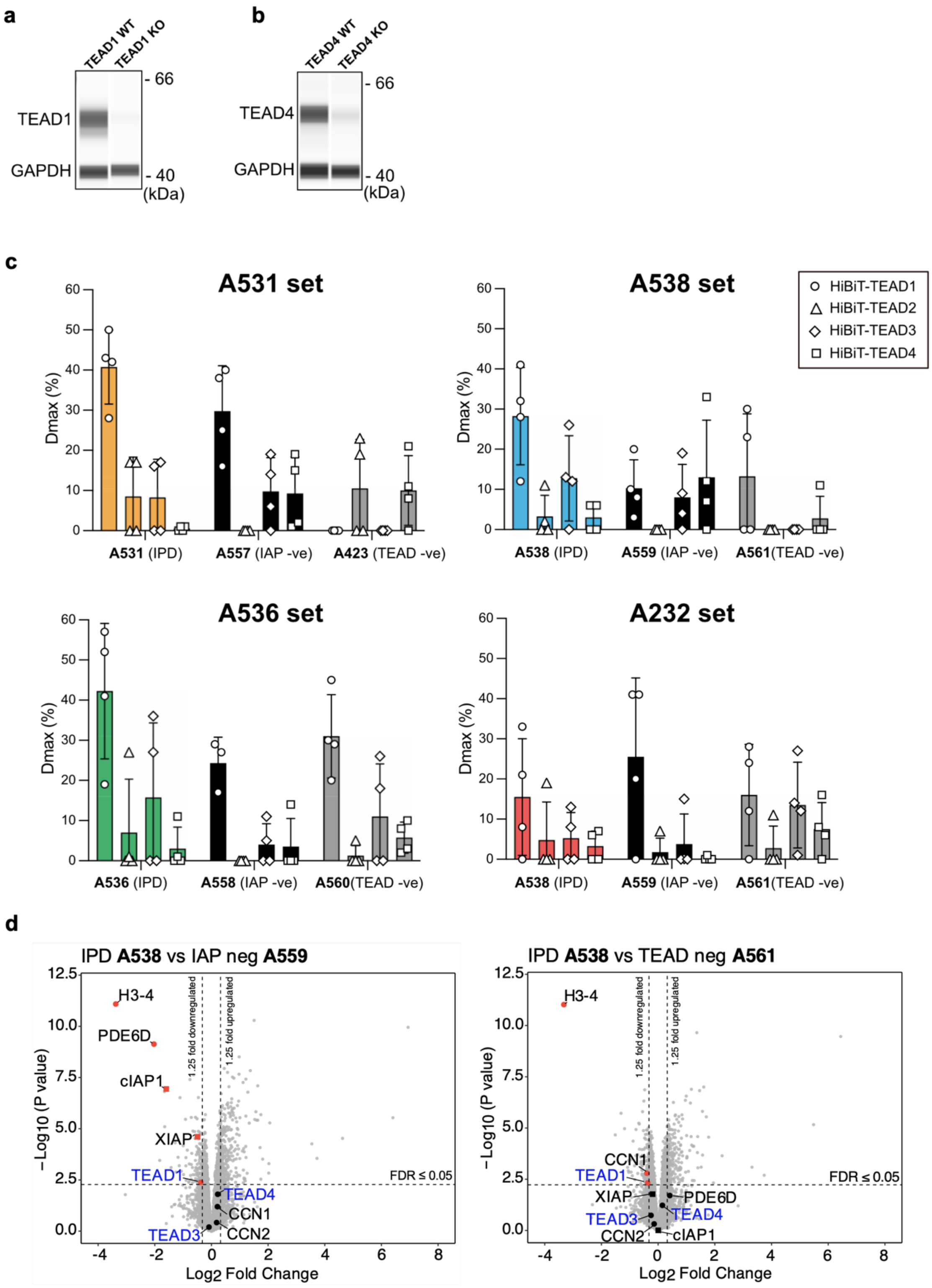

Supplementary Figure 6 (continued)

e

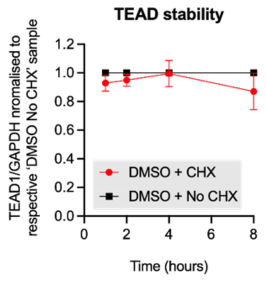

f

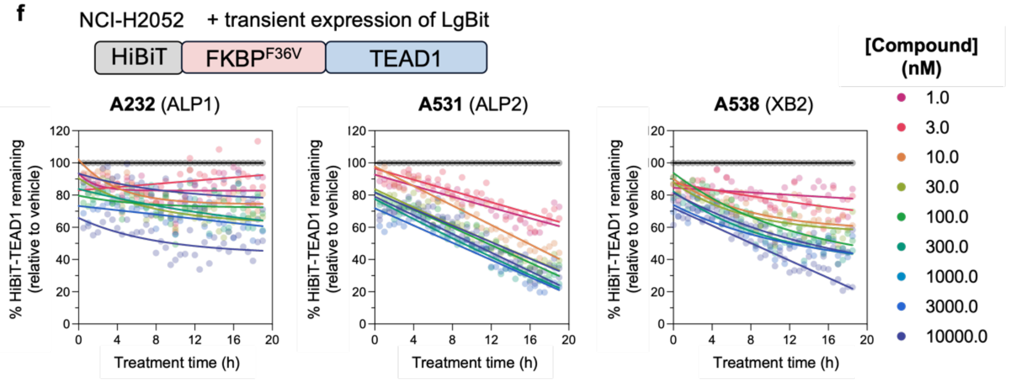

Supplementary Figure 6. a-b Validation of TEAD western antibodies. RIPA lysates were subjected to capillary western electrophoresis, probed with target antibody and GAPDH antibody as loading control. Represented in (a) NCI-H2052 wildtype cells and *TEAD1* CRISPR KO (sorted) cells probed with TEAD1 antibody and in (b) HEK293T wildtype cells and *TEAD4* CRISPR KO (unsorted) cells probed with TEAD-4 antibody. Data is representative of n=2 biologically independent experiments. **c** TEAD paralogue specificity profiling measuring degradation (Dmax) in HiBiT TEAD1-4 tagged NCI-H226 cells for IPD hits (coloured) and their matched IAP (black) and TEAD (grey) negative controls in an 18 h dose response experiment, normalized to CTG viability assay. Each symbol indicates a tagged HiBiT- TEAD paralogue. Data are representing mean ± SD of n=4 biologically independent experiments, each with two technical replicates. **d** Volcano plots showing relative protein abundance (log2 fold change) vs significance (-log10 p-value) of quantified proteins in NCI-H2052 cells treated for 16 h with 0.5 µM of **A538** vs matched IAP negative control **A559** or TEAD negative control **A561**. Proteins significantly altered lie above the horizontal dashed line (adjusted p-value 'or' FDR $\leq$ 0.05) and beyond vertical cut off lines (left, 1.25 times downregulated; right 1.25 times upregulated in **A538** treated cells). **e Half-life of TEAD1.** NCI-H226 cells were treated with 100 µg/ml of Cycloheximide (CHX) or only DMSO for 0, 2, 4, 6 and 8 h. RIPA lysates subjected to capillary western electrophoresis were probed with TEAD1 and loading control GAPDH antibodies. Graph represents mean TEAD level (normalized to GAPDH) over various time points comparing CHX treated (red dots) and untreated samples (black dots). Each data point represents Mean ± SD from n=2 biologically independent experiments, with two technical replicates within each experiment. **f Kinetics of HiBiT-TEAD1 degradation by IPDs**. NCI-H2052 cells stably expressing HiBiT-TEAD were transiently transfected with LgBit (Promega) for complementation of the active nanoluciferase were treated in a time course experiment with a dose-response of IPDs (**A232**, **A531** or **A538**) or DMSO vehicle control as indicated, as well as Vivazine substrate (Promega). Percentage of HiBiT-TEAD1 remaining (measured luminescence time) was plotted relative to DMSO vehicle. Data represent a single experiment (n=1; 2 technical replicates).

Supplementary Tables

Supplementary Table 1: Crystallographic data collection and refinement statistics.

	XIAP-BIR3 : A171 (PDB 9N1R)	XIAP-BIR3 : A250 (PDB 9N21)	cIAP1-BIR3 : A273 (PDB 9N23)
**Data collection**[a]			
Space group	$P4_1\ 2_1\ 2$	$P4_1\ 2\ 2$	$P2_1\ 2_1\ 2_1$
Cell dimensions			
a, *b*, *c* (Å)	99.71, 99.71, 105.43	71.24, 71.24, 105.55	30.25, 68.39, 124.22
a, b, g (°)	90, 90, 90	90, 90, 90	90, 90, 90
Resolution (Å)	45.07 - 2.8 (3.02 - 2.8)[b]	42.41 - 2.74 (3.14 - 2.74)[b]	35.42 - 1.8 (1.87 - 1.8)[b]
R_{merge}	0.268 (5.356)	0.130 (1.528)	0.327 (5.520)
$I / \sigma I$	14.6 (2.0)	16.3 (3.6)	9.0 (1.3)
Completeness (%)	100.0 (100.0)	99.5 (95.4)	99.9 (99.9)
Redundancy	26.4 (27.3)	25.4 (24.1)	13.2 (13.2)
**Refinement**			
Resolution (Å)	45.07 - 2.80	42.41 - 2.74	35.42 - 1.80
No. reflections	13589	7573	24758
R_{work} / R_{free}	0.2049 / 0.2128	0.2015 / 0.2189	0.1932/ 0.2225
No. atoms	1761	871	1692
Protein	1694	839	1539
Ligand/ion	56	21	64
Water	11	11	89
B-factors	80.78	75.61	26.98
Protein	80.60	75.61	26.52
Ligand/ion	89.02	84.22	31.13
Water	65.98	59.22	31.94
R.m.s. deviations			
Bond lengths (Å)	0.010	0.008	0.007
Bond angles (°)	1.13	1.02	1.04

[a]Data are from one crystal for each structure.

[b]Values in parentheses are for the highest-resolution shell

Synthetic Chemistry

General Methods

Commercially available dry solvents were used from Sigma Aldrich. All reagents unless otherwise noted were commercially available and purchased from Sigma Aldrich, Combi Blocks, BLD, ABCR, Fluorochem, Activate, Thermo Fisher or Enamine, at least 95% pure and used without further purification.

Normal phase TLC was carried out on pre-coated silica plates (Kieselgel 60 F254, BDH) with visualization via UV light (UV 254 and/or 365 nm) and/or basic potassium permanganate solution. Flash column chromatography was performed using either a Teledyne Isco Combiflash Rf with prepacked Redisep silica gel columns (particle size 0.040-0.063mm).

Strong cation exchange (SCX) chromatography was carried out using Biotage Isolute SCX-2 columns. NMR Spectra were recorded on a Bruker Avance DRX 300 MHz NMR System. Chemical shifts are quoted in ppm and referenced to the residual solvent signals: ^{1}H NMR δ (ppm) = 7.26 ($CDCl_3$-*d*), ^{13}C NMR δ (ppm) = 77.2 ($CDCl_3$-*d*), ^{1}H NMR δ (ppm) = 2.50 (DMSO-d_6), ^{13}C NMR δ (ppm) = 39.5 (DMSO-d_6), ^{1}H NMR δ (ppm) = 3.31 (MeOD-d_4), ^{13}C NMR δ (ppm) = 49.0 (MeOD-d_4). Signal splitting patterns are described as singlet (s), doublet (d), triplet (t), quartet (q), quintet (quin.), multiplet (m), broad (b) or a combination thereof. Coupling constants (*J*) are measured in Hertz (Hz).

Analytical MS Methods and Instrumentation

Method 1: HRMS data were recorded on a ThermoFisher Exactive plus mass spectrometer coupled to an Ultimate 3000 HPLC system. The HPLC system was setup to directly infuse (no separation column) the sample into the standard HESI source. Sample dilution: 10 mM DMSO stock solution was diluted 1:20 in acetonitrile. 1 µL of the diluted sample was injected by the autosampler. The carrier solvent was 50% acetonitrile, 50% water, containing 0.1% formic acid at a flow rate of 0.1ml/min and a total run time of 60s. For the source, spray voltage was 4000V, capillary temperature was 320°C. The mass spectrometer was set to a mass range of 200 to 2000 m/z, with a resolution of 140,000, an AGC target of 5e5 and a maximum IT of 50ms.

Method 2: LCMS data were recorded on an Agilent G6120B MSD, using 1260 Infinity II G7129B binary pump with 1290 detector Infinity G71172B DAD, the LC conditions as follows, column Luna, Omega 3.0 µM PS C18 100 A, 50 x 2.1 mm; injection volume: 1 µL; flow rate 0.6 ml/min; gradient 5-100 %of B over 3.8 min, (Solvent A: water, 0.1% formic acid; solvent B: acetonitrile, 0.1% formic acid). MS conditions were as follows, ion source: single-quadrupole, ion mode: ES positive unless otherwise specified, source temperature: 150 °C, desolvation temperature: 350 °C, detection: ion counting, Capillary (KV)-3.00, Cone(V): 30, Extractor (V): 3, RF Lens (V): 0.1, Scan Range: 100-1000 Amu, Scan Time: 0.5 sec, Acquisition time: 4.1 min, Gas flow: desolvation L/hr-650, Cone L/hr-100.

Preparative Purification Methods and Instrumentation:

Method 3: Mass-directed auto-preparative HPLC conditions were as follows, column: XBridge TM prep C18 5 µm 19x100 mm; injection volume: 500 µL; flow rate 20 mL/min; gradient: 30-100% of B over 15 min, (Solvent A: water, 0.1% formic acid; solvent B: acetonitrile, 0.1% formic acid); detection: AcQuity QDA mass detector and Waters 2998 photodiode array detector. MS conditions were as follows, ion

source: single-quadrupole, ion mode: ES positive unless otherwise specified, source temperature: 150 °C, desolvation temperature: 350 °C, detection: ion counting, Capillary (KV)-3.00, Cone(V): 30, Extractor (V): 3, RF Lens (V): 0.1, Scan Range: 150-1250 Amu, Scan Time: 0.5 sec, Acquisition time: 17 min, Gas flow: desolvation L/hr-650, Cone L/hr-100.

Method 4: Mass-directed auto-preparative HPLC conditions were as follows, column: Phenomenex Luna Omega 5 µm PS C18 21x100 mm; injection volume: 800 µL; flow rate 20 mL/min; gradient: 30-100% of B over 20 min, (Solvent A: water, 0.1% formic acid; solvent B: acetonitrile, 0.1% formic acid); detection: Waters 3100 mass detector and Waters 2996 photodiode array detector. MS conditions were as follows, ion source: single-quadrupole, ion mode: ES positive unless otherwise specified, source temperature: 150 °C, desolvation temperature: 350 °C, detection: ion counting, Capillary (KV)-3.00, Cone(V): 30, Extractor (V): 3, RF Lens (V): 0.1, Scan Range: 150-1250 Amu, Scan Time: 0.5 sec, Acquisition time: 20 min, Gas flow: desolvation L/hr-650, Cone L/hr-100.

General Synthetic Procedures 1-2

General procedure 1: Boc deprotection with TFA

To a solution of Boc-protected amine (1 eq.) in DCM (2 mL) at 0 °C was added TFA (20 eq.) and the mixture was left to stir at rt for 18 h. The progress of the reaction was monitored by LCMS. The mixture was aspirated and subjected to acidic RP HPLC according to the General Method. Pure, product-containing fractions were combined, then concentrated, and further freeze dried to obtain the desired product.

General procedure 2: Amide coupling

To a solution of the carboxylic acid (1 eq.) and amine (1.1 eq.) in DMF (1 mL) was added DIPEA (5 eq.) followed by HATU (1.5 eq.) The reaction was left to stir at rt and the progress of the reaction was monitored by LCMS. The mixture was diluted with EtOAc and washed with chilled brine. The organic layer was dried over $MgSO_4$ and concentrated under reduced pressure. The crude was purified by column chromatography to obtain the desired product.

Synthesis of IAP Binders

*Synthesis of **A250***

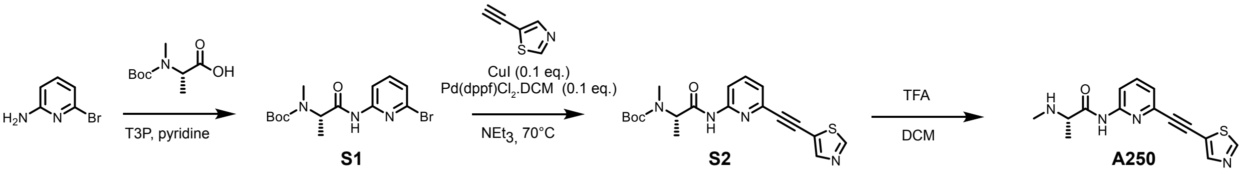

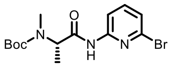

***tert*-butyl N-[(1*S*)-2-[(6-bromo-2-pyridyl)amino]-1-methyl-2-oxo-ethyl]-N-methyl-carbamate (S1):** To T3P (50% in EtOAc) (13.8 mL, 23.1 mmol, 1.9 eq.) and Pyridine (47 mL) were dissolved 6-bromopyridin-2-amine (2000 mg, 12 mmol, 1 eq.) and then (2*S*)-2-[*tert*-butoxycarbonyl(methyl)amino]propanoic acid (2820 mg, 13.9 mmol, 1.1 eq.) and stirred at rt overnight. LCMS showed some residual amine SM, therefore 0.1 eq. of acid and T3P were added. LCMS showed the same profile, therefore the reaction was diluted with water and extracted with EtOAc. The organic layer was washed with water (x5) and dried over $MgSO_4$ and concentrated. The residue was purified by column chromatography using 0-30% EtOAc in heptane to give product as a white solid (2000 mg, 48% yield).

^{1}H NMR (300 MHz, $CDCl_3$) δ 8.44 (s, 1H), 8.17 (dd, *J* = 8.2, 0.8 Hz, 1H), 7.55 (t, *J* = 7.9 Hz, 1H), 7.21 (dd, *J* = 7.7, 0.7 Hz, 1H), 4.76 (bs, 1H), 2.84 (s, 3H), 1.49 (s, 9H), 1.41 (d, *J* = 7.1 Hz, 3H).

LCMS (Method 2) RT = 2.54 min, $[M+H]^+$ = 358.2/360.2

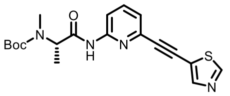

***tert*-butyl *N*-methyl-*N*-[(1*S*)-1-methyl-2-oxo-2-[[6-(2-thiazol-5-ylethynyl)-2-pyridyl]amino]ethyl]carbamate (S2)**: *tert*-butyl *N*-[(1*S*)-2-[(6-bromo-2-pyridyl)amino]-1-methyl-2-oxo-ethyl]-*N*-methyl-carbamate (**S1**) (90 mg, 0.25 mmol, 1 eq.) and 5-ethynylthiazole (0.030 mL, 0.30 mmol, 1.2 eq.) was dissolved in Triethylamine (1.8 mL) and degassed with N_2. Copper (I) iodide (4.8 mg, 0.025 mmol, 0.1 eq.) and Pd(dppf)Cl_2.DCM (21 mg, 0.025 mmol, 0.1 eq.) was added and heated to 70 °C overnight. When LCMS showed complete conversion, the reaction mixture was diluted with EtOAc and water. 10% citric acid was added until pH = 4 and extracted (x2). The organic layer was washed with brine, dried with $MgSO_4$ and evaporated to give crude product which was separated on flash chromatography (0-50% EtOAc in Heptane) to give product (87 mg, 90% yield).

^{1}H NMR (300 MHz, $CDCl_3$) δ 8.80 (s, 1H), 8.53 (s, 1H), 8.27 (dd, *J* = 8.5, 0.9 Hz, 1H), 8.16 (s, 1H), 7.73 (t, *J* = 8.0 Hz, 1H), 7.30 (dd, *J* = 7.5, 0.9 Hz, 1H), 4.77 (bs, 1H), 2.85 (s, 3H), 1.48 (s, 9H), 1.43 (d, *J* = 7.0 Hz, 3H).

LCMS (Method 2) RT = 2.42 min, $[M+H]^+$ = 387.2

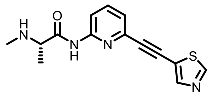

(2*S*)-2-(methylamino)-*N*-[6-(2-thiazol-5-ylethynyl)-2-pyridyl]propenamide (A250): *tert*-butyl *N*-methyl-*N*-[(1*S*)-1-methyl-2-oxo-2-[[6-(2-thiazol-5-ylethynyl)-2-pyridyl]amino]ethyl]carbamate (87 mg, 0.23 mmol, 1 eq.) was dissolved in DCM (3 mL) and TFA (0.5 mL) added. Stirred at rt for 4 h. When LCMS showed complete conversion, the reaction mixture was concentrated. The residue was diluted with EtOAc and water. Sat. $NaHCO_3$ was added so that pH = 9 and extracted with EtOAc (x2). The organic layer was washed with brine, dried with $MgSO_4$ and evaporated to give crude product. The crude residue separated on flash chromatography (0-5% MeOH in DCM) to give product as an off-white solid (48 mg, 74% yield).

^{1}H NMR (300 MHz, $CDCl_3$) δ 9.86 (s, 1H), 8.76 (s, 1H), 8.29 (d, *J* = 8.3 Hz, 1H), 8.11 (s, 1H), 7.69 (t, *J* = 8.0 Hz, 1H), 7.26 (d, *J* = 7.4 Hz, 1H), 3.16 (q, *J* = 7.0 Hz, 1H), 2.42 (s, 3H), 1.35 (d, *J* = 7.0 Hz, 3H).

^{13}C NMR (75 MHz, $CDCl_3$) δ 174.4, 154.4, 151.7, 148.4, 140.5, 138.7, 123.3, 118.1, 114.1, 95.0, 78.5, 61.1, 35.5, 19.4.

LCMS (Method 2) RT = 1.12 min, $[M+H]^+$ = 287.2

HRMS (ESI+) *m/z:* $[M+H]^+$ calcd for $C_{14}H_{15}N_4OS$ 287.0961; found 287.0952.

*Synthesis of **A171***

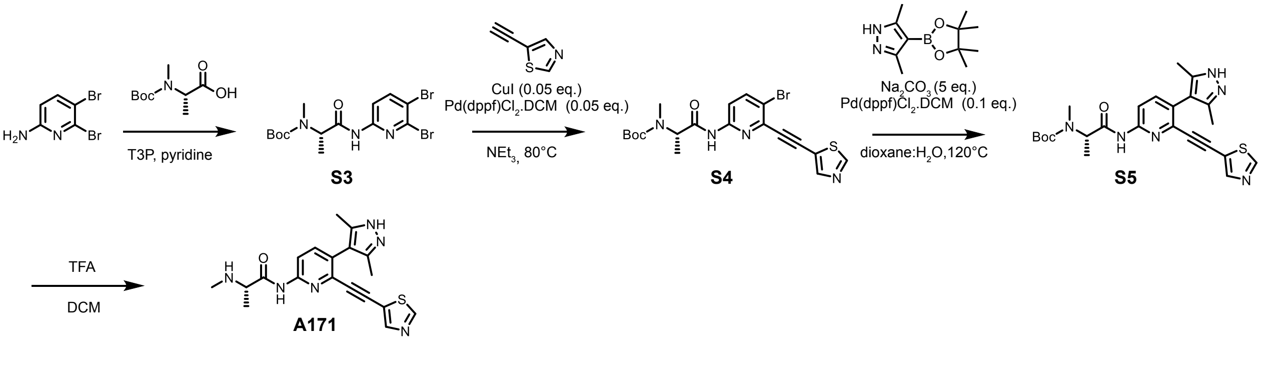

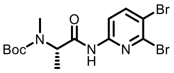

(*S*)-*tert*-butyl (1-((5,6-dibromopyridin-2-yl)amino)-1-oxopropan-2-yl)(methyl)carbamate (S3): A solution of 5,6-dibromopyridin-2-amine (10 g, 39.697 mmol, 1 eq.) and *N*-(*tert*-butoxycarbonyl)-*N*-methyl-L-alanine (16.14 g, 79.39 mmol, 2 eq.) in pyridine (50 mL) was cooled to -20 °C and stirred for 10 min. After that, T3P (60.15 mL, 198.48 mmol, 5 eq.) was added to the reaction mixture at -20°C dropwise. Reaction was stirred for 4 h at -20 °C. Progress of reaction was monitored by TLC and LCMS. After consumption of the starting material, the reaction mixture was diluted with EtOAc, and washed with water, and then brine. The organic layer was concentrated to give the crude product which was

purified by combi flash chromatography, eluting with 0-20% ethyl acetate in pet ether to give product as a white solid (14 g, 81% yield).

^{1}H NMR (300 MHz, $CDCl_3$) δ 8.54 (s, 1H), 8.08 (d, *J* = 8.6 Hz, 1H), 7.84 (d, *J* = 8.6 Hz, 1H), 5.07 – 4.29 (m, 1H), 2.83 (s, 3H), 1.48 (s, 9H), 1.41 (d, *J* = 7.0 Hz, 3H).

LCMS (Method 2) RT = 2.49 min, $[M+H]^+$ = 435.8

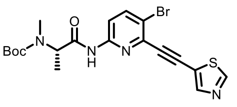

(*S*)-*tert*-butyl (1-((5-bromo-6-(thiazol-5-ylethynyl)pyridin-2-yl)amino)-1-oxopropan-2-yl)(methyl)carbamate (S4): A stirred solution of *tert*-butyl (*S*)-(1-((5,6-dibromopyridin-2-yl)amino)-1-oxopropan-2-yl)(methyl)carbamate (**S3**) (8 g, 18.30 mmol, 1 eq.) and 5-ethynylthiazole (2.60 g, 23.79 mmol, 1.3 eq.) in triethylamine (80 mL) in a closed vessel was degassed with N_2. After 15 min, CuI (0.17 g, 0.92 mmol, 0.05 eq.) and Pd(dppf)Cl_2 (0.64 g, 0.92 mmol, 0.05 eq.) was added to the reaction mixture and then stirred at 80 °C for 16 h. The reaction was monitored by LCMS and TLC. After complete consumption of SM, the reaction mixture was diluted with water and EtOAc and filtered through a Celite pad. The organic layer was washed with water, brine, and then dried over anhydrous Na_2SO_4 and concentrated under reduced pressure to give crude product, which was purified by flash chromatography using 230-400 silica, 0-30% EtOAc/pet ether as the mobile phase. Collected pure fractions were concentrated under reduced pressure to give product as a white solid (5.1 g, 59% yield).

^{1}H NMR (300 MHz, $CDCl_3$) δ 8.83 (s, 1H), 8.54 (s, 1H), 8.23 – 8.12 (m, 2H), 7.90 (d, *J* = 8.9 Hz, 1H), 4.96 – 4.51 (m, 1H), 2.84 (s, 3H), 1.48 (s, 9H), 1.42 (d, *J* = 7.0 Hz, 3H).

LCMS (Method 2) RT = 2.54 min, $[M+H]^+$ = 464.8

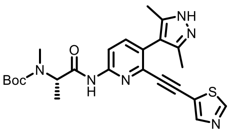

***tert*-butyl ((2*S*)-1-((5-(3,5-dimethyl-1*H*-pyrazol-4-yl)-6-(thiazol-5-ylethynyl)pyridin-2-yl)amino)-1-oxopropan-2-yl)(methyl)carbamate (S5):** To a solution of (*S*)-*tert*-butyl (1-((5-bromo-6-(thiazol-5-ylethynyl)pyridin-2-yl)amino)-1-oxopropan-2-yl)(methyl)carbamate (**S4**) (2.5 g, 5.37 mmol, 1 eq.) in dioxane: H_2O (30 mL) at rt, was added 3,5-dimethyl-4-(4,4,5,5-tetramethyl-1,3,2-dioxaborolan-2-yl)-1*H*-pyrazole (2.4 g, 10.74 mmol, 2.0 eq.) and Na_2CO_3 (2.9 g, 26.86 mmol, 5 eq.). The reaction mixture was degassed for 15 min at rt. After this Pd(dppf)Cl_2.DCM (0.5 g, 0.54 mmol, 0.1 eq.) was added to the reaction mixture at rt and stirred for 16 h at 120 °C. Progress of the reaction was monitored by TLC and LCMS. After complete consumption of SM, the reaction mixture was diluted with water and extracted with EtOAc. The organic layer was washed with water, brine, and then dried over anhydrous Na_2SO_4 and

concentrated under reduced pressure to give crude product, which was purified by flash chromatography using 230-400 silica, 0-70% acetone/pet ether as the mobile phase. Collected pure fractions were concentrated under reduced pressure to give product as an off white solid (1.25 g, 51% yield).

^{1}H NMR (300 MHz, $CDCl_3$) δ 8.74 (s, 1H), 8.59 (s, 1H), 8.31 (d, *J* = 8.5 Hz, 1H), 7.97 (s, 1H), 7.62 (d, *J* = 8.6 Hz, 1H), 5.10 – 4.45 (m, 1H), 2.86 (s, 3H), 2.23 (s, 6H), 1.50 (s, 9H), 1.44 (d, *J* = 7.1 Hz, 3H).

LCMS (Method 2) RT = 2.14 min, $[M+H]^+$ = 481.0

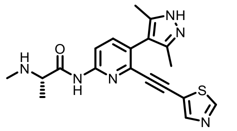

(2*S*)-*N*-[5-(3,5-dimethyl-1*H*-pyrazol-4-yl)-6-(2-thiazol-5-ylethynyl)-2-pyridyl]-2-(methylamino)propenamide (A171): *tert*-butyl *N*-[(1*S*)-2-[[5-(3,5-dimethyl-1H-pyrazol-4-yl)-6-(2-thiazol-5-ylethynyl)-2-pyridyl]amino]-1-methyl-2-oxo-ethyl]-*N*-methyl-carbamate (60.0 mg, 0.125 mmol, 1 eq.) was dissolved in DCM (1 mL) and treated with a solution of TFA (1.0 mL, 13 mmol, 104 eq.) in DCM (4 mL). The reaction was stirred at rt overnight, after which LCMS showed full conversion to the product. The residue was partitioned between DCM and sat. $NaHCO_3$. The aqueous phase was extracted twice with DCM and the combined organic phases were washed once with brine. The organic phase was dried (Na_2SO_4) and concentrated under reduced pressure to a yellow oil that was triturated to obtain the title product as a white solid (37.0 mg, 78% yield).

^{1}H NMR (300 MHz, DMSO-d_6) δ 12.43 (s, 1H), 9.19 (s, 1H), 8.22 (d, *J* = 8.6 Hz, 1H), 8.11 (s, 1H), 7.78 (d, *J* = 8.6 Hz, 1H), 3.23 (q, *J* = 6.9 Hz, 1H), 2.26 (s, 3H), 2.12 (s, 6H), 1.21 (d, *J* = 6.8 Hz, 3H).

^{13}C NMR (75 MHz, DMSO-d_6) δ 175.3, 157.2, 150.8, 148.4, 141.5, 139.3, 128.8, 117.6, 114.1, 113.7, 95.8, 82.5, 80.3, 60.0, 34.6, 25.2, 19.2.

LCMS (Method 2) RT = 0.93 min, $[M+H]^+$ = 381.2

HRMS (ESI+) *m/z:* $[M+H]^+$ calcd for $C_{19}H_{21}N_6OS$ 381.1498; found 381.1480.

*Synthesis of **A238***

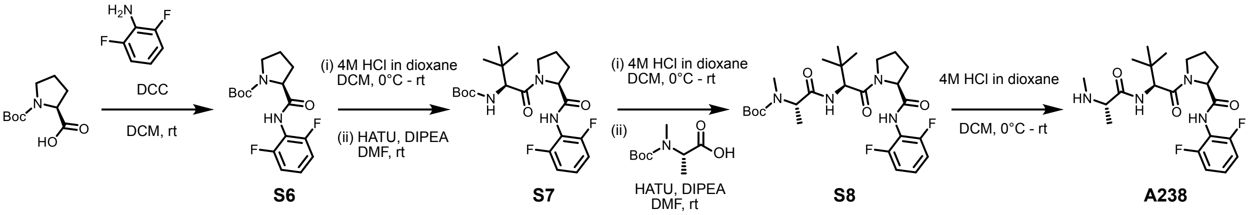

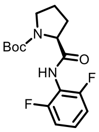

***tert*-butyl (2*S*)-2-[(2,6-difluorophenyl)carbamoyl]pyrrolidine-1-carboxylate (S6)**: To a solution of (2*S*)-1-*tert*-butoxycarbonylpyrrolidine-2-carboxylic acid (198 mg, 0.920 mmol, 1 eq,) and 2,6-difluoroaniline (104 µL, 0.97 mmol, 1.05 eq.) in DCM (8 mL) was added DCC (200 mg, 0.97 mmol, 1.05 eq.). A white precipitate formed - likely DCU. The mixture was stirred overnight at rt. LCMS showed presence of the product and the aniline SM. 0.5 eq. DCC was added to encourage conversion, however LCMS appeared the same after several hours. The mixture was filtered, and the filtrate was concentrated. The residue was separated by column chromatography (solid loading, 0-80% EtOAc in heptane) to give product as a white solid (210 mg, 70% yield) . The product contains a trace of DCU.

^{1}H NMR (300 MHz, $CDCl_3$) δ 9.07 (bs, 1H), 7.18 (bs, 1H), 6.94 (t, *J* = 8.3 Hz, 2H), 4.53 (bs, 1H), 3.49 (bs, 2H), 2.40 (bd, *J* = 87.2 Hz, 1H), 2.01 – 1.90 (m, 3H), 1.50 (s, 9H).

LCMS (Method 2) RT = 1.88 min, $[M+H-Boc]^+$ = 227.2, $[M-H]^-$ = 325.2

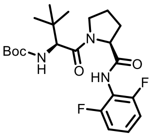

***tert*-butyl *N*-[(1*S*)-1-[(2*S*)-2-[(2,6-difluorophenyl)carbamoyl]pyrrolidine-1-carbonyl]-2,2-dimethyl-propyl]carbamate (S7)**: To a solution of *tert*-butyl (2*S*)-2-[(2,6-difluorophenyl)carbamoyl]pyrrolidine-1-carboxylate (507 mg, 1.55 mmol, 1 eq.) in DCM (12 mL) at 0 °C was added 4 M HCl in dioxane (3.88 mL, 15.5 mmol, 10 eq.) and the mixture was stirred overnight at rt. LCMS showed conversion to the desired product. The solvents were evaporated in vacuo and the residue was azeotroped twice with toluene to yield intermediate (2*S*)-*N*-(2,6-difluorophenyl)pyrrolidin-1-ium-2-carboxamide chloride as a white solid.

To a solution of (2*S*)-*N*-(2,6-difluorophenyl)pyrrolidin-1-ium-2-carboxamide chloride (100 mg, 0.34 mmol, 1 eq.), (*S*)-2-((*tert*-Butoxycarbonyl)amino)-3,3-dimethylbutanoic acid (83 mg, 0.36 mmol, 1.05 eq.) and HATU (195 mg, 0.514 mmol, 1.5 eq.) in DMF (3 mL) was added DIPEA (239 µL, 1.37 mmol, 4 eq.) and the mixture was stirred overnight at rt. LCMS showed formation of the desired product. The mixture was diluted in EtOAc and partitioned with sat. NH_4Cl. The aqueous layer was extracted (x3) and the combined

organics were washed with brine and dried over $MgSO_4$ and concentrated. The crude residue was purified by column chromatography using 0-100% gradient of EtOAc in heptane to give the product as a yellow solid (130 mg, 89% yield).

^{1}H NMR (300 MHz, $CDCl_3$) δ 8.90 (s, 1H), 7.25 – 7.09 (m, 1H), 6.92 (t, *J* = 7.9 Hz, 2H), 5.24 (d, *J* = 9.9 Hz, 1H), 4.89 (d, *J* = 6.3 Hz, 1H), 4.36 (d, *J* = 9.7 Hz, 1H), 3.82 (q, *J* = 9.2 Hz, 1H), 3.75 – 3.62 (m, 1H), 3.48 (s, 1H), 2.57 (s, 1H), 2.00 – 1.82 (m, 1H), 1.44 (s, 9H), 1.03 (s, 9H).

LCMS (Method 2) RT = 2.34 min, $[M+H]^+$ = 440.2, $[M-H]^-$ = 438.4

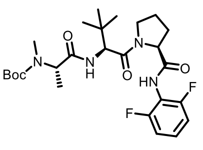

***tert*-butyl *N*-[(1*S*)-2-[[(1*S*)-1-[(2*S*)-2-[(2,6-difluorophenyl)carbamoyl]pyrrolidine-1-carbonyl]-2,2-dimethyl-propyl]amino]-1-methyl-2-oxo-ethyl]-*N*-methyl-carbamate (S8)**: To a solution of *tert*-butyl *N*-[(1*S*)-1-[(2*S*)-2-[(2,6-difluorophenyl)carbamoyl]pyrrolidine-1-carbonyl]-2,2-dimethyl-propyl]carbamate (130 mg, 0.27 mmol, 1 eq.) in DCM (3 mL) at 0 °C was added 4 M HCl in dioxane (686 µL, 2.74 mmol, 10 eq.) and the mixture was stirred at rt overnight. LCMS showed conversion to the desired amine. The mixture was dried *in vacuo* and azeotroped with toluene.

The crude amine, Boc-*N*-methyl-L-alanine (59 mg, 0.29 mmol, 1.1 eq.) and HATU (157 mg, 0.412 mmol, 1.4 eq.) were dissolved in DMF (2 mL). To the mixture was added DIPEA (191 µL, 1.10 mmol, 4 eq.) and stirred overnight at rt. LCMS showed formation of the desired product. The mixture was diluted in EtOAc and partitioned with sat. NH_4Cl. The aqueous layer was extracted (x3) and the combined organics were washed with brine and dried over $MgSO_4$ and concentrated. The crude residue was purified by column chromatography using 0-100% gradient of EtOAc in heptane to give product as a colourless solid (130 mg, 89% yield).

^{1}H NMR (300 MHz, $CDCl_3$) δ 8.92 (s, 1H), 7.22 – 7.06 (m, 1H), 7.04 – 6.73 (m, 3H), 4.85 (dd, *J* = 7.9, 2.4 Hz, 1H), 4.72 (bs, 1H), 4.64 (d, *J* = 9.3 Hz, 1H), 3.83 (td, *J* = 9.6, 7.0 Hz, 1H), 3.68 (ddd, *J* = 9.9, 8.0, 3.5 Hz, 1H), 2.78 (s, 3H), 2.52 (ddt, *J* = 12.5, 6.5, 2.8 Hz, 1H), 2.26 – 1.81 (m, 3H), 1.47 (s, 9H), 1.31 (d, *J* = 7.1 Hz, 3H), 0.99 (s, 9H).

LCMS (Method 2) RT = 2.36 min, $[M+H-Boc]^+$ = 425.4, $[M-H]^-$ = 523.4

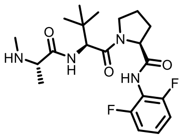

(2*S*)-*N*-(2,6-difluorophenyl)-1-[(2*S*)-3,3-dimethyl-2-[[(2*S*)-2-(methylamino)propanoyl]amino]butanoyl]pyrrolidine-2-carboxamide (A238): To a solution of *tert*-butyl *N*-[(1*S*)-2-[[(1*S*)-1-[(2*S*)-2-[(2,6-difluorophenyl)carbamoyl]pyrrolidine-1-carbonyl]-2,2-dimethyl-propyl]amino]-1-methyl-2-oxo-ethyl]-*N*-methyl-carbamate (89 mg, 0.17 mmol, 1 eq.) in DCM (2 mL) at 0

°C was added 4 M HCl in dioxane (424 µL, 1.70 mmol, 10 eq.) and the mixture was stirred overnight at rt. LCMS showed conversion to the desired product. The mixture was aspirated and subjected to acidic reverse phase HPLC. Product containing fractions were combined and dried in vacuo then further freeze dried to give product as a white solid (31 mg, 41% yield).

^{1}H NMR (300 MHz, MeOD) δ 7.31 (tt, *J* = 8.3, 6.1 Hz, 1H), 7.10 – 6.96 (m, 2H), 4.68 (dd, *J* = 8.1, 5.3 Hz, 1H), 4.64 (s, 1H), 3.96 (dt, *J* = 10.1, 6.4 Hz, 1H), 3.77 (dt, *J* = 10.0, 6.8 Hz, 1H), 3.47 (q, *J* = 6.9 Hz, 1H), 2.45 (s, 3H), 2.32 (ddd, *J* = 11.0, 8.1, 5.2 Hz, 1H), 2.22 – 1.94 (m, 3H), 1.34 (d, *J* = 6.8 Hz, 3H), 1.08 (s, 9H).

^{19}F NMR (282 MHz, MeOD) δ -119.62, -119.64, -119.64, -119.67.

^{13}C NMR (75 MHz, MeOD) δ 174.4, 173.2, 171.7, 171.6, 170.2, 161.3, 161.2, 158.0, 157.9, 129.4, 129.2, 129.1, 115.5, 115.2, 115.0, 112.9, 112.8, 112.8, 112.6, 112.6, 112.5, 62.5, 61.5, 59.6, 59.4, 59.1, 59.0, 49.8, 37.2, 36.2, 33.6, 33.3, 33.1, 30.9, 27.0, 26.9, 26.2, 23.1, 18.2, 17.7.

LCMS (Method 2) RT = 1.13 min, $[M+H]^+$ = 425.4

HRMS (ESI+) *m/z*: $[M+H]^+$ calcd for $C_{28}H_{26}F_3N_4O_3$ 523.1957; found 523.1937.

Synthesis of ***A273***

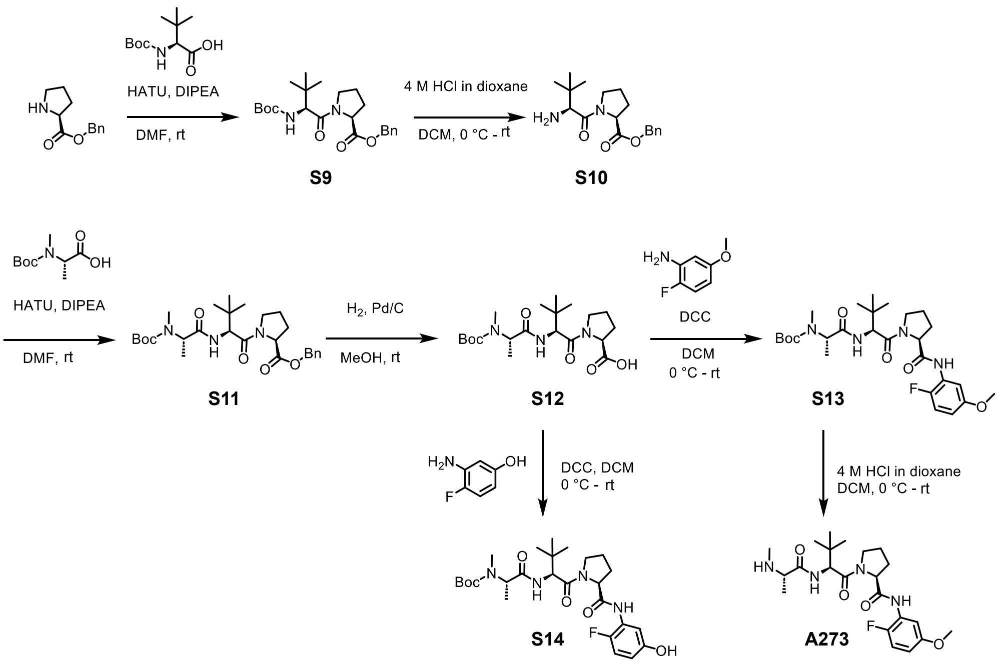

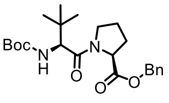

benzyl (2*S*)-1-[(2*S*)-2-(*tert*-butoxycarbonylamino)-3,3-dimethyl-butanoyl]pyrrolidine-2-carboxylate (S9): To a solution of benzyl (2*S*)-pyrrolidin-1-ium-2-carboxylate chloride (3000 mg, 12 mmol, 1 eq.), (*S*)-2-((*tert*-Butoxycarbonyl)amino)-3,3-dimethylbutanoic acid (3000 mg, 13 mmol, 1.05 eq.) and HATU (5680 mg, 14.9 mmol, 1.2 eq.) in DMF (50 mL) was added DIPEA (8680 µL, 49.8 mmol, 4 eq.) and the

mixture was stirred overnight at rt. The mixture was diluted in EtOAc and partitioned with sat. NH_4Cl. The aqueous layer was extracted (x3) and the combined organics were washed with brine and dried over $MgSO_4$ and concentrated. The crude residue was separated by column chromatography (Combi-Flash) eluting with 0-50% EtOAc in heptane to obtain the title product as a yellow oil (4700 mg, 81% yield).

^1H NMR (300 MHz, $CDCl_3$) δ 7.36 – 7.31 (m, 5H), 5.36 – 5.23 (m, 1H), 5.23 – 5.08 (m, 2H), 4.68 – 4.52 (m, 1H), 4.29 (d, *J* = 9.9 Hz, 1H), 3.81 (dd, *J* = 10.1, 6.0 Hz, 1H), 3.67 (dt, *J* = 9.9, 6.8 Hz, 1H), 2.23 (ddd, *J* = 10.9, 8.0, 6.2 Hz, 1H), 1.98 (h, *J* = 6.9 Hz, 3H), 1.42 (s, 9H), 0.99 (s, 9H).

LCMS (Method 2) RT = 2.65 min, $[M+H]^+$ = 419.4

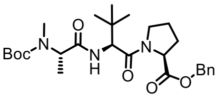

benzyl (2*S*)-1-[(2*S*)-2-[[(2*S*)-2-[*tert*-butoxycarbonyl(methyl)amino]propanoyl]amino]-3,3-dimethyl-butanoyl]pyrrolidine-2-carboxylate (S11): To a solution of benzyl (2*S*)-1-[(2*S*)-2-(*tert*-butoxycarbonylamino)-3,3-dimethyl-butanoyl]pyrrolidine-2-carboxylate (4200 mg, 10 mmol, 1 eq.) in DCM (40 mL) at 0 °C was added 4 M HCl in dioxane (15.2 mL, 60.8 mmol, 6 eq.) and the mixture was stirred at rt overnight. The mixture was dried *in vacuo* and azeotroped with toluene. The crude amine **S10**, Boc-*N*-methyl-L-alanine (2200 mg, 11 mmol, 1.05 eq.) and HATU (4630 mg, 12.2 mmol, 1.2 eq.) were dissolved in DMF (30 mL) at 0°C. To the mixture was added DIPEA (7060 µL, 40.6 mmol, 4 eq.) and it was stirred overnight at rt. The mixture was diluted in EtOAc and partitioned with sat. NH_4Cl. The aqueous layer was extracted (x3) and the combined organics were washed with brine and dried over $MgSO_4$ and concentrated. The crude residue was separated using column chromatography (Combi-Flash) eluting with 0-80% EtOAc in heptane to yield the title product as a yellow oil (4600 mg, 90% yield).

^1H NMR (300 MHz, $CDCl_3$) δ 7.38 – 7.30 (m, 5H), 5.16 (q, *J* = 12.3 Hz, 2H), 4.75 – 4.51 (m, 3H), 3.85 (dt, *J* = 9.9, 6.5 Hz, 1H), 3.74 – 3.61 (m, 1H), 2.78 (s, 3H), 2.30 – 2.11 (m, 1H), 2.08 – 1.87 (m, 3H), 1.60 (s, 1H), 1.48 (s, 9H), 1.31 (d, *J* = 7.1 Hz, 3H), 0.98 (s, 9H).

LCMS (Method 2) RT = 2.70 min, $[M+H]^+$ = 504.4

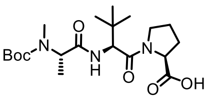

(2*S*)-1-[(2*S*)-2-[[(2*S*)-2-[*tert*-butoxycarbonyl(methyl)amino]propanoyl]amino]-3,3-dimethyl-butanoyl]pyrrolidine-2-carboxylic acid (S12): A solution of benzyl (2*S*)-1-[(2*S*)-2-[[(2*S*)-2-[*tert*-butoxycarbonyl(methyl)amino]propanoyl]amino]-3,3-dimethyl-butanoyl]pyrrolidine-2-carboxylate (460 mg, 0.913 mmol, 1 eq.) in MeOH (8 mL) was prepared. The mixture was degassed before 10% Pd/C (9.1 mg, 0.091 mmol, 0.1 eq.) was added. The mixture was degassed again before the introduction of a H_2 balloon. The mixture was stirred overnight at rt, after which LCMS showed formation of the desired product. The mixture was filtered over Celite and dried in vacuo to obtain the title product as an off-white solid (370 mg, 97% yield).

^{1}H NMR (300 MHz, $CDCl_3$) δ 6.88 (s, 1H), 4.76 – 4.48 (m, 4H), 3.94 – 3.74 (m, 1H), 3.75 – 3.61 (m, 1H), 3.49 (s, 1H), 2.78 (s, 3H), 2.39 – 2.20 (m, 1H), 2.05 (m, 3H), 1.48 (s, 9H), 1.32 (d, *J* = 7.1 Hz, 3H), 1.00 (s, 9H).

LCMS (Method 2) RT = 1.85 min, $[M+H]^+$ = 414.4

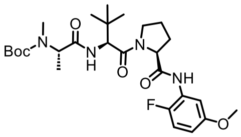

***tert*-butyl *N*-[(1*S*)-2-[[(1*S*)-1-[(2*S*)-2-[(2-fluoro-5-methoxyphenyl)carbamoyl]pyrrolidine-1-carbonyl]-2,2-dimethyl-propyl]amino]-1-methyl-2-oxo-ethyl]-*N*-methyl-carbamate (S13):** To a suspension of (2*S*)-1-[(2*S*)-2-[[(2*S*)-2-[tert-butoxycarbonyl(methyl)amino]propanoyl]amino]-3,3-dimethyl-butanoyl]pyrrolidine-2-carboxylic acid (40 mg, 0.097 mmol, 1 eq.) in DCM (1 mL) and 2-fluoro-5-methoxy-aniline (13 µL, 0.11 mmol, 1.1 eq.) at RT was added DCC (22.0 mg, 0.11 mmol, 1.1 eq.). The mixture was stirred overnight at RT. The mixture was filtered and subjected to column chromatography using a 0-5% gradient of MeOH in DCM to yield *tert*-butyl *N*-[(1*S*)-2-[[(1*S*)-1-[(2*S*)-2-[(4-methoxyphenyl)carbamoyl]pyrrolidine-1-carbonyl]-2,2-dimethyl-propyl]amino]-1-methyl-2-oxo-ethyl]-*N*-methylcarbamate (48 mg, 75% pure, 69% yield) as a white solid. The product contained acid starting material and dicyclohexylurea, but was taken forward without further purification.

^{1}H NMR (300 MHz, $CDCl_3$) δ ^{1}H NMR (300 MHz, $CDCl_3$) δ 9.46 (s, 1H), 7.97 (dd, *J* = 6.4, 3.1 Hz, 1H), 6.98 (dd, *J* = 10.3, 9.0 Hz, 1H), 6.55 (dt, *J* = 9.0, 3.5 Hz, 1H), 4.86 (dd, *J* = 8.0, 2.2 Hz, 1H), 4.61 (m, 1H), 3.97 – 3.78 (m, 1H), 3.79 (s, 3H), 3.77 – 3.61 (m, 1H), 2.80 (s, 3H), 2.65 – 2.51 (m, 1H), 2.23 – 2.02 (m, 2H), 2.01 – 1.79 (m, 2H), 1.52 (s, 9H), 1.34 (d, *J* = 7.1 Hz, 3H), 1.00 (s, 9H).

LCMS (Method 2) RT = 2.53 min, $[M+H]^+$ = 537.4

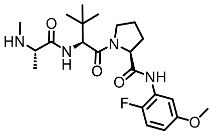

(2*S*)-1-[(2*S*)-3,3-dimethyl-2-[[(2*S*)-2-(methylamino)propanoyl]amino]butanoyl]-*N*-(2-fluoro-5-methoxyphenyl)pyrrolidine-2-carboxamide (A273): To a solution of *tert*-butyl *N*-[(1*S*)-2-[[(1*S*)-1-[(2*S*)-2-[(2-fluoro-5-methoxy-phenyl)carbamoyl]pyrrolidine-1-carbonyl]-2,2-dimethyl-propyl]amino]-1-methyl-2-oxoethyl]-*N*-methyl-carbamate (48.0 mg, 75% pure, 0.0671 mmol, 1 eq.) in DCM (1 mL) at 0 °C was added 4 M HCl in 1,4-dioxane (170 µL, 0.67 mmol, 10 eq.) and the mixture was stirred at RT overnight. The solution was aspirated and the resultant solid was subjected to SCX chromatography. The ammonia fraction was dried in vacuo and subjected to HPLC. Product fractions were dried in speedvac and further freeze dried to yield (2*S*)-1-[(2*S*)-3,3-dimethyl-2-[[(2*S*)-2-(methylamino)propanoyl]amino]butanoyl]-*N*-(2-fluoro-5-methoxy-phenyl)pyrrolidine-2-carboxamide (14 mg, 44% yield) as a white solid.

^{1}H NMR (300 MHz, DMSO-d_6) δ 9.84 (s, 1H), 8.25 (s, 1H), 7.97 (d, *J* = 9.2 Hz, 1H), 7.60 (dd, *J* = 6.6, 3.1 Hz, 1H), 7.17 (dd, *J* = 10.4, 7.8 Hz, 1H), 6.68 (dt, *J* = 9.0, 3.4 Hz, 1H), 4.65 (m, 1H), 4.52 (d, *J* = 9.2 Hz, 1H), 4.01 (bs, 1H), 3.83 – 3.64 (m, 2H), 3.72 (s, 3H), 3.11 (m, 1H), 2.23 (s, 3H), 2.17-2.06 (m, 1H), 2.05 – 1.79 (m, 2H), 1.15 (d, *J* = 7.1 Hz, 3H), 0.98 (s, 9H).

^{19}F NMR (282 MHz, DMSO-d_6) δ -136.18, -136.66

^{13}C NMR (75 MHz, DMSO-d_6) δ 173.8, 171.2, 169.9, 164.5, 155.7, 127.2, 116.1, 115.9, 109.2, 60.4, 59.2, 56.6, 56.0, 48.5, 40.8, 40.5, 40.3, 40.0, 39.7, 39.4, 39.1, 35.5, 34.2, 29.5, 26.7, 25.3, 19.0.

LCMS (Method 2) RT = 1.32 min, $[M+H]^+$ = 437.4

*Synthesis of **L118** (low affinity IAP ligand for cIAP1 crystallization)*

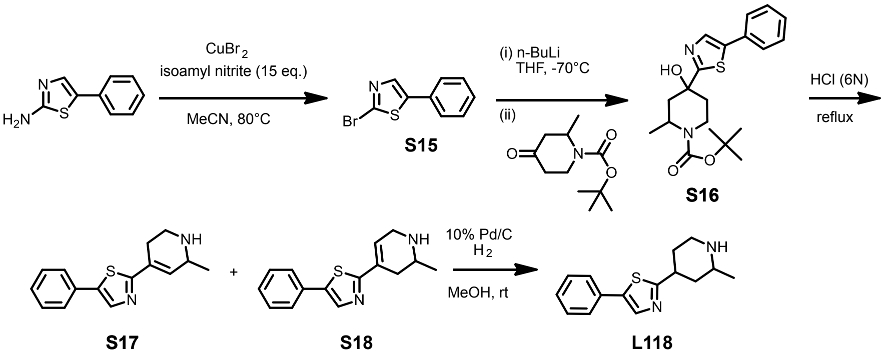

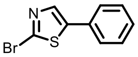

2-bromo-5-phenylthiazole (S15): Copper(II) bromide (1.88 g, 8.52 mmol, 3.0 eq.) was added to a mixture of 5-phenylthiazol-2-amine (0.50 g, 2.84 mmol) and isoamyl nitrite (4.99 g, 42.6 mmol, 15 eq.) in acetonitrile (10 mL). The resulting mixture was stirred at 80°C for 4 h. It was extracted with DCM, washed with brine, dried over sodium sulfate and concentrated to obtain crude 2-bromo-5-phenylthiazole (0.40 g, 58% yield), which was used in the next step without further purification.

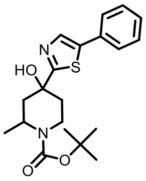

***tert*-butyl 4-hydroxy-2-methyl-4-(5-phenylthiazol-2-yl)piperidine-1-carboxylate (S16):** n-Butyl lithium (2.5 M in hexanes, 1.08 mL, 2.69 mmol, 1.6 eq.) was added dropwise to a solution of 2-bromo-5-phenylthiazole (**S15**) (0.40 g, 1.67 mmol) in dry THF (4.3 mL) at -70°C. Then a solution of ethyl 2-methyl-4-oxopiperidine-1-carboxylate in dry THF was added and stirring was continued for 2 h. The mixture was partitioned between water and EtOAc. The organic layer was washed with brine, dried over sodium

sulfate and concentrated to give crude *tert*-butyl 4-hydroxy-2-methyl-4-(5-phenylthiazol-2-yl)piperidine-1-carboxylate (0.25 g, 40% yield) as an oil, which was used in the next step without further purification.

LCMS RT = 0.90 min, $[M+H]^+$ = 375.2

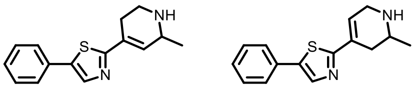

2-(6-methyl-1,2,3,6-tetrahydropyridin-4-yl)-5-phenylthiazole (S17) and **2-(2-methyl-1,2,3,6-tetrahydropyridin-4-yl)-5-phenylthiazole (S18):** A solution of *tert*-butyl 4-hydroxy-2-methyl-4-(5-phenylthiazol-2-yl)piperidine-1-carboxylate (**S16**) (0.30 g, 0.80 mmol) in hydrochloric acid (6 N, 30 mL) was stirred at reflux for 3 days. The solvent was removed under reduced pressure and the resulting crude mixture of 2-(6-methyl-1,2,3,6-tetrahydropyridin-4-yl)-5-phenylthiazole and 2-(2-methyl-1,2,3,6-tetrahydropyridin-4-yl)-5-phenylthiazole (0.04 g, 20% yield) as an oil was forwarded to the next step without further purification.

LCMS RT = 1.2, 1.3 min, $[M+H]^+$ = 275.2, 275.2

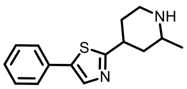

2-(2-methylpiperidin-4-yl)-5-phenylthiazole (L118): A mixture of 2-(6-methyl-1,2,3,6-tetrahydropyridin-4-yl)-5-phenylthiazole (**S17**) and 2-(2-methyl-1,2,3,6-tetrahydropyridin-4-yl)-5-phenylthiazole (**S18**) (0.04 g, 0.16 mmol) was dissolved in methanol (50 mL) and 10% Pd/C (50 mg) was added. The mixture was hydrogenated for 2 h under 20 psi of hydrogen at room temperature. The catalyst was filtered off, and the filtrate was concentrated. The residue was dissolved in EtOAc, washed with aqueous sodium bicarbonate solution and brine, dried over sodium sulfate and concentrated. The crude product was purified by prep. HPLC to give 2-(2-methylpiperidin-4-yl)-5-phenylthiazole (0.03 g, 74%).

^{1}H NMR (400 MHz, MeOD) δ 7.99 (s, 1H), 7.63 (dt, *J* = 8.4, 2.3 Hz, 2H), 7.49 – 7.41 (m, 2H), 7.41 – 7.33 (m, 1H), 3.53 (ddd, *J* = 12.8, 4.3, 2.2 Hz, 1H), 3.49 – 3.34 (m, 3H), 3.19 (td, *J* = 13.2, 3.0 Hz, 1H), 2.38 (t, *J* = 14.6 Hz, 2H), 1.96 (qd, *J* = 13.5, 4.2 Hz, 1H), 1.84 – 1.70 (m, 1H), 1.40 (d, *J* = 6.5 Hz, 3H).

LCMS RT = 2.45 min, $[M+H]^+$ = 259.1

Synthesis of TEAD Binders

*Synthesis of **A262***

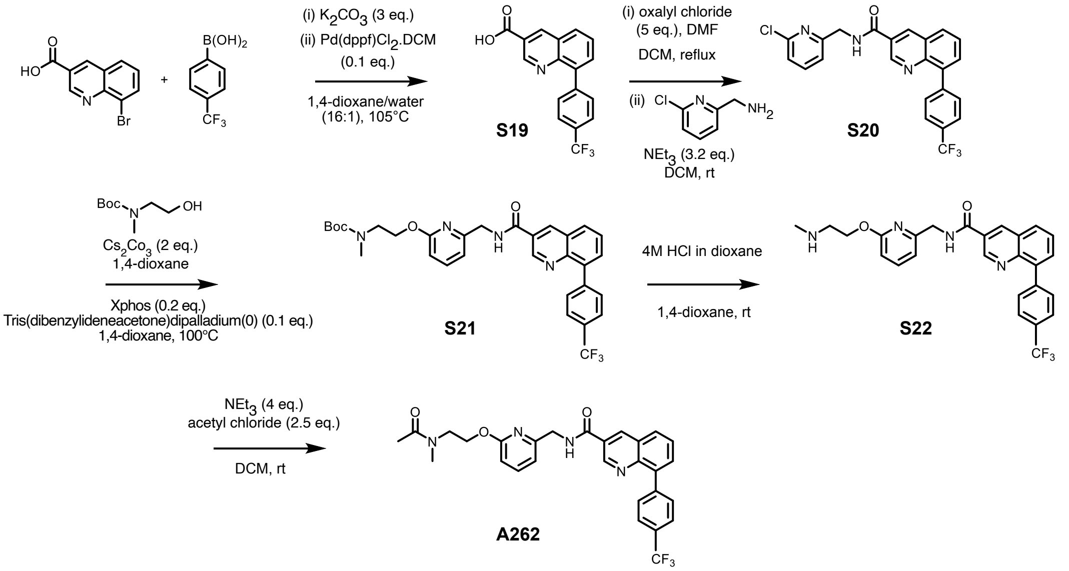

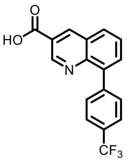

8-[4-(trifluoromethyl)phenyl]quinoline-3-carboxylic acid (S19): 8-bromoquinoline-3-carboxylic acid (3000 mg, 11.9 mmol, 1 eq.), [4-(trifluoromethyl)phenyl]boronic acid (2500 mg, 13 mmol, 1.1 eq.) and K_2CO_3 (6370 mg, 35.7 mmol, 3 eq.) were taken up in 1,4-dioxane (80 mL) and Water (5 mL) and the mixture was degassed with a stream of bubbling nitrogen for 45 min. Pd(dppf)Cl_2.DCM (970 mg, 1.2 mmol, 0.1 eq.) was added and the reaction mixture was degassed for a further 15 min followed by heating at 105 °C. Once the reaction was complete (as monitored by LCMS), the mixture was cooled, concentrated, diluted with EtOAc and water and filtered through a bed of Celite. The filtrate was transferred to a separating funnel and diluted further with brine. The organic layer was collected, dried ($MgSO_4$) then concentrated to a crude solid. The crude material was purified by Combi-Flash eluting with MeOH:DCM (0-5%) with ca. 1% AcOH to afford the title product as a brown solid (1150 mg, 41% yield).

^{1}H NMR (300 MHz, $CDCl_3$) δ 9.54 (s, 1H), 9.00 (s, 1H), 8.05 (d, *J* = 8.0 Hz, 1H), 7.90 (d, *J* = 7.0 Hz, 1H), 7.77 (dt, *J* = 15.1, 6.3 Hz, 5H).

LCMS (Method 2) RT = 2.57 min, $[M+H]^+$ = 318.2

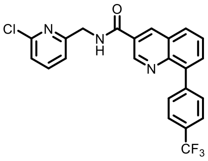

***N*-[(6-chloro-2-pyridyl)methyl]-8-[4-(trifluoromethyl)phenyl]quinoline-3-carboxamide (S20):** 8-[4-(trifluoromethyl)phenyl]quinoline-3-carboxylic acid (**S19**) (3000 mg, 9.46 mmol, 1 eq.) was dissolved in DCM (10 mL) then treated with oxalyl chloride (4.1 mL, 47 mmol, 5 eq.) and a drop of DMF. The bubbling solution stirred for 5 min at rt and then heated to reflux for 30 min. The solution was concentrated and dried further under high vac. The residue was re-dissolved in DCM (10 mL) then treated with a solution of (6-chloro-2-pyridyl)methanamine dihydrochloride (2240 mg, 10.4 mmol, 1.1 eq.) and triethylamine (4.2 mL, 30 mmol, 3.2 eq.) in DCM (10 mL). Once the reaction was complete (monitored by LCMS) the reaction was diluted with DCM and washed with sat. $NaHCO_3$, 10% citric acid solution followed by brine. The organic layer was dried ($MgSO_4$) then concentrated to yield the crude product as a brown foam. The crude material was purified on Combi-Flash eluting with EtOAc/heptane (0-30%) to afford the title product as an off-white solid (2600 mg, 62% yield).

^{1}H NMR (300 MHz, $CDCl_3$) δ 9.36 (d, *J* = 2.3 Hz, 1H), 8.75 (d, *J* = 2.3 Hz, 1H), 7.99 (dd, *J* = 8.1, 1.5 Hz, 1H), 7.86 – 7.64 (m, 7H), 7.60 (s, 1H), 7.30 (t, *J* = 8.5 Hz, 2H), 4.81 (d, *J* = 5.0 Hz, 2H).

LCMS (Method 2) RT = 2.57 min, $[M+H]^+$ = 318.2

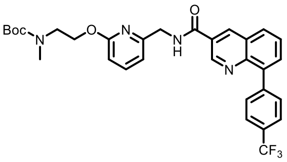

***tert*-butyl *N*-methyl-*N*-[2-[[6-[[[8-[4-(trifluoromethyl)phenyl]quinoline-3-carbonyl]amino]methyl]-2-pyridyl]oxy]ethyl]carbamate (S21):** *N*-[(6-chloro-2-pyridyl)methyl]-8-[4-(trifluoromethyl)phenyl]quinoline-3-carboxamide (**S20**) (537 mg, 1.22 mmol, 1 eq.), tert-butyl *N*-(2-hydroxyethyl)-*N*-methyl-carbamate (256 mg, 1.46 mmol, 1.2 eq.) and Cesium Carbonate (816 mg, 2.43 mmol, 2 eq.) were taken up in 1,4-Dioxane (12 mL) and degassed with a stream of bubbling nitrogen for 30 min. Xphos (116 mg, 0.24 mmol, 0.2 eq.) and Tris(dibenzylideneacetone)dipalladium(0) (110 mg, 0.12 mmol, 0.1 eq.) were added and the reaction mixture was degassed for a further 10 min before being heated to 100 °C. After 1.5 h, LCMS showed full conversion to the product and the reaction was removed from the heat, partitioned between water and EtOAc and stood overnight. The organic layer was collected and the aqueous layer extracted twice with EtOAc. The combined organic layers were dried over $MgSO_4$ then concentrated under reduced pressure to a brown residue. The crude material was purified by column chromatography (Combi-Flash) eluting with EtOAc:n-Hept (0-60%) to yield the title product as a yellow oil (480 mg, 61%), with some starting material contaminant.

^{1}H NMR (300 MHz, $CDCl_3$) δ 9.39 (s, 1H), 8.82 (d, *J* = 22.2 Hz, 1H), 7.98 (d, *J* = 8.1 Hz, 1H), 7.90 – 7.65 (m, 7H), 7.59 (t, *J* = 7.7 Hz, 1H), 6.97 (s, 1H), 6.67 (d, *J* = 8.2 Hz, 1H), 4.73 (s, 2H), 4.50 (t, *J* = 5.5 Hz, 2H), 3.62 (s, 2H), 2.89 (s, 3H), 1.36 (s, 9H).

LCMS (Method 2) RT = 3.07 min, $[M+H]^+$ = 581.4

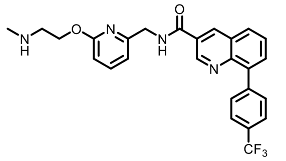

***N*-[[6-[2-(methylamino)ethoxy]-2-pyridyl]methyl]-8-[4-(trifluoromethyl)phenyl]quinoline-3-carboxamide (S22)**: *tert*-butyl *N*-methyl-*N*-[2-[[6-[[[8-[4-(trifluoromethyl)phenyl]quinoline-3-carbonyl]amino]methyl]-2-pyridyl]oxy]ethyl]carbamate (**S21**) (150 mg, 0.23 mmol, 1 eq.) was dissolved in 1,4-Dioxane (2 mL) then treated with 4M HCl in dioxane (0.29 mL, 1.2 mmol, 5 eq.). The reaction formed a suspension and stirred at rt for 16 h. LCMS showed full conversion to the product. The reaction was concentrated under reduced pressure and the solid was partitioned between EtOAc and sat. $NaHCO_3$. The organic layer was collected, and the aqueous layer extracted twice with EtOAc. The combined organic layers were dried over $MgSO_4$ and concentrated under reduced pressure to yield the title compound as a sticky yellow solid (140 mg, 97%).

^{1}H NMR (300 MHz, $CDCl_3$) δ 9.29 (d, *J* = 2.3 Hz, 1H), 8.68 (d, *J* = 2.3 Hz, 1H), 7.96 (dd, *J* = 8.1, 1.5 Hz, 1H), 7.85 – 7.63 (m, 6H), 7.27 (t, *J* = 5.8 Hz, 1H), 7.00 – 6.92 (m, 2H), 6.86 (d, *J* = 8.3 Hz, 1H), 6.64 (s, 1H), 4.69 (d, *J* = 5.6 Hz, 2H), 4.07 (t, *J* = 5.1 Hz, 2H), 2.96 (t, *J* = 5.1 Hz, 2H), 2.49 (s, 3H).

LCMS (Method 2) RT = 1.93 min, $[M+H]^+$ = 480.0

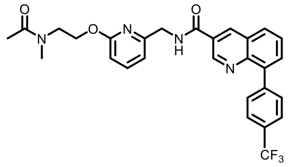

***N*-[[6-[2-[acetyl(methyl)amino]ethoxy]-2-pyridyl]methyl]-8-[4-(trifluoromethyl)phenyl]quinoline-3-carboxamide (A262):** *N*-[[6-[2-(methylamino)ethoxy]-2-pyridyl]methyl]-8-[4-(trifluoromethyl)phenyl]quinoline-3-carboxamide (**S22**) (80 mg, 0.11 mmol, 1 eq.) and triethylamine ((60 µL, 0.43 mmol, 4 eq.) were dissolved in DCM (1 mL) and treated with acetyl chloride (19.3 µL, 0.271 mmol, 2.5 eq.). Once the reaction was complete (monitored by LCMS), the mixture was diluted with DCM and partitioned with water. The aqueous layer was extracted (x3) and the combined organics were washed with brine, dried over $MgSO_4$ and concentrated. The residue was separated using column chromatography (Combi-Flash) with a gradient of 0-10% MeOH in DCM to yield the title compound as a white solid (35 mg, 61% yield). NMR in $CDCl_3$ shows evidence of rotamers, LCMS shows one peak.

^{1}H NMR (300 MHz, $CDCl_3$) δ 9.38 (t, *J* = 2.2 Hz, 1H), 8.84 (dd, *J* = 7.6, 2.3 Hz, 1H), 8.10 – 7.91 (m, 2H), 7.90 – 7.69 (m, 6H), 7.67 (t, *J* = 7.6 Hz, 1H), 7.56 (ddd, *J* = 8.4, 7.2, 1.3 Hz, 1H), 6.94 (dd, *J* = 16.9, 7.2 Hz, 1H),

6.63 (d, *J* = 8.2 Hz, 1H), 4.71 (dd, *J* = 10.5, 5.1 Hz, 2H), 4.57 – 4.46 (m, 2H), 3.70 (dt, *J* = 14.0, 5.6 Hz, 2H), 3.05 (s, 2H), 2.93 (s, 1H), 2.09 (s, 1H), 1.97 (s, 2H).

^{19}F NMR (282 MHz, $CDCl_3$) δ -62.40, -62.42.

^{13}C NMR (75 MHz, $CDCl_3$) δ 171.5, 171.2, 165.7, 165.4, 163.2, 163.0, 154.4, 153.9, 148.8, 148.5, 146.8, 146.7, 142.9, 142.8, 140.0, 139.7, 139.5, 139.5, 136.6, 136.4, 132.1, 132.0, 131.1, 131.0, 129.9, 129.8, 129.5, 129.5, 129.4, 129.4, 127.7, 127.3, 127.2, 127.2, 127.1, 126.3, 125.1, 125.1, 125.1, 125.0, 125.0, 125.0, 115.8, 115.0, 109.9, 109.5, 63.3, 62.8, 50.7, 47.4, 45.3, 44.7, 38.0, 34.3, 21.9, 21.5.

LCMS (Method 2) RT = 2.57 min, $[M+H]^+$ = 523.2

HRMS (ESI+) *m/z*: $[M+H]^+$ calcd for $C_{28}H_{26}F_3N_4O_3$ 523.1957; found 523.1937.

*Synthesis of **A341***

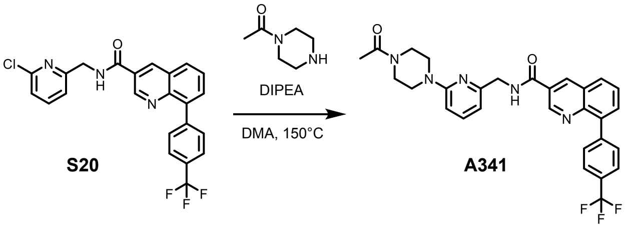

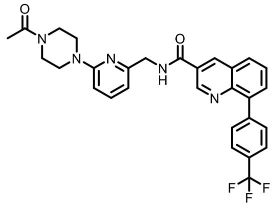

***N*-[[6-(4-acetylpiperazin-1-yl)-2-pyridyl]methyl]-8-[4-(trifluoromethyl)phenyl]quinoline-3-carboxamide (A341):** *N*-[(6-chloro-2-pyridyl)methyl]-8-[4-(trifluoromethyl)phenyl]quinoline-3-carboxamide (**S20**) (40 mg, 0.091 mmol, 1 eq.) was dissolved in DMA (1 mL) in a screw capped vial then treated with DIPEA (78.8 µL, 0.45 mmol, 5 eq.) and 1-acetalpiperazine (58.0 mg, 0.45 mmol, 5 eq.). The vial was sealed and heated for 16 h at 150 °C. LCMS showed approximately 40% conversion to the product. An additional 100 mg of 1-acetylpiperazine was added and the reaction was stirred at 150 °C. After 8 h, more conversion was observed, but there was still significant unreacted starting material observed. An additional 100 mg of 1-acetylpiperazine was added and the reaction stirred for 16 h at 150 °C. The reaction was cooled to rt then partitioned between EtOAc and chilled brine. The organic phase was washed with chilled brine (x4), dried ($MgSO_4$) then concentrated under reduced pressure to a yellow oil. The crude product was purified by Combi-Flash (Solvent A: DCM, B: DCM:MeOH:NH_4OH (aq.) 5:4:1) to obtain the title compound as a yellow oil which was lyophilized to a white solid (29 mg, 48% yield).

^{1}H NMR (300 MHz, $CDCl_3$) δ 9.32 (d, *J* = 2.3 Hz, 1H), 8.77 (d, *J* = 2.3 Hz, 1H), 7.97 (dd, *J* = 8.2, 1.6 Hz, 1H), 7.86 – 7.61 (m, 8H), 7.53 (dd, *J* = 8.5, 7.3 Hz, 1H), 6.71 (d, *J* = 7.3 Hz, 1H), 6.59 (d, *J* = 8.4 Hz, 1H), 4.71 (d, *J* = 4.7 Hz, 2H), 3.85 – 3.72 (m, 2H), 3.70 – 3.57 (m, 4H), 3.56 – 3.48 (m, 2H), 2.13 (s, 3H).

^{19}F NMR (282 MHz, $CDCl_3$) δ -62.43.

^{13}C NMR (75 MHz, $CDCl_3$) δ 169.4, 165.2, 158.7, 154.0, 147.9, 146.8, 142.8, 142.7, 139.6, 138.9, 136.6, 132.2, 131.0, 129.5, 129.4 (q, *J* = 32.8 Hz), 127.7, 127.5, 127.3, 126.2 (q, *J* = 272.1 Hz), 125.1 (q, *J* = 3.8 Hz), 111.9, 106.1, 46.0, 45.3, 45.2, 44.9, 41.1, 21.5.

LCMS (Method 2) RT = 2.15 min, $[M+H]^+$ = 534.4

HRMS (ESI+) *m/z*: $[M+H]^+$ calcd for $C_{29}H_{27}F_3N_5O_2$ 534.2117; found 534.2098.

Synthesis of TEAD IPDs and negative controls

Synthesis of ***A531***

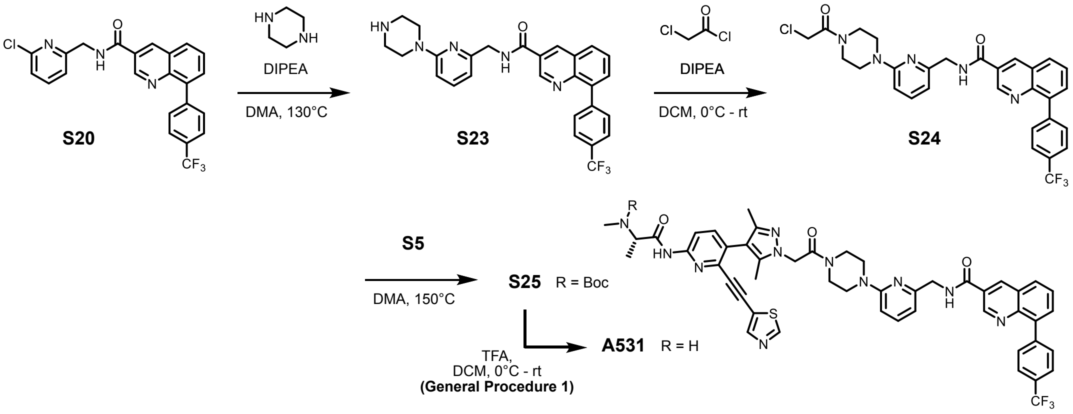

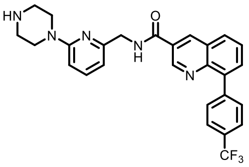

***N*-[(6-piperazin-1-yl-2-pyridyl)methyl]-8-[4-(trifluoromethyl)phenyl]quinoline-3-carboxamide (S23):** *N*-[(6-chloro-2-pyridyl)methyl]-8-[4-(trifluoromethyl)phenyl]quinoline-3-carboxamide (50 mg, 0.11 mmol, 1 eq.) was dissolved in DMA (1 mL) in a 4 mL screw capped vial then treated with DIPEA (19.7 µL, 0.113 mmol.) and piperazine (48.7 mg, 0.57 mmol, 5 eq.). The vial was sealed then heated for 16 h at 130 °C. After the reaction had stopped progressing (as monitored by LCMS) the mixture was cooled to rt then partitioned between EtOAc and sat. $NaHCO_3$. The organic phase was collected then washed with chilled brine (x4), dried ($MgSO_4$) then concentrated under reduced pressure. The crude was purified by Combi-Flash on a 4 g silica column eluting with A: DCM and B: MeOH:NH_4OH (aq.) (9:1) to obtain the title product as white solid (38 mg, 62% yield).

^{1}H NMR (300 MHz, $CDCl_3$) δ 9.33 (d, *J* = 2.3 Hz, 1H), 8.75 (d, *J* = 2.3 Hz, 1H), 7.95 (dd, *J* = 8.2, 1.5 Hz, 1H), 7.87 – 7.60 (m, 7H), 7.49 (dd, *J* = 8.5, 7.3 Hz, 1H), 6.67 (d, *J* = 7.3 Hz, 1H), 6.57 (d, *J* = 8.5 Hz, 1H), 4.69 (d, *J* = 4.6 Hz, 2H), 3.69 – 3.47 (m, 5H), 3.11 – 2.96 (m, 6H).

LCMS (Method 2) RT = 1.94 min, $[M+H]^+$ = 492.2

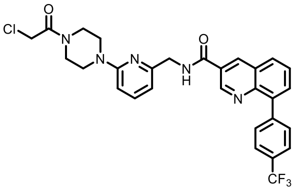

N-[[6-[4-(2-chloroacetyl)piperazin-1-yl]-2-pyridyl]methyl]-8-[4-(trifluoromethyl)phenyl]quinoline-3-carboxamide (S24): *N*-[(6-piperazin-1-yl-2-pyridyl)methyl]-8-[4-(trifluoromethyl)phenyl]quinoline-3-carboxamide (**S23**) (500 mg, 1.0 mmol, 1 eq.) was dissolved in DCM (10 mL) and cooled to 0°C. 2-chloroacetyl chloride (89 µL, 1.1 mmol. 1.1 eq.) in 1 mL DCM was added, followed by DIPEA (190 µL, 1.1 mmol, 1.1 eq.). The mixture was left to stir at rt overnight. Once the reaction was complete (monitored by LCMS), the mixture was diluted with DCM and partitioned with water. The aqueous layer was extracted (x3) and the combined organic layers were dried over $MgSO_4$ and concentrated. The residue was separated using column chromatography with a gradient of 0-100% EtOAc in heptane to yield the title compound as an off-white solid (430 mg, 75% yield).

^{1}H NMR (300 MHz, $CDCl_3$) δ 9.32 (d, J = 2.3 Hz, 1H), 8.77 (d, J = 2.3 Hz, 1H), 7.99 (dd, J = 8.1, 1.5 Hz, 1H), 7.88 – 7.78 (m, 3H), 7.81 – 7.66 (m, 3H), 7.55 (dd, J = 8.5, 7.3 Hz, 1H), 7.50 (s, 1H), 6.73 (d, J = 7.3 Hz, 1H), 6.61 (d, J = 8.5 Hz, 1H), 4.72 (d, J = 4.7 Hz, 2H), 4.11 (s, 2H), 3.82 – 3.54 (m, 8H).

LCMS (Method 2) RT = 2.35 min, $[M+H]^+$ = 568.2

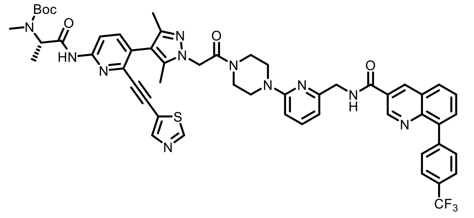

***tert*-butyl *N*-[(1*S*)-2-[[5-[3,5-dimethyl-1-[2-oxo-2-[4-[6-[[[8-[4-(trifluoromethyl)phenyl]quinoline-3-carbonyl]amino]methyl]-2-pyridyl]piperazin-1-yl]ethyl]pyrazol-4-yl]-6-(2-thiazol-5-ylethynyl)-2-pyridyl]amino]-1-methyl-2-oxo-ethyl]-*N*-methyl-carbamate (S25):** *N*-[[6-[4-(2-chloroacetyl)piperazin-1-yl]-2-pyridyl]methyl]-8-[4-(trifluoromethyl)phenyl]quinoline-3-carboxamide (**S24**) (40.0 mg, 0.070 mmol, 1 eq.), *tert*-butyl ((2*S*)-1-((5-(3,5-dimethyl-1*H*-pyrazol-4-yl)-6-(thiazol-5-ylethynyl)pyridin-2-yl)amino)-1-oxopropan-2-yl)(methyl)carbamate (**S5**) (33.8 mg, 0.070 mmol, 1 eq.) and Cs_2CO_3 (69 mg, 0.21 mmol, 3 eq.) were dissolved in acetonitrile (1.5 mL) and stirred at rt overnight. LCMS shows 50% completion, the reaction mixture was then heated at 60 °C for 2 h, at which point the LCMS showed reaction progressed to completion. The reaction mixture was diluted with DCM and passed through a syringe filter. The concentrated residue was separated using column chromatography (Combi-Flash) eluting with 0-100% EtOAc in Heptane to obtain the title product as a light brown solid (64 mg, 90% yield).

^{1}H NMR (300 MHz, $CDCl_3$) δ 9.32 (d, *J* = 2.3 Hz, 1H), 8.76 (d, *J* = 2.3 Hz, 1H), 8.62 (d, *J* = 15.5 Hz, 1H), 8.29 (d, *J* = 8.6 Hz, 1H), 8.05 (s, 1H), 7.97 (dd, *J* = 8.2, 1.5 Hz, 1H), 7.84 – 7.65 (m, 7H), 7.66 – 7.57 (m, 2H), 7.56 – 7.46 (m, 1H), 6.72 (d, *J* = 7.3 Hz, 1H), 6.56 (d, *J* = 8.4 Hz, 1H), 4.97 (s, 2H), 4.70 (d, *J* = 4.8 Hz, 2H), 3.90 – 3.43 (m, 9H), 2.85 (s, 3H), 2.21 – 2.13 (m, 6H), 1.49 (s, 98), 1.43 (d, *J* = 7.1 Hz, 3H), 1.36 (s, 1H).

LCMS (Method 2) RT = 2.75 min, $[M+2H]/2^+$ = 507.0

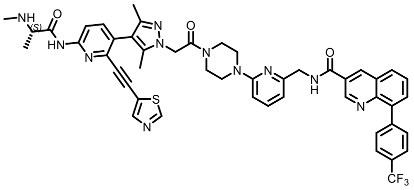

_N_-[[6-[4-[2-[3,5-dimethyl-4-[6-[[(2_S_)-2-(methylamino)propanoyl]amino]-2-(2-thiazol-5-ylethynyl)-3-pyridyl]pyrazol-1-yl]acetyl]piperazin-1-yl]-2-pyridyl]methyl]-8-[4-(trifluoromethyl)phenyl]quinoline-3-carboxamide (A531): *tert*-butyl *N*-[(1*S*)-2-[[5-[3,5-dimethyl-1-[2-oxo-2-[4-[6-[[[8-[4-(trifluoromethyl)phenyl]quinoline-3-carbonyl]amino]methyl]-2-pyridyl]piperazin-1-yl]ethyl]pyrazol-4-yl]-6-(2-thiazol-5-ylethynyl)-2-pyridyl]amino]-1-methyl-2-oxo-ethyl]-*N*-methyl-carbamate (**S25**) (64 mg, 0.063 mmol, 1 eq.) was subjected to **General Procedure 1.** HPLC and freeze-drying afforded the title compound as a white solid (26 mg, 43% yield).

^{1}H NMR (300 MHz, MeOD) δ 9.27 (d, J = 2.2 Hz, 1H), 8.95 (s, 1H), 8.85 (d, J = 2.3 Hz, 1H), 8.22 (d, J = 8.2 Hz, 1H), 8.07 (d, J = 6.6 Hz, 2H), 7.91 – 7.68 (m, 8H), 7.53 (t, J = 7.9 Hz, 1H), 6.71 (dd, J = 16.0, 7.9 Hz, 2H), 5.11 (s, 2H), 4.62 (s, 2H), 3.80 (d, J = 7.0 Hz, 1H), 3.63 (d, J = 27.4 Hz, 8H), 2.63 (s, 3H), 2.12 (s, 6H), 1.55 (d, J = 7.0 Hz, 3H).

^{19}F NMR (282 MHz, MeOD) δ -63.78.

^{13}C NMR (75 MHz, MeOD) δ 172.0, 168.0, 167.4, 160.1, 157.1, 157.0, 151.6, 149.7, 149.2, 147.8, 147.6, 144.4, 142.4, 141.5, 141.2, 140.4, 139.6, 137.6, 133.4, 132.3, 130.8, 130.7, 130.2, 128.9, 128.7, 128.6, 127.7, 125.8, 125.8, 124.1, 119.3, 116.6, 115.6, 112.0, 107.0, 96.0, 81.5, 59.8, 51.3, 46.3, 46.1, 45.7, 43.0, 32.9, 17.3, 12.4, 10.6.

LCMS (Method 2) RT = 1.99 min, $[M+2H]/2^+$ = 456.6

HRMS (ESI+) *m/z*: $[M+H]^+$ calcd for $C_{48}H_{45}F_3N_{11}O_3S$ 912.3380; found 912.3378.

*Synthesis of **A557** (**A531** IAP -ve control)*

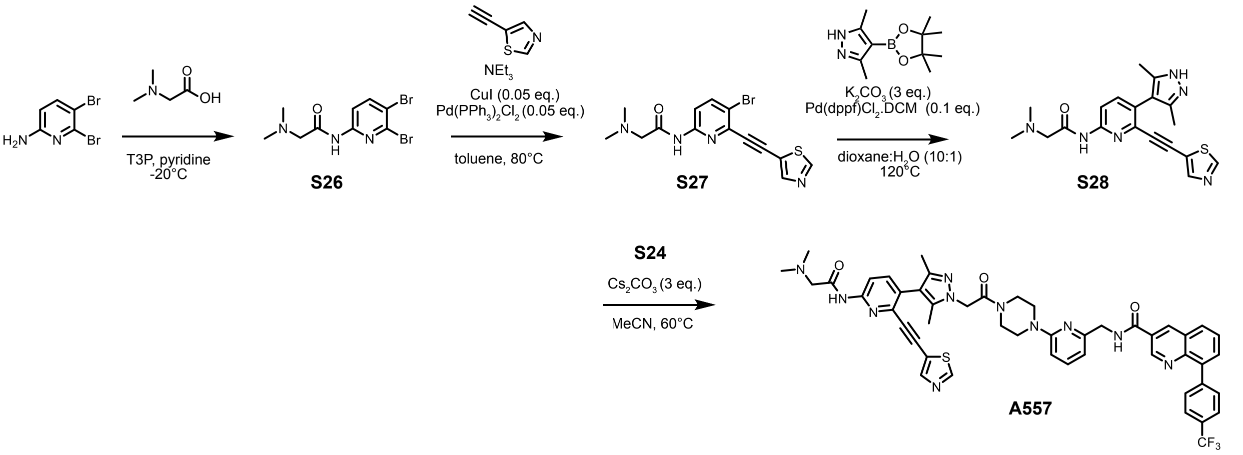

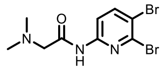

N-(5,6-dibromopyridin-2-yl)-2-(dimethylamino)acetamide (S26): To a cooled (-20 °C) solution of 5,6-dibromopyridin-2-amine (10 g, 39.69 mmol, 1 eq.) and dimethylglycine (8.1 g, 79.39 mmol, 2.0 eq.) in pyridine (300.0 mL) was added T3P 50% in EtOAc (58.5 mL, 198.49 mmol, 5.0 eq.) drop wise. The reaction mixture was stirred at the same temperature for 6 h. Upon completion of the starting material, reaction mixture was concentrated and diluted with water and 1N HCl solution and extracted with EtOAc (x2). The aqueous layer was neutralised with saturated aq. $NaHCO_3$ solution (till pH become neutral) and extracted with EtOAc. The organic layer was evaporated under reduced pressure to give the product as a pale brown solid (6.2 g, 77% yield).

^1H NMR (300 MHz, $CDCl_3$) δ 9.74 (s, 1H), 8.15 (d, J = 8.6 Hz, 1H), 7.84 (d, J = 8.6 Hz, 1H), 3.09 (s, 2H), 2.36 (s, 6H).

LCMS (Method 2) RT = 1.12 min, $[M+H]^+$ = 335.8/337.8/339.8

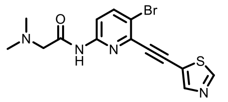

***N*-(5-bromo-6-(thiazol-5-ylethynyl)pyridin-2-yl)-2-(dimethylamino)acetamide (S27)**: To a degassed solution of *N*-(5,6-dibromopyridin-2-yl)-2-(dimethylamino)acetamide (**S26**) (6 g, 17.80 mmol, 1 eq.) and 5-ethynylthiazole (3.8 g, 35.58 mmol, 2.5 eq.) in toluene (50 mL) was added triethylamine (7.2 mL, 44.51 mmol, 3 eq.) and stirred at rt for 15 min. To this was added CuI (0.14 g, 0.742 mmol, 0.05 eq.) and $Pd(PPh_3)_2Cl_2$ (0.62 g, 0.742 mmol, 0.05 eq.) portionwise and the reaction was stirred at 80 °C for 16 h. The reaction mixture was then filtered through Celite and evaporated under reduced pressure to give crude product which was diluted with DCM and washed with water (x2). The organic layer was concentrated to get the crude product, which was further purified by flash chromatography 230-400 silica (neutralised by 3% triethylamine in pet ether). The desired product eluted with 30% EtOAc in pet ether and was isolated as a pale brown solid (3.8 g, 59% yield).

^{1}H NMR (300 MHz, $CDCl_3$) δ 9.80 (s, 1H), 8.82 (s, 1H), 8.27 – 8.17 (m, 2H), 7.90 (d, *J* = 8.9 Hz, 1H), 3.10 (s, 2H), 2.36 (s, 6H).

LCMS (Method 2) RT = 1.37 min, $[M+H]^+$ = 364.9/366.9

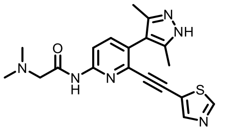

***N*-(5-(3,5-dimethyl-1*H*-pyrazol-4-yl)-6-(thiazol-5-ylethynyl)pyridin-2-yl)-2-(dimethylamino)acetamide (S28):** To a degassed solution of *N*-(5-bromo-6-(thiazol-5-ylethynyl)pyridin-2-yl)-2-(dimethylamino)acetamide (**S27**) (20.0 g, 0.055 mmol, 1 eq.), 3,5-dimethyl-4-(4,4,5,5-tetramethyl-1,3,2-dioxaborolan-2-yl)-1*H*-pyrazole (18.24 g, 0.082 mmol, 1.5 eq.) and K_2CO_3 (22.70 g, 0.164 mmol, 3 eq.) in 1,4 dioxane (200 mL)/ water (20 mL) was added Pd(dppf)Cl_2.DCM (4.47 g, 0.0054 mmol, 0.1 eq.). The reaction mixture was then stirred at 120 °C for 16 h in a sealed tube. Upon completion of the reaction, the reaction mixture was diluted with DCM and filtered through celite. The filtrate was diluted with DCM and washed with water. The organic layer was concentrated under reduced pressure to get the crude product, which was further purified by silica gel column chromatography and further purified by RP column (C18 cartridge, ABC in water and acetonitrile as eluent, product eluted with 26% acetonitrile in ABC) to isolate the pure compound as an off-white solid (5.1 g, 24% yield).

^{1}H NMR (300 MHz, $CDCl_3$) δ 9.84 (s, 1H), 8.74 (s, 1H), 8.36 (d, *J* = 8.5 Hz, 1H), 7.97 (s, 1H), 7.62 (d, *J* = 8.5 Hz, 1H), 3.14 (s, 2H), 2.39 (s, 6H), 2.24 (s, 6H).

LCMS (Method 2) RT = 1.12 min, $[M+H]^+$ = 381.0

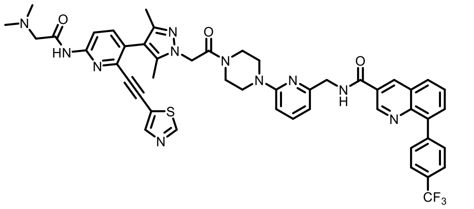

***N*-[[6-[4-[2-[4-[6-[[2-(dimethylamino)acetyl]amino]-2-(2-thiazol-5-ylethynyl)-3-pyridyl]-3,5-dimethyl-pyrazol-1-yl]acetyl]piperazin-1-yl]-2-pyridyl]methyl]-8-[4-(trifluoromethyl)phenyl]quinoline-3-carboxamide (A557)**: *N*-[[6-[4-(2-chloroacetyl)piperazin-1-yl]-2-pyridyl]methyl]-8-[4-(trifluoromethyl)phenyl]quinoline-3-carboxamide (**S24**) (30.0 mg, 0.053 mmol, 1 eq.), 2-(dimethylamino)-*N*-[5-(3,5-dimethyl-1*H*-pyrazol-4-yl)-6-(2-thiazol-5-ylethynyl)-2-pyridyl]acetamide (**S28**) (20.1 mg, 0.053 mmol, 1 eq.) and Cs_2CO_3 (52 mg, 0.16 mmol, 3 eq.) were dissolved in acetonitrile (1.5 mL) and heated at 60 °C for 16 h. When the reaction was complete, the reaction mixture was concentrated, redissolved in DCM and filtered. The filtrate was concentrated and further purified by prepLCMS. The clean fractions were collected and lyophilized to give the formate salt of the product as a white solid (29 mg, 52% yield).

^{1}H NMR (300 MHz, MeOD) δ 9.25 (d, *J* = 2.3 Hz, 1H), 8.94 (s, 1H), 8.83 (d, *J* = 2.3 Hz, 1H), 8.22 (d, *J* = 8.3 Hz, 1H), 8.12 – 8.00 (m, 2H), 7.89 – 7.65 (m, 7H), 7.51 (t, *J* = 7.9 Hz, 1H), 6.70 (dd, *J* = 18.5, 7.9 Hz, 2H), 5.09 (s, 2H), 4.61 (s, 2H), 3.61 (d, *J* = 27.9 Hz, 8H), 3.37 (s, 2H), 2.51 (s, 6H), 2.11 (s, 6H).

^{13}C NMR (75 MHz, MeOD) δ 170.6, 168.0, 167.3, 160.1, 157.1, 157.0, 151.5, 149.7, 149.3, 147.7, 147.5, 144.4, 142.5, 141.3, 141.2, 140.4, 139.6, 137.6, 133.4, 132.3, 130.7, 130.6, 130.2, 129.9, 128.9, 128.6, 128.6, 127.7, 125.8, 125.8, 125.7, 124.1, 119.3, 116.6, 115.3, 112.0, 107.0, 95.9, 81.7, 63.4, 51.3, 46.3, 46.2, 46.1, 45.8, 43.0, 12.4, 10.7.

^{19}F NMR (282 MHz, MeOD) δ -63.75.

LCMS (Method 2) RT = 1.99 min, $[M+2H]/2^{+}$ = 456.7

HRMS (ESI+) *m/z*: $[M+H]^{+}$ calcd for $C_{48}H_{45}F_3N_{11}O_3S$ 912.3380; found 912.3377.

*Synthesis of **A423** (**A531** TEAD -ve control)*

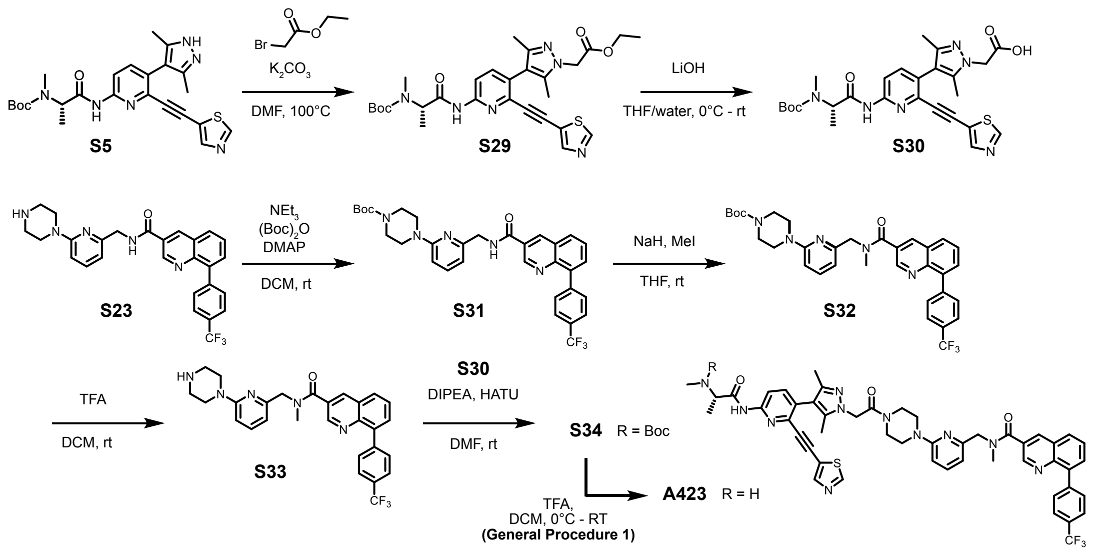

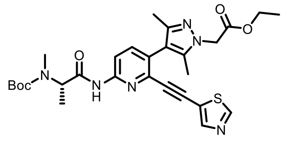

ethyl 2-[4-[6-[[(2*S*)-2-[*tert*-butoxycarbonyl(methyl)amino]propanoyl]amino]-2-(2-thiazol-5-ylethynyl)-3-pyridyl]-3,5-dimethyl-pyrazol-1-yl]acetate (S29): tert-butyl ((2*S*)-1-((5-(3,5-dimethyl-1H-pyrazol-4-yl)-6-(thiazol-5-ylethynyl)pyridin-2-yl)amino)-1-oxopropan-2-yl)(methyl)carbamate (**S5**) (500 mg, 1.0 mmol, 1 eq.), K_2CO_3 (430 mg, 3.1 mmol, 3 eq.), and ethyl 2-bromoacetate (230 µL, 2.1 mmol, 2 eq.) were combined in DMF (3 mL) and heated to 100 °C overnight. Once the reaction was complete (monitored by LCMS) the reaction mixture was diluted with EtOAc and then washed with chilled brine (x4) to remove

DMF. The organic layers were dried with $MgSO_4$ and evaporated under reduced pressure to obtain the crude material. The crude residue was purified by column chromatography (Combi-Flash) eluting with MeOH/DCM (0-5%) to obtain the title product as a brown oil (472 mg, 80%).

^{1}H NMR (300 MHz, $CDCl_3$) δ 8.73 (s, 1H), 8.54 (s, 1H), 8.29 (d, *J* = 8.6 Hz, 1H), 8.05 (s, 1H), 7.63 (d, *J* = 8.6 Hz, 1H), 4.84 (s, 2H), 4.31 – 4.04 (m, 3H), 2.85 (s, 3H), 2.17 (d, *J* = 9.8 Hz, 6H), 1.49 (s, 9H), 1.43 (d, *J* = 7.0 Hz, 3H), 1.27 (t, *J* = 7.2 Hz, 4H).

LCMS (Method 2) RT = 2.41 min, $[M+H]^+$ = 567.2

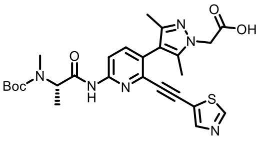

2-[4-[6-[[(2*S*)-2-[*tert*-butoxycarbonyl(methyl)amino]propanoyl]amino]-2-(2-thiazol-5-ylethynyl)-3-pyridyl]-3,5-dimethyl-pyrazol-1-yl]acetic acid (S30): ethyl 2-[4-[6-[[(2*S*)-2-[*tert*-butoxycarbonyl(methyl)amino]propanoyl]amino]-2-(2-thiazol-5-ylethynyl)-3-pyridyl]-3,5-dimethyl-pyrazol-1-yl]acetate (**S29**) (472 mg, 0.83 mmol, 1 eq.) was dissolved in a THF (5 mL) / water (1 mL) solution and cooled to 0 °C. The mixture was treated with lithium hydroxide (99.6 mg, 4.16 mmol, 5 eq.) and left stirring overnight. Once the reaction was complete (monitored by LCMS), the reaction was diluted with ethyl acetate and acidified with 10% aq. citric acid solution. The organic layer was collected, and the aqueous layer was extracted twice more with ethyl acetate. The organic layers were combined and washed twice with water followed by brine, then dried over $MgSO_4$ and concentrated under reduced pressure to obtain the title compound as a yellow solid (400 mg, 90% yield).

^{1}H NMR (300 MHz, MeOD) δ 8.89 (s, 1H), 8.12 (d, J = 8.6 Hz, 1H), 7.96 (s, 1H), 7.64 (d, J = 8.6 Hz, 1H), 4.82 (s, 2H), 4.57 – 4.48 (m, 1H), 2.87 (s, 3H), 2.08 (s, 3H), 2.05 (s, 3H), 1.41 – 1.34 (m, 12H).

LCMS (Method 2) RT = 2.28 min, $[M+H]^+$ = 539.3

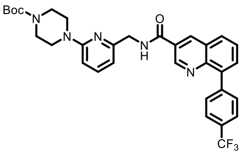

***tert*-butyl 4-[6-[[[8-[4-(trifluoromethyl)phenyl]quinoline-3-carbonyl]amino]methyl]-2-pyridyl]piperazine-1-carboxylate (S31):** *N*-[(6-piperazin-1-yl-2-pyridyl)methyl]-8-[4-(trifluoromethyl)phenyl]quinoline-3-carboxamide (**S23**) (200 mg, 0.41 mmol, 1 eq.) was dissolved in DCM (5 mL) and treated with triethylamine (0.068 mL, 0.49 mmol, 1.2 eq.), di-*tert*-butyl dicarbonate (130 mg, 0.61 mmol, 1.5 eq.) followed by DMAP (4.97 mg, 0.041 mmol, 0.1 eq.) under nitrogen and then left to stir at rt overnight. Once the reaction was complete (monitored by LCMS), the reaction mixture was diluted with DCM and the organic solution was washed with 10% citric acid solution followed by brine. The organic phase was dried ($MgSO_4$) and concentrated under reduced pressure to a crude

residue. The residue was separated using column chromatography with a gradient of 0-100% EtOAc in heptane to yield the title compound as a white solid (100 mg, 42% yield).

^{1}H NMR (300 MHz, $CDCl_3$) δ 9.33 (d, *J* = 2.3 Hz, 1H), 8.77 (d, *J* = 2.3 Hz, 1H), 8.08 – 7.93 (m, 1H), 7.87 – 7.70 (m, 7H), 7.64 (t, *J* = 4.7 Hz, 1H), 7.52 (dd, *J* = 8.5, 7.3 Hz, 1H), 6.68 (d, *J* = 7.3 Hz, 1H), 6.59 (d, *J* = 8.4 Hz, 1H), 4.71 (d, *J* = 4.5 Hz, 2H), 3.56 (s, 8H), 1.48 (s, 9H).

LCMS (Method 2) RT = 2.64 min, $[M+H]^+$ = 592.4

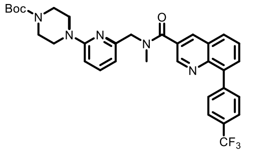

***tert*-butyl 4-[6-[[methyl-[8-[4-(trifluoromethyl)phenyl]quinoline-3-carbonyl]amino]methyl]-2-pyridyl]piperazine-1-carboxylate (S32):** *tert*-butyl 4-[6-[[[8-[4-(trifluoromethyl)phenyl]quinoline-3-carbonyl]amino]methyl]-2-pyridyl]piperazine-1-carboxylate (**S31**) (100 mg, 0.17 mmol, 1 eq.) was dissolved in dry THF (5 mL) and treated with sodium hydride (14 mg, 0.34 mmol, 2 eq.) at rt. The reaction bubbled and was stirred for 30 min, after which the gas evolution had ceased. Iodomethane (12.6 µL, 0.203 mmol, 1.2 eq.) was added and the reaction stirred at rt. Once the reaction was complete (monitored by LCMS), the reaction was diluted with EtOAc then quenched with water and brine. The organic phase was collected, and the aqueous phase was extracted twice more with EtOAc. The combined organic phases were dried over $MgSO_4$ and concentrated under reduced pressure to obtain the crude residue. The crude material was purified by Combi-Flash with an eluent of EtOAc:n-Hept (0-100%) to obtain the title compound as a clear oil (67 mg, 65% yield). NMR in $CDCl_3$ shows evidence of rotamers, LCMS shows a single peak.

^{1}H NMR (300 MHz, $CDCl_3$) δ 9.06 (d, *J* = 2.3 Hz, 1H), 8.42 (d, *J* = 2.2 Hz, 1H), 7.92 – 7.69 (m, 7H), 7.70 – 7.61 (m, 1H), 7.54 – 7.43 (m, 1H), 6.55 (d, *J* = 8.6 Hz, 1H), 6.49 (d, *J* = 7.3 Hz, 1H), 4.64 (d, *J* = 82.9 Hz, 2H), 3.54 (d, *J* = 3.5 Hz, 8H), 3.13 (d, *J* = 6.5 Hz, 3H), 1.48 (s, 9H).

LCMS (Method 2) RT = 2.91 min, $[M+H]^+$ = 606.4

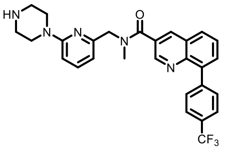

***N*-methyl-*N*-[(6-piperazin-1-yl-2-pyridyl)methyl]-8-[4-(trifluoromethyl)phenyl]quinoline-3-carboxamide (S33) :** *tert*-butyl 4-[6-[[methyl-[8-[4-(trifluoromethyl)phenyl]quinoline-3-carbonyl]amino]methyl]-2-pyridyl]piperazine-1-carboxylate (**S34**) (67 mg, 0.11 mmol, 1 eq.) was dissolved in DCM (3 mL) and treated with TFA (167 µL, 2.21 mmol, 20 eq.) and left to stir at rt. After 1 h, 50% conversion had occurred, another portion of TFA (167 µL, 2.21 mmol, 20 eq.) was added and the reaction was left to stir for 16 h. Once the reaction was complete, the mixture was carefully treated with sat. $NaHCO_3$. The

organic layer was collected and the aqueous later extracted twice with DCM. The combined organic layers were washed with brine, dried ($MgSO_4$) then concentrated under reduced pressure to give the title product an off-white solid (53.0 mg, 95% yield). NMR in $CDCl_3$ shows evidence of rotamers. LCMS shows a single peak.

^{1}H NMR (300 MHz, $CDCl_3$) δ 9.10 – 9.01 (m, 1H), 8.60 – 8.27 (m, 1H), 7.85 – 7.70 (m, 6H), 7.72 – 7.57 (m, 1H), 7.56 – 7.40 (m, 1H), 6.55 (d, *J* = 8.5 Hz, 1H), 6.46 (d, *J* = 7.2 Hz, 1H), 4.63 (d, *J* = 84.7 Hz, 2H), 3.75 – 3.40 (m, 4H), 3.13 (d, *J* = 6.5 Hz, 3H), 3.05 – 2.81 (m, 4H).

LCMS (Method 2) RT = 1.87 min, $[M+H]^+$ = 506.2

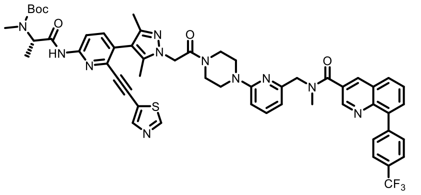

***tert*-butyl *N*-[(1*S*)-2-[[5-[3,5-dimethyl-1-[2-[4-[6-[[methyl-[8-[4-(trifluoromethyl)phenyl]quinoline-3-carbonyl]amino]methyl]-2-pyridyl]piperazin-1-yl]-2-oxo-ethyl]pyrazol-4-yl]-6-(2-thiazol-5-ylethynyl)-2-pyridyl]amino]-1-methyl-2-oxo-ethyl]-*N*-methyl-carbamate (S34):** 2-[4-[6-[[(2*S*)-2-[*tert*-butoxycarbonyl(methyl)amino]propanoyl]amino]-2-(2-thiazol-5-ylethynyl)-3-pyridyl]-3,5-dimethyl-pyrazol-1-yl]acetic acid (**S30**) (25 mg, 0.046 mmol, 1 eq.) was reacted with *N*-methyl-*N*-[(6-piperazin-1-yl-2-pyridyl)methyl]-8-[4-(trifluoromethyl)phenyl]quinoline-3-carboxamide (**S33**) (23 mg, 0.046 mmol, 1 eq.) according to **General Procedure 2.** Column chromatography (0-10% MeOH in DCM) afforded the title compound as a yellow oil (40 mg, 84% yield). NMR in $CDCl_3$ shows evidence of rotamers, LCMS shows a single peak.

^{1}H NMR (300 MHz, $CDCl_3$) δ 9.05 (d, *J* = 2.2 Hz, 1H), 8.69 (s, 1H), 8.57 – 8.50 (m, 1H), 8.43 – 8.37 (m, 1H), 8.29 (d, *J* = 8.6 Hz, 1H), 8.09 (s, 1H), 7.88 – 7.59 (m, 8H), 7.56 – 7.44 (m, 1H), 6.66 – 6.43 (m, 2H), 4.97 (d, *J* = 4.5 Hz, 2H), 4.78 (s, 1H), 4.52 (s, 1H), 3.81 – 3.64 (m, 7H), 3.63 – 3.50 (m, 3H), 3.15 (s, 2H), 2.95 (s, 1H), 2.85 (s, 3H), 2.19 (d, *J* = 3.3 Hz, 6H), 1.50 (s, 9H), 1.43 (d, *J* = 7.0 Hz, 3H).

LCMS (Method 2) RT = 2.94 min, $[M+2H]/2^+$ = 513.8

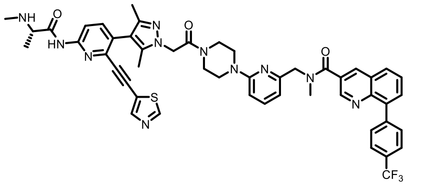

***N*-[[6-[4-[2-[3,5-dimethyl-4-[6-[[(2*S*)-2-(methylamino)propanoyl]amino]-2-(2-thiazol-5-ylethynyl)-3-pyridyl]pyrazol-1-yl]acetyl]piperazin-1-yl]-2-pyridyl]methyl]-N-methyl-8-[4-(trifluoromethyl)phenyl]quinoline-3-carboxamide (A423):** tert-butyl N-[(1S)-2-[[5-[3,5-dimethyl-1-[2-[4-[6-[[methyl-[8-[4-(trifluoromethyl)phenyl]quinoline-3-carbonyl]amino]methyl]-2-pyridyl]piperazin-1-yl]-2-oxo-ethyl]pyrazol-4-yl]-6-(2-thiazol-5-ylethynyl)-2-pyridyl]amino]-1-methyl-2-oxo-ethyl]-*N*-methyl-

carbamate (**S34**) (40 mg, 0.039 mmol, 1 eq.) was subjected to **General Procedure 1.** HPLC and freeze drying afforded the title compound as a white solid (13 mg, 36% yield). NMR in MeOD-d4 shows evidence of rotamers, LCMS shows one peak.

^{1}H NMR (500 MHz, MeOD) δ 8.96 (d, *J* = 3.1 Hz, 2H), 8.60 – 8.51 (m, 1H), 8.25 (d, *J* = 8.6 Hz, 1H), 8.10 (s, 1H), 8.03 – 7.97 (m, 1H), 7.90 – 7.83 (m, 2H), 7.82 – 7.73 (m, 5H), 7.54 (s, 1H), 6.76 (d, *J* = 8.5 Hz, 1H), 6.58 (d, *J* = 7.2 Hz, 1H), 5.14 (s, 2H), 4.58 (s, 3H), 3.74 (d, *J* = 12.1 Hz, 6H), 3.66 – 3.52 (m, 3H), 3.16 (d, *J* = 13.9 Hz, 3H), 2.53 (s, 3H), 2.16 (d, *J* = 3.7 Hz, 6H), 1.46 (d, *J* = 7.0 Hz, 3H). 2:1 rotamer splitting

^{19}F NMR (471 MHz, MeOD) δ -63.85 (d, *J* = 5.3 Hz), -76.91.

^{13}C NMR (126 MHz, MeOD) δ 174.1, 172.3, 167.4, 160.4, 157.1, 155.0, 151.8, 149.5, 149.4, 149.2, 147.8, 146.6, 144.5, 142.4, 141.5, 141.2, 140.5, 139.8, 136.8, 136.7, 133.0, 132.3, 130.9, 130.6, 130.3, 130.2, 130.1, 128.8, 128.6, 127.0, 125.8 (q, *J* = 3.8 Hz), 124.8, 119.3, 116.7, 115.5, 112.6, 107.6, 107.3, 95.9, 81.5, 60.5, 57.6, 53.7, 51.3, 46.1, 45.7, 43.0, 39.0, 34.7, 33.8, 18.2, 12.4, 10.6.

LCMS (Method 2) RT = 2.07 min, $[M+H]^+$ = 926.4

HRMS (ESI+) *m/z*: $[M+H]^+$ calcd for $C_{49}H_{47}F_3N_{11}O_3S$ 926.3536; found 926.3531.

Synthesis of ***A538***

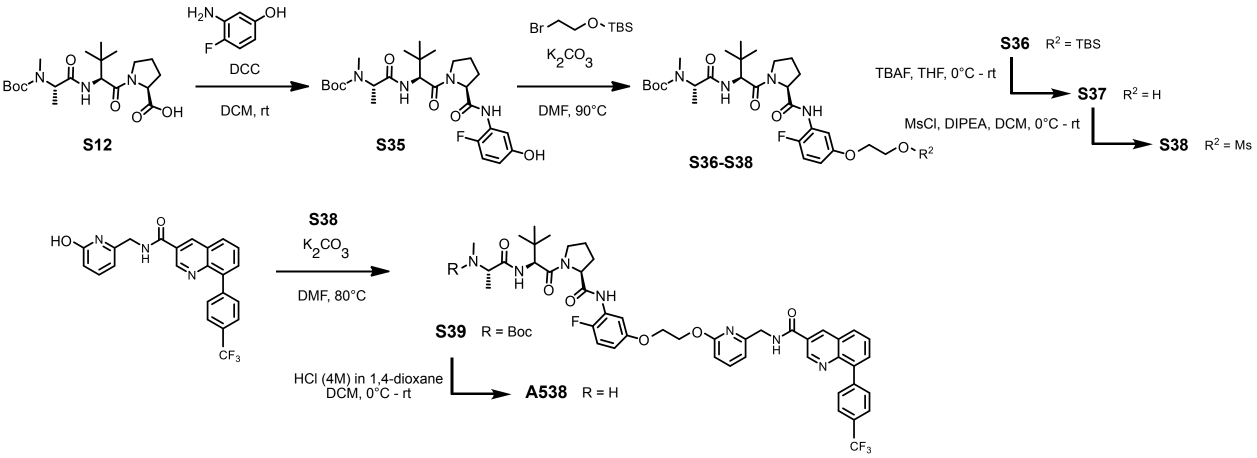

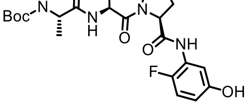

***tert*-butyl *N*-[(1*S*)-2-[[(1*S*)-1-[(2*S*)-2-[(2-fluoro-5-hydroxy-phenyl)carbamoyl]pyrrolidine-1-carbonyl]-2,2-dimethyl-propyl]amino]-1-methyl-2-oxo-ethyl]-*N*-methyl-carbamate (S35):** To a suspension of (2*S*)-1-[(2*S*)-2-[[(2*S*)-2-[*tert*-butoxycarbonyl(methyl)amino]propanoyl]amino]-3,3-dimethyl-butanoyl]pyrrolidine-2-carboxylic acid (**S12**) (500 mg, 1.2 mmol, 1 eq.) and 3-amino-4-fluoro-phenol (310

mg, 2.4 mmol, 2 eq.) in DCM (15 mL) at rt was added DCC (499 mg, 2.42 mmol, 2 eq.). The mixture was stirred at rt for 30 min. The mixture was filtered and subjected to column chromatography (Combi-Flash) eluting with 0-5% MeOH in DCM to yield the title product as an off-white solid (450 mg, 71% yield).

^{1}H NMR (300 MHz, $CDCl_3$) δ 9.38 (s, 1H), 7.95 (dd, *J* = 6.4, 3.0 Hz, 1H), 7.04 – 6.84 (m, 3H), 6.50 (dt, *J* = 8.9, 3.5 Hz, 1H), 4.84 (dd, *J* = 7.9, 2.8 Hz, 1H), 4.78 – 4.54 (m, 2H), 3.88 (q, *J* = 8.6 Hz, 1H), 3.69 (td, *J* = 8.7, 3.8 Hz, 1H), 2.79 (s, 3H), 2.57 – 2.40 (m, 1H), 2.23 – 1.85 (m, 3H), 1.49 (s, 9H), 1.32 (d, *J* = 7.1 Hz, 3H), 0.98 (s, 9H).

LCMS (Method 2) RT = 2.13 min, $[M+H]^-$ = 521.4

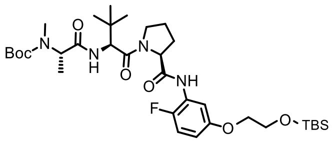

***tert*-butyl *N*-[(1*S*)-2-[[(1*S*)-1-[(2*S*)-2-[[5-[2-[*tert*-butyl(dimethyl)silyl]oxyethoxy]-2-fluoro-phenyl]carbamoyl]pyrrolidine-1-carbonyl]-2,2-dimethyl-propyl]amino]-1-methyl-2-oxo-ethyl]-*N*-methyl-carbamate (S36):** A mixture of 2-bromoethoxy-*tert*-butyl-dimethyl-silane (42 mg, 0.18 mmol, 1.3 eq.), K_2CO_3 (56 mg, 0.40 mmol, 3 eq.), and *tert*-butyl *N*-[(1*S*)-2-[[(1*S*)-1-[(2*S*)-2-[(2-fluoro-5-hydroxy-phenyl)carbamoyl]pyrrolidine-1-carbonyl]-2,2-dimethyl-propyl]amino]-1-methyl-2-oxo-ethyl]-*N*-methyl-carbamate (**S35**) (80 mg, 0.13 mmol, 1 eq.) in DMF (3 mL) was heated to 90°C for 2 h. Once the reaction was complete (monitored by LCMS), the reaction mixture was cooled, diluted in EtOAc, partitioned with water and the aqueous layer was extracted (x3). The combined organics were washed with brine and dried over $MgSO_4$ and concentrated. The crude residue was purified by column chromatography using a gradient of 0-80% EtOAc in heptane to yield the title product as a yellow solid (67 mg, 73% yield).

^{1}H NMR (300 MHz, $CDCl_3$) δ 9.41 (d, *J* = 2.8 Hz, 1H), 7.93 (dd, *J* = 6.4, 3.1 Hz, 1H), 6.94 (dd, *J* = 10.3, 9.0 Hz, 1H), 6.54 (ddd, *J* = 9.0, 3.9, 3.1 Hz, 1H), 4.83 (dd, *J* = 7.9, 2.2 Hz, 1H), 4.71 (s, 1H), 4.62 (d, *J* = 9.3 Hz, 1H), 4.05 – 3.88 (m, 4H), 3.89 – 3.75 (m, 1H), 3.66 (ddd, *J* = 10.0, 7.9, 3.4 Hz, 1H), 2.78 (s, 3H), 2.54 (ddt, *J* = 12.2, 6.2, 2.8 Hz, 1H), 2.25 – 1.97 (m, 2H), 1.99 – 1.77 (m, 1H), 1.49 (s, 9H), 1.31 (d, *J* = 7.1 Hz, 3H), 1.26 – 1.22 (m, 1H), 0.97 (s, 9H), 0.88 (s, 9H), 0.07 (s, 6H).

LCMS (Method 2) RT = 3.31, $[M+H]^-$ = 679.4

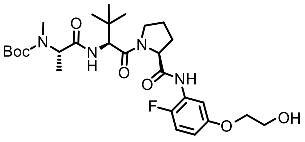

***tert*-butyl *N*-[(1*S*)-2-[[(1*S*)-1-[(2*S*)-2-[[2-fluoro-5-(2-hydroxyethoxy)phenyl]carbamoyl]pyrrolidine-1-carbonyl]-2,2-dimethyl-propyl]amino]-1-methyl-2-oxo-ethyl]-*N*-methyl-carbamate (S37):** To a solution of *tert*-butyl *N*-[(1*S*)-2-[[(1*S*)-1-[(2*S*)-2-[[5-[2-[*tert*-butyl(dimethyl)silyl]oxyethoxy]-2-fluoro-phenyl]carbamoyl]pyrrolidine-1-carbonyl]-2,2-dimethyl-propyl]amino]-1-methyl-2-oxo-ethyl]-*N*-methyl-

carbamate (**S36**) (67 mg, 0.098 mmol, 1 eq.) in THF (2 mL) at 0 °C was added TBAF (1.0 M in THF) (200 µL, 0.20 mmol, 2 eq.) and the mixture was allowed to reach rt. Once the reaction was complete (monitored by LCMS), the reaction mixture was diluted in EtOAc and washed with NH_4Cl, then brine. The organic layer was dried over $MgSO_4$ and concentrated. The crude was purified by column chromatography using a 0-10% gradient of MeOH in DCM to obtain the title product as a colorless solid (47 mg, 84% yield). NMR in $CDCl_3$ shows evidence of rotamers. LCMS shows a single peak.

^{1}H NMR (300 MHz, $CDCl_3$) δ 9.45 (s, 1H), 7.97 (dd, J = 6.4, 3.1 Hz, 1H), 6.96 (dd, J = 10.4, 9.0 Hz, 1H), 6.55 (dt, J = 9.0, 3.8 Hz, 1H), 4.83 (dd, J = 8.0, 2.3 Hz, 1H), 4.72 (s, 1H), 4.62 (d, J = 9.2 Hz, 1H), 4.10 – 3.95 (m, 3H), 3.91 (q, J = 3.9 Hz, 3H), 3.85 (td, J = 8.1, 5.8 Hz, 1H), 3.67 (ddd, J = 10.0, 7.9, 3.6 Hz, 1H), 2.78 (s, 3H), 2.54 (ddt, J = 12.4, 6.4, 3.0 Hz, 1H), 2.18 (s, 1H), 2.07 (dq, J = 7.0, 3.5 Hz, 1H), 1.98 – 1.79 (m, 1H), 1.49 (s, 9H), 1.32 (d, J = 7.1 Hz, 3H), 1.20 (d, J = 6.1 Hz, 3H), 0.97 (s, 9H).

LCMS (Method 2) RT = 2.13 min, $[M+H-Boc]^+$ = 467.4, $[M-H]^-$ = 565.4

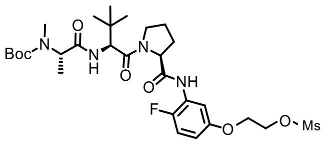

2-[4-[[(2*S*)-1-[(2*S*)-2-[[(2*S*)-2-[*tert*-butoxycarbonyl(methyl)amino]propanoyl]amino]-3,3-dimethyl-butanoyl]pyrrolidine-2-carbonyl]amino]-3-fluoro-phenoxy]ethyl methanesulfonate (S38): To a solution of *tert*-butyl *N*-[(1*S*)-2-[[(1*S*)-1-[(2*S*)-2-[[2-fluoro-4-(2-hydroxyethoxy)phenyl]carbamoyl]pyrrolidine-1-carbonyl]-2,2-dimethyl-propyl]amino]-1-methyl-2-oxo-ethyl]-*N*-methyl-carbamate (**S37**) (47 mg, 0.083 mmol, 1 eq.) in DCM (2 mL) at 0 ℃ was added methanesulfonyl chloride (9.6 µL, 0.12 mmol, 1.5 eq.) and DIPEA (43 µL, 0.25 mmol, 3 eq.) and the mixture was left to stir at rt. Once the reaction was complete (monitored by LCMS), the mixture was diluted in DCM and partitioned with sat. $NaHCO_3$. The aqueous layer was extracted (x3) and the combined organics were washed with brine and dried over $MgSO_4$ and concentrated. The residue was separated by column chromatography (Combi-Flash) eluting with 0-100% EtOAc in heptane to obtain the title product as a colorless oil (44.0 mg, 82% yield).

^{1}H NMR (300 MHz, $CDCl_3$) δ 9.52 – 9.45 (m, 1H), 7.97 (dd, J = 6.3, 3.1 Hz, 1H), 6.97 (dd, J = 10.3, 9.0 Hz, 1H), 6.54 (dt, J = 9.0, 3.5 Hz, 1H), 4.82 (dd, J = 7.9, 2.4 Hz, 1H), 4.71 (s, 1H), 4.61 (d, J = 9.2 Hz, 1H), 4.56 – 4.47 (m, 2H), 4.26 – 4.15 (m, 2H), 3.85 (td, J = 9.8, 7.5 Hz, 1H), 3.66 (td, J = 8.8, 3.6 Hz, 1H), 3.07 (s, 3H), 2.78 (s, 3H), 2.52 (ddt, J = 12.4, 6.3, 3.0 Hz, 1H), 2.13 – 1.98 (m, 1H), 1.98 – 1.79 (m, 1H), 1.49 (s, 9H), 1.31 (d, J = 7.1 Hz, 3H), 0.97 (s, 9H).

LCMS (Method 2) RT = 2.35 min, $[M+H-Boc]^+$ = 545.3, $[M-H]^-$ = 643.4

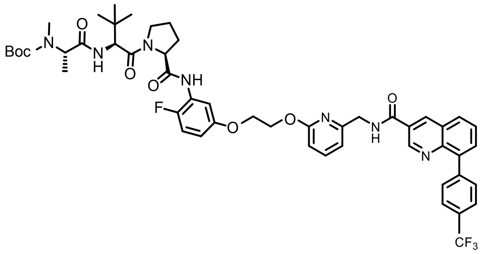

***tert*-butyl *N*-[(1*S*)-2-[[(1*S*)-1-[(2*S*)-2-[[2-fluoro-5-[2-[3-[[[8-[4-(trifluoromethyl)phenyl]quinoline-3-carbonyl]amino]methyl]phenoxy]ethoxy]phenyl]carbamoyl]pyrrolidine-1-carbonyl]-2,2-dimethyl-propyl]amino]-1-methyl-2-oxo-ethyl]-*N*-methyl-carbamate (S39):** A mixture of 2-[3-[[(2*S*)-1-[(2*S*)-2-[[(2*S*)-2-[*tert*-butoxycarbonyl(methyl)amino]propanoyl]amino]-3,3-dimethyl-butanoyl]pyrrolidine-2-carbonyl]amino]-4-fluoro-phenoxy]ethyl methanesulfonate (**S38**) (44 mg, 0.068 mmol, 1 eq.), *N*-[(3-hydroxyphenyl)methyl]-8-[4-(trifluoromethyl)phenyl]quinoline-3-carboxamide (29 mg, 0.068 mmol, 1 eq.) and K_2CO_3 (28 mg, 0.20 mmol, 3 eq.) in DMF (1.5 mL) was heated to 80 ℃ overnight. Once the reaction was complete (monitored by LCMS), the mixture was quenched with NH_4Cl and diluted with EtOAc. The aqueous layer was extracted with EtOAc (x3) and the combined organics were washed with brine and dried over $MgSO_4$. The crude residue was separated by column chromatography (Combi-Flash) eluting with 0-100% EtOAc in heptane to yield obtain the title product as a white solid (31 mg, 47% yield).

^{1}H NMR (300 MHz, $CDCl_3$) δ 9.41 (s, 1H), 9.32 (d, *J* = 2.3 Hz, 1H), 8.67 (d, *J* = 2.3 Hz, 1H), 8.00 (dd, *J* = 6.4, 3.1 Hz, 1H), 7.94 (dd, *J* = 8.2, 1.5 Hz, 1H), 7.87 – 7.65 (m, 6H), 7.29 (d, *J* = 8.1 Hz, 1H), 7.02 – 6.77 (m, 6H), 6.57 (dt, *J* = 9.1, 3.5 Hz, 1H), 4.79 (dd, *J* = 8.0, 2.4 Hz, 1H), 4.70 (d, *J* = 5.6 Hz, 2H), 4.61 (d, *J* = 9.2 Hz, 2H), 4.28 (s, 4H), 3.92 – 3.78 (m, 1H), 3.72 – 3.55 (m, 1H), 2.78 (s, 3H), 2.55 – 2.42 (m, 1H), 1.95 – 1.77 (m, 1H), 1.65 – 1.58 (m, 2H), 1.49 (s, 10H), 1.32 (d, *J* = 7.1 Hz, 3H), 0.97 (s, 9H).

LCMS (Method 2) RT = 3.75 min, $[M+H]^+$ = 545.3

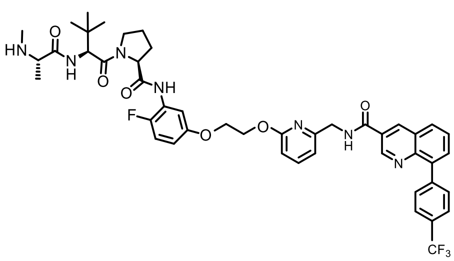

***N*-[[3-[2-[3-[[(2*S*)-1-[(2*S*)-3,3-dimethyl-2-[[(2*S*)-2-(methylamino)propanoyl]amino]butanoyl]pyrrolidine-2-carbonyl]amino]-4-fluoro-phenoxy]ethoxy]phenyl]methyl]-8-[4-(trifluoromethyl)phenyl]quinoline-3-carboxamide (A538):** To a solution of *tert*-butyl *N*-[(1*S*)-2-[[(1*S*)-1-[(2*S*)-2-[[2-fluoro-5-[2-[3-[[[8-[4-(trifluoromethyl)phenyl]quinoline-3-carbonyl]amino]methyl]phenoxy]ethoxy]phenyl]carbamoyl]pyrrolidine-1-carbonyl]-2,2-dimethyl-propyl]amino]-1-methyl-2-oxo-ethyl]-*N*-methyl-carbamate (**S39**) (22 mg, 0.023 mmol, 1 eq.) in DCM (2

mL) at 0 °C was added 4 M HCl in 1,4-dioxane (0.057 mL, 0.23 mmol, 10 eq.) and the mixture was stirred overnight at rt. The solvents were removed by aspiration and the residue was purified by acidic RP HPLC. Product containing fractions were dried then combined and further freeze dried to obtain the title product as a white solid (13 mg, 61% yield). NMR in MeOD-d4 shows evidence of rotamers, LCMS shows a single peak.

^{1}H NMR (300 MHz, MeOD) δ 10.01 (d, *J* = 2.4 Hz, 1H), 9.57 (dd, *J* = 6.9, 2.3 Hz, 1H), 8.80 (dd, *J* = 8.3, 1.5 Hz, 1H), 8.68 – 8.42 (m, 7H), 8.03 (t, *J* = 7.9 Hz, 1H), 7.83 – 7.71 (m, 3H), 7.64 (dd, *J* = 8.1, 2.5 Hz, 1H), 7.43 (dt, *J* = 9.0, 3.4 Hz, 1H), 5.48 – 5.34 (m, 4H), 5.10 – 4.94 (m, 4H), 4.71 (dt, *J* = 12.1, 6.1 Hz, 1H), 4.51 (dt, *J* = 9.4, 6.4 Hz, 1H), 4.43 (q, *J* = 6.9 Hz, 1H), 3.31 (s, 3H), 3.09 – 2.67 (m, 4H), 2.17 (d, *J* = 6.9 Hz, 3H), 1.84 (s, 9H).

^{13}C NMR (75 MHz, MeOD) δ 172.9, 172.4, 172.3, 171.8, 170.1, 167.8, 160.4, 156.2, 156.1, 151.2, 149.7, 148.0, 147.5, 144.4, 144.4, 141.5, 140.3, 137.5, 133.4, 132.3, 130.8, 130.7, 130.6, 130.2, 128.8, 128.6, 128.5, 128.5, 127.7, 127.7, 127.6, 125.8, 125.8, 125.7, 124.1, 121.3, 116.5, 116.2, 115.2, 114.5, 114.4, 111.9, 111.8, 110.8, 68.5, 67.8, 62.1, 59.3, 59.1, 58.9, 44.6, 37.1, 36.1, 35.8, 33.4, 33.0, 32.8, 32.8, 30.2, 27.0, 26.9, 26.9, 26.2, 25.6, 23.3, 17.6, 17.5.

^{19}F NMR (282 MHz, MeOD) δ -63.82 (d, *J* = 2.7 Hz), -76.86, -138.53 (ddd, *J* = 10.4, 6.5, 3.8 Hz).

LCMS (Method 2) RT = 2.39min, $[M+H]^+$ = 871.4

HRMS (ESI+) *m/z*: $[M+H]^+$ calcd for $C_{47}H_{51}F_4N_6O_6$ 871.3806; found 871.3802.

*Synthesis of **A559** (**A538** IAP -ve control)*

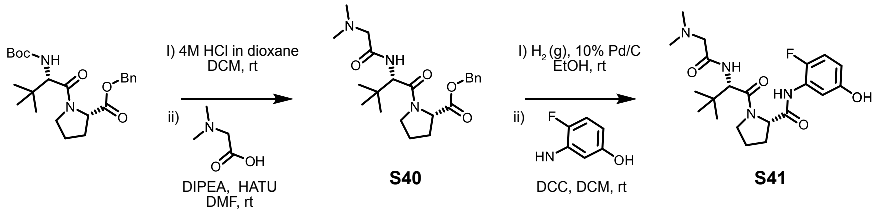

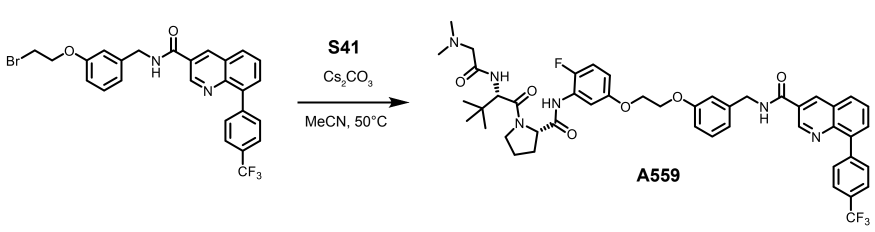

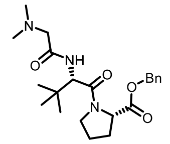

benzyl (2*S*)-1-[(2*S*)-2-[[2-(dimethylamino)acetyl]amino]-3,3-dimethyl-butanoyl]pyrrolidine-2-carboxylate (S40): Boc-*t*Leu-Pro-OBn ester (240 mg, 0.573 mmol, 1 eq) was dissolved in DCM (2 mL). 4 M HCl in dioxane (0.57 mL, 2.3 mmol, 4 eq.) was added and the reaction was stirred at rt for 16 h. When the reaction was complete, the reaction mixture was concentrated to give the crude amine intermediate as a HCl salt.

This was redissolved in DMF (2 mL) and DIPEA (0.80 mL, 4.6 mmol, 8 eq.) added. To the reaction mixture was added 2-(dimethylamino)acetic acid (77 mg, 0.75 mmol, 1.3 eq.), followed by HATU (330 mg, 0.86 mmol, 1.5 eq.) and stirred at rt for 16 h. When the reaction was complete, the reaction mixture was diluted with water and EtOAc and stirred at rt. Aqueous layer was made basic with sat. $NaHCO_3$ and extracted with EtOAc. The organic layer was washed with brine, dried with $MgSO_4$ and evaporated to give crude product which was separated on flash chromatography (0-3% MeOH in DCM) to give product (290 mg, 130% yield) contaminated with tetramethylguanidine by-product. Used without further purification.

LCMS (Method 2) RT = 1.52 min, $[M+H]^+$ = 404.0

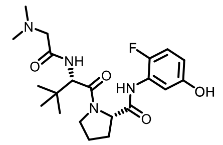

(2*S*)-1-[(2*S*)-2-[[2-(dimethylamino)acetyl]amino]-3,3-dimethyl-butanoyl]-N-(2-fluoro-5-hydroxy-phenyl)pyrrolidine-2-carboxamide (S41): benzyl (2*S*)-1-[(2*S*)-2-[[2-(dimethylamino)acetyl]amino]-3,3-dimethyl-butanoyl]pyrrolidine-2-carboxylate (**S40**) (290 mg, 0.719 mmol, 1 eq) was dissolved in Ethanol (3 mL) and degassed with N_2. 10% Pd/C (20 mg) was added to the reaction mixture, a H_2 balloon fitted over and purged with H_2. Stirred at rt for 16 h under H_2. When the reaction was complete, the reaction mixture was purged with N_2 and filtered through celite, washing with EtOH. The filtrate was concentrated to give crude intermediate.

This was redissolved in DCM (3 mL) and 3-amino-4-fluoro-phenol (180 mg, 1.4 mmol, 2 eq.) was added, followed by DCC (300 mg, 1.4 mmol, 2 eq). The reaction was stirred at rt for 1 h. When the reaction was complete, sat NH_4Cl was added and stirred overnight. This was diluted with DCM and sat. $NaHCO_3$ and filtered. The filtrate extracted and the organic layer was washed with brine, dried with $MgSO_4$ and evaporated to give crude product. Crude residue was separated on flash chromatography (0 10% MeOH in DCM) and further purified by prepLCMS. The clean fractions were combined and lyophilized to give the formate salt of the product as a white solid (51 mg, 17% yield).

^{1}H NMR (300 MHz, $CDCl_3$) δ 9.34 (d, *J* = 3.0 Hz, 1H), 7.96 (dd, *J* = 6.5, 3.0 Hz, 1H), 7.86 (d, *J* = 9.4 Hz, 1H), 6.86 (dd, *J* = 10.6, 8.8 Hz, 1H), 6.47 (dt, *J* = 9.0, 3.8 Hz, 1H), 4.85 (dd, *J* = 8.0, 3.1 Hz, 1H), 4.66 (d, *J* = 9.5

Hz, 1H), 3.99 – 3.85 (m, 1H), 3.70 (ddd, *J* = 10.0, 7.7, 4.0 Hz, 1H), 3.07 – 2.85 (m, 2H), 2.42 (dp, *J* = 12.1, 4.5 Hz, 1H), 2.29 (s, 6H), 2.21 – 1.84 (m, 3H), 1.02 (s, 9H).

LCMS (Method 2) RT = 1.03 min, $[M+H]^+$ = 423.2

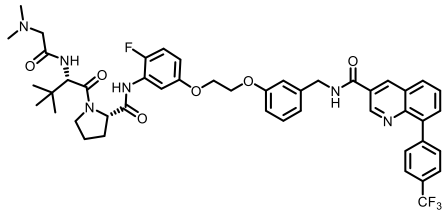

***N*-[[3-[2-[3-[[(2*S*)-1-[(2*S*)-2-[[2-(dimethylamino)acetyl]amino]-3,3-dimethyl-butanoyl]pyrrolidine-2-carbonyl]amino]-4-fluoro-phenoxy]ethoxy]phenyl]methyl]-8-[4-(trifluoromethyl)phenyl]quinoline-3-carboxamide (A559)**: (2*S*)-1-[(2*S*)-2-[[2-(dimethylamino)acetyl]amino]-3,3-dimethyl-butanoyl]-*N*-(2-fluoro-5-hydroxy-phenyl)pyrrolidine-2-carboxamide (**S41**) (51 mg, 0.12 mmol, 1 eq.) and *N*-[[3-(2-bromoethoxy)phenyl]methyl]-8-[4-(trifluoromethyl)phenyl]quinoline-3-carboxamide (63.9 mg, 0.121 mmol, 1 eq.) were dissolved in acetonitrile (1 mL). Cs_2CO_3 (120 mg, 0.36 mmol, 3 eq.) was added and the reaction was heated to 50 °C for 16 h. When the reaction was complete, solvent was removed on aspirator and azeotroped once with DCM. Redissolved resdiue in DCM and filtered. Filtrate concentrated and purified by prepLCMS. The clean fractions were combined and lyophilized to give the formate salt of the product as a white solid (57 mg, 57% yield).

^{1}H NMR (300 MHz, MeOD) δ 9.19 (d, *J* = 2.3 Hz, 1H), 8.72 (t, *J* = 2.8 Hz, 1H), 8.02 – 7.91 (m, 1H), 7.86 – 7.60 (m, 7H), 7.21 (t, *J* = 7.9 Hz, 1H), 7.04 – 6.88 (m, 3H), 6.83 (td, *J* = 8.3, 2.9 Hz, 1H), 6.59 (dt, *J* = 9.1, 3.5 Hz, 1H), 4.70 – 4.49 (m, 4H), 4.20 (dtt, *J* = 9.6, 7.4, 4.0 Hz, 4H), 3.89 (dt, *J* = 9.9, 6.3 Hz, 1H), 3.69 (dt, *J* = 10.1, 6.6 Hz, 1H), 3.02 (s, 2H), 2.31 (s, 6H), 2.25 – 1.83 (m, 4H), 0.99 (d, *J* = 11.0 Hz, 9H).

^{19}F NMR (282 MHz, MeOD) δ -63.72, -138.44 (ddd, *J* = 9.9, 6.3, 3.7 Hz).

^{13}C NMR (75 MHz, MeOD) δ 172.4, 172.4, 172.3, 171.9, 171.3, 167.7, 160.4, 160.4, 156.1, 156.1, 151.1, 149.7, 148.0, 147.4, 144.3, 144.3, 141.6, 141.5, 140.2, 137.5, 133.3, 132.3, 131.3, 131.0, 130.7, 130.7, 130.6, 130.2, 129.7, 128.8, 128.5, 128.4, 127.7, 127.7, 127.6, 125.8, 125.8, 125.7, 125.7, 124.1, 121.3, 116.5, 116.2, 115.2, 114.5, 111.9, 111.8, 111.7, 110.8, 68.5, 67.8, 63.2, 62.6, 62.0, 58.4, 58.2, 46.1, 46.0, 44.6, 37.3, 36.4, 33.4, 30.2, 26.9, 26.9, 26.2, 23.3.

LCMS (Method 2) RT = 2.36 min, $[M+H]^+$ = 871.0

HRMS (ESI+) *m/z*: $[M+H]^+$ calcd for $C_{47}H_{51}F_4N_6O_6$ 871.3806; found 871.3804.

*Synthesis of **A561** (**A538** TEAD -ve control)*

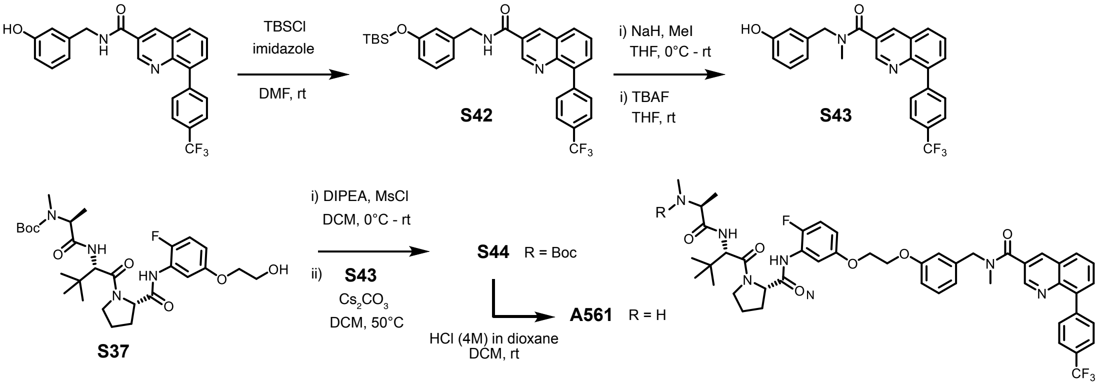

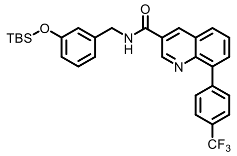

***N*-[[3-[*tert*-butyl(dimethyl)silyl]oxyphenyl]methyl]-8-[4-(trifluoromethyl)phenyl]quinoline-3-carboxamide (S42)**: *N*-[(3-hydroxyphenyl)methyl]-8-[4-(trifluoromethyl)phenyl]quinoline-3-carboxamide (250 mg, 0.59 mmol, 1 eq.) and imidazole (81 mg, 1.2 mmol, 2 eq.) were dissolved in DMF (5 mL). *tert*-butyl-chloro-dimethyl-silane (107 mg, 0.710 mmol, 1.2 eq.) added and stirred at rt overnight. Diluted with sat. NH_4Cl and Et_2O and extracted. The organic layer was washed with brine, dried with $MgSO_4$ and evaporated to give crude product. The crude residue was separated on flash chromatography (0-20% EtOAc in Heptane) to give product as a white solid (220 mg, 69% yield).

^{1}H NMR (300 MHz, $CDCl_3$) δ 9.28 (d, *J* = 2.4 Hz, 1H), 8.67 (d, *J* = 2.3 Hz, 1H), 7.97 (dd, *J* = 8.2, 1.7 Hz, 1H), 7.87 – 7.65 (m, 6H), 7.22 (d, *J* = 7.9 Hz, 1H), 6.98 (d, *J* = 7.6 Hz, 1H), 6.87 (t, *J* = 2.2 Hz, 1H), 6.84 – 6.75 (m, 1H), 6.49 (s, 1H), 4.67 (d, *J* = 5.7 Hz, 2H), 0.98 (s, 9H), 0.20 (s, 6H).

LCMS (Method 2) RT = 3.26 min, $[M+H]^+$ = 537.0

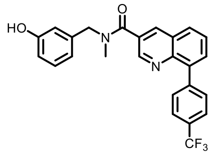

***N*-[(3-hydroxyphenyl)methyl]-*N*-methyl-8-[4-(trifluoromethyl)phenyl]quinoline-3-carboxamide (S43)**: *N*-[[3-[*tert*-butyl(dimethyl)silyl]oxyphenyl]methyl]-8-[4-(trifluoromethyl)phenyl]quinoline-3-carboxamide (**S42**) (220 mg, 0.41 mmol, 1 eq.) was dissolved in dry THF (4 mL) and cooled to 0 °C under N_2. iodomethane (0.051 mL, 0.82 mmol, 2 eq.) was added, followed by Sodium Hydride (33 mg, 60% purity, 0.82 mmol, 2 eq.) and the reaction was slowly warmed to rt.

Quenched with sat. NH_4Cl and extracted with EtOAc. The organic layer was washed with brine, dried with $MgSO_4$ and evaporated to give crude intermediate *N*-[[3-[*tert*-butyl(dimethyl)silyl]oxyphenyl]methyl]-N-methyl-8-[4-(trifluoromethyl)phenyl]quinoline-3-carboxamide.

The intermediate was dissolved THF (4 mL) and TBAF (0.82 mL, 0.82 mmol, 2 eq.) added. The reaction was stirred at rt for 1 h. It was then diluted with EtOAc and water and extracted. The organic layer was washed with brine, dried with $MgSO_4$ and evaporated to give crude product which was separated on flash chromatography (0- 50% EtOAc in Heptane) to give the product as a white oil. NMR showed EtOAc and Heptane impurities as well as 1:1 rotamer splitting (210mg, 93% yield). Used without further purification.

^{1}H NMR (300 MHz, $CDCl_3$) δ 9.01 (d, *J* = 2.3 Hz, 1H), 8.34 (s, 1H), 8.08 (s, 1H), 7.88 – 7.53 (m, 6H), 7.12 (dt, *J* = 7.8, 4.6 Hz, 1H), 6.96 (s, 0.5H), 6.88 – 6.53 (m, 2.5H), 4.75 (s, 1H), 4.49 (s, 1H), 3.12 (s, 1.5H), 2.93 (s, 1.5H). 1:1 rotamer splitting

LCMS (Method 2) RT = 2.45 min, $[M+H]^+$ = 437.0

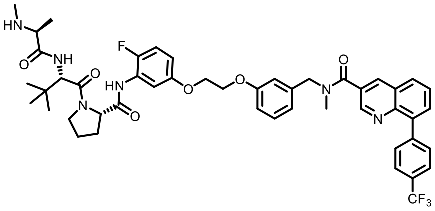

***N*-[[3-[2-[3-[[(2*S*)-1-[(2*S*)-3,3-dimethyl-2-[[(2*S*)-2-(methylamino)propanoyl]amino]butanoyl]pyrrolidine-2-carbonyl]amino]-4-fluoro-phenoxy]ethoxy]phenyl]methyl]-*N*-methyl-8-[4-(trifluoromethyl)phenyl]quinoline-3-carboxamide (A561)**: *tert*-butyl *N*-[(1*S*)-2-[[(1*S*)-1-[(2*S*)-2-[[2-fluoro-5-(2-hydroxyethoxy)phenyl]carbamoyl]pyrrolidine-1-carbonyl]-2,2-dimethyl-propyl]amino]-1-methyl-2-oxo-ethyl]-*N*-methyl-carbamate (**S37**) (68 mg, 0.072 mmol, 1 eq.) was dissolved in DCM (3 mL) and cooled on ice. DIPEA (0.038 mL, 0.22 mmol, 3 eq.) was added, followed by methanesulfonyl chloride (0.017 mL, 0.22 mmol, 3 eq.). The reaction was slowly warmed to rt for 1 h. Sat. $NaHCO_3$ was added to quench the reaction and the mixture was extracted with DCM. The organic layer was washed with brine, dried with $MgSO_4$ and evaporated to give crude mesylate intermediate.

This was dissolved in DCM and *N*-[(3-hydroxyphenyl)methyl]-*N*-methyl-8-[4-(trifluoromethyl)phenyl]quinoline-3-carboxamide (**S43**) (31 mg, 0.072 mmol, 1 eq.) and Cs_2CO_3 (70 mg, 0.22 mmol, 3 eq.) added. The reaction was heated to 50 °C overnight. After this time, the solvent was removed under N_2 flow and redissolved in DCM and filtered. The filtrate concentrated and separated on flash chromatography (0-10% MeOH in DCM) to give Boc -protected product **S44** (23 mg, 32%).

This was dissolved in DCM (2 mL) and HCl in dioxane (0.058 mL, 0.23 mmol) added. The reaction was stirred at rt for 10 min. MeOH added to make the reaction mixture homogenous and solvents were removed under N_2 flow. The resulting residue was purified by prepLCMS. The clean fractions was collected and lyophilized to give the formate salt of the product as a white solid (16 mg, 73% yield from Boc-protected intermediate).

^{1}H NMR (300 MHz, MeOD) δ 8.90 (d, *J* = 15.4 Hz, 1H), 8.58 – 8.40 (m, 1H), 8.00 (dd, *J* = 33.7, 7.8 Hz, 1H), 7.79 (dt, *J* = 17.4, 8.8 Hz, 7H), 7.30 (d, *J* = 7.6 Hz, 1H), 7.12 – 6.59 (m, 5H), 4.80 (s, 1H), 4.70 – 4.51 (m, 3H), 4.41 – 4.15 (m, 4H), 3.93 (dt, *J* = 11.9, 6.4 Hz, 1H), 3.73 (q, *J* = 6.5 Hz, 1H), 3.25 (q, *J* = 6.9 Hz, 1H),

3.14 (s, 1.5H), 3.01 (s, 1.5H), 2.34 (s, 3H), 2.32 – 1.85 (m, 4H), 1.26 (d, *J* = 6.9 Hz, 3H), 1.02 (d, *J* = 13.6 Hz, 9H). 1:1 rotamer splitting

^{13}C NMR (126 MHz, MeOD) δ 176.4, 172.5, 172.3, 172.0, 171.7, 171.0, 170.2, 160.7, 156.2, 150.6, 149.4, 149.1, 148.7, 146.6, 144.4, 140.4, 139.6, 136.8, 136.4, 133.0, 132.3, 131.3, 131.0, 130.6, 130.5, 130.3, 130.2, 130.1, 129.2, 128.9, 128.6, 127.8, 127.7, 127.0, 125.9, 125.8, 125.8, 125.8, 124.8, 122.7, 121.8, 120.4, 116.5, 116.3, 116.2, 115.6, 115.0, 114.4, 111.8, 110.8, 68.6, 67.9, 62.7, 62.1, 60.5, 60.2, 58.8, 58.5, 56.3, 52.0, 49.9, 47.9, 37.8, 37.2, 36.2, 34.5, 34.3, 33.4, 30.3, 27.0, 26.9, 26.8, 26.2, 23.3, 19.2, 19.0.

^{19}F NMR (282 MHz, MeOD) δ -63.85, -138.61.

LCMS (Method 2) RT = 2.38 min, $[M+H]^+$ = 884.9

HRMS (ESI+) *m/z*: $[M+H]^+$ calcd for $C_{48}H_{53}F_4N_6O_6$ 885.3963; found 885.3937.

*Synthesis of **A536***

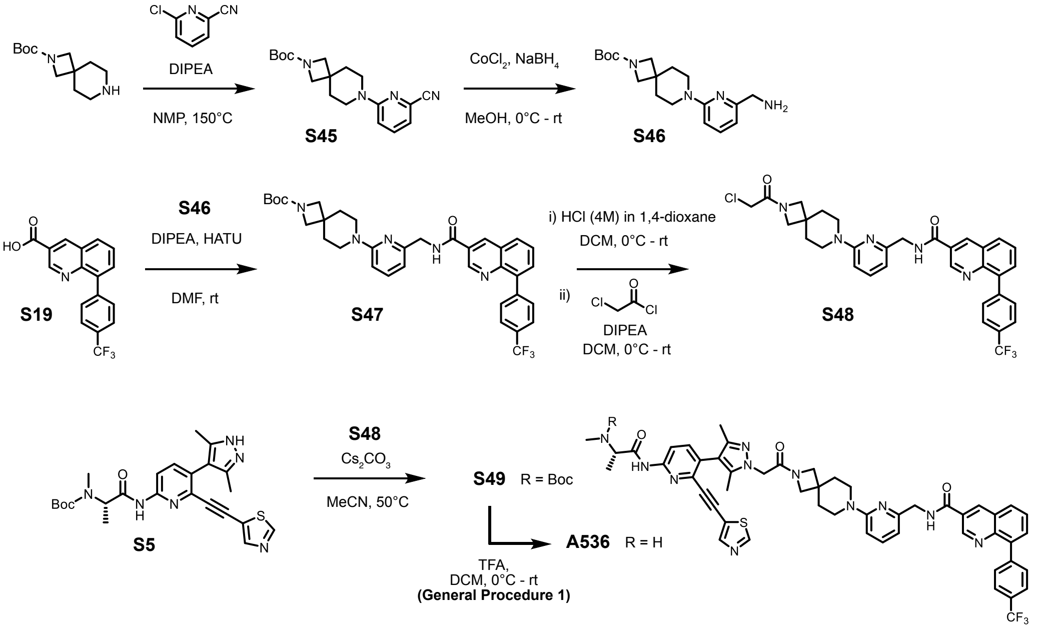

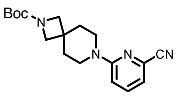

***tert*-butyl 7-(6-cyano-2-pyridyl)-2,7-diazaspiro[3.5]nonane-2-carboxylate (S45)**: 6-chloropyridine-2-carbonitrile (150 mg, 1.08 mmol, 1 eq.) and *tert*-butyl 2,7-diazaspiro[3.5]nonane-2-carboxylate (250 mg, 1.1 mmol, 1 eq.) was dissolved in NMP (3 mL). DIPEA (0.57 mL, 3.2 mmol, 3 eq.) was added and the reaction was heated to 150 °C in a microwave reactor for 1 h. After the reaction had stopped progressing (as monitored by LCMS), it was diluted with EtOAc, then washed (x3) with cold brine. The organic layer was dried *in vacuo* and subjected to column chromatography using a gradient of 0-60% EtOAc in heptane to give product as a white solid (250 mg, 70% yield)

^{1}H NMR (300 MHz, $CDCl_3$) δ 7.49 (dd, *J* = 8.9, 7.2 Hz, 1H), 6.94 (d, *J* = 7.2 Hz, 1H), 6.83 (d, *J* = 8.8 Hz, 1H), 3.69 (s, 4H), 3.57 – 3.52 (m, 4H), 1.84 – 1.75 (m, 4H), 1.45 (s, 9H).

LCMS (Method 2) RT = 2.44, $[M+H]^+$ = 329.3

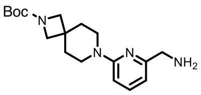

***tert*-butyl 7-[6-(aminomethyl)-2-pyridyl]-2,7-diazaspiro[3.5]nonane-2-carboxylate (S46)**: *tert*-butyl 7-(6-cyano-2-pyridyl)-2,7-diazaspiro[3.5]nonane-2-carboxylate (**S45**) (85 mg, 0.26 mmol, 1 eq.) was dissolved in MeOH (3 mL) and cooled to 0 °C . $CoCl_2$ (200 mg, 1.6 mmol, 5 eq.) added, followed by portionwise addition of $NaBH_4$ (29 mg, 0.78 mmol, 3 eq.). The reaction was slowly warmed to rt. After 30 min, the reaction was quenched with sat. NH_4Cl, diluted with EtOAc and made basic with sat $NaHCO_3$. The mixture was filtered through Celite, and filtrate extracted with EtOAc. The organic layer was washed with brine, dried with $MgSO_4$ and evaporated to give crude product. This was separated on flash chromatography (0-10% MeOH in DCM) to give the product as a yellow/brown oil (52 mg, 60% yield).

^{1}H NMR (300 MHz, $CDCl_3$) δ 7.41 (t, *J* = 8.0 Hz, 1H), 6.54 (d, *J* = 8.3 Hz, 2H), 4.04 (bs, 2H), 3.67 (s, 4H), 3.49 (t, *J* = 5.6 Hz, 4H), 2.16 (bs, 2H), 1.79 (t, *J* = 5.7 Hz, 4H), 1.45 (s, 9H).

LCMS (Method 2) RT = 1.47 min, $[M+H]^+$ = 333.4

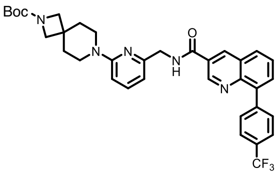

***tert*-butyl 7-[6-[[[8-[4-(trifluoromethyl)phenyl]quinoline-3-carbonyl]amino]methyl]-2-pyridyl]-2,7-diazaspiro[3.5]nonane-2-carboxylate (S47)**: 8-[4-(trifluoromethyl)phenyl]quinoline-3-carboxylic acid (**S19**) (55 mg, 0.17 mmol, 1 eq.) and *tert*-butyl 7-[6-(aminomethyl)-2-pyridyl]-2,7-diazaspiro[3.5]nonane-2-carboxylate (**S46**) (52 mg, 0.16 mmol, 1 eq.) was dissolved in DMF (1 mL). DIPEA (0.082 mL, 0.47 mmol, 3 eq.) was added, followed by HATU (77 mg, 0.20 mmol, 1.3 eq.) and stirred at rt. After 10 min, the reaction was diluted with EtOAc and water. 10% citric acid was added until pH = 3 and extracted with EtOAc. The organic layer was washed with $NaHCO_3$, then brine, dried with $MgSO_4$ and evaporated to give crude product which was separated on flash chromatography (0-100% EtOAc in Heptane) to give the product as a white solid (75 mg, 76% yield).

^{1}H NMR (300 MHz, $CDCl_3$) δ 9.34 (d, *J* = 2.3 Hz, 1H), 8.75 (d, *J* = 2.3 Hz, 1H), 7.98 (dd, *J* = 8.1, 1.5 Hz, 1H), 7.88 – 7.67 (m, 6H), 7.65 (s, 1H), 7.54 – 7.36 (m, 1H), 6.71 – 6.53 (m, 2H), 4.69 (d, *J* = 4.5 Hz, 2H), 3.69 (s, 4H), 3.55 (t, *J* = 5.7 Hz, 4H), 1.83 (t, *J* = 5.6 Hz, 4H), 1.45 (s, 9H).

LCMS (Method 2) RT = 2.50 min, $[M+H]^+$ = 632.4

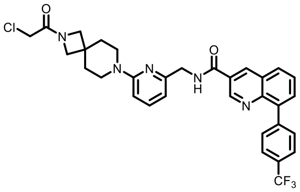

N-[[6-[2-(2-chloroacetyl)-2,7-diazaspiro[3.5]nonan-7-yl]-2-pyridyl]methyl]-8-[4-(trifluoromethyl)phenyl]quinoline-3-carboxamide (S48): To a solution of *tert*-butyl 7-[6-[[[8-[4-(trifluoromethyl)phenyl]quinoline-3-carbonyl]amino]methyl]-2-pyridyl]-2,7-diazaspiro[3.5]nonane-2-carboxylate (**S47**) (250 mg, 0.40 mmol, 1 eq.) in DCM (5 mL) at 0 °C was added 4 M HCl in 1,4-dioxane (1.0 mL) and the mixture was left to stir at rt over the weekend. The mixture was dried *in vacuo* then azeotroped with toluene to yield *N*-[[6-(7-aza-2-azoniaspiro[3.5]nonan-7-yl)-2-pyridyl]methyl]-8-[4-(trifluoromethyl)phenyl]quinoline-3-carboxamide chloride (290 mg, 100% yield) as a yellow solid.

N-[[6-(7-aza-2-azoniaspiro[3.5]nonan-7-yl)-2-pyridyl]methyl]-8-[4-(trifluoromethyl)phenyl]quinoline-3-carboxamide chloride (120 mg, 0.169 mmol, 1 eq.) was dissolved in DCM (3 mL) and cooled to 0 °C, then treated with 2-chloroacetyl chloride (20 µL, 0.25 mmol, 1.5 eq.), and then DIPEA (120 µL, 0.68 mmol, 4 eq.) in DCM (1 mL). The reaction was warmed to rt and stirred for 1 h. The reaction was then diluted with brine and DCM. The organic layer was collected and the aqueous layer was extracted twice more with DCM. The combined organic layers were dried over $MgSO_4$ then concentrated under reduced pressure. The crude product was purified by Combi-Flash on a silica column eluting with 0-5% MeOH in DCM to yield a yellow solid (100 mg, 97% yield from amine HCl salt).

^{1}H NMR (300 MHz, $CDCl_3$) δ 9.33 (d, *J* = 2.3 Hz, 1H), 8.76 (d, *J* = 2.3 Hz, 1H), 7.98 (dt, *J* = 8.2, 2.6 Hz, 1H), 7.87 – 7.66 (m, 6H), 7.71 – 7.58 (m, 1H), 7.49 (dd, *J* = 8.5, 7.3 Hz, 1H), 6.74 – 6.53 (m, 2H), 4.69 (d, *J* = 4.6 Hz, 2H), 4.02 (s, 2H), 3.91 (s, 2H), 3.83 (s, 2H), 3.55 (dd, *J* = 9.7, 4.5 Hz, 4H), 1.86 (t, *J* = 5.6 Hz, 4H).

LCMS (Method 2) RT = 2.14 min, $[M+H]^+$ = 608.3/610.2

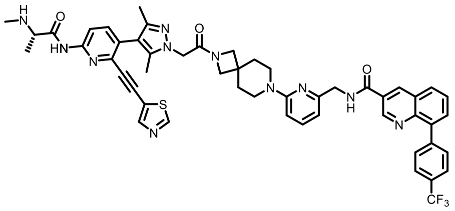

N-[[6-[2-[2-[3,5-dimethyl-4-[6-[[(2S)-2-(methylamino)propanoyl]amino]-2-(2-thiazol-5-ylethynyl)-3-pyridyl]pyrazol-1-yl]acetyl]-2,7-diazaspiro[3.5]nonan-7-yl]-2-pyridyl]methyl]-8-[4-(trifluoromethyl)phenyl]quinoline-3-carboxamide (A536): *N*-[[6-[2-(2-chloroacetyl)-2,7-diazaspiro[3.5]nonan-7-yl]-2-pyridyl]methyl]-8-[4-(trifluoromethyl)phenyl]quinoline-3-carboxamide (**S48**) (22 mg, 0.036 mmol), *tert*-butyl ((2*S*)-1-((5-(3,5-dimethyl-1*H*-pyrazol-4-yl)-6-(thiazol-5-ylethynyl)pyridin-2-yl)amino)-1-oxopropan-2-yl)(methyl)carbamate (**S5**) (19 mg, 0.040 mmol), and Cs_2CO_3 (35 mg, 0.11 mmol) was dissolved in acetonitrile (1 mL) and heated to 50 °C overnight. After this time, the solvent was removed under N_2 flow and the residue redissolved in DCM and filtered. The filtrate was concentrated and separated on flash chromatography (0-3% MeOH in DCM) to give boc-protected intermediate *tert*-butyl *N*-[(1*S*)-2-[[5-[3,5-dimethyl-1-[2-oxo-2-[7-[6-[[[8-[4-(trifluoromethyl)phenyl]quinoline-3-

carbonyl]amino]methyl]-2-pyridyl]-2,7-diazaspiro[3.5]nonan-2-yl]ethyl]pyrazol-4-yl]-6-(2-thiazol-5-ylethynyl)-2-pyridyl]amino]-1-methyl-2-oxo-ethyl]-*N*-methyl-carbamate (**S49**) (36 mg, 0.034 mmol) as a yellow oil.

This was dissolved in DCM (1.5 mL) and TFA (0.15 mL) added at 0 °C. The reaction was stirred at rt for 3 h. After this, the solvents were removed under N_2 flow and the residue was purified by prepLCMS. The clean fractions were combined and lyophilized to give the formate salt of the product **A536** as a white solid. (36 mg, 60% yield over 2 steps).

^{1}H NMR (300 MHz, MeOD) δ 9.26 (d, *J* = 2.3 Hz, 1H), 8.93 (s, 1H), 8.84 (d, *J* = 2.3 Hz, 1H), 8.23 (d, *J* = 8.6 Hz, 1H), 8.11 – 7.99 (m, 2H), 7.92 – 7.69 (m, 7H), 7.53 – 7.42 (m, 1H), 6.66 (dd, *J* = 7.9, 4.3 Hz, 2H), 4.83 (s, 2H), 4.60 (s, 2H), 3.93 (q, *J* = 8.8 Hz, 2H), 3.76 (s, 3H), 3.51 (p, *J* = 2.8 Hz, 4H), 2.62 (s, 3H), 2.17 (s, 3H), 2.14 (s, 3H), 1.77 – 1.65 (m, 4H), 1.54 (d, *J* = 7.0 Hz, 3H).

^{19}F NMR (282 MHz, MeOD) δ -63.78

^{13}C NMR (75 MHz, MeOD) δ 172.1, 168.8, 168.0, 160.3, 157.1, 156.9, 151.7, 149.8, 149.1, 148.0, 147.5, 144.4, 142.3, 141.5, 141.1, 140.4, 139.5, 137.6, 133.4, 132.3, 130.7, 130.7, 130.2, 130.0, 128.9, 128.7, 128.6, 127.7, 125.9, 125.8, 124.1, 119.3, 116.8, 115.6, 111.1, 106.9, 96.0, 81.5, 61.0, 59.9, 59.3, 46.2, 43.4, 35.8, 35.5, 33.0, 17.4, 12.5, 10.5.

LCMS (Method 2) RT = 1.91 min, $[M+2H]/2^{+}$ = 476.8

HRMS (ESI+) *m/z*: $[M+H]^{+}$ calcd for $C_{51}H_{49}F_3N_{11}O_3S$ 952.3693; found 952.3655.

*Synthesis of **A558** (**A536** IAP -ve control)*

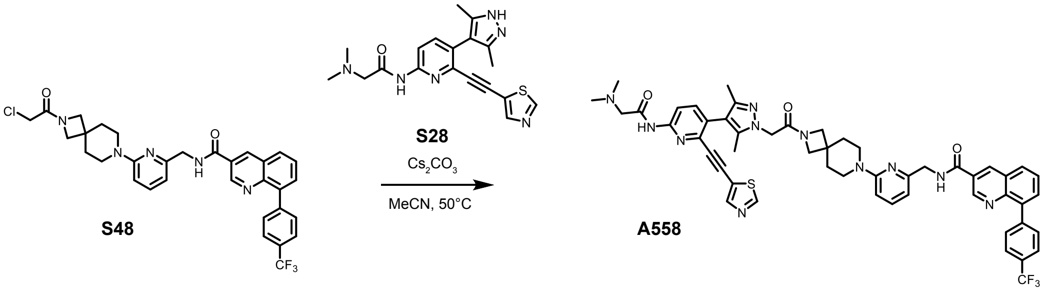

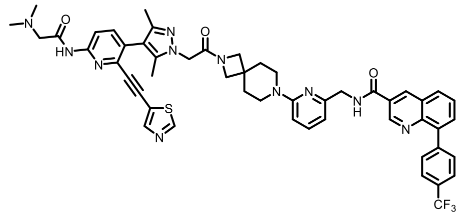

***N*-[[6-[2-[2-[4-[6-[[2-(dimethylamino)acetyl]amino]-2-(2-thiazol-5-ylethynyl)-3-pyridyl]-3,5-dimethyl-pyrazol-1-yl]acetyl]-2,7-diazaspiro[3.5]nonan-7-yl]-2-pyridyl]methyl]-8-[4-(trifluoromethyl)phenyl]quinoline-3-carboxamide (A558)**: *N*-[[6-[2-(2-chloroacetyl)-2,7-diazaspiro[3.5]nonan-7-yl]-2-pyridyl]methyl]-8-[4-(trifluoromethyl)phenyl]quinoline-3-carboxamide (**S48**)

(22 mg, 0.036 mmol, 1 eq.), *N*-(5-(3,5-dimethyl-1*H*-pyrazol-4-yl)-6-(thiazol-5-ylethynyl)pyridin-2-yl)-2-(dimethylamino)acetamide (**S28**) (15 mg, 0.040 mmol, 1.1 eq.), and Cs_2CO_3 (35 mg, 0.11 mmol, 3 eq.) was dissolved in acetonitrile (1 mL) and heated to 50 °C overnight. After this time, the solvent was removed under N_2 flow and redissolved in DCM. This was filtered and the filtrate concentrated, and the residue was purified by prepLCMS. The clean fractions were combined and lyophilized to give the formate salt of the product as a white solid (24 mg, 66% yield).

^{1}H NMR (300 MHz, MeOD) δ 9.26 (d, *J* = 2.3 Hz, 1H), 8.92 (s, 1H), 8.84 (d, *J* = 2.4 Hz, 1H), 8.25 (d, *J* = 8.5 Hz, 1H), 8.11 – 7.98 (m, 2H), 7.91 – 7.67 (m, 7H), 7.48 (dd, *J* = 8.5, 7.3 Hz, 1H), 6.66 (dd, *J* = 7.9, 4.0 Hz, 2H), 4.83 (s, 2H), 4.60 (s, 2H), 3.91 (q, *J* = 8.5 Hz, 2H), 3.76 (s, 2H), 3.51 (q, *J* = 6.0 Hz, 4H), 3.22 (s, 2H), 2.42 (s, 6H), 2.17 (s, 3H), 2.14 (s, 3H), 1.72 (dd, *J* = 7.9, 4.1 Hz, 4H).

^{19}F NMR (282 MHz, MeOD) δ -63.78.

^{13}C NMR (75 MHz, MeOD) δ 171.5, 168.8, 168.0, 160.3, 157.2, 156.9, 151.7, 149.7, 149.1, 148.0, 147.6, 144.4, 142.5, 141.3, 141.1, 140.4, 139.5, 137.6, 133.4, 132.3, 130.7, 130.7, 130.3, 129.7, 128.9, 128.7, 128.6, 127.7, 125.9, 125.8, 125.7, 124.1, 119.2, 116.8, 115.3, 111.1, 106.9, 95.9, 81.6, 63.9, 61.0, 59.3, 50.0, 46.2, 46.0, 45.9, 43.5, 35.8, 35.5, 12.5, 10.5.

LCMS (Method 2) RT = 1.93 min, $[M+2H]/2^+$ = 476.6

HRMS (ESI+) *m/z*: $[M+H]^+$ calcd for $C_{51}H_{49}F_3N_{11}O_3S$ 952.3693; found 952.3688.

*Synthesis of **A560** (**A536** TEAD -ve control)*

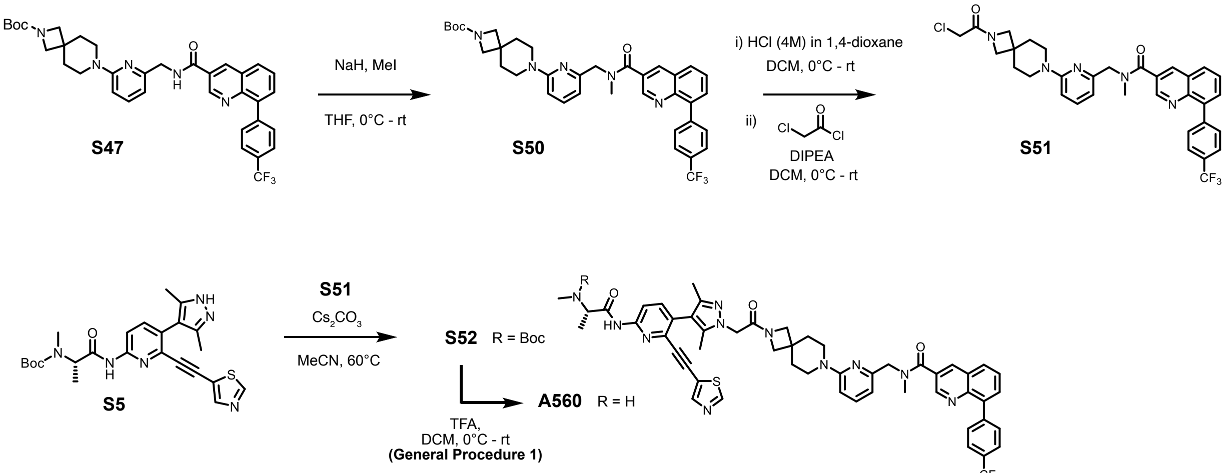

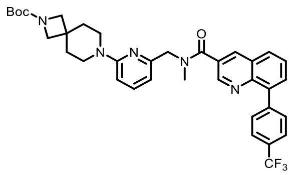

tert-butyl 7-[6-[[methyl-[8-[4-(trifluoromethyl)phenyl]quinoline-3-carbonyl]amino]methyl]-2-pyridyl]-2,7-diazaspiro[3.5]nonane-2-carboxylate (S50): *tert*-butyl 7-[6-[[[8-[4-(trifluoromethyl)phenyl]quinoline-3-carbonyl]amino]methyl]-2-pyridyl]-2,7-diazaspiro[3.5]nonane-2-carboxylate (**S47**) (75 mg, 0.12 mmol,

1 eq.) dissolved in dry THF (2 mL) and cooled to 0 °C under N_2. Iodomethane (0.015 mL, 0.24 mmol, 2 eq.) was added to the reaction mixture, followed by Sodium Hydride (9.5 mg 60% purity, 0.24 mmol, 2 eq.). The reaction was slowly warmed to rt and stirred for 2 h. After this, it was quenched with sat. NH_4Cl and extracted between EtOAc and water. The organic layer was washed with brine, dried with $MgSO_4$ and evaporated to give crude product that was separated on flash chromatography (0-30% EtOAc in Heptane) to give product (61 mg, 80% yield).

^{1}H NMR (300 MHz, $CDCl_3$) δ 9.10 – 9.01 (m, 1H), 8.49 – 8.34 (m, 1H), 7.78 (dd, *J* = 14.2, 5.3 Hz, 5H), 7.75 – 7.59 (m, 2H), 7.43 (dd, *J* = 9.6, 6.0 Hz, 1H), 6.67 (d, J = 7.2 Hz, 0.4H), 6.57 (d, J = 8.6 Hz, 1H), 6.43 (d, *J* = 7.3 Hz, 0.6H), 4.75 (d, *J* = 7.4 Hz, 0.8H), 4.48 (s, 1.2H), 3.68 (s, 4H), 3.52 (t, *J* = 5.6 Hz, 4H), 3.13 (d, *J* = 6.2 Hz, 3H), 1.79 (t, *J* = 5.7 Hz, 4H), 1.45 (s, 9H). 4:6 rotamer splitting

LCMS (Method 2) RT = 2.74 min, $[M+H]^+$ = 646.0

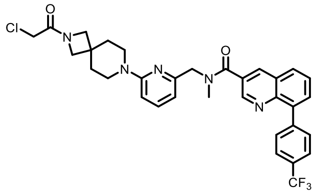

***N*-[[6-[2-(2-chloroacetyl)-2,7-diazaspiro[3.5]nonan-7-yl]-2-pyridyl]methyl]-N-methyl-8-[4-(trifluoromethyl)phenyl]quinoline-3-carboxamide (S51)**: *tert*-butyl 7-[6-[[methyl-[8-[4-(trifluoromethyl)phenyl]quinoline-3-carbonyl]amino]methyl]-2-pyridyl]-2,7-diazaspiro[3.5]nonane-2-carboxylate (**S50**) (61 mg, 0.094 mmol, 1 eq.) was dissolved in DCM (2 mL) at 0°C, then 4 M HCl in 1,4-dioxane (0.07 mL, 0.283 mmol, 3 eq.) was added and the reaction allowed to warm to rt and stirred for 2 h.

After this, the solvent was removed under N_2 flow and residue was redissolved in DCM (2 mL) and DIPEA (0.082 mL, 0.47 mmol, 5 eq.) was added. The reaction was cooled to 0 °C and 2-chloroacetyl chloride (0.0098 mL, 0.12 mmol, 1.3 eq.) added. The reaction was warmed to rt and stirred for 10 min. Sat. $NaHCO_3$ was added to quench and the mixture was extracted with DCM. The organic layer was washed with brine, dried with $MgSO_4$ and evaporated to give crude product that was separated on flash chromatography (0-50-100% EtOAc in Heptane) to give product (50 mg, 85% yield)

^{1}H NMR (300 MHz, $CDCl_3$) δ 9.14 – 9.01 (m, 1H), 8.48 – 8.34 (m, 1H), 7.96 – 7.39 (m, 8H), 6.74 – 6.41 (m, 2H), 4.77 (s, 0.6H), 4.49 (s, 1.4H), 4.01 (s, 2H), 3.91 (s, 2H), 3.82 (s, 2H), 3.55 (d, *J* = 5.4 Hz, 4H), 3.13 (d, *J* = 5.5 Hz, 3H), 1.84 (d, *J* = 5.5 Hz, 4H). 3:7 rotamer splitting

LCMS (Method 2) RT = 2.36 min, $[M+H]^+$ = 622.0/624.0

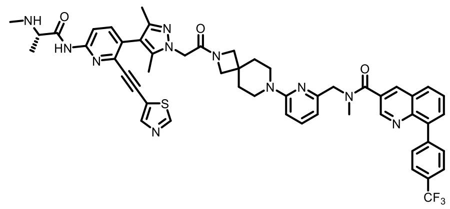

N-[[6-[2-[2-[3,5-dimethyl-4-[6-[[(2S)-2-(methylamino)propanoyl]amino]-2-(2-thiazol-5-ylethynyl)-3-pyridyl]pyrazol-1-yl]acetyl]-2,7-diazaspiro[3.5]nonan-7-yl]-2-pyridyl]methyl]-N-methyl-8-[4-(trifluoromethyl)phenyl]quinoline-3-carboxamide (A560): *N*-[[6-[2-(2-chloroacetyl)-2,7-diazaspiro[3.5]nonan-7-yl]-2-pyridyl]methyl]-*N*-methyl-8-[4-(trifluoromethyl)phenyl]quinoline-3-carboxamide (**S51**) (50 mg, 0.080 mmol, 1 eq.) and *tert*-butyl ((2*S*)-1-((5-(3,5-dimethyl-1*H*-pyrazol-4-yl)-6-(thiazol-5-ylethynyl)pyridin-2-yl)amino)-1-oxopropan-2-yl)(methyl)carbamate (**S5**) (38.6 mg, 0.080 mmol, 1 eq.) was dissolved in acetonitrile (1.5 mL). Cs_2CO_3 (79 mg, 0.24 mmol, 1 eq.) was added and the reaction was heated at 60 °C overnight. Solvent was removed under N_2 flow and redissolved in DCM and filtered. The filtrate was concentrated and crude residue separated on flash chromatography (0-10% MeOH in DCM) to give Boc-protected intermediate *tert*-butyl *N*-[(1*S*)-2-[[5-[3,5-dimethyl-1-[2-[7-[6-[[methyl-[8-[4-(trifluoromethyl)phenyl]quinoline-3-carbonyl]amino]methyl]-2-pyridyl]-2,7-diazaspiro[3.5]nonan-2-yl]-2-oxo-ethyl]pyrazol-4-yl]-6-(2-thiazol-5-ylethynyl)-2-pyridyl]amino]-1-methyl-2-oxo-ethyl]-*N*-methyl-carbamate (**S52**) (60 mg).

This was dissolved in DCM (2 mL), TFA (0.20 mL) added at 0°C, and allowed to warm and stir at rt for 20 min. The solvents were removed under N_2 flow, and the residue was purified by prepLCMS. The clean fractions were combined and lyophilized to give the formate salt of the product **A560** as a white solid (32 mg, 39% yield over 2 steps).

^{1}H NMR (300 MHz, MeOD) δ 9.00 – 8.89 (m, 2H), 8.53 (d, *J* = 2.3 Hz, 1H), 8.24 (d, *J* = 8.5 Hz, 1H), 8.09 – 7.90 (m, 2H), 7.88 – 7.64 (m, 8H), 7.48 (dt, *J* = 20.6, 7.6 Hz, 1H), 6.68 (dd, *J* = 8.3, 3.9 Hz, 1.3H), 6.47 (d, *J* = 7.3 Hz, 0.7H), 4.84 (s, 2H), 4.75 (s, 0.7H), 4.52 (s, 1.3H), 3.94 (q, *J* = 8.4 Hz, 2H), 3.78 (s, 2H), 3.53 (q, *J* = 8.0 Hz, 5H), 3.12 (d, *J* = 8.3 Hz, 3H), 2.50 (s, 3H), 2.16 (d, *J* = 10.6 Hz, 6H), 1.74 (d, *J* = 5.8 Hz, 4H), 1.44 (d, *J* = 6.9 Hz, 3H). 4:6 rotamer splitting

^{19}F NMR (282 MHz, MeOD) δ -63.69, -63.72.

^{13}C NMR (75 MHz, MeOD) δ 174.2, 172.2, 171.0, 168.8, 160.5, 157.1, 155.4, 154.9, 151.8, 149.6, 149.4, 149.1, 148.0, 146.5, 144.4, 142.3, 141.4, 141.1, 140.4, 139.6, 136.6, 132.9, 132.3, 131.3, 130.9, 130.6, 130.2, 129.9, 128.8, 128.5, 127.7, 125.8, 125.8, 124.1, 119.3, 116.8, 115.5, 111.8, 107.4, 107.0, 96.0, 81.5, 61.0, 60.5, 59.3, 57.6, 53.7, 49.9, 43.3, 39.0, 35.8, 35.6, 34.6, 33.8, 18.2, 12.5, 10.6.

LCMS (Method 2) RT = 2.05 min, $[M+2H]/2^+$ = 483.8

HRMS (ESI+) *m/z*: $[M+H]^+$ calcd for $C_{52}H_{51}F_3N_{11}O_3S$ 966.3849; found 966.3809.

Synthesis of ***A232***

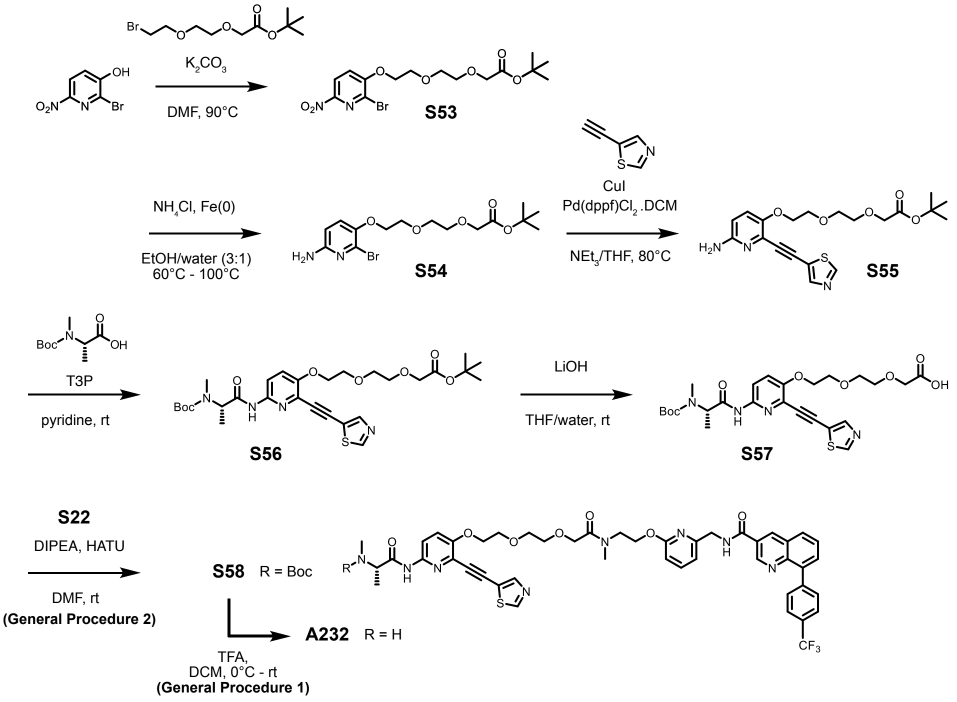

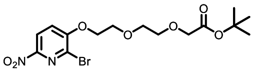

***tert*-butyl 2-[2-[2-[(2-bromo-6-nitro-3-pyridyl)oxy]ethoxy]ethoxy]acetate (S53):** *tert*-butyl 2-[2-(2-bromoethoxy)ethoxy]acetate (2100 mg, 7.5 mmol, 1.1 eq.) was dissolved in DMF (45 mL) and treated with 2-bromo-6-nitro-pyridin-3-ol (1500 mg, 6.8 mmol, 1 eq.) and K_2CO_3 (1890 mg, 13.7 mmol). The yellow mixture was stirred at 90 °C overnight. Once the reaction was complete (monitored by LCMS), the reaction mixture was cooled to rt and diluted with EtOAc and brine. The organic layer was collected and washed five times with chilled brine, dried ($MgSO_4$) then concentrated under reduced pressure. The crude residue was separated using column chromatography (Combi-Flash) with EtOAc:Hept (0-60%) to obtain the title product as a yellow oil (1390 mg, 48% yield).

^{1}H NMR (300 MHz, $CDCl_3$) δ 8.25 (dd, *J* = 8.7, 1.5 Hz, 1H), 7.49 (dd, *J* = 8.7, 1.6 Hz, 1H), 4.46 – 4.34 (m, 2H), 4.04 – 3.94 (m, 4H), 3.83 – 3.66 (m, 4H), 1.47 (d, *J* = 1.2 Hz, 9H).

LCMS (Method 2) RT = 2.34, $[M+H - tBu]^+$ = 365.0/367.0

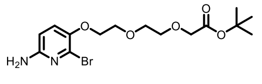

***tert*-butyl 2-[2-[2-[(6-amino-2-bromo-3-pyridyl)oxy]ethoxy]ethoxy]acetate (S54):** *tert*-butyl 2-[2-[2-[(2-bromo-6-nitro-3-pyridyl)oxy]ethoxy]ethoxy]acetate (**S53**) (1380 mg, 2.46 mmol, 1 eq.) was taken up in EtOH (30 mL) and Water (10 mL) then treated with Ammonium Chloride (590 mg, 11 mmol, 4.5 eq.). The mixture was heated to 60 °C, with small additions of water until all the solid was dissolved. The solution was then treated with Iron (440 mg, 7.9 mmol, 3.2 eq.) before being heated to 100 °C. Once the reaction was complete (monitored by LCMS), the reaction was cooled to rt, diluted with EtOAc and sat.

$NaHCO_3$. The organic layer was washed twice with brine, dried (MgSO4) then concentrated under reduced pressure to a yellow oil. The crude residue was separated using column chromatography (Combi-Flash) eluting with EtOAc:Hept (0-75%) to obtain the title product as a colorless oil (1100 mg, 94% yield, 80% purity).

^{1}H NMR (300 MHz, $CDCl_3$) δ 7.15 (d, *J* = 8.5 Hz, 1H), 6.40 (d, *J* = 8.5 Hz, 1H), 4.23 (bs, 2H), 4.14 – 4.04 (m, 2H), 4.02 (s, 2H), 3.89 – 3.80 (m, 2H), 3.80 – 3.67 (m, 4H), 1.45 (s, 9H).

LCMS (Method 2) RT = 2.00, $[M+H]^+$ = 391.2/393.2

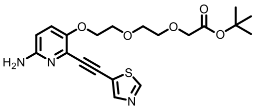

***tert*-butyl 2-[2-[2-[[6-amino-2-(2-thiazol-5-ylethynyl)-3-pyridyl]oxy]ethoxy]ethoxy]acetate (S55):** *tert*-butyl 2-[2-[2-[(6-amino-2-bromo-3-pyridyl)oxy]ethoxy]ethoxy]acetate (**S54**) (1000 mg, 2.0 mmol, 1 eq.) was taken up in triethylamine (13 mL) and THF (3 mL) then the solution was treated with 4-ethynylthiazole (268 mg, 2.45 mmol, 1.2 eq). The reaction solution was degassed by bubbling nitrogen for 15 min. Pd(dppf)Cl_2.DCM (417 mg, 0.511 mmol, 0.25 eq.) and CuI (97 mg, 0.51 mmol, 0.25 eq.) were added and the mixture was degassed for a further 10 min. The reaction was heated to 80 °C under nitrogen for 16 h. Once the reaction was complete (monitored by LCMS), the reaction was cooled and diluted with EtOAc and the organics washed with chilled brine (x5). The organics were then separated and dried ($MgSO_4$) before concentrating to dryness. The crude residue was then separated by column chromatography (Combi-Flash) eluting with 0-100% EtOAc/*n*-Heptane to yield the title product as a yellow oil (690 mg, 75% yield).

^{1}H NMR (300 MHz, $CDCl_3$) δ 8.75 (s, 1H), 8.09 (s, 1H), 7.23 (dt, *J* = 8.9, 1.9 Hz, 1H), 6.52 (d, *J* = 8.6 Hz, 1H), 4.29 (bs, 2H), 4.16 (td, *J* = 4.8, 1.4 Hz, 2H), 3.99 (d, *J* = 1.0 Hz, 2H), 3.92 – 3.83 (m, 2H), 3.78 (ddd, *J* = 5.7, 3.8, 1.2 Hz, 2H), 3.74 – 3.66 (m, 2H), 1.45 (s, 9H).

LCMS (Method 2) RT = 1.58, $[M+H]^+$ = 420.2

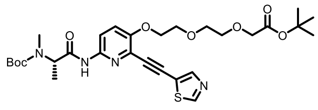

***tert*-butyl 2-[2-[2-[[6-[[(2*S*)-2-[*tert*-butoxycarbonyl(methyl)amino]propanoyl]amino]-2-(2-thiazol-5-ylethynyl)-3-pyridyl]oxy]ethoxy]ethoxy]acetate (S56)**: Boc-*N*-methyl-L-alanine (139 mg, 0.684 mmol, 1.2 eq.) was dissolved in Pyridine (2.29 mL, 50 eq.) and treated with *tert*-butyl 2-[2-[2-[[6-amino-2-(2-thiazol-5-ylethynyl)-3-pyridyl]oxy]ethoxy]ethoxy]acetate (**S55**) (239 mg, 0.570 mmol, 1 eq.) then T3P (0.998 mL, 1.71 mmol, 3 eq.). The reaction was stirred at rt for 10 min and was then diluted with EtOAC, brine and sat. $NaHCO_3$. The organic phase was collected and washed with chilled brine (x4), dried over $MgSO_4$ then concentrated under reduced pressure to a brown liquid. The crude material was separated using column chromatography (Combi-Flash) eluting with 0-75% EtOAc:*n*-Hept to obtain the title product as a yellow oil (249 mg, 72% yield).

^{1}H NMR (300 MHz, $CDCl_3$) δ 8.76 (s, 1H), 8.50 – 8.30 (m, 1H), 8.20 (d, *J* = 9.1 Hz, 1H), 8.09 (s, 1H), 7.33 (d, *J* = 9.1 Hz, 1H), 4.76 (bs, 1H), 4.25 – 4.16 (m, 2H), 3.99 – 3.85 (m, 4H), 3.83 – 3.74 (m, 2H), 3.74 – 3.63 (m, 2H), 2.80 (s, 3H), 1.44 (s, 9H), 1.42 (s, 9H), 1.38 (d, *J* = 7.0 Hz, 3H).

LCMS (Method 2) RT = 2.63, $[M+H]^+$ = 605.4

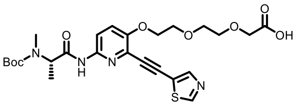

2-[2-[2-[[6-[[(2*S*)-2-[*tert*-butoxycarbonyl(methyl)amino]propanoyl]amino]-2-(2-thiazol-5-ylethynyl)-3-pyridyl]oxy]ethoxy]ethoxy]acetic acid (S57): *tert*-butyl 2-[2-[2-[[6-[[(2*S*)-2-[*tert*-butoxycarbonyl(methyl)amino]propanoyl]amino]-2-(2-thiazol-5-ylethynyl)-3-pyridyl]oxy]ethoxy]ethoxy]acetate (**S56**) (249 mg, 0.412 mmol, 1 eq.) was dissolved in THF (2.8 mL) and treated with a solution of hydroxylithium hydrate (20.7 mg, 0.494 mmol, 1.2 eq.) in Water (1.2 mL). The reaction formed a biphasic mixture, so additional water and THF was added until the two phases became one, with a final volume that was approx. 10 mL. Once the reaction was complete (monitored by LCMS), the THF was removed under reduced pressure and the remaining aqueous mixture was washed once with EtOAc to remove any unreacted starting material. The aqueous phase was collected and was acidified with 10% citric acid. The acidified aqueous layer was extracted three times with EtOAc, dried ($MgSO_4$) then concentrated under reduced pressure to yield the title product as a pale-yellow foam (176 mg, 78% yield).

^{1}H NMR (300 MHz, $CDCl_3$) δ 8.80 (s, 1H), 8.74 (s, 1H), 8.25 (d, *J* = 9.1 Hz, 1H), 8.14 (s, 1H), 7.38 (d, *J* = 9.2 Hz, 1H), 4.63 (bs, 1H), 4.25 (dd, *J* = 5.6, 3.5 Hz, 2H), 4.13 (s, 2H), 3.95 (dd, *J* = 5.5, 3.6 Hz, 2H), 3.87 – 3.79 (m, 2H), 3.75 (dt, *J* = 6.6, 2.2 Hz, 2H), 2.85 (s, 3H), 1.47 (s, 9H), 1.42 (d, *J* = 7.1 Hz, 3H).

LCMS (Method 2) RT = 2.11, $[M+H]^+$ = 549.4

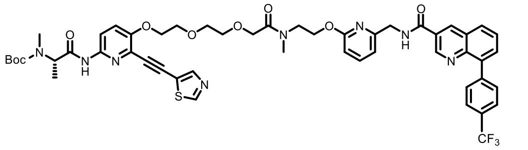

***tert*-butyl *N*-methyl-*N*-[(1*S*)-1-methyl-2-[[5-[2-[2-[2-[methyl-[2-[[6-[[[8-[4-(trifluoromethyl)phenyl]quinoline-3-carbonyl]amino]methyl]-2-pyridyl]oxy]ethyl]amino]-2-oxo-ethoxy]ethoxy]ethoxy]-6-(2-thiazol-5-ylethynyl)-2-pyridyl]amino]-2-oxo-ethyl]carbamate (S58):** 2-[2-[2-[[6-[[(2*S*)-2-[*tert*-butoxycarbonyl(methyl)amino]propanoyl]amino]-2-(2-thiazol-5-ylethynyl)-3-pyridyl]oxy]ethoxy]ethoxy]acetic acid (**S57**) (55 mg, 0.10 mmol, 1 eq.) and *N*-[[6-[2-(methylamino)ethoxy]-2-pyridyl]methyl]-8-[4-(trifluoromethyl)phenyl]quinoline-3-carboxamide (**S22**) (48 mg, 0.08 mmol, 0.8 eq.) were treated according to **General Procedure 2**. Column chromatography (0-5% MeOH in DCM) afforded the title compound as a clear oil (20 mg, 20 % yield). NMR in $CDCl_3$ shows evidence of rotamers, LCMS shows one peak.

^{1}H NMR (300 MHz, $CDCl_3$) δ 9.40 (t, *J* = 1.8 Hz, 1H), 8.88 (d, *J* = 2.1 Hz, 1H), 8.74 (s, 1H), 8.38 (s, 1H), 8.17 (t, *J* = 9.1 Hz, 1H), 8.08 (s, 1H), 7.99 (ddd, *J* = 15.4, 8.0, 1.5 Hz, 2H), 7.83 – 7.76 (m, 4H), 7.73 (d, *J* = 8.3 Hz, 2H), 7.66 (dd, *J* = 8.1, 7.2 Hz, 1H), 7.60 – 7.49 (m, 1H), 7.31 – 7.20 (m, 1H), 6.95 (dd, *J* = 12.5, 7.2 Hz, 1H), 6.61 (t, *J* = 7.6 Hz, 1H), 4.70 (dd, *J* = 14.6, 5.2 Hz, 2H), 4.50 (dt, *J* = 11.1, 5.5 Hz, 2H), 4.28 (s, 1H), 4.22 –

4.00 (m, 4H), 3.83 (dt, *J* = 6.2, 3.1 Hz, 2H), 3.78 – 3.55 (m, 6H), 2.96 (d, *J* = 16.2 Hz, 3H), 2.82 (s, 3H), 1.46 (s, 9H), 1.40 (dd, *J* = 7.2, 1.8 Hz, 3H).

LCMS (Method 2) RT = 3.03, $[M+2H]/2^+$ = 506.4

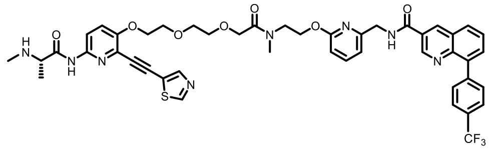

***N*-[[6-[2-[methyl-[2-[2-[2-[[6-[[(2*S*)-2-(methylamino)propanoyl]amino]-2-(2-thiazol-5-ylethynyl)-3-pyridyl]oxy]ethoxy]ethoxy]acetyl]amino]ethoxy]-2-pyridyl]methyl]-8-[4-(trifluoromethyl)phenyl]quinoline-3-carboxamide (A232):** *tert*-butyl *N*-methyl-*N*-[(1*S*)-1-methyl-2-[[5-[2-[2-[2-[methyl-[2-[[6-[[[8-[4-(trifluoromethyl)phenyl]quinoline-3-carbonyl]amino]methyl]-2-pyridyl]oxy]ethyl]amino]-2-oxo-ethoxy]ethoxy]ethoxy]-6-(2-thiazol-5-ylethynyl)-2-pyridyl]amino]-2-oxo-ethyl]carbamate (**S58**) (20 mg, 0.02 mmol, 1 eq.) was subjected to **General Procedure 1.** HPLC and freeze drying afforded the title compound as a white solid (14.5 mg, 79% yield). NMR in MeOD-d4 shows evidence of rotamers, LCMS shows one peak.

^{1}H NMR (300 MHz, MeOD) δ 9.26 (dd, *J* = 3.9, 2.2 Hz, 1H), 8.97 (d, *J* = 2.3 Hz, 1H), 8.85 (t, *J* = 2.2 Hz, 1H), 8.11 – 7.99 (m, 3H), 7.90 – 7.66 (m, 6H), 7.58 (td, *J* = 7.8, 3.0 Hz, 1H), 7.45 (dd, *J* = 9.2, 7.3 Hz, 1H), 6.97 (d, *J* = 7.4 Hz, 1H), 6.60 (dd, *J* = 8.3, 5.2 Hz, 1H), 4.64 (s, 2H), 4.45 (td, *J* = 5.6, 2.8 Hz, 2H), 4.24 (s, 1H), 4.21 – 4.10 (m, 3H), 3.87 – 3.78 (m, 2H), 3.76 – 3.54 (m, 5H), 3.49 (d, *J* = 7.2 Hz, 1H), 3.35 (d, *J* = 2.0 Hz, 1H), 2.95 (s, 1.5H), 2.89 (s, 1.5H), 2.48 (s, 3H), 1.41 (d, *J* = 6.9 Hz, 3H). 1:1 rotamer splitting

^{19}F NMR (282 MHz, MeOD) δ -63.77, -63.79.

^{13}C NMR (75 MHz, MeOD) δ 173.3, 172.1, 171.9, 168.0, 167.9, 164.5, 164.2, 157.0, 156.8, 156.7, 155.3, 149.8, 149.8, 148.6, 147.5, 145.8, 145.8, 144.4, 141.0, 140.8, 140.4, 140.3, 137.7, 133.5, 133.4, 132.4, 131.3, 130.9, 130.8, 130.8, 130.6, 130.2, 128.9, 128.6, 128.5, 128.4, 128.4, 127.7, 125.8, 125.8, 125.7, 124.1, 123.8, 119.8, 119.7, 117.0, 115.7, 115.4, 110.3, 110.3, 93.3, 93.2, 83.5, 83.5, 72.1, 71.8, 71.7, 70.7, 70.7, 70.6, 70.5, 64.0, 63.9, 60.3, 46.0, 45.9, 36.1, 34.3, 33.8, 18.2.

LCMS (Method 3 - Bundoora) RT = 1.97, $[M+H]^+$ = 911.2

HRMS (ESI+) *m/z*: $[M+H]^+$ calcd for $C_{46}H_{46}F_3N_8O_7S$ 911.3162; found 911.3140.

*Synthesis of **A230** (**A232** IAP negative control)*

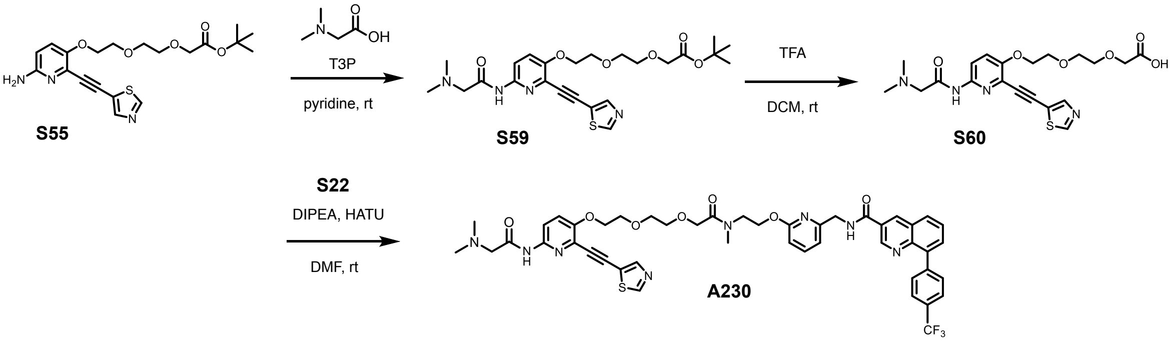

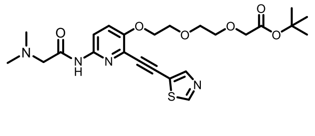

***tert*-butyl 2-[2-[2-[[6-[[2-(dimethylamino)acetyl]amino]-2-(2-thiazol-5-ylethynyl)-3-pyridyl]oxy]ethoxy]ethoxy]acetate (S59)**: *tert*-butyl 2-[2-[2-[[6-amino-2-(2-thiazol-5-ylethynyl)-3-pyridyl]oxy]ethoxy]ethoxy]acetate (**S55**) (150 mg, 0.37 mmol, 1 eq.) was dissolved in pyridine (2.29 ml) and treated with 2-(dimethylamino)acetic acid (45 mg, 0.44 mmol, 1.2 eq.) and then T3P (0.998 ml 50% in EtOAc, 1.71 mmol, 3 eq.). The reaction stirred at rt for 30 min. The reaction was diluted with EtOAc, then brine and sat. $NaHCO_3$ were added. The biphasic solution was stirred until bubbling ceased. The organic phase was collected and washed with chilled brine (x4), dried over $MgSO_4$ then concentrated under reduced pressure to a brown liquid (contained pyridine). The crude material was purified by combi-flash on a 40 g column eluting with MeOH:DCM (0-5%) to give product as a pale yellow oil (117 mg, 63% yield).

^{1}H NMR (300 MHz, $CDCl_3$) δ 9.66 (s, 1H), 8.76 (d, *J* = 0.6 Hz, 1H), 8.25 (d, *J* = 9.1 Hz, 1H), 8.10 (d, *J* = 0.7 Hz, 1H), 7.34 (d, *J* = 9.1 Hz, 1H), 4.22 (dd, *J* = 5.5, 4.1 Hz, 2H), 3.98 (s, 2H), 3.92 (dd, *J* = 5.5, 4.1 Hz, 2H), 3.84 – 3.74 (m, 2H), 3.74 – 3.65 (m, 2H), 3.08 (s, 2H), 2.34 (s, 6H), 1.44 (s, 9H).

LCMS (Method 2) RT = 1.47 min, $[M+H]^+$ = 505.3

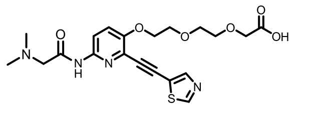

2-[2-[2-[[6-[[2-(dimethylamino)acetyl]amino]-2-(2-thiazol-5-ylethynyl)-3-pyridyl]oxy]ethoxy]ethoxy]acetic acid (S60): *tert*-butyl 2-[2-[2-[[6-[[2-(dimethylamino)acetyl]amino]-2-(2-thiazol-5-ylethynyl)-3-pyridyl]oxy]ethoxy]ethoxy]acetate (**S59**) (120 mg, 0.23 mmol, 1 eq.) was dissolved in DCM (3 mL) and then treated with TFA (1 mL). The solution was stirred at rt overnight. The reaction was concentrated under reduced pressure to a yellow oil. The TFA crude salt was free-based with an SCX column (washing with MeOH, loading and washing with MeOH, then eluting with ~1N NH_3 in MeOH). The product containing filtrate was concentrated under reduced pressure to a light brown oily solid (92 mg, 80% yield). H-NMR and LCMS showed product with a minor impurity (~10%).

^{1}H NMR (300 MHz, $CDCl_3$) δ 9.77 (s, 1H), 8.79 (s, 1H), 8.24 (d, *J* = 9.1 Hz, 1H), 8.14 (s, 1H), 7.37 (d, *J* = 9.1 Hz, 1H), 4.25 (t, *J* = 4.5 Hz, 2H), 4.10 (s, 2H), 3.95 (t, *J* = 4.6 Hz, 2H), 3.83 (dd, *J* = 5.8, 2.7 Hz, 2H), 3.78 – 3.69 (m, 2H), 3.18 (s, 2H), 2.41 (s, 6H).

LCMS (Method 2) RT = 1.10 min, $[M+H]^+$ = 449.2

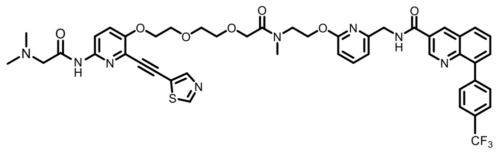

***N*-[[6-[2-[[2-[2-[2-[[6-[[2-(dimethylamino)acetyl]amino]-2-(2-thiazol-5-ylethynyl)-3-pyridyl]oxy]ethoxy]ethoxy]acetyl]-methyl-amino]ethoxy]-2-pyridyl]methyl]-8-[4-(trifluoromethyl)phenyl]quinoline-3-carboxamide (A230)**: 2-[2-[2-[[6-[[2-(dimethylamino)acetyl]amino]-2-(2-thiazol-5-ylethynyl)-3-pyridyl]oxy]ethoxy]ethoxy]acetic acid (**S60**) (88 mg, 0.18 mmol, 1.2 eq.) and *N*-[[6-[2-(methylamino)ethoxy]-2-pyridyl]methyl]-8-[4-(trifluoromethyl)phenyl]quinoline-3-carboxamide (**S22**) (87 mg, 0.15 mmol, 1 eq.) were dissolved in DMF (1.5 mL) then treated with DIPEA (31 µL, 0.18 mmol, 1.2 eq.), and then HATU (101 mg, 0.265 mmol, 1.5 eq.) and stirred at rt for 35 min. The reaction mixture was partitioned between EtOAc and brine. The organic layer was washed with chilled brine (x5), dried ($MgSO_4$), and then concentrated under reduced pressure. The crude residue was purifed by Combi-Flash on a 24 g column eluting with MeOH:DCM (0-5%) and further purified by prep-LCMS. The clean fractions were combined and lyophilized to give the formate salt of the product as a white solid (55 mg, 35% yield).

^{1}H NMR (300 MHz, MeOD) δ 9.26 (dd, *J* = 3.9, 2.2 Hz, 1H), 8.97 (d, *J* = 2.3 Hz, 1H), 8.85 (t, *J* = 2.2 Hz, 1H), 8.11 – 7.99 (m, 3H), 7.90 – 7.66 (m, 6H), 7.58 (td, *J* = 7.8, 3.0 Hz, 1H), 7.45 (dd, *J* = 9.2, 7.3 Hz, 1H), 6.97 (d, *J* = 7.4 Hz, 1H), 6.60 (dd, *J* = 8.3, 5.2 Hz, 1H), 4.64 (s, 2H), 4.45 (td, *J* = 5.6, 2.8 Hz, 2H), 4.24 (s, 1H), 4.21 – 4.10 (m, 3H), 3.87 – 3.78 (m, 2H), 3.76 – 3.54 (m, 5H), 3.49 (d, *J* = 7.2 Hz, 1H), 3.35 (d, *J* = 2.0 Hz, 1H), 2.95 (s, 1.5H), 2.89 (s, 1.5H), 2.48 (s, 3H), 1.41 (d, *J* = 6.9 Hz, 3H). 1:1 rotamer splitting

^{19}F NMR (282 MHz, MeOD) δ -63.77, -63.79.

^{13}C NMR (75 MHz, MeOD) δ 172.1, 171.9, 170.5, 168.0, 167.9, 164.6, 164.3, 157.0, 156.8, 156.7, 155.3, 149.8, 148.6, 147.6, 145.7, 144.4, 141.0, 140.8, 140.4, 137.7, 133.4, 132.4, 130.8, 130.8, 128.9, 128.6, 128.5, 125.8, 125.8, 124.1, 119.8, 116.8, 115.7, 115.4, 110.3, 93.2, 72.1, 71.8, 71.7, 70.7, 64.0, 63.9, 63.5, 46.0, 45.9, 36.1, 34.3.

LCMS (Method 2) RT = 1.75 min, $[M+H]^+$ = 911.2

HRMS (ESI+) *m/z*: $[M+H]^+$ calcd for $C_{46}H_{46}F_3N_8O_7S$ 911.3162; found 911.3168.

*Synthesis of **A231** (**A232** TEAD negative control)*

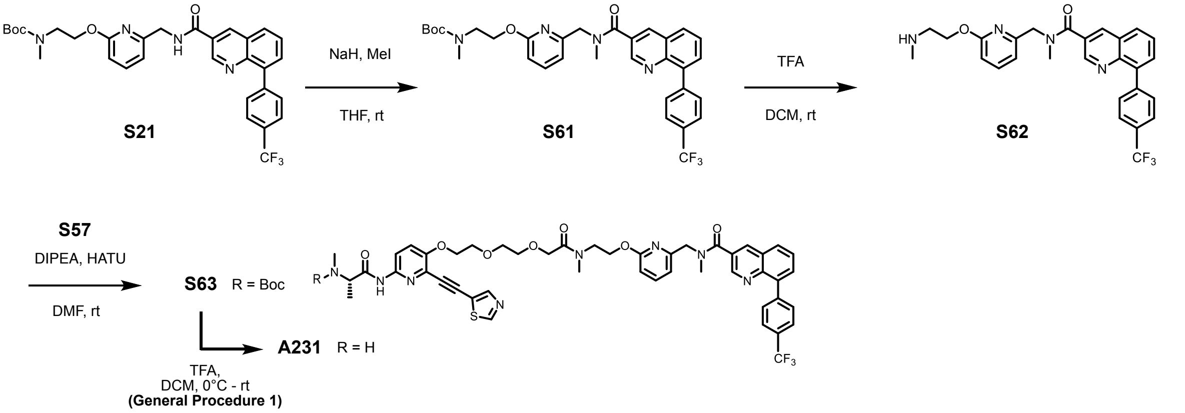

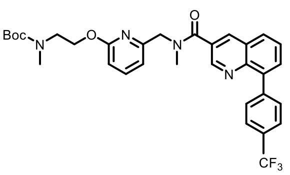

tert-butyl *N*-methyl-*N*-[2-[[6-[[methyl-[8-[4-(trifluoromethyl)phenyl]quinoline-3-carbonyl]amino]methyl]-2-pyridyl]oxy]ethyl]carbamate (S61): *tert*-butyl *N*-methyl-*N*-[2-[[6-[[[8-[4-(trifluoromethyl)phenyl]quinoline-3-carbonyl]amino]methyl]-2-pyridyl]oxy]ethyl]carbamate (**S21**) (110 mg, 0.17 mmol, 1 eq.) was dissolved in dry THF (2 mL) and treated with Sodium Hydride (17 mg 60% purity, 0.43 mmol, 2.5 eq.) at rt. The reaction bubbled and was stirred for 30 min, after which time gas evolution had ceased. Iodomethane (12.7 µL, 0.205 mmol, 1.2 eq.) was added and the reaction stirred at rt for 30 min. The reaction was diluted with EtOAc, quenched with water, and brine was added. The organic phase was collected, then the aqueous phase was extracted twice more with EtOAc. The combined organic phases were dried over $MgSO_4$ and concentrated under reduced pressure. The crude material was purified by Combi-Flash on a 40 g silica column eluting with EtOAc:n-Hept (0-60%) to give a yellow oil (59 mg, 58% yield).

^{1}H NMR (300 MHz, $CDCl_3$) δ 9.11 – 9.02 (m, 1H), 8.41 (d, *J* = 12.4 Hz, 1H), 8.01 – 7.48 (m, 8H), 6.97 (d, *J* = 7.3 Hz, 0.5H), 6.75 (d, *J* = 7.2 Hz, 0.5H), 6.66 (d, *J* = 8.2 Hz, 1H), 4.81 (s, 1H), 4.55 (s, 1H), 4.47 – 4.37 (m, 2H), 3.60 (s, 2H), 3.15 (s, 3H), 2.94 (d, *J* = 5.4 Hz, 3H), 1.40 (s, 9H). 1:1 rotamer splitting

LCMS (Method 2) RT = 3.12 min, $[M+H]^+$ = 595.4

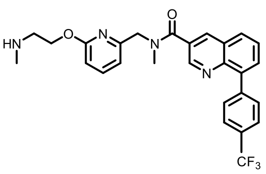

***N*-methyl-N-[[6-[2-(methylamino)ethoxy]-2-pyridyl]methyl]-8-[4-(trifluoromethyl)phenyl]quinoline-3-carboxamide (S62)**: *tert*-butyl *N*-methyl-*N*-[2-[[6-[[methyl-[8-[4-(trifluoromethyl)phenyl]quinoline-3-carbonyl]amino]methyl]-2-pyridyl]oxy]ethyl]carbamate (**S61**) (59 mg, 0.100 mmol, 1 eq.) was dissolved in DCM (4 mL) and treated with a solution of TFA (1 mL) in DCM (1 mL). Stirred at rt for 2.5 h. The

reaction was treated carefully with sat. $NaHCO_3$ until pH 10. The organic layer was collected and the aqueous layer extracted twice with DCM. The combined organic layers were washed once with brine, dried ($MgSO_4$) then concentrated under reduced pressure to an off-white solid (50 mg, 96% yield)

^{1}H NMR (300 MHz, $CDCl_3$) δ 9.06 (d, *J* = 14.2 Hz, 1H), 8.43 (d, *J* = 2.4 Hz, 1H), 8.01 – 7.48 (m, 8H), 6.97 (d, *J* = 7.3 Hz, 0.5H), 6.71 (t, *J* = 9.5 Hz, 1.5H), 4.81 (s, 1H), 4.54 (s, 1H), 4.51 – 4.39 (m, 2H), 3.14 (s, 3H), 3.07 – 2.96 (m, 2H), 2.62 – 2.39 (m, 4H). 1:1 rotamer splitting

LCMS (Method 2) RT = 1.83 min, $[M+H]^+$ = 495.2

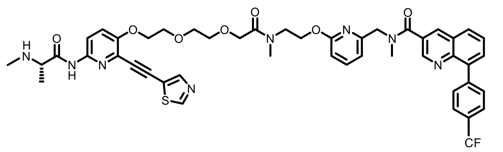

***N*-methyl-*N*-[[6-[2-[methyl-[2-[2-[2-[[6-[[(2*S*)-2-(methylamino)propanoyl]amino]-2-(2-thiazol-5-ylethynyl)-3-pyridyl]oxy]ethoxy]ethoxy]acetyl]amino]ethoxy]-2-pyridyl]methyl]-8-[4-(trifluoromethyl)phenyl]quinoline-3-carboxamide (A231)**: 2-[2-[2-[[6-[[(2*S*)-2-[*tert*-butoxycarbonyl(methyl)amino]propanoyl]amino]-2-(2-thiazol-5-ylethynyl)-3-pyridyl]oxy]ethoxy]ethoxy]acetic acid (**S57**) (55 mg, 0.10 mmol, 1 eq.) and *N*-methyl-*N*-[[6-[2-(methylamino)ethoxy]-2-pyridyl]methyl]-8-[4-(trifluoromethyl)phenyl]quinoline-3-carboxamide (**S62**) (49.6 mg, 0.100 mmol, 1 eq.) were dissovled in DMF (1 mL) and treated with DIPEA (87 µL, 0.50 mmol, 5 eq.) then HATU (57 mg, 0.15 mmol, 1.5 eq.). The reaction mixture was stirred at rt for 10 min. The reaction was diluted with EtOAc and washed with chilled brine (x5). The organic layer was dried ($MgSO_4$) and then concentrated under reduced pressure to a crude residue which was purifed by Combi-Flash on a 24 g column eluting with MeOH:DCM (0-5%) to give Boc-protected intermediate **S63** (72 mg).

The Boc-protected intermediate **S63** was dissolved in DCM (2 mL) at 0°C and treated with TFA (0.268 mL, 3.50 mmol) in DCM (1.5 mL). The reaction was stirred at rt for 16 h. The reaction was treated carefully with sat. $NaHCO_3$. When bubbling ceased the organic layer was collected and the aqueous phase was extracted twice more with DCM. The combined organic layers were washed once with brine, dried ($MgSO_4$) and concentrated under reduced pressure to an off-white solid. The crude material was purified by prep-LCMS. The clean fractions were concentrated then freeze dried to give the title compound formate salt as a white solid (25.8 mg, 40% yield).

^{1}H NMR (300 MHz, MeOD) δ 9.05 – 8.91 (m, 2H), 8.59 – 8.50 (m, 1H), 8.14 – 8.03 (m, 2H), 8.06 – 7.94 (m, 1H), 7.87 – 7.66 (m, 6H), 7.54 (dddd, *J* = 40.8, 17.1, 10.6, 6.3 Hz, 2H), 6.91 (dd, *J* = 50.5, 7.5 Hz, 1H), 6.65 (t, *J* = 8.7 Hz, 1H), 4.80 (s, 1H), 4.58 (s, 1H), 4.45 (dt, *J* = 14.6, 4.9 Hz, 2H), 4.21 (d, *J* = 20.2 Hz, 4H), 3.89 – 3.56 (m, 8H), 3.42 (dd, *J* = 6.9, 3.1 Hz, 1H), 3.14 (d, *J* = 17.1 Hz, 3H), 2.95 (dd, *J* = 25.2, 13.6 Hz, 3H), 2.44 (d, *J* = 3.7 Hz, 3H), 1.38 (dd, *J* = 7.0, 3.2 Hz, 3H). 1:1:1:1 rotamer splitting.

^{19}F NMR (282 MHz, MeOD) δ -63.77, -63.78, -63.80, -63.81.

^{13}C NMR (126 MHz, MeOD) δ 174.4, 172.1, 172.0, 171.9, 171.0, 165.0, 164.7, 164.4, 157.0, 155.3, 155.3, 154.8, 154.8, 149.5, 149.5, 149.4, 148.6, 146.6, 146.6, 146.6, 145.9, 145.9, 144.4, 141.2, 141.1, 141.0, 140.9, 140.4, 136.9, 136.8, 136.7, 133.0, 132.4, 130.9, 130.8, 130.7, 130.4, 130.3, 130.3, 128.9, 128.8, 128.7, 127.0, 125.8, 125.8, 125.8, 124.8, 123.9, 123.8, 119.8, 119.8, 117.0, 116.9, 116.6, 116.4, 116.3,

116.1, 111.4, 111.4, 110.6, 93.3, 93.2, 83.5, 83.5, 72.1, 72.0, 71.7, 71.6, 70.8, 70.7, 70.6, 64.5, 64.3, 64.1, 60.6, 57.3, 53.4, 39.2, 39.1, 36.1, 36.0, 34.5, 34.5, 34.2, 34.1, 18.7.

LCMS (Method 2) RT = 1.83 min, $[M+H]^+$ = 925.2.

HRMS (ESI+) *m/z*: $[M+H]^+$ calcd for $C_{47}H_{48}F_3N_8O_7S$ 925.3319; found 925.3312.

Synthesis of Tracers

*Synthesis of **A191** (Biotinylated XB2 TR-FRET Tracer)*

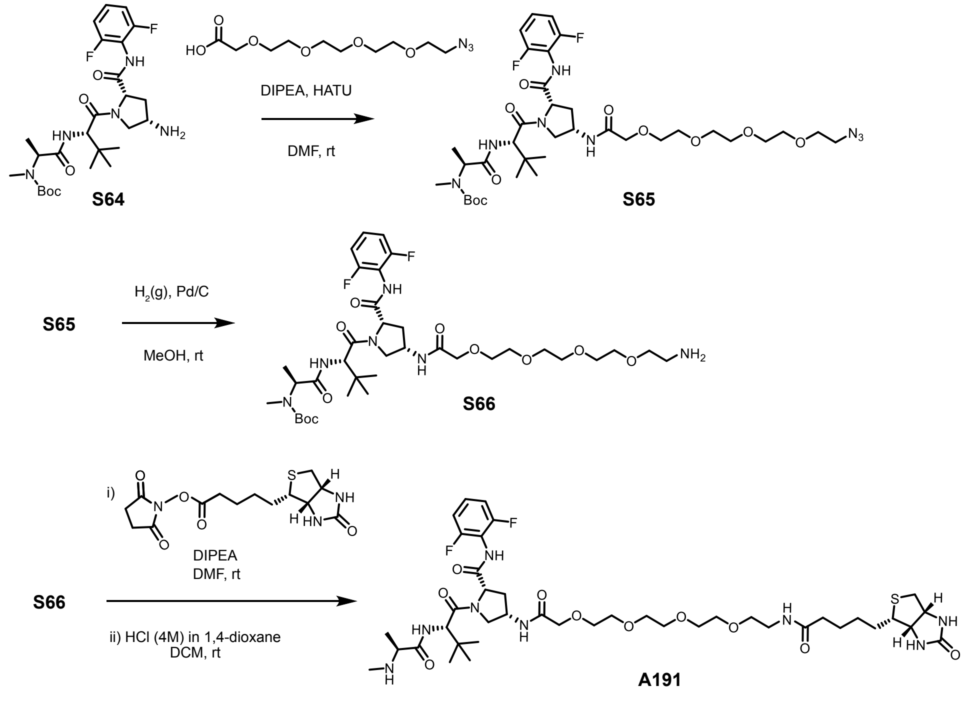

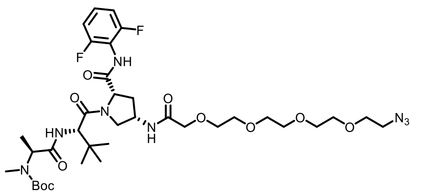

***tert*-butyl *N*-[(1*S*)-2-[[(1*S*)-1-[(2*S*,4*S*)-4-[[2-[2-[2-[2-(2-azidoethoxy)ethoxy]ethoxy]ethoxy]acetyl]amino]-2-[(2,6-difluorophenyl)carbamoyl]pyrrolidine-1-carbonyl]-2,2-dimethyl-propyl]amino]-1-methyl-2-oxo-ethyl]-*N*-methyl-carbamate (S65)**: *tert*-butyl *N*-[(1*S*)-2-[[(1*S*)-1-[(2*S*,4*S*)-4-amino-2-[(2,6-difluorophenyl)carbamoyl]pyrrolidine-1-carbonyl]-2,2-dimethyl-propyl]amino]-1-methyl-2-oxo-ethyl]-*N*-methyl-carbamate (**S64**) (50 mg, 0.083 mmol, 1 eq.) was dissolved in DMF (2.0 mL) and treated with 2-[2-[2-[2-(2-azidoethoxy)ethoxy]ethoxy]ethoxy]acetic acid (167 µL, 0.083 mmol, 1 eq.), DIPEA (58 µL, 0.33 mmol, 4 eq.) and HATU (47.6 mg, 0.125 mmol, 1.5 eq.). The yellow solution was stirred at rt for 5 min. The reaction was diluted with EtOAc and washed twice with chilled brine. The organic layer was dried ($MgSO_4$) and then concentrated under reduced pressure. The crude material was purified by Combi-Flash on a 12 g silica column eluting with MeOH:DCM (0-10%). Product fractions were concentrated to give the product as a pale yellow oil (25.0 mg, 38% yield).

^{1}H NMR (300 MHz, $CDCl_3$) δ 9.17 (s, 1H), 8.25 (d, *J* = 6.8 Hz, 1H), 7.27 – 7.13 (m, 1H), 6.94 (t, *J* = 8.1 Hz, 3H), 5.02 (d, *J* = 8.2 Hz, 1H), 4.71 (bs, 1H), 4.62 (q, *J* = 7.0 Hz, 1H), 4.48 (d, *J* = 8.6 Hz, 1H), 4.15 (dd, *J* =

11.0, 5.4 Hz, 1H), 4.00 (d, J = 15.8 Hz, 1H), 3.90 (d, J = 15.7 Hz, 1H), 3.74 (d, J = 10.4 Hz, 1H), 3.70 – 3.40 (m, 14H), 3.37 (t, J = 5.1 Hz, 2H), 2.78 (s, 3H), 2.55 (d, J = 14.3 Hz, 1H), 2.38 – 2.22 (m, 1H), 1.49 (s, 9H), 1.32 (d, J = 7.1 Hz, 3H), 0.98 (s, 9H).

LCMS (Method 2) RT= 2.37 min, $[M-H]^-$ = 797.4

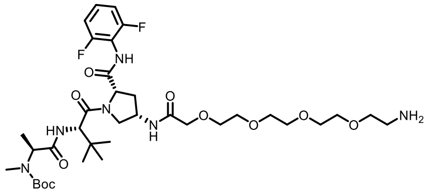

***tert*-butyl *N*-[(1*S*)-2-[[(1*S*)-1-[(2*S*,4*S*)-4-[[2-[2-[2-[2-(2-aminoethoxy)ethoxy]ethoxy]ethoxy]acetyl]amino]-2-[(2,6-difluorophenyl)carbamoyl]pyrrolidine-1-carbonyl]-2,2-dimethyl-propyl]amino]-1-methyl-2-oxo-ethyl]-*N*-methyl-carbamate (S66)**: A solution of *tert*-butyl *N*-[(1*S*)-2-[[(1*S*)-1-[(2*S*,4*S*)-4-[[2-[2-[2-[2-(2-azidoethoxy)ethoxy]ethoxy]ethoxy]acetyl]amino]-2-[(2,6-difluorophenyl)carbamoyl]pyrrolidine-1-carbonyl]-2,2-dimethyl-propyl]amino]-1-methyl-2-oxo-ethyl]-*N*-methyl-carbamate (**S65**) (25 mg, 0.031 mmol, 1 eq.) in MeOH (2 mL) was degassed with N_2 at rt. 10% Pd/C (0.33 mg, 0.0033 mmol) was added and the mixture was degassed again before the introduction of a balloon of H_2. The mixture was stirred overnight at rt. The mixture was subjected to SCX chromatography using a small pad of Celite at the top of the column. The ammonia fraction was concentrated to give product as a yellow solid (18 mg, 74% yield).

^{1}H NMR (300 MHz, $CDCl_3$) δ 9.20 (s, 1H), 8.21 (d, J = 6.8 Hz, 1H), 7.26 – 7.13 (m, 1H), 6.93 (t, J = 8.1 Hz, 3H), 5.01 (d, J = 7.4 Hz, 1H), 4.70 (s, 1H), 4.62 (d, J = 5.7 Hz, 1H), 4.48 (d, J = 8.6 Hz, 1H), 4.16 (dd, J = 10.8, 5.6 Hz, 1H), 4.00 (d, J = 15.5 Hz, 1H), 3.91 (d, J = 15.6 Hz, 1H), 3.73 (d, J = 11.5 Hz, 1H), 3.69 – 3.41 (m, 14H), 2.89 (d, J = 4.9 Hz, 1H), 2.77 (s, 3H), 2.53 (d, J = 14.1 Hz, 1H), 2.48 – 2.23 (m, 2H), 2.10 (s, 2H), 1.48 (s, 9H), 1.31 (d, J = 7.2 Hz, 3H), 0.98 (s, 9H).

LCMS (Method 2) RT =1.62 min, $[M+H]^+$ = 773.5

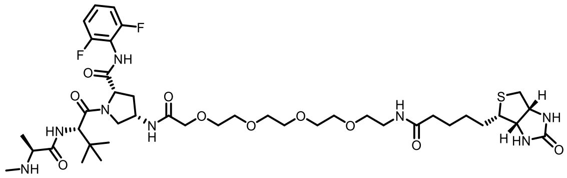

(2*S*,4*S*)-4-[[2-[2-[2-[2-[2-[5-[(3a*S*,4*S*,6a*R*)-2-oxo-1,3,3a,4,6,6a-hexahydrothieno[3,4-d]imidazol-4-yl]pentanoylamino]ethoxy]ethoxy]ethoxy]ethoxy]acetyl]amino]-*N*-(2,6-difluorophenyl)-1-[(2S)-3,3-dimethyl-2-[[(2*S*)-2-(methylamino)propanoyl]amino]butanoyl]pyrrolidine-2-carboxamide (A191): NHS-Biotin (8.7 mg, 0.026 mmol, 1.1 eq.) was dissolved in DMF (1 mL) and treated with *tert*-butyl *N*-[(1*S*)-2-[[(1*S*)-1-[(2*S*,4*S*)-4-[[2-[2-[2-[2-(2-aminoethoxy)ethoxy]ethoxy]ethoxy]acetyl]amino]-2-[(2,6-difluorophenyl)carbamoyl]pyrrolidine-1-carbonyl]-2,2-dimethyl-propyl]amino]-1-methyl-2-oxo-ethyl]-*N*-methyl-carbamate (**S66**) (18.0 mg, 0.023 mmol, 1 eq.) and DIPEA (10.1 µL, 0.0582 mmol, 2.5 eq.). The

reaction was stirred at rt overnight. The reaction was then diluted with acetonitrile and aspirated under nitrogen. The residue was taken up in DCM (1 mL) and 4 M HCl in 1,4-dioxane (116 µL, 0.47 mmol, 20 eq.) was added. The mixture was stirred overnight at rt. LCMS showed complete deprotection of the amine. The solvents were removed by aspiration and the crude residue was subjected to acidic RP HPLC. Product containing fractions were combined, dried *in vacuo* and further freeze-dried to give the formate salt of the product as a white solid (4.3 mg, 18% yield).

^{1}H NMR (300 MHz, $CDCl_3$) δ 10.06 (s, 1H), 8.49 (d, *J* = 8.8 Hz, 1H), 7.93 (d, *J* = 6.8 Hz, 1H), 7.18 (dd, *J* = 17.1, 8.7 Hz, 2H), 6.92 (t, *J* = 8.0 Hz, 2H), 6.59 (s, 1H), 5.97 (s, 1H), 4.89 (s, 1H), 4.78 (d, *J* = 9.4 Hz, 1H), 4.63 (s, 1H), 4.52 (s, 1H), 4.38 (s, 1H), 4.19 (s, 2H), 4.04 (s, 2H), 3.69 – 3.49 (m, 14H), 3.41 (t, *J* = 5.3 Hz, 3H), 3.22 – 3.14 (m, 1H), 2.98 – 2.87 (m, 1H), 2.81 – 2.67 (m, 2H), 2.57 (s, 3H), 2.20 (t, *J* = 7.1 Hz, 3H), 1.67 (q, *J* = 7.2 Hz, 5H), 1.44 (d, *J* = 14.7 Hz, 5H), 1.03 (s, 9H).

LCMS (Method 2) RT =1.32 min, $[M+H]^+$ = 899.4

HRMS (ESI+) *m/z:* $[M+H]^+$ calcd for $C_{48}H_{60}BF_2N_8O_7S$ 941.4367; found 941.4345.

*Synthesis of **B678** (IAP nanoBRET Tracer)*

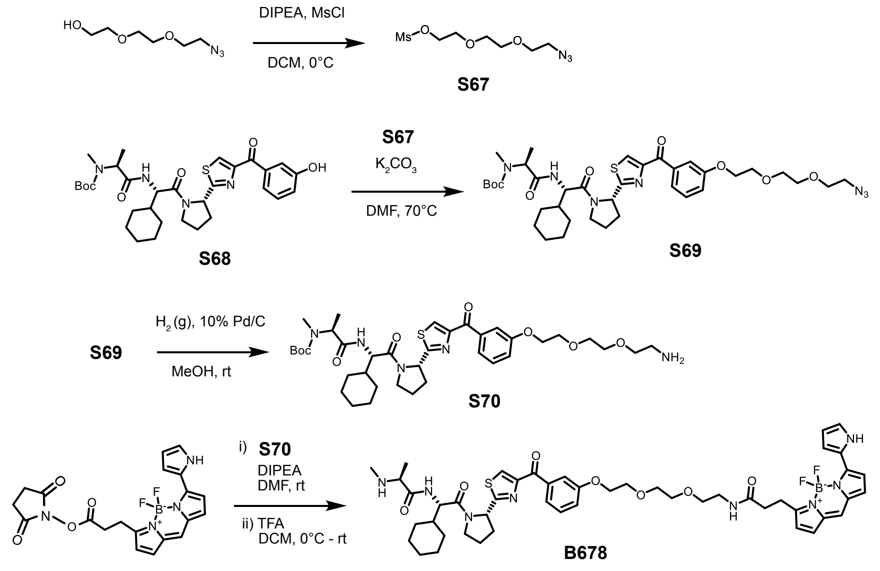

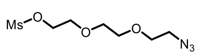

2-[2-(2-azidoethoxy)ethoxy]ethyl methanesulfonate (S67): To a solution of 2-[2-(2-azidoethoxy)ethoxy]ethanol (210 mg, 1.2 mmol, 1 eq.) in DCM (12 mL) at 0 °C was added DIPEA (630 µL, 3.6 mmol, 3 eq.) followed by methanesulfonyl chloride (140 µL, 1.8 mmol, 1.5 eq.). The mixture was stirred at rt for 30 min. TLC (100% EtOAc) showed some starting material remaining, therefore 0.5 eq. of MsCl were added. After 30 min, TLC showed no trace of starting material. The mixture was diluted in DCM and partitioned with 10% aq. $NaHCO_3$. The aqueous layer was extracted (x3) and the combined

organics were dried over $MgSO_4$ and concentrated. The residue was purified by column chromatography (silica gel, 0-100% EtOAc in hept) to yield product as a yellow oil (250 mg, 82% yield).

^{1}H NMR (300 MHz, $CDCl_3$) δ 4.43 – 4.34 (m, 2H), 3.83 – 3.76 (m, 2H), 3.68 (qd, *J* = 4.0, 2.2 Hz, 6H), 3.39 (t, *J* = 5.0 Hz, 2H), 3.07 (s, 3H).

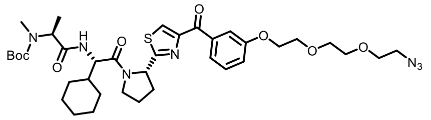

***tert*-butyl *N*-[(1*S*)-2-[[(1*S*)-2-[(2*S*)-2-[4-[3-[2-[2-(2-azidoethoxy)ethoxy]ethoxy]benzoyl]thiazol-2-yl]pyrrolidin-1-yl]-1-cyclohexyl-2-oxo-ethyl]amino]-1-methyl-2-oxo-ethyl]-*N*-methyl-carbamate (S69)**: *tert*-butyl ((*S*)-1-(((*S*)-1-cyclohexyl-2-((*S*)-2-(4-(3-hydroxybenzoyl)thiazol-2-yl)pyrrolidin-1-yl)-2-oxoethyl)amino)-1-oxopropan-2-yl)(methyl)carbamate (**S68**) (50 mg, 0.084 mmol, 1 eq.) and K_2CO_3 (21.6 mg, 0.167 mmol, 2 eq.) were taken up in DMF (1 mL) under nitrogen. 2-[2-(2-azidoethoxy)ethoxy]ethyl methanesulfonate (**S67**) (23 mg, 0.092 mmol, 1.1 eq.) was added and the reaction was stirred at 70 ^oC for 16 h. The reaction was diluted with EtOAc and washed with chilled brine (x3). The organic phase was dried with $MgSO_4$ then concentrated under reduced pressure. The residue was purified by column chromatography (0-5% MeOH in DCM) to yield product (47 mg, 74% yield).

^{1}H NMR (300 MHz, $CDCl_3$) δ 8.10 (s, 1H), 7.79 (dt, *J* = 7.7, 1.3 Hz, 1H), 7.73 – 7.63 (m, 1H), 7.37 (t, *J* = 7.9 Hz, 1H), 7.16 (ddd, *J* = 8.3, 2.7, 1.1 Hz, 1H), 6.69 (s, 1H), 5.55 (dd, *J* = 7.7, 2.6 Hz, 1H), 4.63 (dd, *J* = 8.9, 6.3 Hz, 2H), 4.20 (dd, *J* = 5.6, 3.9 Hz, 2H), 3.89 (dd, *J* = 5.6, 4.0 Hz, 2H), 3.84 – 3.58 (m, 7H), 3.38 (t, *J* = 5.1 Hz, 2H), 2.79 (s, 3H), 2.48 (ddd, *J* = 12.0, 6.4, 3.5 Hz, 1H), 2.37 – 2.02 (m, 3H), 1.68 (dd, *J* = 19.3, 10.2 Hz, 7H), 1.47 (s, 9H), 1.33 (d, *J* = 7.1 Hz, 3H), 1.10 (ddt, *J* = 35.5, 21.5, 11.3 Hz, 5H).

LCMS (Method 2) RT = 3.07 min, $[M+H]^+$ = 756.4

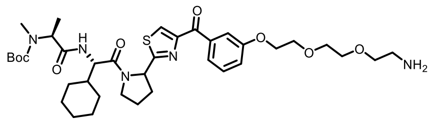

***tert*-butyl *N*-[(1*S*)-2-[[(1*S*)-2-[(2*S*)-2-[4-[3-[2-[2-(2-aminoethoxy)ethoxy]ethoxy]benzoyl]thiazol-2-yl]pyrrolidin-1-yl]-1-cyclohexyl-2-oxo-ethyl]amino]-1-methyl-2-oxo-ethyl]-*N*-methyl-carbamate (S70)**: *tert*-butyl *N*-[(1*S*)-2-[[(1*S*)-2-[(2*S*)-2-[4-[3-[2-[2-(2-azidoethoxy)ethoxy]ethoxy]benzoyl]thiazol-2-yl]pyrrolidin-1-yl]-1-cyclohexyl-2-oxo-ethyl]amino]-1-methyl-2-oxo-ethyl]-*N*-methyl-carbamate (**S69**) (47 mg, 0.062 mmol, 1 eq.) was dissolved in MeOH (2 mL). The mixture was degassed with N_2 then treated with 10% Pd/C (3.31 mg, 0.0311 mmol) and degassed again before a balloon of Hydrogen was introduced. The mixture was stirred overnight at rt. LCMS showed incomplete conversion to the product, however addition of fresh Pd/C and H_2 did not improve the conversion. It was separated using SCX chromatography with a small pad of Celite loaded on top of the column. The ammonia fraction contained the product (35 mg, 77% yield).

^{1}H NMR (300 MHz, $CDCl_3$) δ 8.10 (s, 1H), 7.79 (dt, *J* = 7.7, 1.3 Hz, 1H), 7.69 (dd, *J* = 2.6, 1.6 Hz, 1H), 7.37 (t, *J* = 7.9 Hz, 1H), 7.16 (ddd, *J* = 8.3, 2.7, 1.1 Hz, 1H), 6.72 (bs, 1H), 5.55 (dd, *J* = 7.8, 2.6 Hz, 1H), 4.78 –

4.57 (m, 2H), 4.20 (dd, *J* = 5.6, 3.9 Hz, 2H), 3.95 – 3.61 (m, 8H), 3.54 (t, *J* = 5.2 Hz, 2H), 2.89 (t, *J* = 5.3 Hz, 2H), 2.79 (s, 3H), 2.53 – 1.98 (m, 7H), 1.85 – 1.56 (m, 6H), 1.47 (s, 9H), 1.33 (d, *J* = 7.1 Hz, 3H), 1.29 – 0.83 (m, 4H).

LCMS (Method 2) RT = 1.70 min, $[M+H]^+$ = 730.4

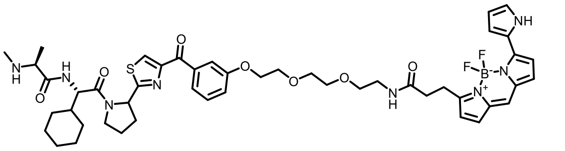

(2*S*)-*N*-[(1*S*)-1-cyclohexyl-2-[(2*S*)-2-[4-[3-[2-[2-[2-[3-[2,2-difluoro-12-(1*H*-pyrrol-2-yl)-1-aza-3-azonia-2-boranuidatricyclo[7.3.0.03,7]dodeca-3,5,7,9,11-pentaen-4-yl]propanoylamino]ethoxy]ethoxy]ethoxy]benzoyl]thiazol-2-yl]pyrrolidin-1-yl]-2-oxo-ethyl]-2-(methylamino)propenamide (B678): To a solution of (2,5-dioxopyrrolidin-1-yl) 3-[2,2-difluoro-12-(1*H*-pyrrol-2-yl)-1-aza-3-azonia-2-boranuidatricyclo[7.3.0.03,7]dodeca-3,5,7,9,11-pentaen-4-yl]propanoate (BODIPY™ 576/589 NHS Ester) (5.0 mg, 0.012 mmol, 1 eq.) and *tert*-butyl *N*-[(1*S*)-2-[[(1*S*)-2-[(2*S*)-2-[4-[3-[2-[2-(2-aminoethoxy)ethoxy]ethoxy]benzoyl]thiazol-2-yl]pyrrolidin-1-yl]-1-cyclohexyl-2-oxo-ethyl]amino]-1-methyl-2-oxo-ethyl]-*N*-methyl-carbamate (**S70**) (10 mg, 0.014 mmol, 1.1 eq.) in DMF (1 mL) was added DIPEA (5.1 µL, 0.029 mmol, 2.4 eq.) and the mixture was stirred for 1 h at rt with foil to protect from light. The solvent was co-evaporated with acetonitrile using a nitrogen line and heating to 40 °C. It was resuspended in DCM (1 mL) and cooled to 0 °C before the addition of TFA (90 µL, 1.17 mmol). The mixture was stirred at rt for 1 h. LCMS showed conversion to the desired product. The mixture was aspirated under N_2 and subjected to acidic RP HPLC. Product containing fractions were combined and freeze dried to yield product (2.0 mg, 18% yield) as a formate salt.

^{1}H NMR (300 MHz, MeOD) δ 8.27 (d, *J* = 6.1 Hz, 1H), 7.72 – 7.64 (m, 2H), 7.38 (t, *J* = 7.9 Hz, 1H), 7.19 (dt, *J* = 6.6, 2.5 Hz, 5H), 7.00 (d, *J* = 4.5 Hz, 1H), 6.89 (d, *J* = 3.9 Hz, 1H), 6.38 – 6.26 (m, 2H), 5.48 – 5.41 (m, 1H), 4.55 (d, *J* = 7.3 Hz, 1H), 4.16 (t, *J* = 4.5 Hz, 2H), 3.98 – 3.80 (m, 4H), 3.80 – 3.60 (m, 5H), 3.55 (t, *J* = 5.5 Hz, 2H), 3.37 (t, *J* = 5.3 Hz, 2H), 3.26 – 3.20 (m, 2H), 2.67 – 2.56 (m, 5H), 2.39 – 1.95 (m, 4H), 1.87 – 1.63 (m, 4H), 1.63 – 1.52 (m, 2H), 1.43 (d, *J* = 6.9 Hz, 3H), 1.27 – 0.99 (m, 6H).

LCMS (XSelect) RT = 2.66 min, $[M+H]^+$ = 941.4

HRMS (ESI+) *m/z*: $[M+H]^+$ calcd for $C_{49}H_{48}BF_5N_9O_5$ 948.3792; found 899.4490.

*Synthesis of **A472** (TEAD nanoBRET Tracer)*

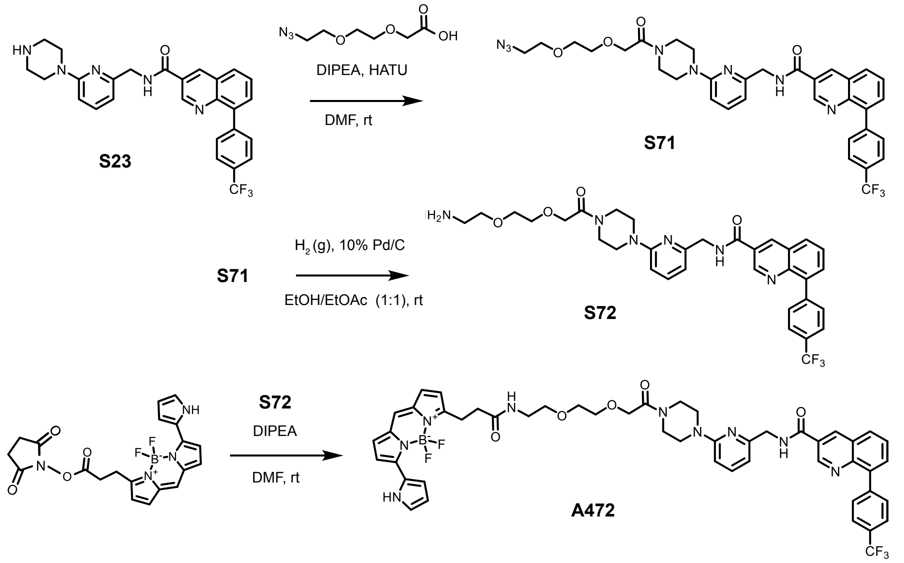

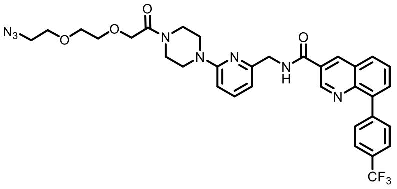

***N*-[[6-[4-[2-[2-(2-azidoethoxy)ethoxy]acetyl]piperazin-1-yl]-2-pyridyl]methyl]-8-[4-(trifluoromethyl)phenyl]quinoline-3-carboxamide (S71)**: 2-[2-(2-azidoethoxy)ethoxy]acetic acid (57.7 mg, 0.305 mmol, 1.5 eq.) and *N*-[(6-piperazin-1-yl-2-pyridyl)methyl]-8-[4-(trifluoromethyl)phenyl]quinoline-3-carboxamide (**S23**) (100 mg, 0.20 mmol, 1 eq.) was dissolved in DMF (2 mL) and DIPEA (0.11 mL, 0.61 mmol, 3 eq.) added. HATU (120 mg, 0.31 mmol, 1.5 eq.) was added and the reaction stirred at rt for 30 min. The reaction was diluted with EtOAc and water and extracted. The organic layer was washed with brine, dried with $MgSO_4$ and evaporated to give a crude residue that was separated on flash chromatography (0- 100% EtOAc in Heptane) to give product (107 mg, 79% yield).

^{1}H NMR (300 MHz, $CDCl_3$) δ 9.32 (d, *J* = 2.2 Hz, 1H), 8.75 (d, *J* = 2.3 Hz, 1H), 7.95 (d, *J* = 8.2 Hz, 1H), 7.73 (tq, *J* = 15.2, 6.6 Hz, 7H), 7.49 (t, *J* = 7.9 Hz, 1H), 6.69 (d, *J* = 7.4 Hz, 1H), 6.56 (d, *J* = 8.5 Hz, 1H), 4.68 (d, *J* = 4.9 Hz, 2H), 4.25 (s, 2H), 3.73 – 3.49 (m, 14H), 3.35 (q, *J* = 7.0 Hz, 2H).

LCMS (Method 2) RT = 2.31 min, $[M+H]^+$ = 663.4

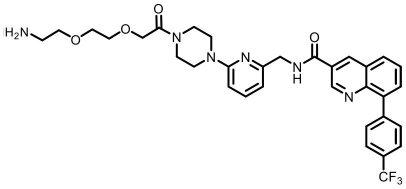

***N*-[[6-[4-[2-[2-(2-aminoethoxy)ethoxy]acetyl]piperazin-1-yl]-2-pyridyl]methyl]-8-[4-(trifluoromethyl)phenyl]quinoline-3-carboxamide (S72)**: *N*-[[6-[4-[2-[2-(2-azidoethoxy)ethoxy]acetyl]piperazin-1-yl]-2-pyridyl]methyl]-8-[4-(trifluoromethyl)phenyl]quinoline-3-carboxamide (**S71**) (107 mg, 0.161 mmol, 1 eq.) dissolved in EtOH (3 mL) and EtOAc (3 mL) and degassed with N_2. Pd/C 10% (1.7 mg, 0.016 mmol) (spatula tip) was added and the reaction was degassed again. A hydrogen balloon was fitted over the reaction mixture, purged once more with H_2 and stirred at rt for 3 d. The reaction was then purged with N_2 and filtered through a Celite pad, washing with MeOH and EtOAc. The filtrate was evaporated and extracted between EtOAc and aq. $NaHCO_3$ (pH 10). The organic layer was washed with brine, dried with $MgSO_4$ and evaporated to give a crude residue that was separated on flash chromatography (0- 5% MeOH in DCM to elute unreacted starting material, then 2/18/80 NH_3OH/MeOH/DCM to elute product) to give the desired product (62 mg, 60% yield).

^1H NMR (300 MHz, $CDCl_3$) δ 9.28 (d, *J* = 2.3 Hz, 1H), 8.75 (d, *J* = 2.3 Hz, 1H), 8.16 (t, *J* = 5.3 Hz, 1H), 7.91 (dd, *J* = 8.3, 1.6 Hz, 1H), 7.82 – 7.57 (m, 6H), 7.42 (t, *J* = 7.9 Hz, 1H), 6.66 (d, *J* = 7.4 Hz, 1H), 6.50 (d, *J* = 8.6 Hz, 1H), 4.61 (d, *J* = 5.1 Hz, 2H), 4.22 (s, 2H), 3.73 – 3.43 (m, 14H), 2.92 (t, *J* = 5.3 Hz, 2H).

LCMS (Method 2) RT = 1.77 min, [M+H] + = 637.4

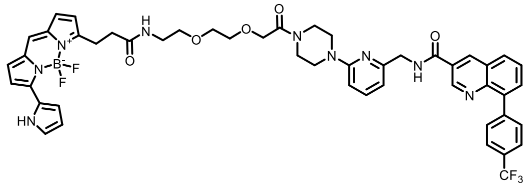

***N*-[[6-[4-[2-[2-[2-[3-[2,2-difluoro-12-(1*H*-pyrrol-2-yl)-1-aza-3-azonia-2-boranuidatricyclo[7.3.0.03,7]dodeca-3,5,7,9,11-pentaen-4-yl]propanoylamino]ethoxy]ethoxy]acetyl]piperazin-1-yl]-2-pyridyl]methyl]-8-[4-(trifluoromethyl)phenyl]quinoline-3-carboxamide (A472)**: (2,5-dioxopyrrolidin-1-yl) 3-[2,2-difluoro-12-(1*H*-pyrrol-2-yl)-1-aza-3-azonia-2-boranuidatricyclo[7.3.0.03,7]dodeca-3,5,7,9,11-pentaen-4-yl]propanoate (BODIPY™ 576/589 NHS Ester) (5.0 mg, 0.012 mmol, 1 eq.) and *N*-[[6-[4-[2-[2-(2-aminoethoxy)ethoxy]acetyl]piperazin-1-yl]-2-pyridyl]methyl]-8-[4-(trifluoromethyl)phenyl]quinoline-3-carboxamide (**S72**) (11 mg, 0.018 mmol, 1.5 eq.) were dissolved in DMF (0.5 mL) in a brown glass vial. DIPEA (0.0051 mL, 0.029 mmol, 2.4 eq.) was added and the reaction was stirred at rt for 1 h. After this, the solvent was removed on aspirator, azeotroping 2x with acetonitrile. The residue was purified by prepLCMS and the clean fractions were combined and lyophilized to give the desired product as a dark blue solid (7.0 mg, 60% yield).

^1H NMR (300 MHz, $CDCl_3$) δ 10.35 (bs, 1H), 9.30 (d, *J* = 2.3 Hz, 1H), 8.76 (d, *J* = 2.4 Hz, 1H), 8.05 (s, 1H), 7.96 (dd, *J* = 8.1, 1.7 Hz, 1H), 7.84 – 7.63 (m, 6H), 7.49 (dd, *J* = 8.4, 7.3 Hz, 1H), 7.12 (q, *J* = 2.8 Hz, 1H),

6.95 (d, *J* = 4.6 Hz, 1H), 6.93 – 6.84 (m, 3H), 6.81 – 6.67 (m, 3H), 6.48 (d, *J* = 8.4 Hz, 1H), 6.30 (dt, *J* = 4.3, 2.3 Hz, 1H), 6.25 (d, *J* = 3.9 Hz, 1H), 4.68 (d, *J* = 4.8 Hz, 2H), 4.18 (s, 2H), 3.63 (d, *J* = 9.5 Hz, 6H), 3.59 – 3.34 (m, 10H), 3.29 (t, *J* = 7.7 Hz, 2H), 2.70 – 2.59 (m, 2H).

^{19}F NMR (282 MHz, $CDCl_3$) δ -62.41, -139.91 (dd, *J* = 69.2, 32.7 Hz).

LCMS (Method 2) RT = 2.96 min, [M+H] + = 948.4

HRMS (ESI+) *m/z:* $[M+H]^+$ calcd for $C_{49}H_{48}BF_5N_9O_5$ 948.3792; found 948.3778.

Spectra and Analytical Data

A250

a. ^{1}H-NMR

300 MHz, $CDCl_3$

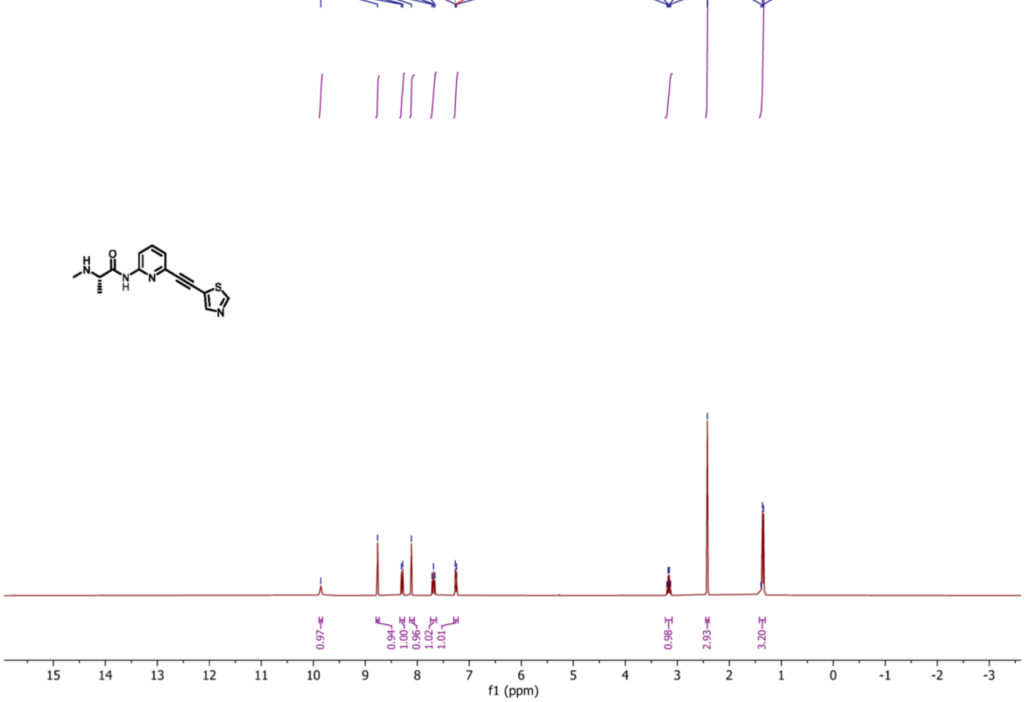

b. ^{13}C-NMR

75 MHz, $CDCl_3$

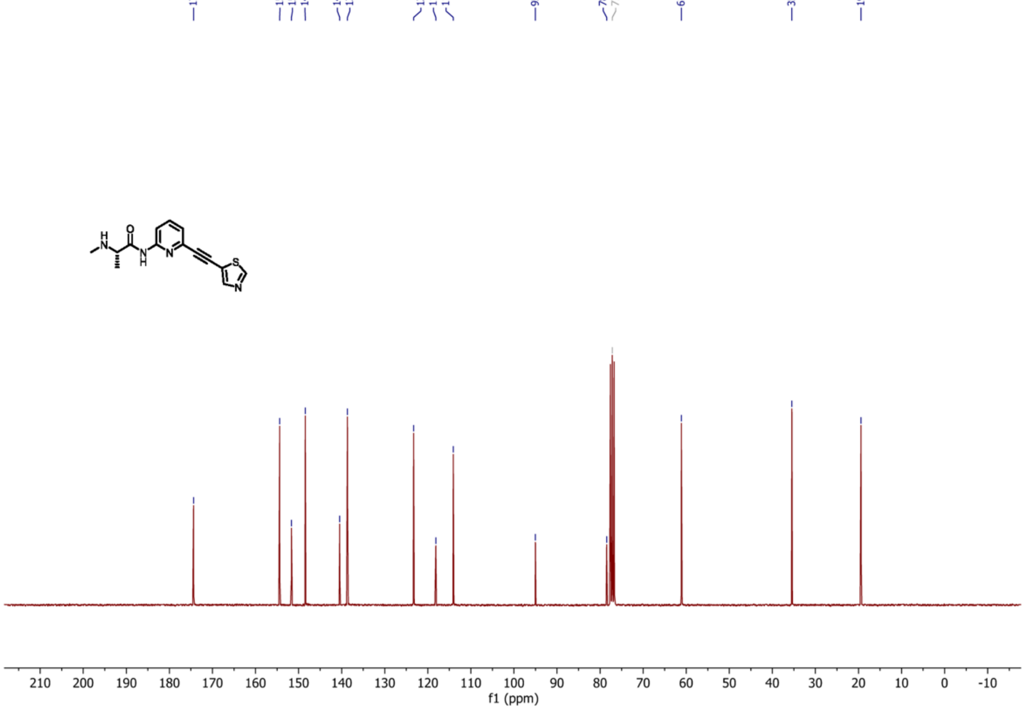

A250

c. LCMS

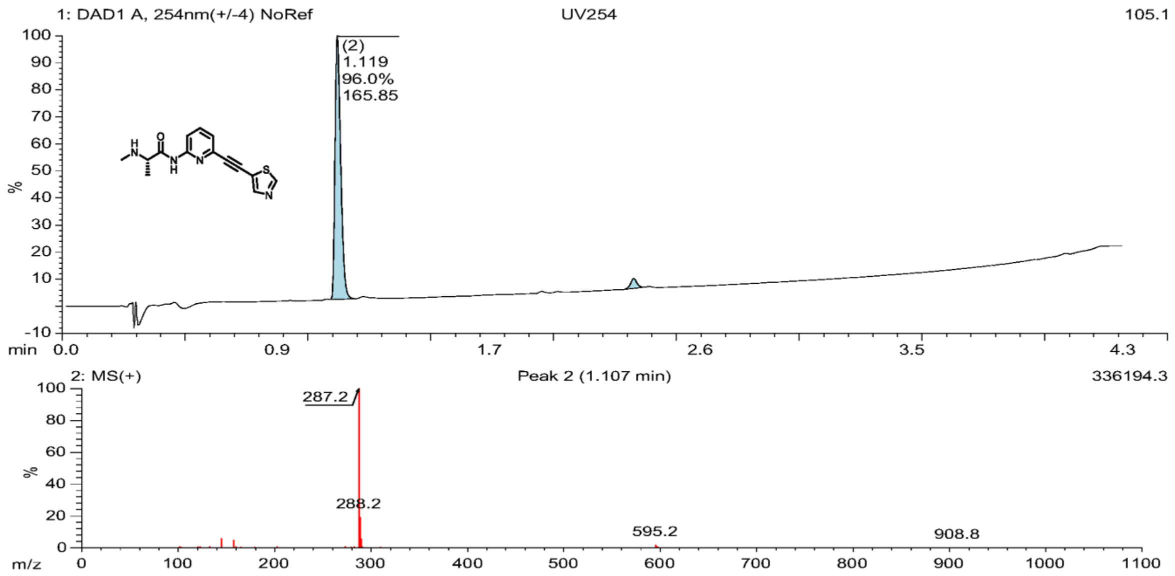

d. HRMS

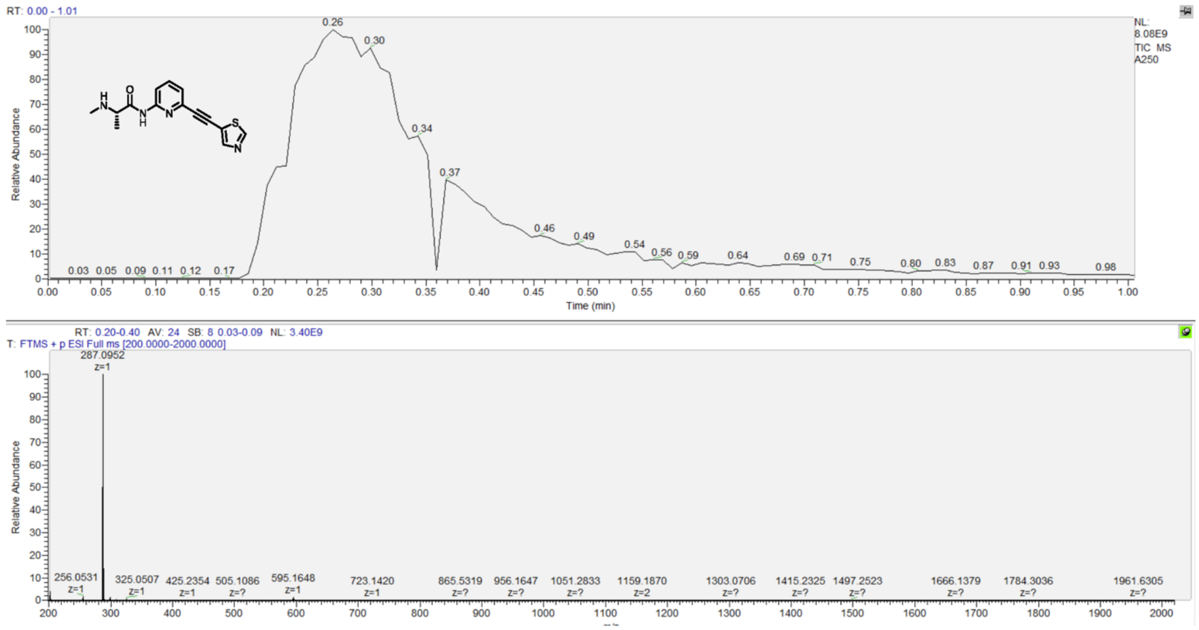

A171

a. ^{1}H-NMR

300 MHz, DMSO-d_6

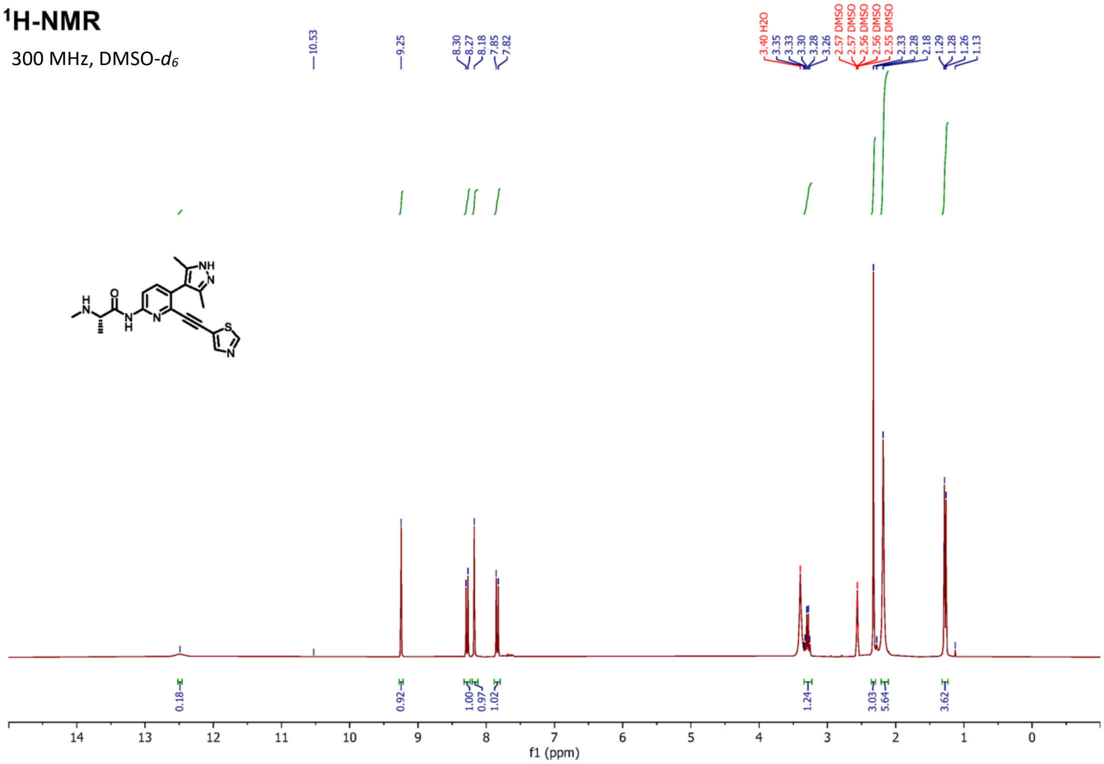

b. ^{13}C-NMR

75 MHz, DMSO-d_6

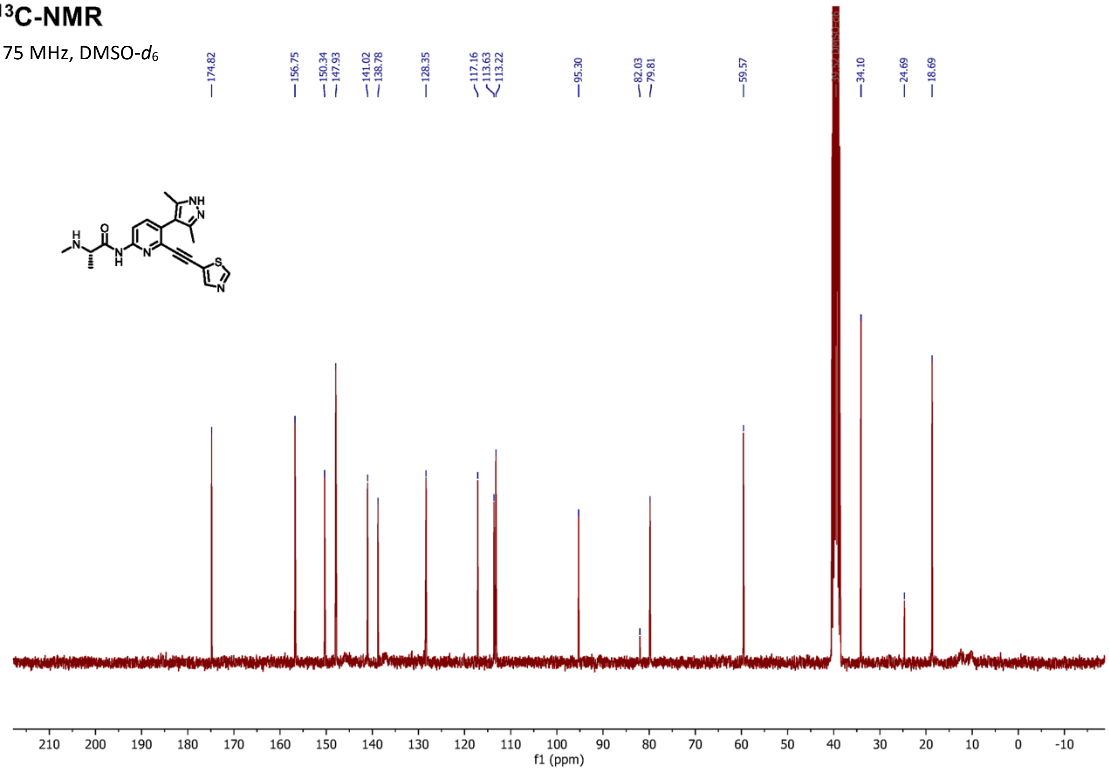

A171

c. LCMS

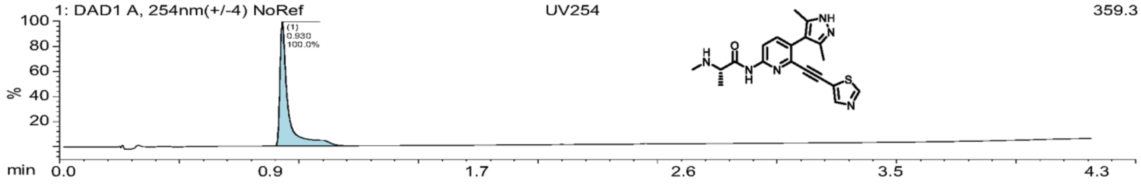

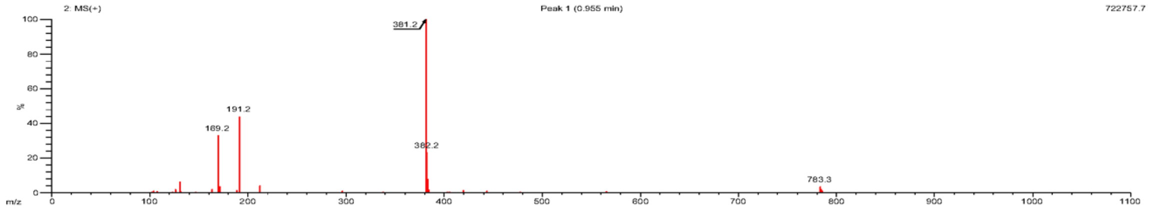

d. HRMS

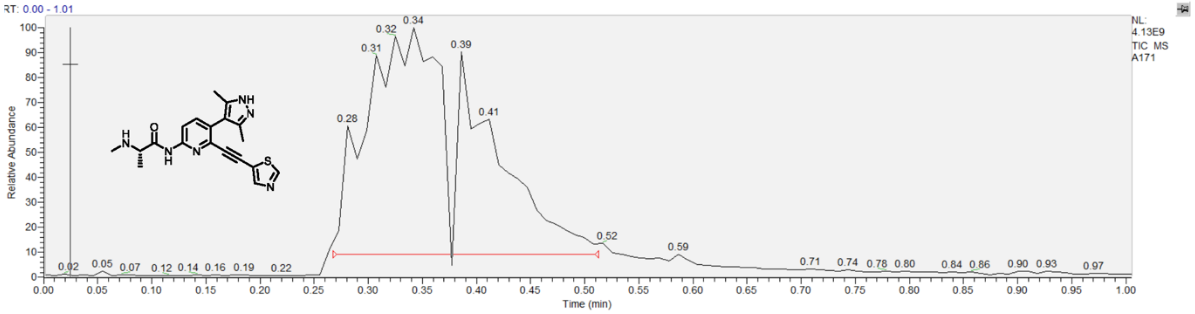

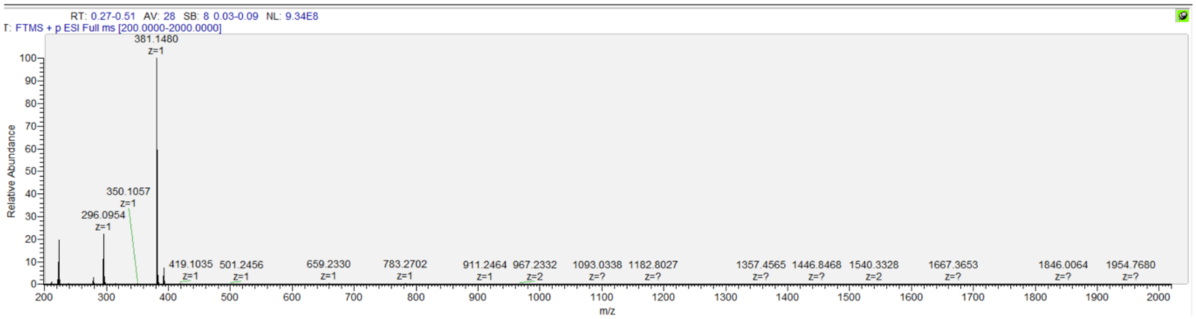

A238

a. ^{1}H-NMR

300 MHz, MeOD

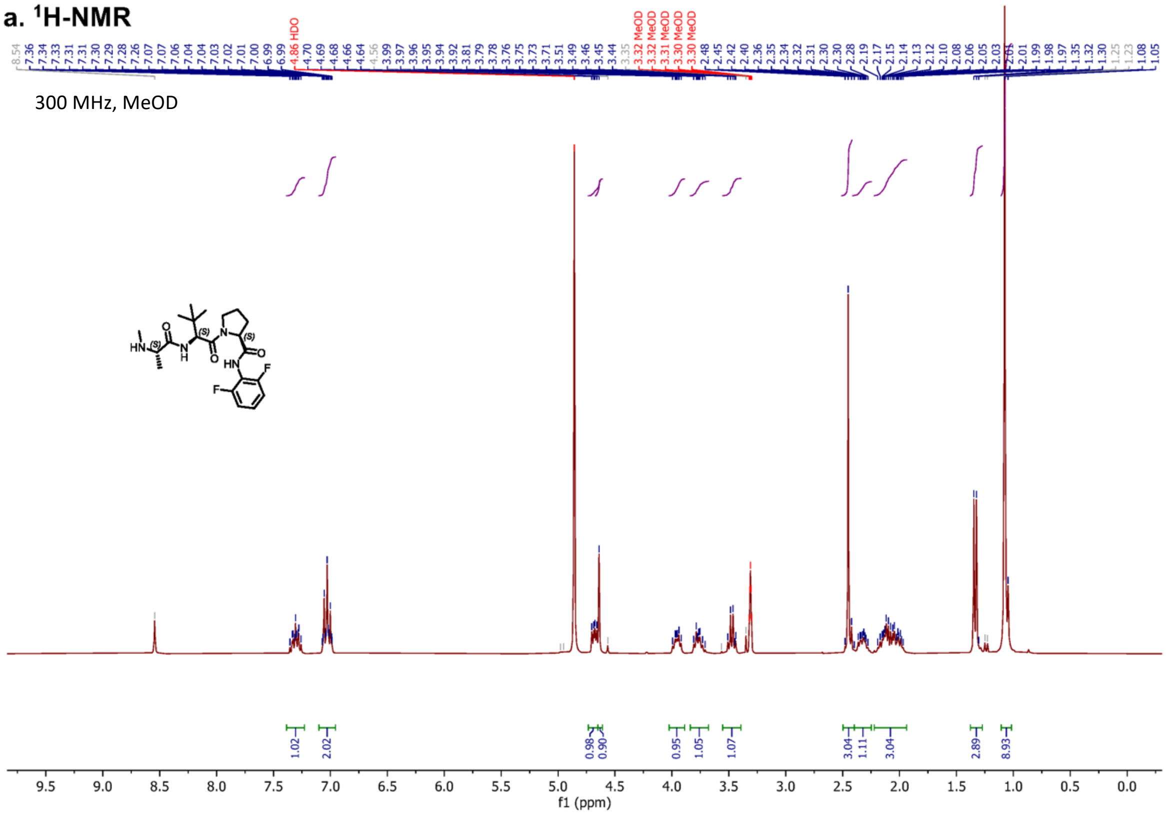

b. ^{13}C-NMR

75 MHz, MeOD

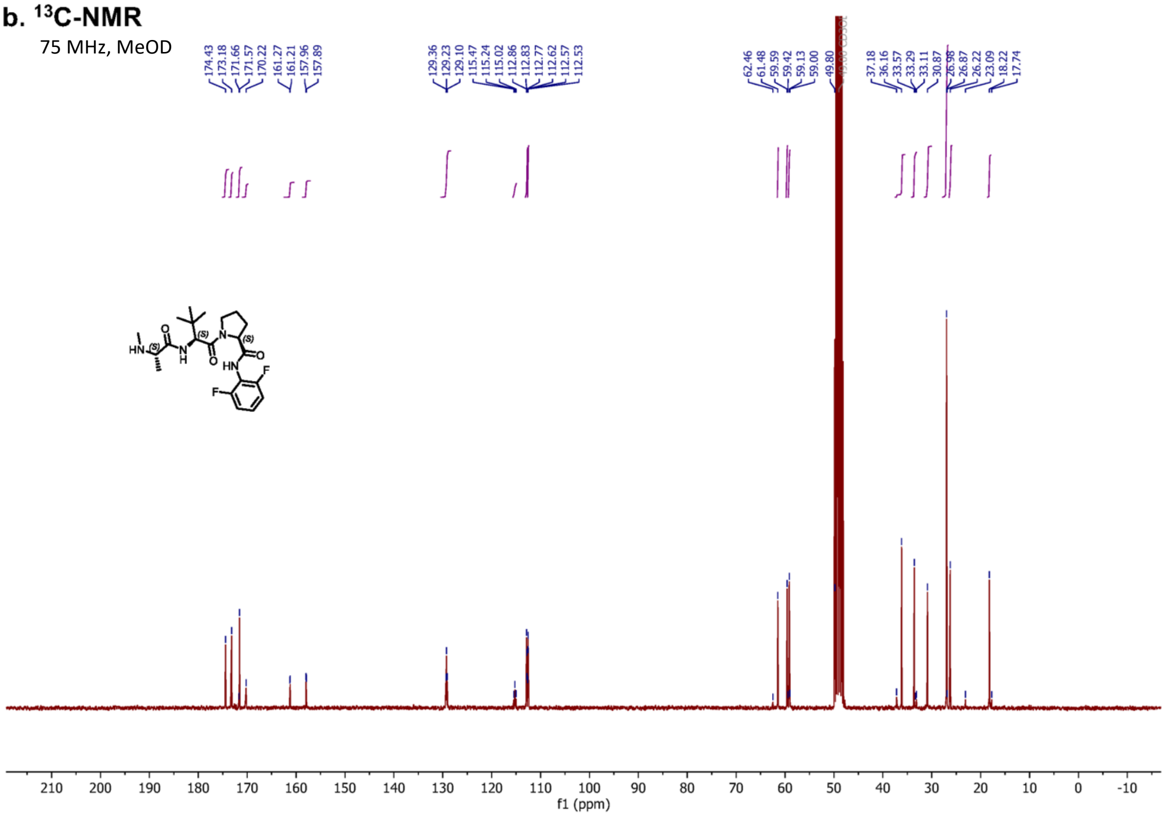

A238

c. ^{19}F-NMR

282 MHz, MeOD

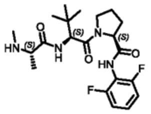

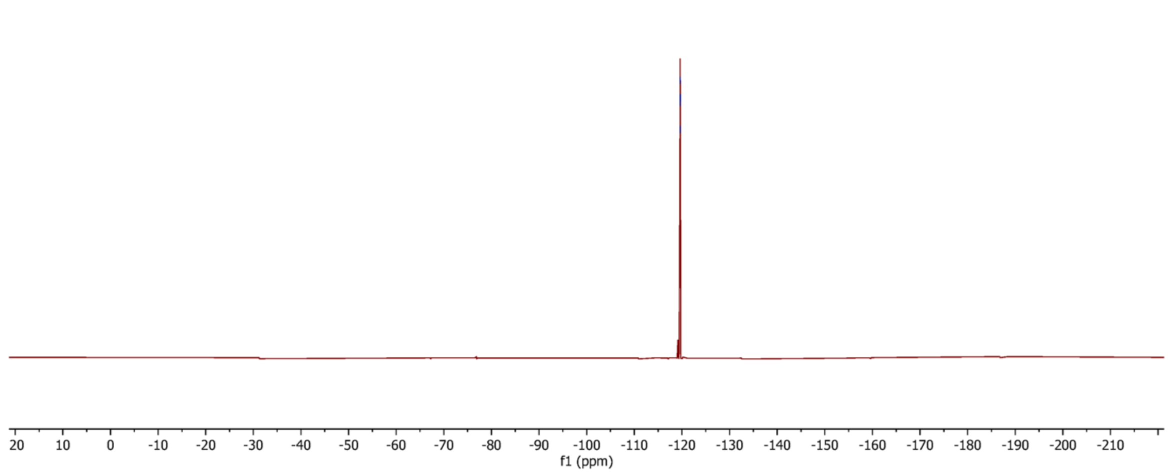

d. LCMS

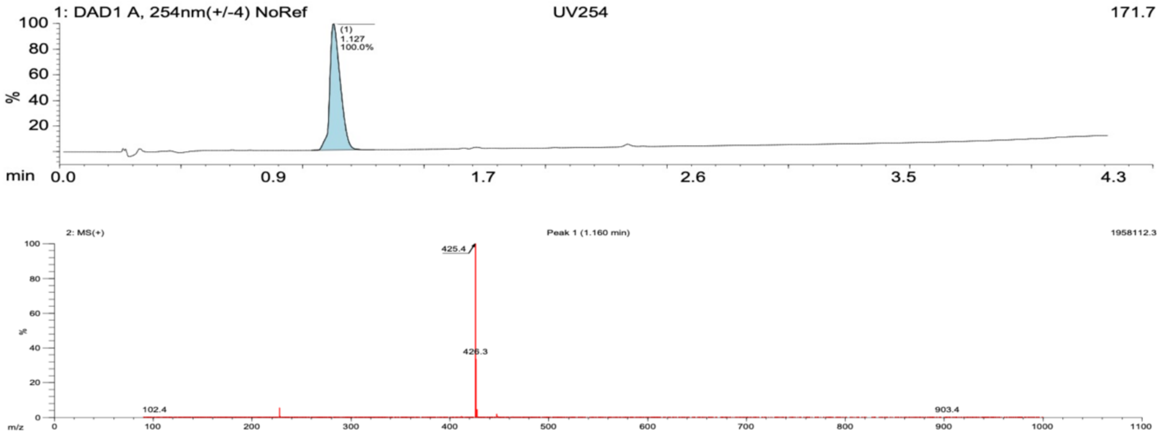

e. HRMS

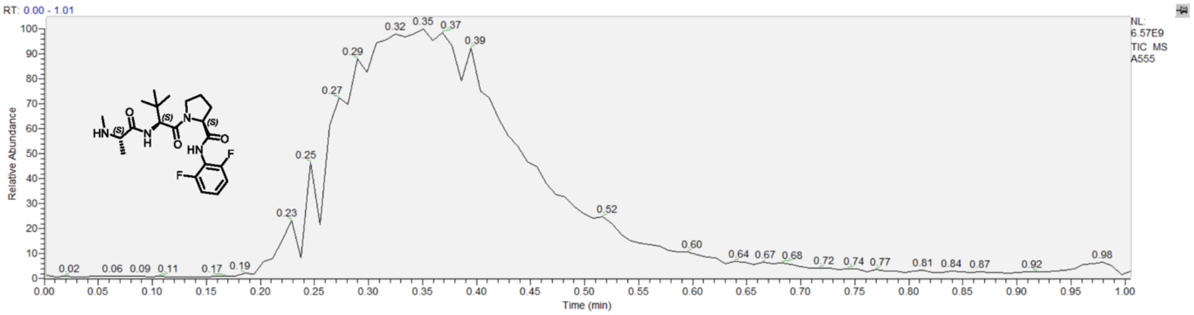
RT: 0.00 - 1.01
NL: 6.57E9 TIC MS A555
Relative Abundance
Time (min)
0.02 0.06 0.09 0.11 0.17 0.19 0.23 0.25 0.27 0.29 0.32 0.35 0.37 0.39 0.52 0.60 0.64 0.67 0.68 0.72 0.74 0.77 0.81 0.84 0.87 0.92 0.98

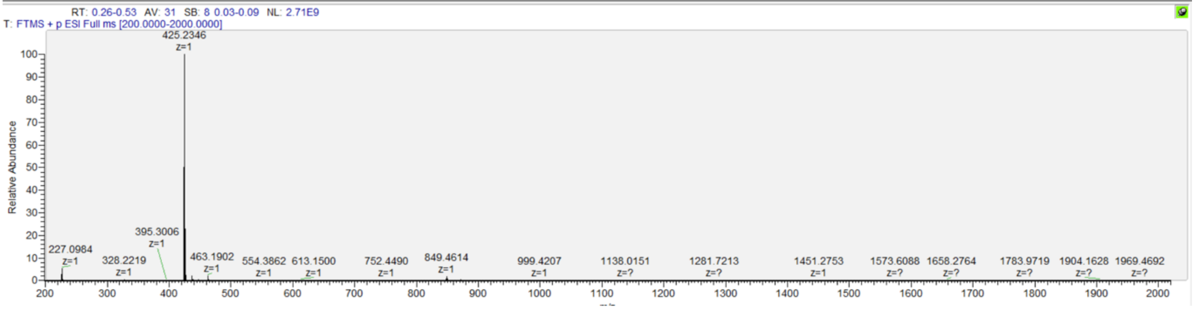
RT: 0.26-0.53 AV: 31 SB: 8 0.03-0.09 NL: 2.71E9
T: FTMS + p ESI Full ms [200.0000-2000.0000]
Relative Abundance
425.2346 z=1
395.3006 z=1
227.0984 z=1
328.2219 z=1
463.1902 z=1
554.3862 z=1
613.1500 z=1
752.4490 z=1
849.4614 z=1
999.4207 z=1
1138.0151 z=?
1281.7213 z=?
1451.2753 z=1
1573.6088 z=?
1658.2764 z=?
1783.9719 z=?
1904.1628 z=?
1969.4692 z=?

L118

a. ¹H-NMR

400 MHz, MeOD

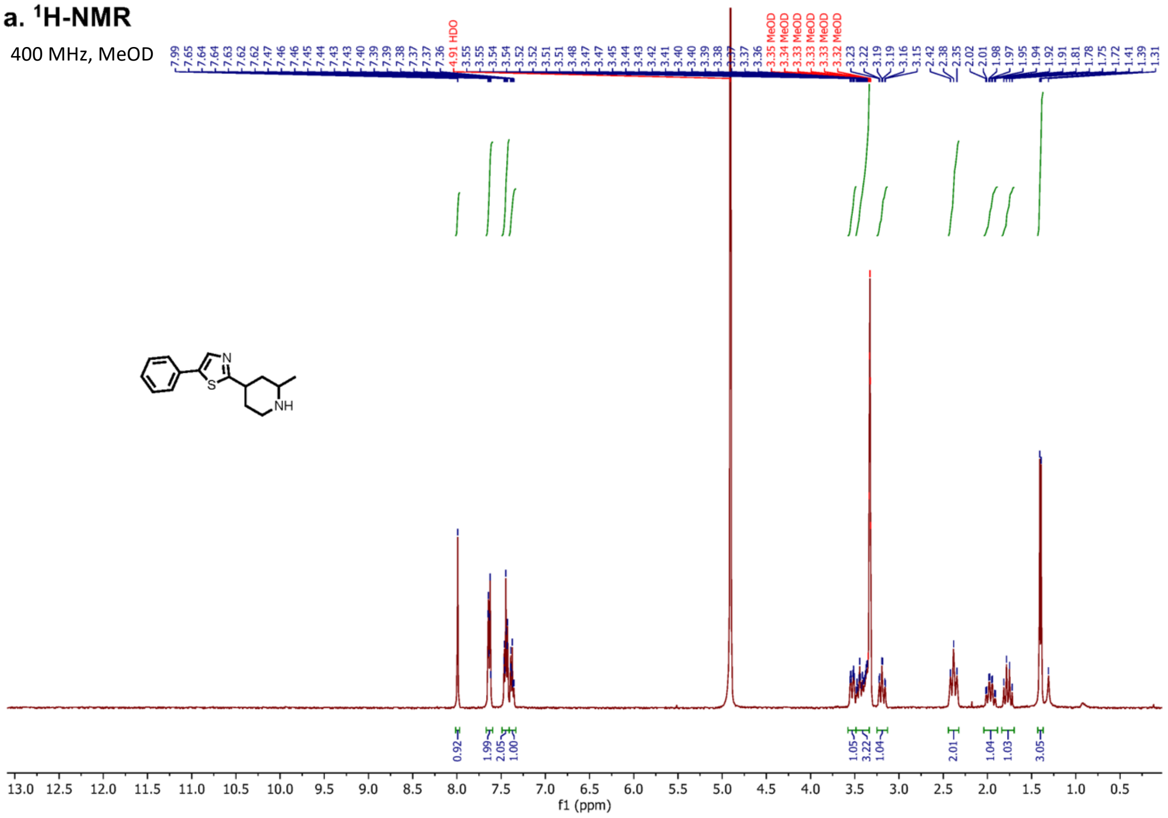

b. LCMS

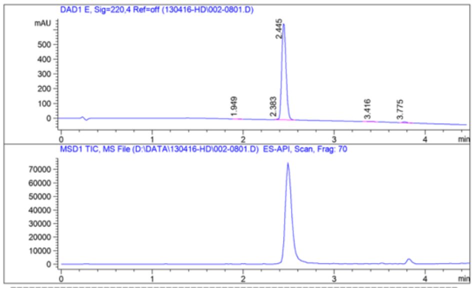

Integration Result

Signal 1 : DAD1 E, Sig=220,4 Ref=off

Peak #	RT [min]	Area	Height	Height %	Width [min]	Area %
1	1.949	3.991	1.292	0.192	0.051	0.185
2	2.383	5.266	3.701	0.550	0.024	0.244
3	2.445	2116.104	658.794	97.840	0.054	98.014
4	3.416	16.235	3.260	0.484	0.083	0.752
5	3.775	17.386	6.293	0.935	0.046	0.805

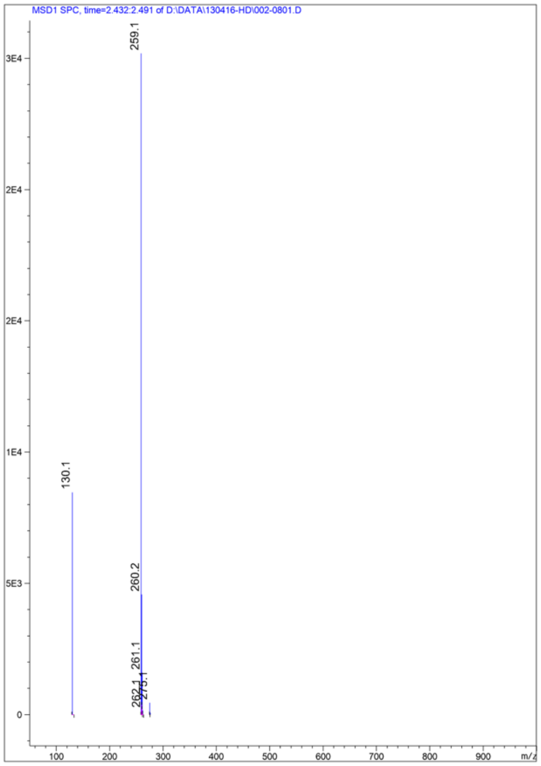

A273

a. ^{1}H-NMR

300 MHz, DMSO-d_6

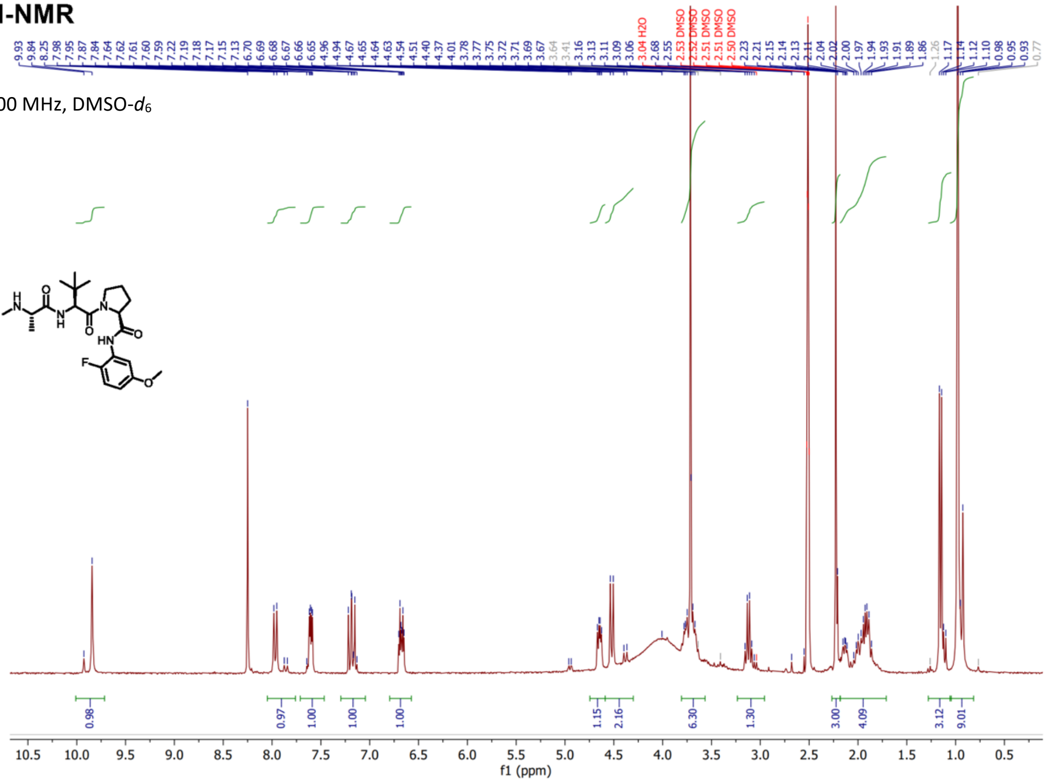

b. ^{13}C-NMR

75 MHz, DMSO-d_6

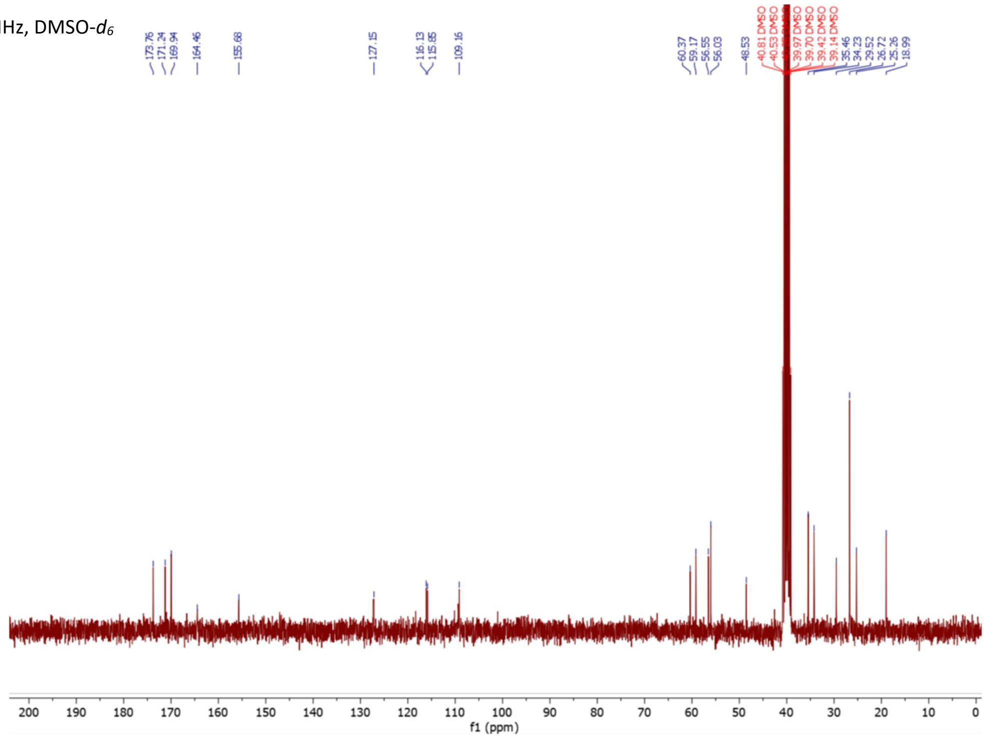

A273

c. ^{19}F-NMR

282 MHz, DMSO-d_6

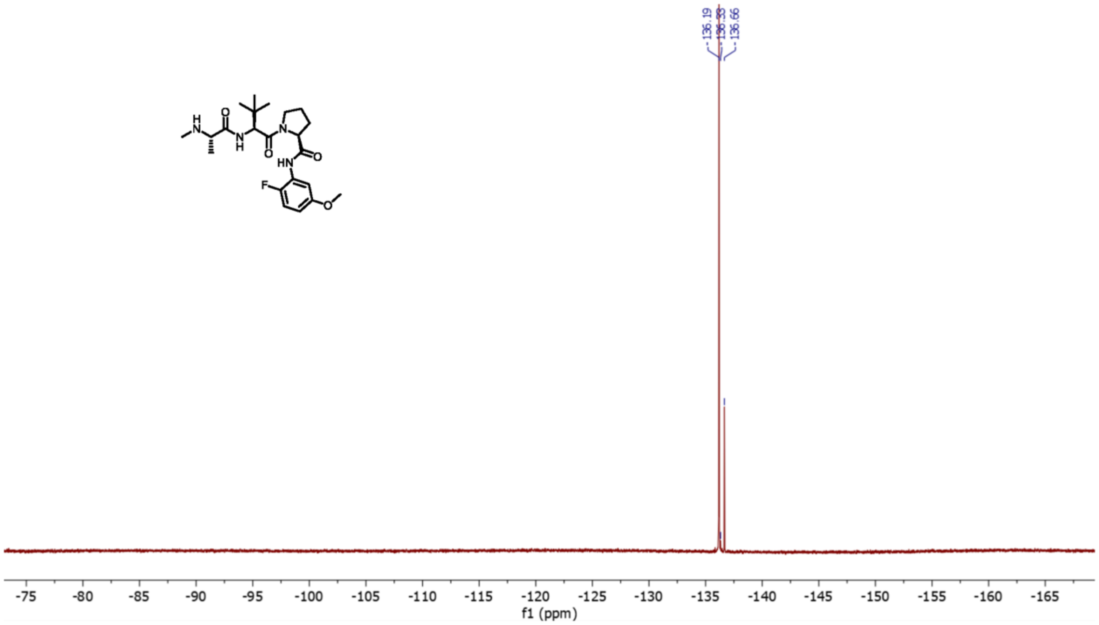

d. LCMS

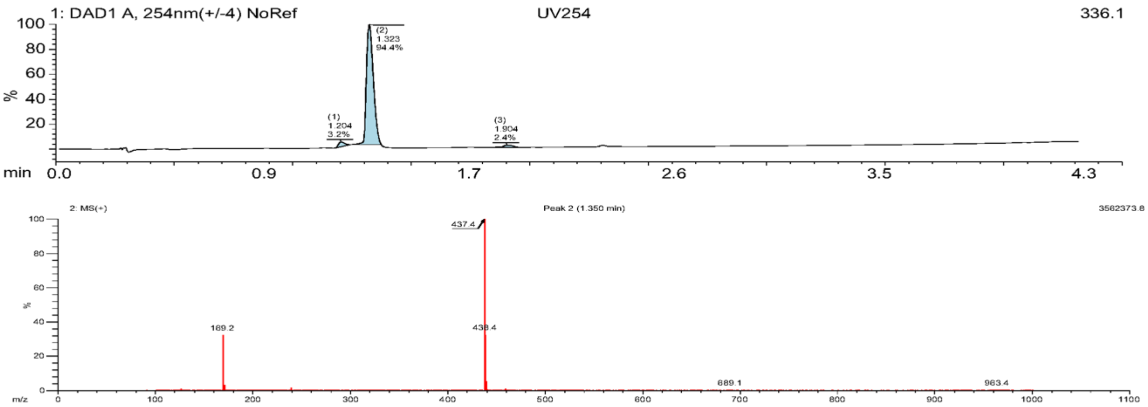

A262

a. ^{1}H-NMR

300 MHz, $CDCl_3$

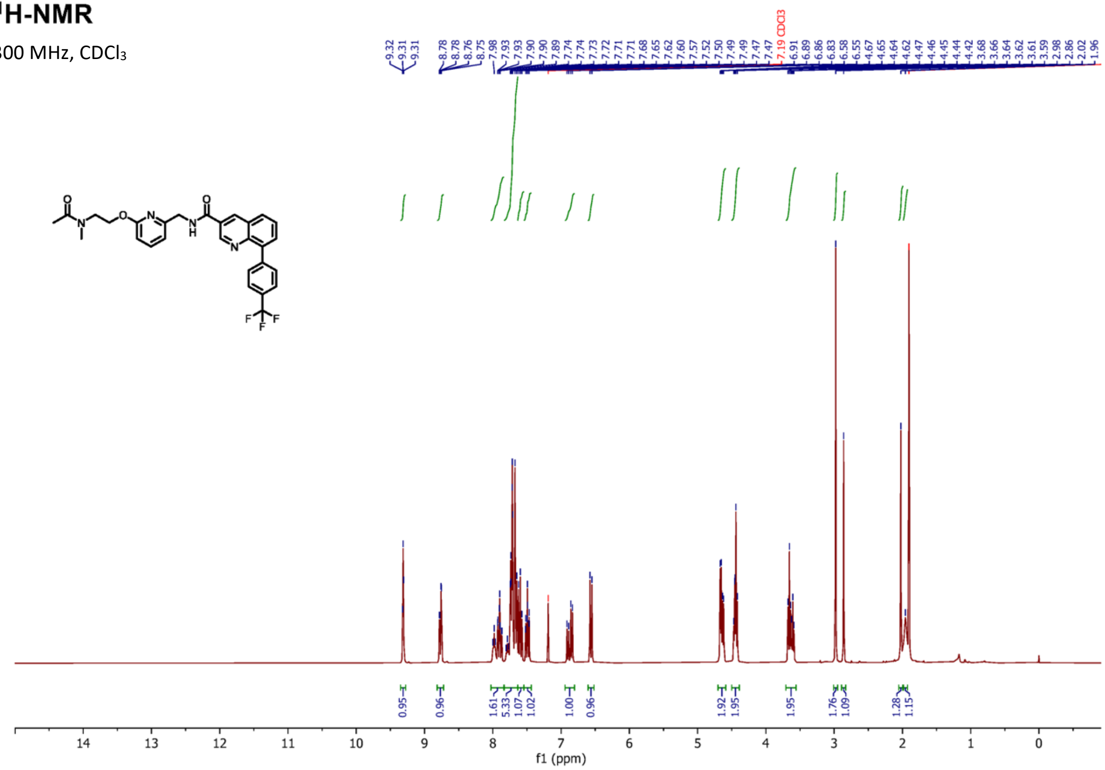

b. ^{13}C-NMR

75 MHz, $CDCl_3$

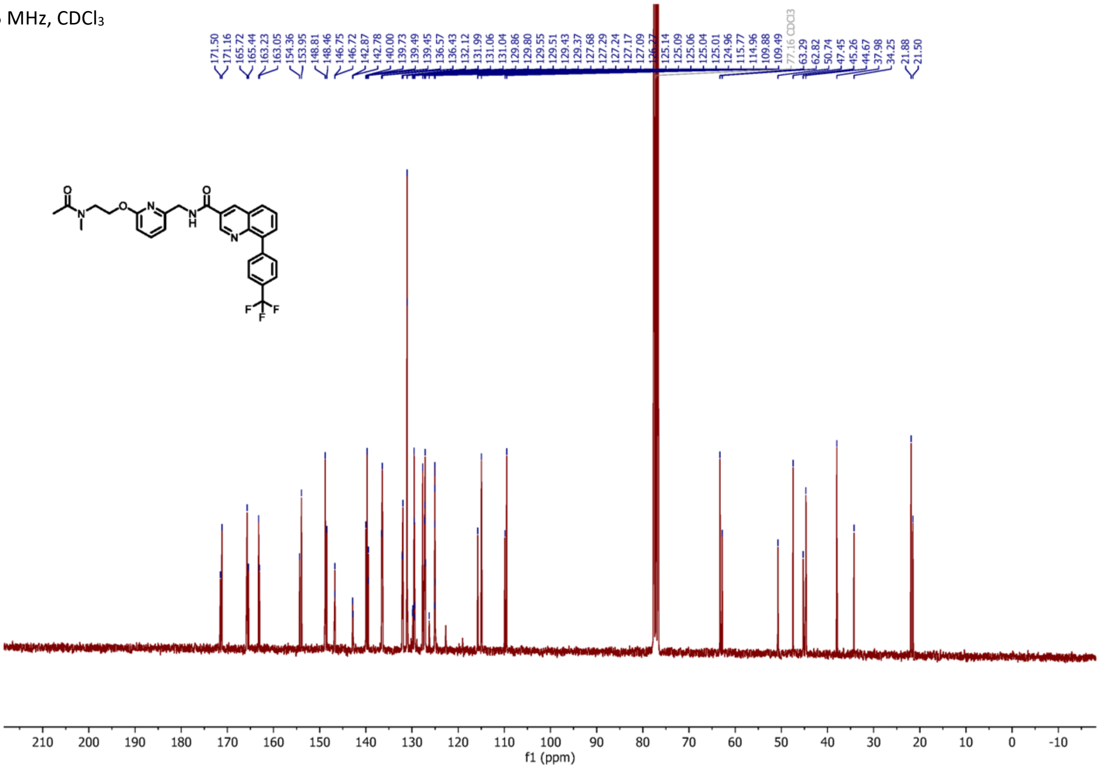

A262

c. ^{19}F-NMR

282 MHz, $CDCl_3$

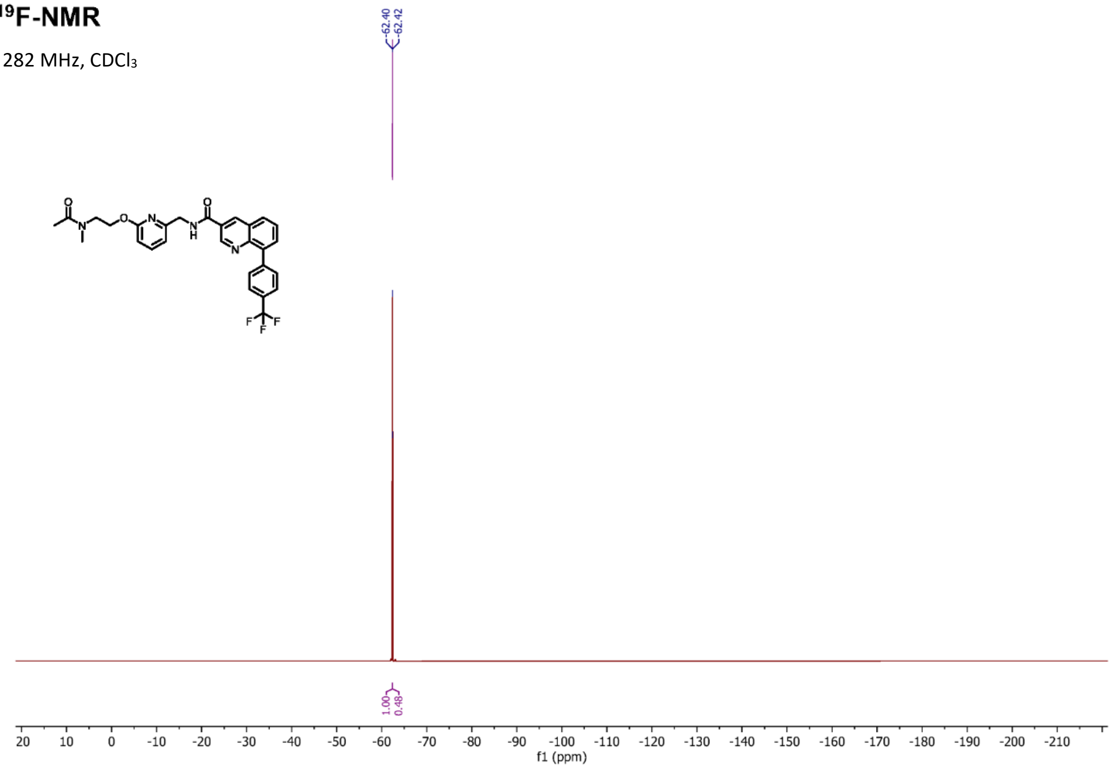

d. LCMS

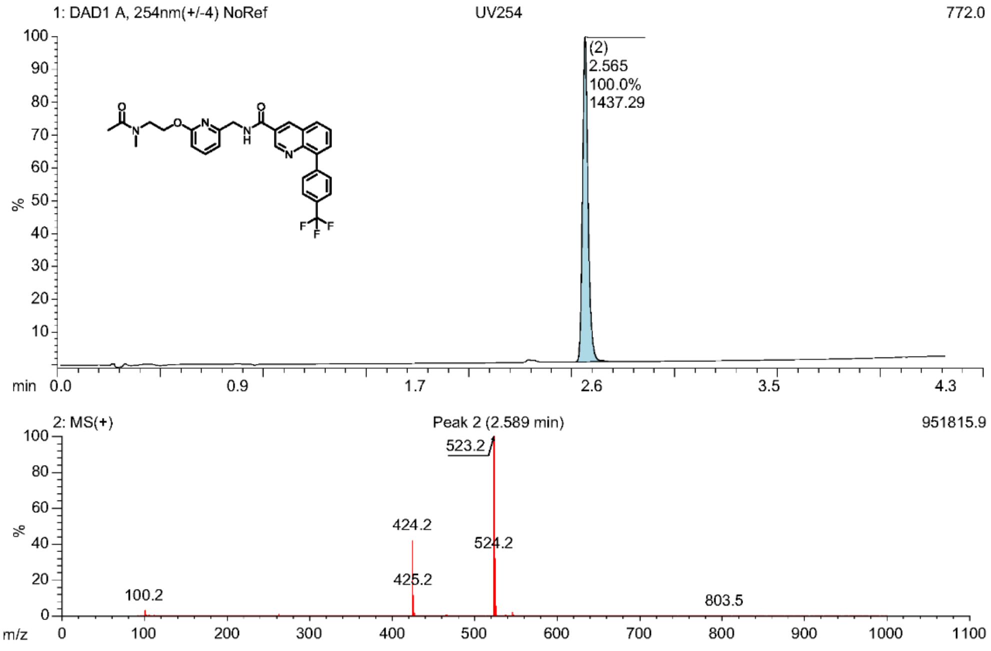

A262

e. HRMS

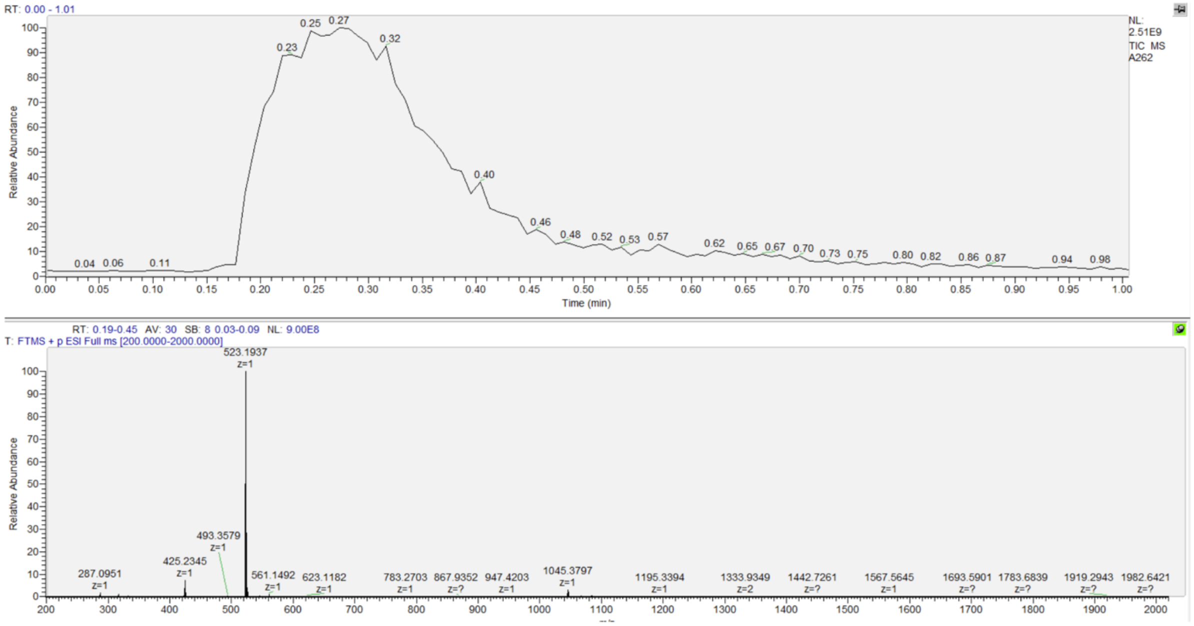
RT: 0.00 - 1.01
NL: 2.51E9 TIC MS A262
Relative Abundance
Time (min)
RT: 0.19-0.45 AV: 30 SB: 8 0.03-0.09 NL: 9.00E8
T: FTMS + p ESI Full ms [200.0000-2000.0000]
523.1937 z=1
493.3579 z=1
425.2345 z=1
287.0951 z=1
561.1492 z=1
623.1182 z=1
783.2703 z=1
867.9352 z=?
947.4203 z=1
1045.3797 z=1
1195.3394 z=1
1333.9349 z=2
1442.7261 z=?
1567.5645 z=1
1693.5901 z=?
1783.6839 z=?
1919.2943 z=?
1982.6421 z=?

A341

a. ^{1}H-NMR

300 MHz, $CDCl_3$

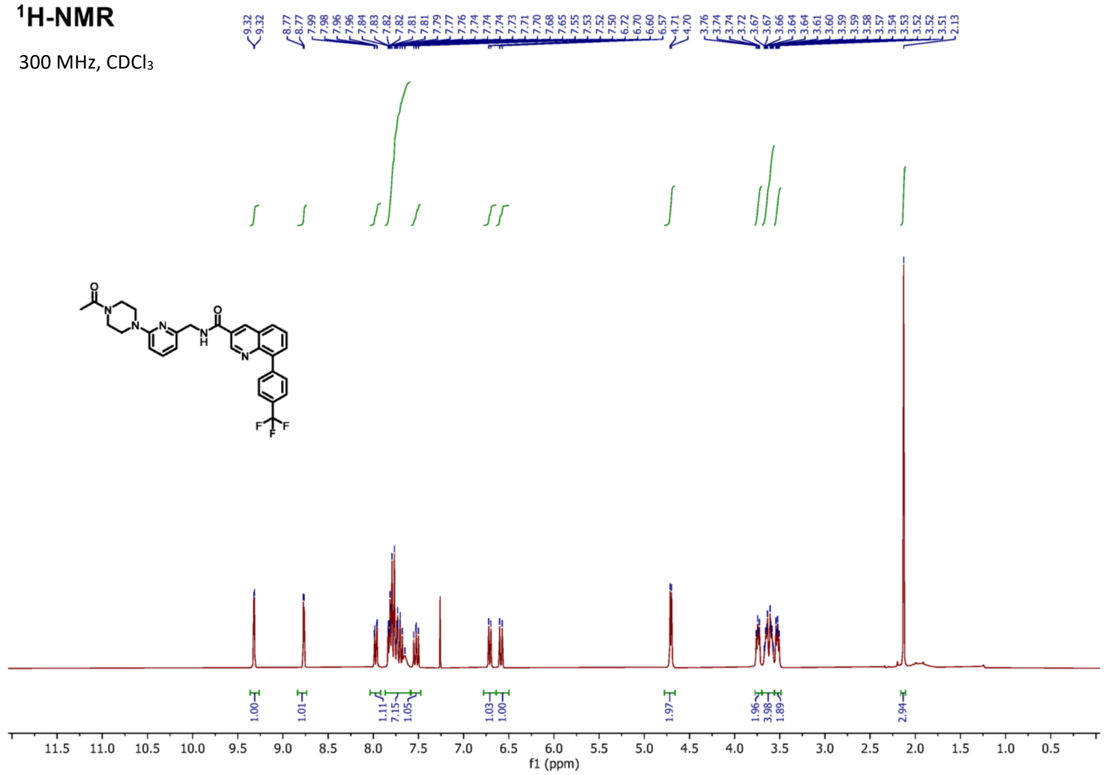

b. ^{13}C-NMR

75 MHz, $CDCl_3$

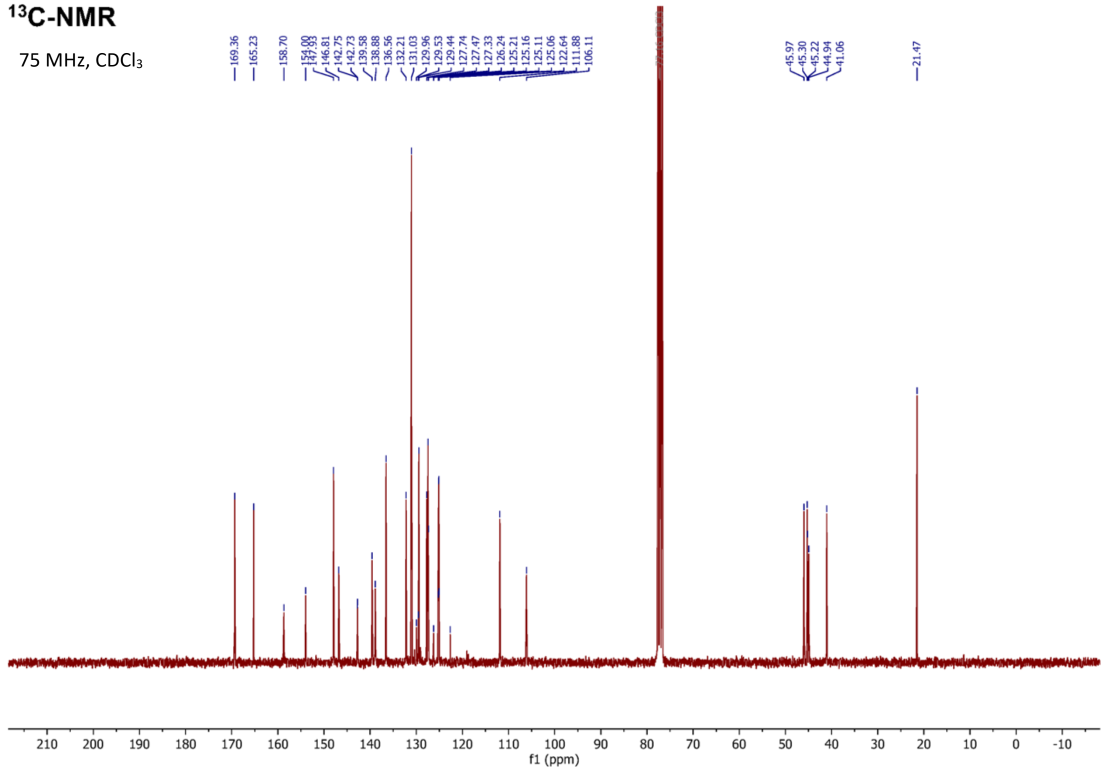

A341

c. ^{19}F-NMR

282 MHz, $CDCl_3$

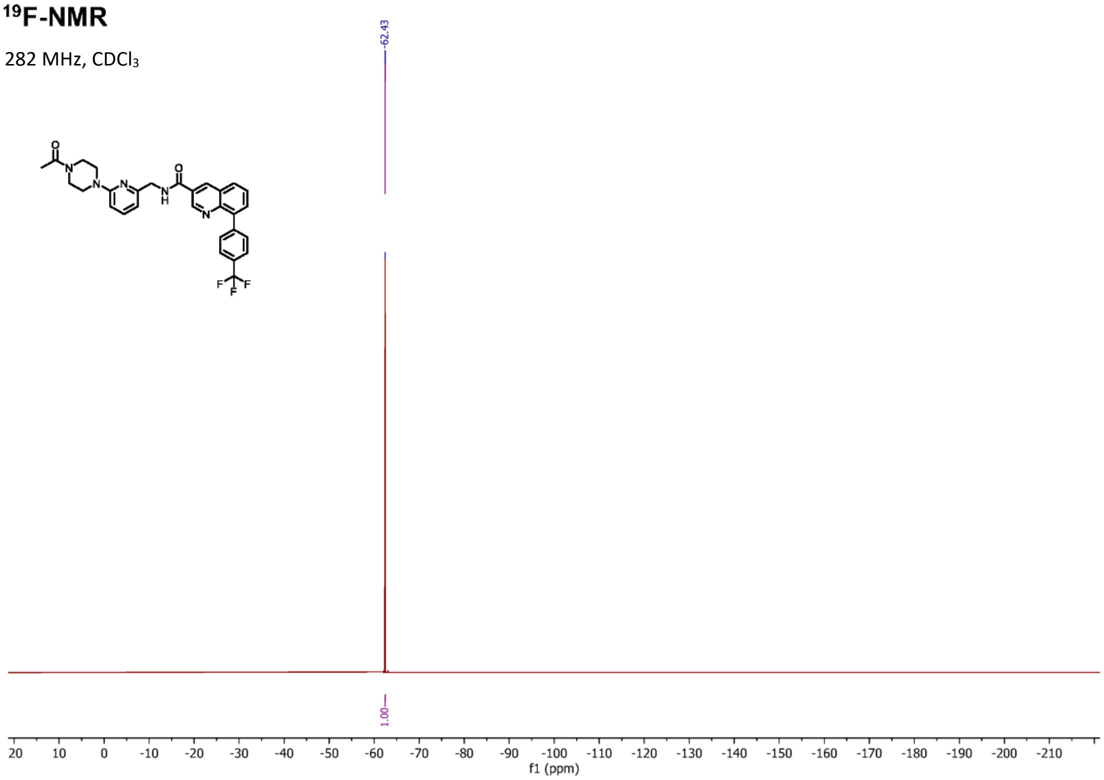

d. LCMS

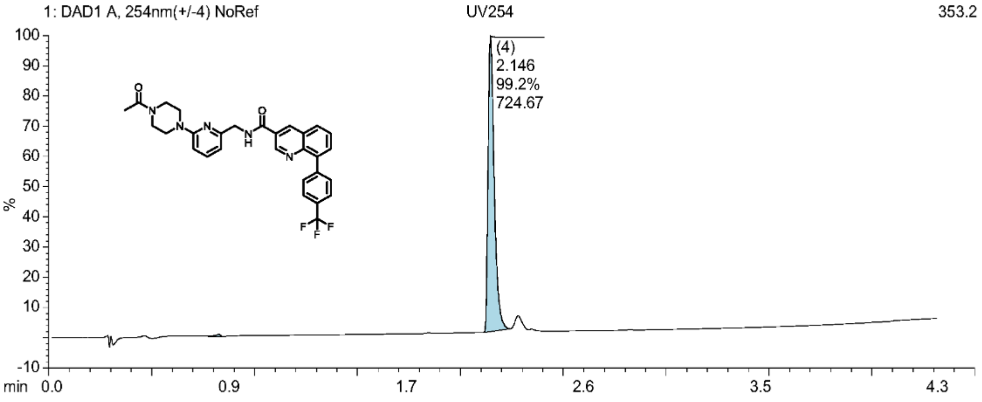

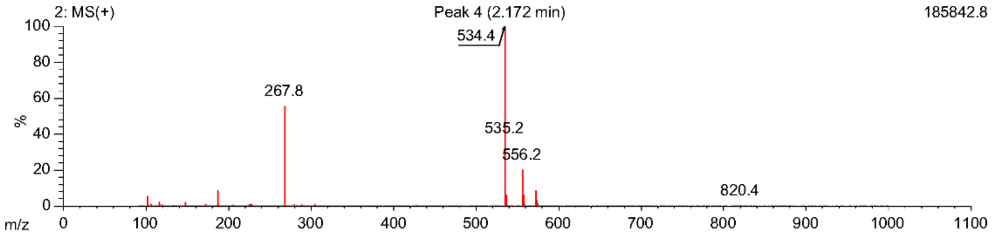

A341

e. HRMS

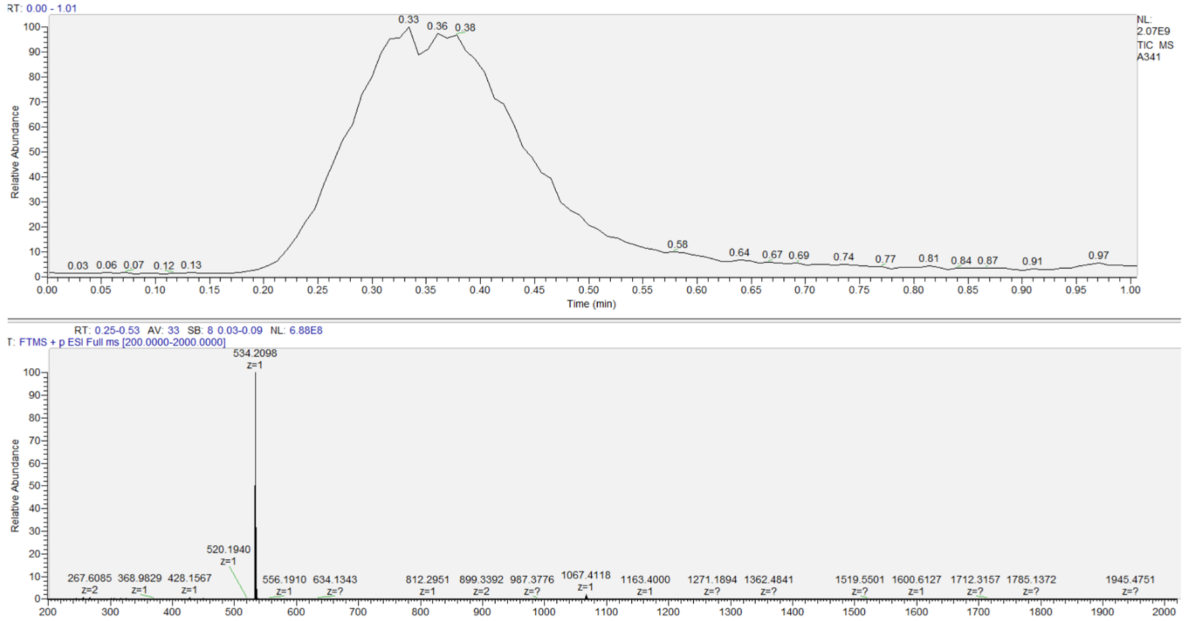

RT: 0.00 - 1.01
NL: 2.07E9 TIC MS A341
Relative Abundance
Time (min)
RT: 0.25-0.53 AV: 33 SB: 8 0.03-0.09 NL: 6.88E8
T: FTMS + p ESI Full ms [200.0000-2000.0000]
534.2098 z=1
520.1940 z=1

A531

a. ^{1}H-NMR

300 MHz, MeOD

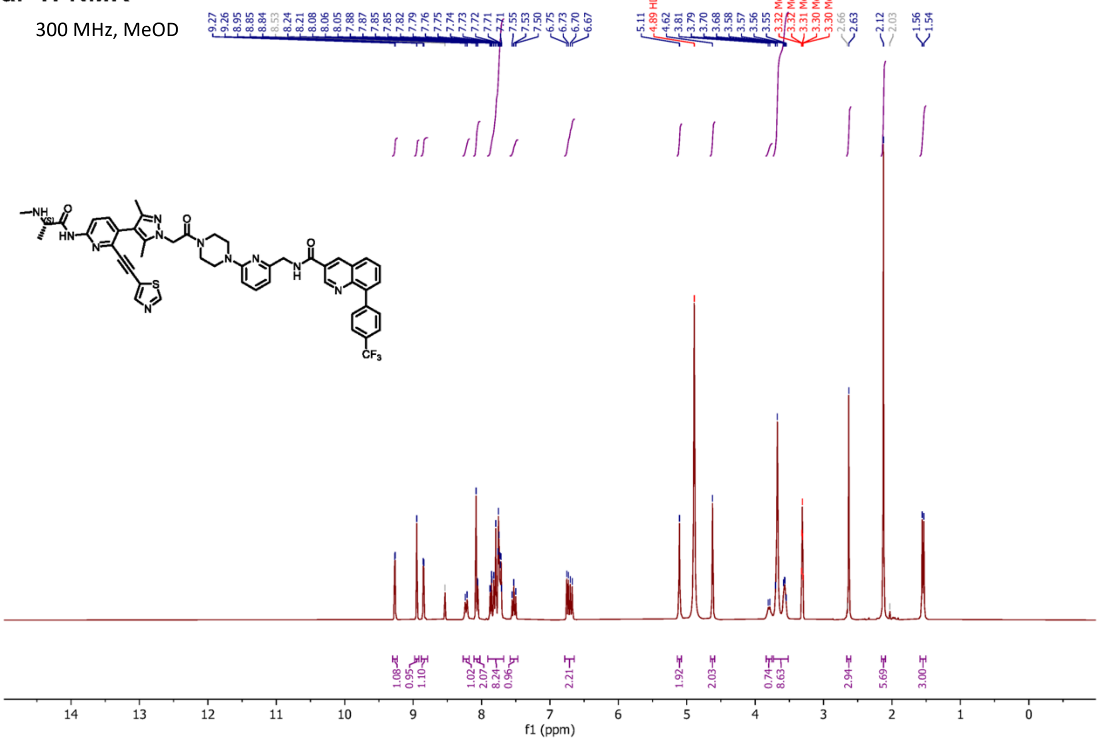

b. ^{13}C-NMR

75 MHz, MeOD

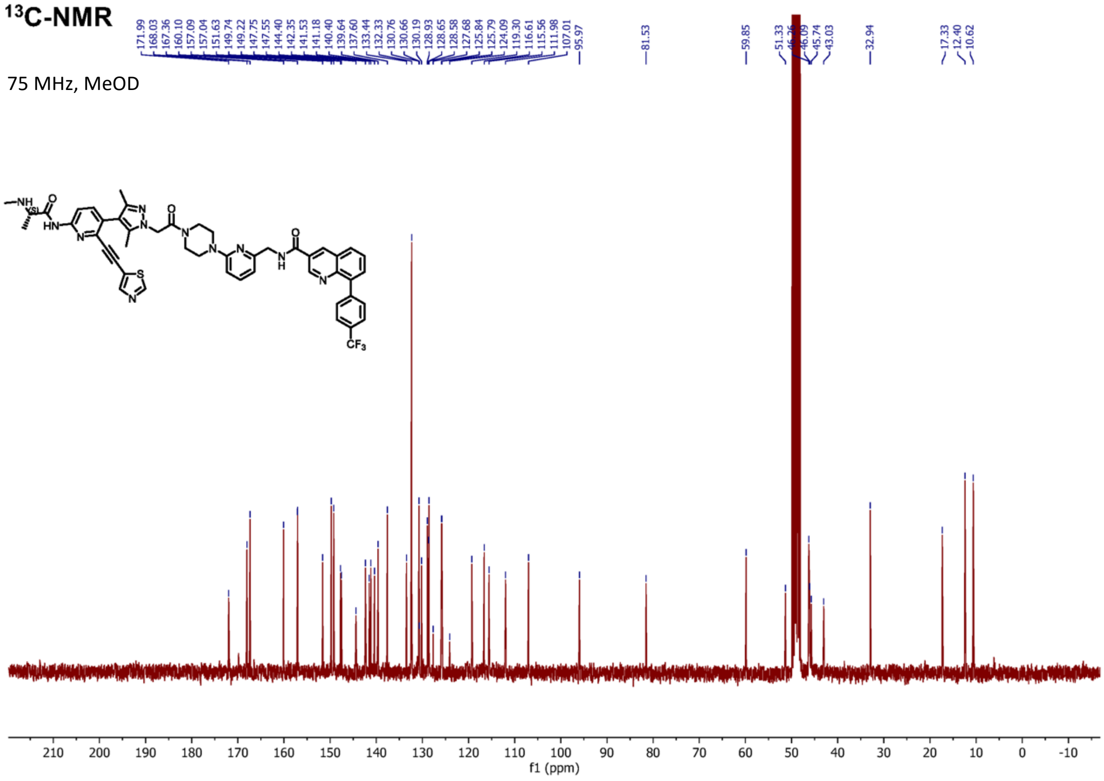

A531

c. ^{19}F-NMR

282 MHz, MeOD

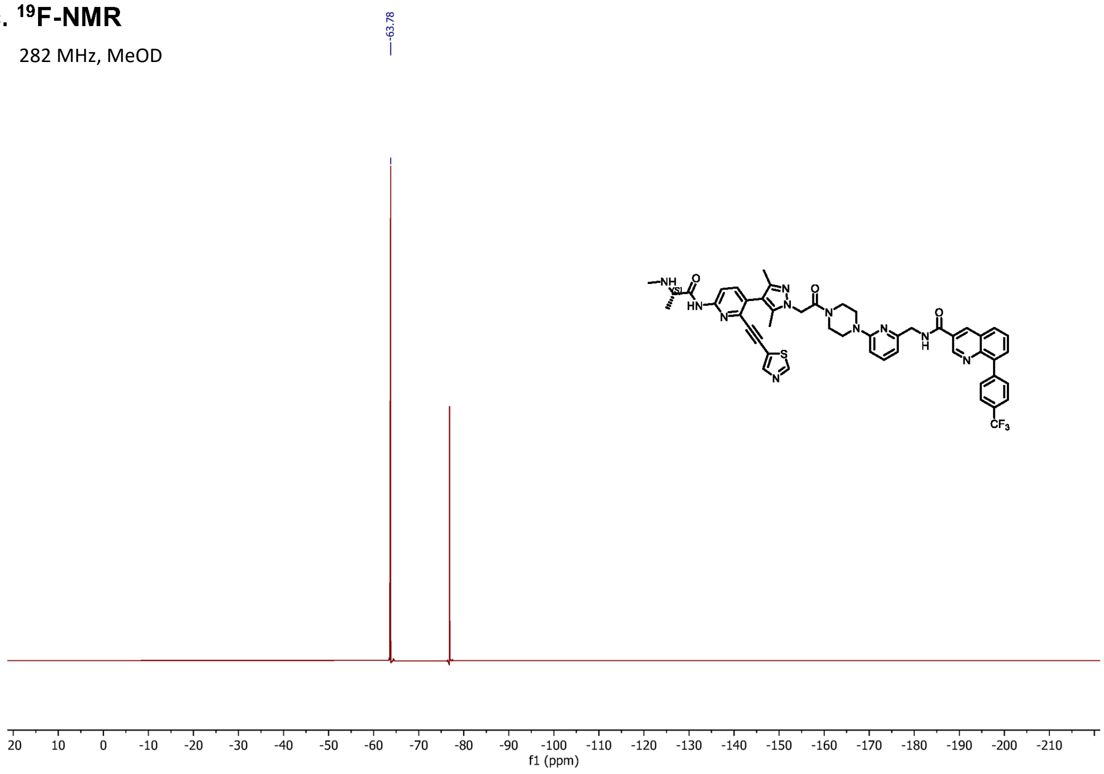

d. LCMS

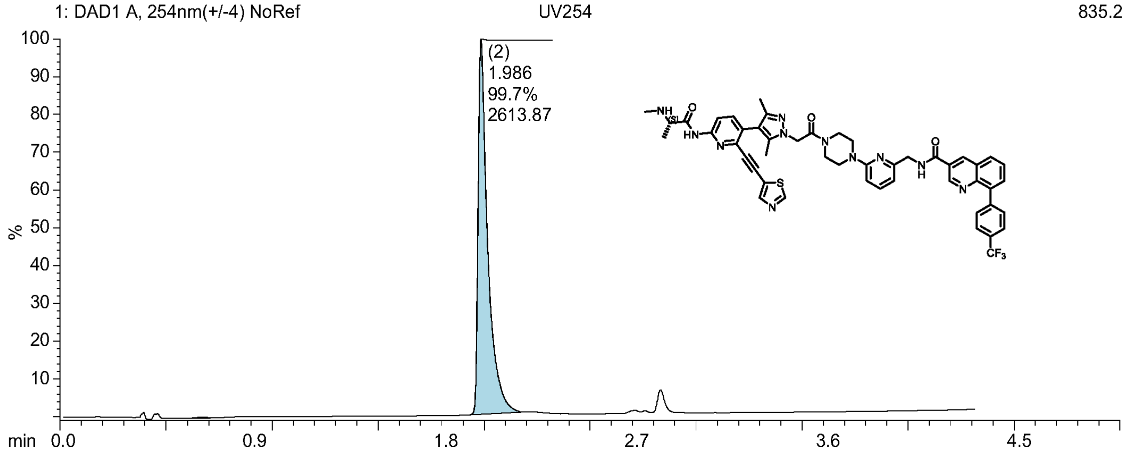

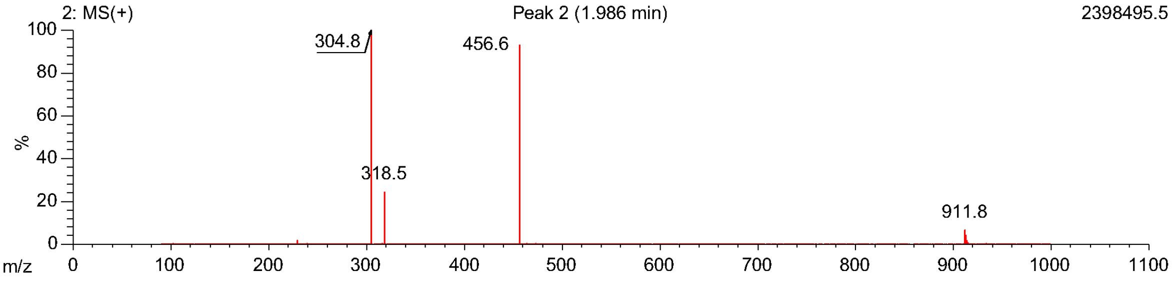

A531

e. HRMS

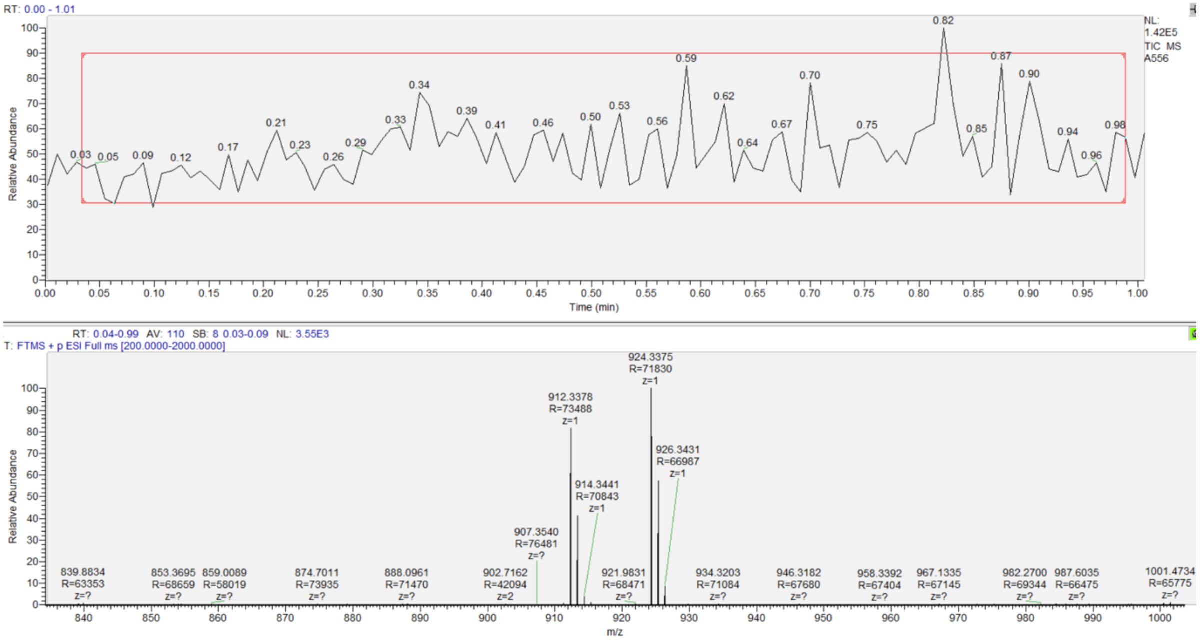
RT: 0.00 - 1.01
NL: 1.42E5 TIC MS A556
Relative Abundance
Time (min)
RT: 0.04-0.99 AV: 110 SB: 8 0.03-0.09 NL: 3.55E3
T: FTMS + p ESI Full ms [200.0000-2000.0000]
924.3375 R=71830 z=1
912.3378 R=73488 z=1
926.3431 R=66987 z=1
914.3441 R=70843 z=1
907.3540 R=76481 z=?
m/z

A557

a. ^{1}H-NMR

300 MHz, MeOD

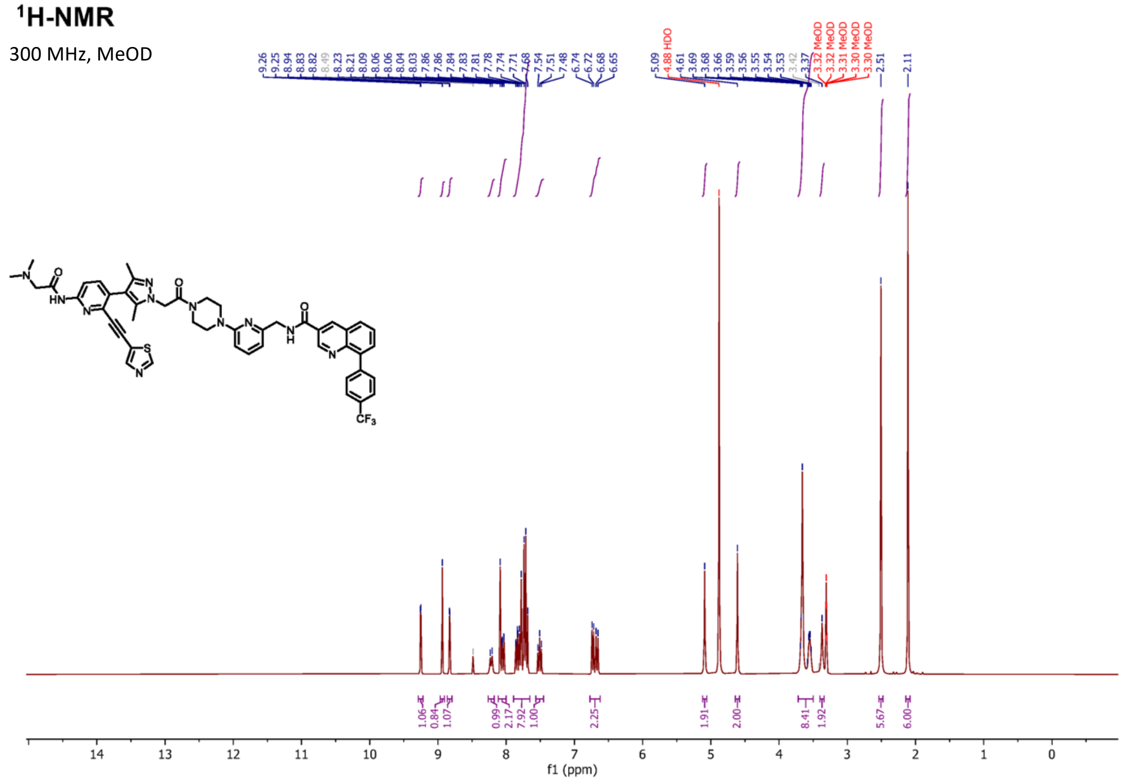

b. ^{13}C-NMR

75 MHz, MeOD

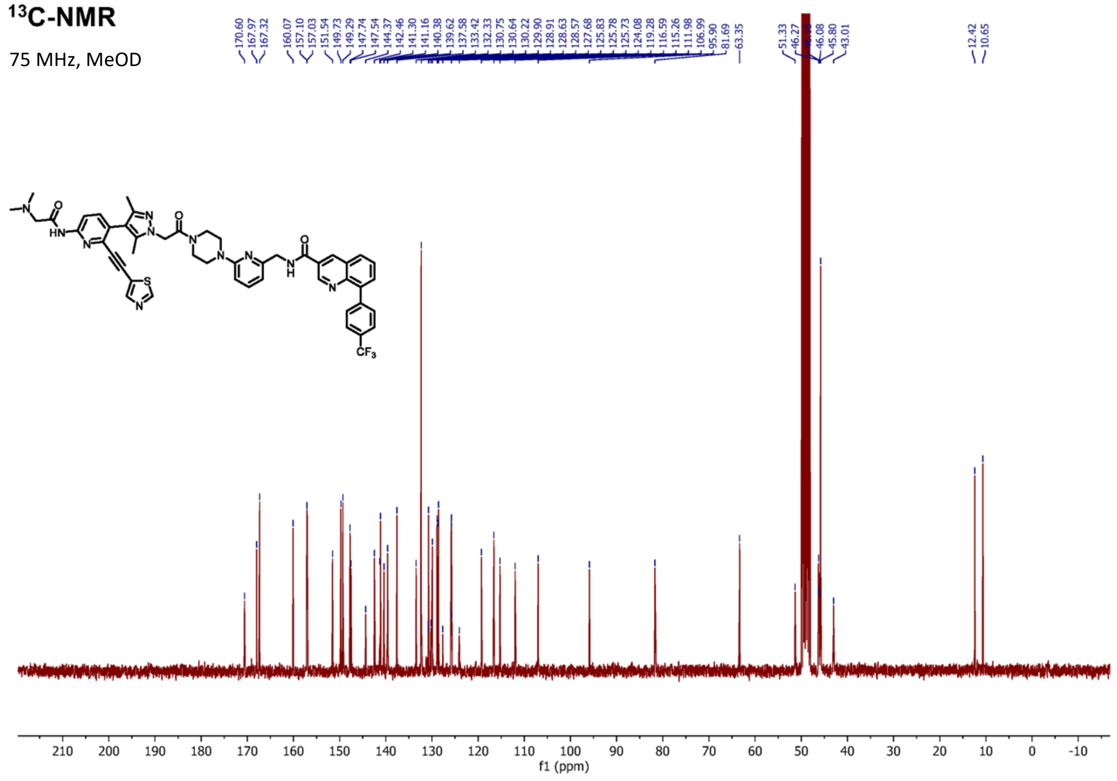

A557

c. ^{19}F-NMR

282 MHz, MeOD

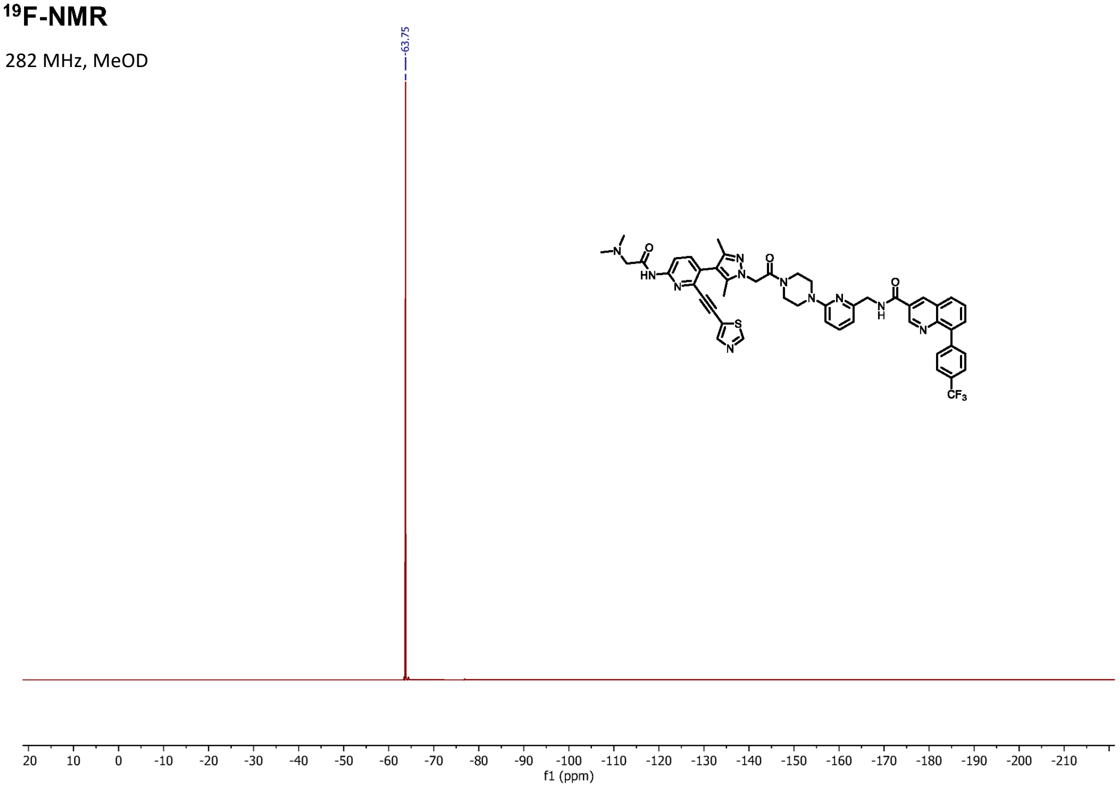

d. LCMS

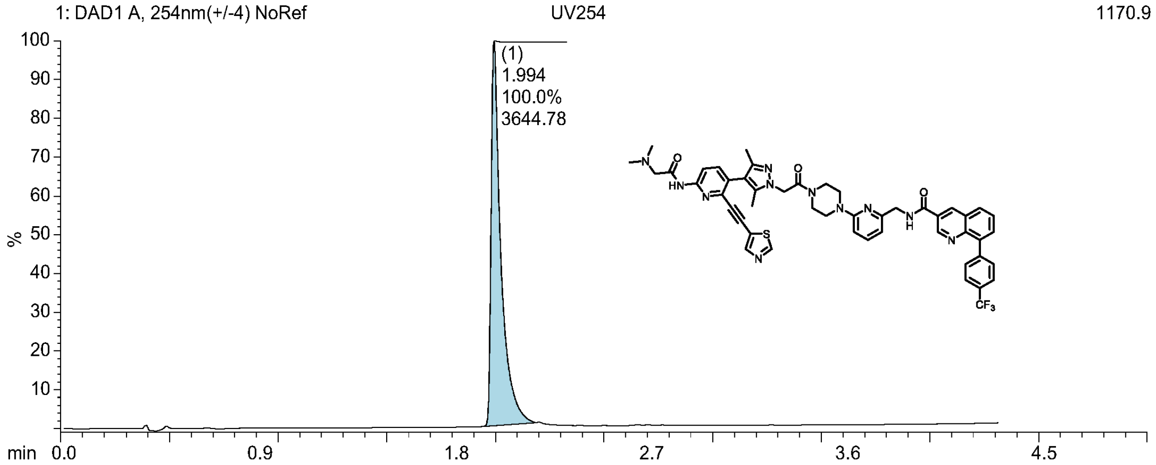

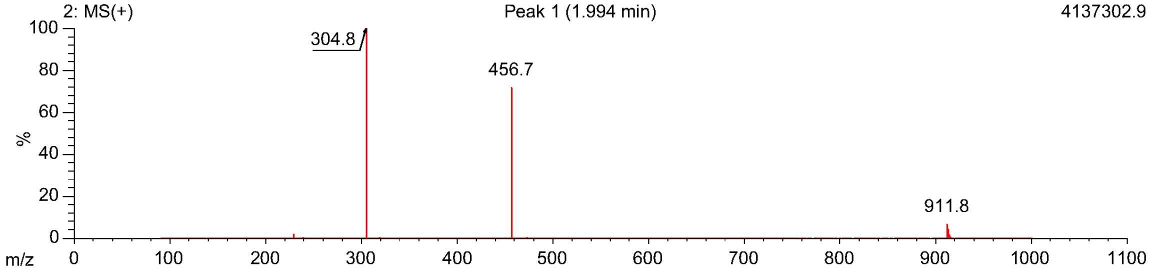

A557

e. HRMS

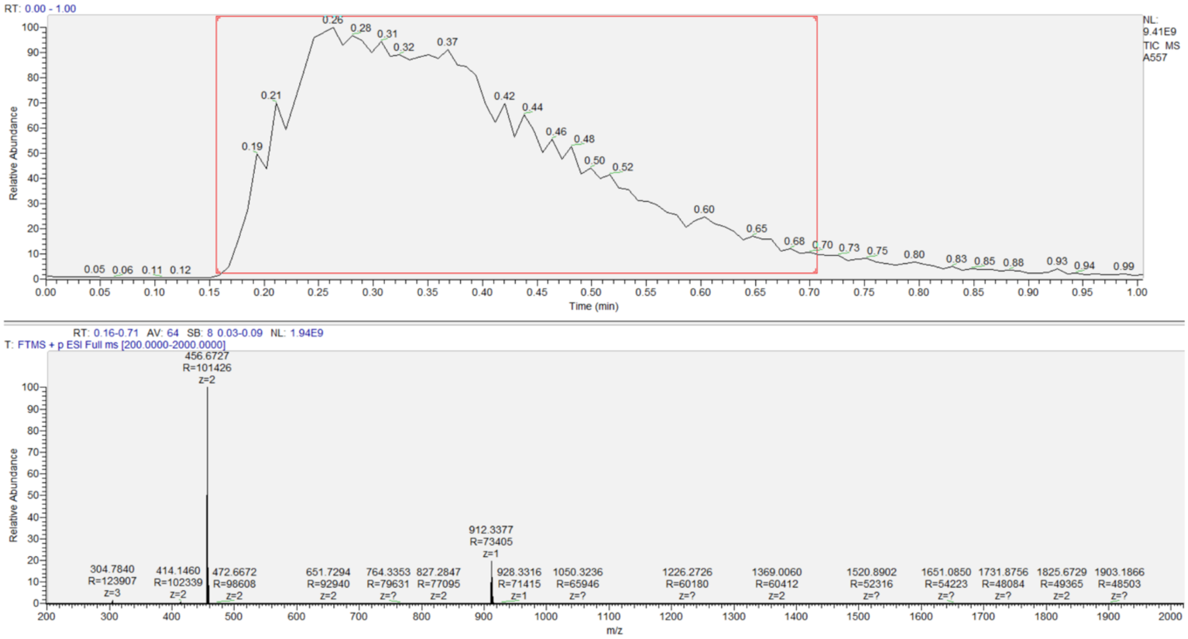

RT: 0.00 - 1.00
NL: 9.41E9 TIC MS A557
Relative Abundance
Time (min)
RT: 0.16-0.71 AV: 64 SB: 8 0.03-0.09 NL: 1.94E9
T: FTMS + p ESI Full ms [200.0000-2000.0000]
456.6727 R=101426 z=2
912.3377 R=73405 z=1
304.7840 R=123907 z=3
414.1460 R=102339 z=2
472.6672 R=98608 z=2
651.7294 R=92940 z=2
764.3353 R=79631 z=?
827.2847 R=77095 z=2
928.3316 R=71415 z=1
1050.3236 R=65946 z=?
1226.2726 R=60180 z=?
1369.0060 R=60412 z=2
1520.8902 R=52316 z=?
1651.0850 R=54223 z=?
1731.8756 R=48084 z=?
1825.6729 R=49365 z=2
1903.1866 R=48503 z=?
m/z

A423

a. ^{1}H-NMR

500 MHz, MeOD

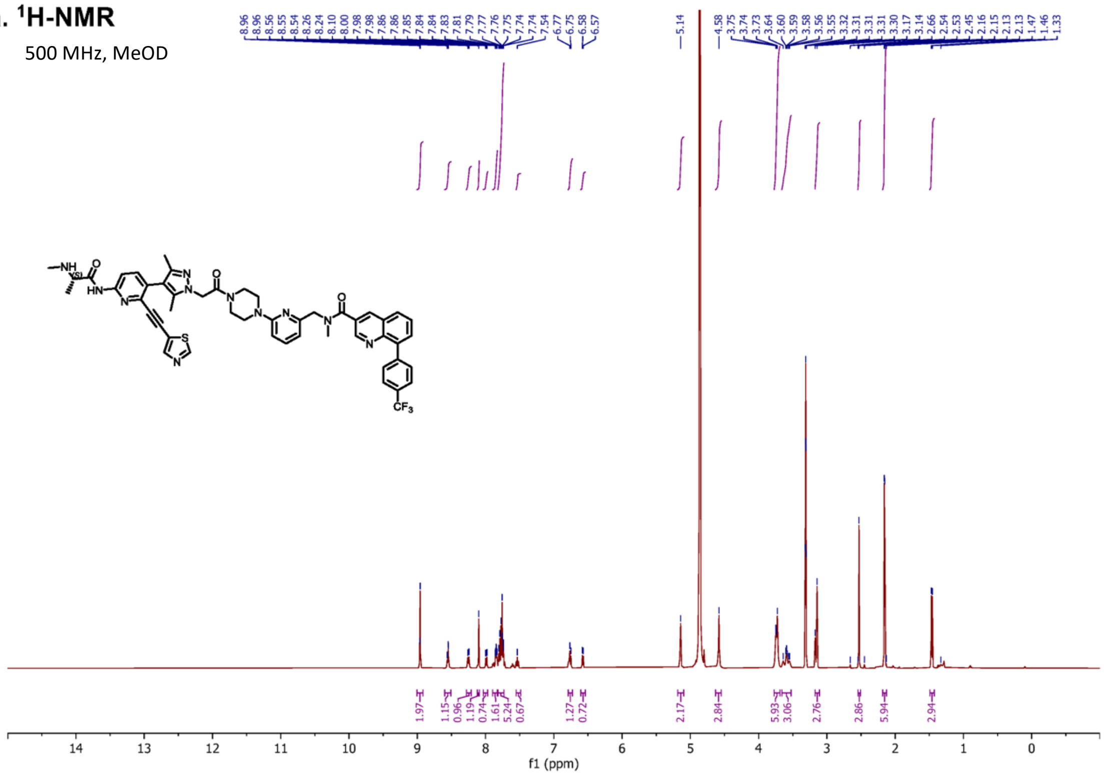

b. ^{13}C-NMR

126 MHz, MeOD

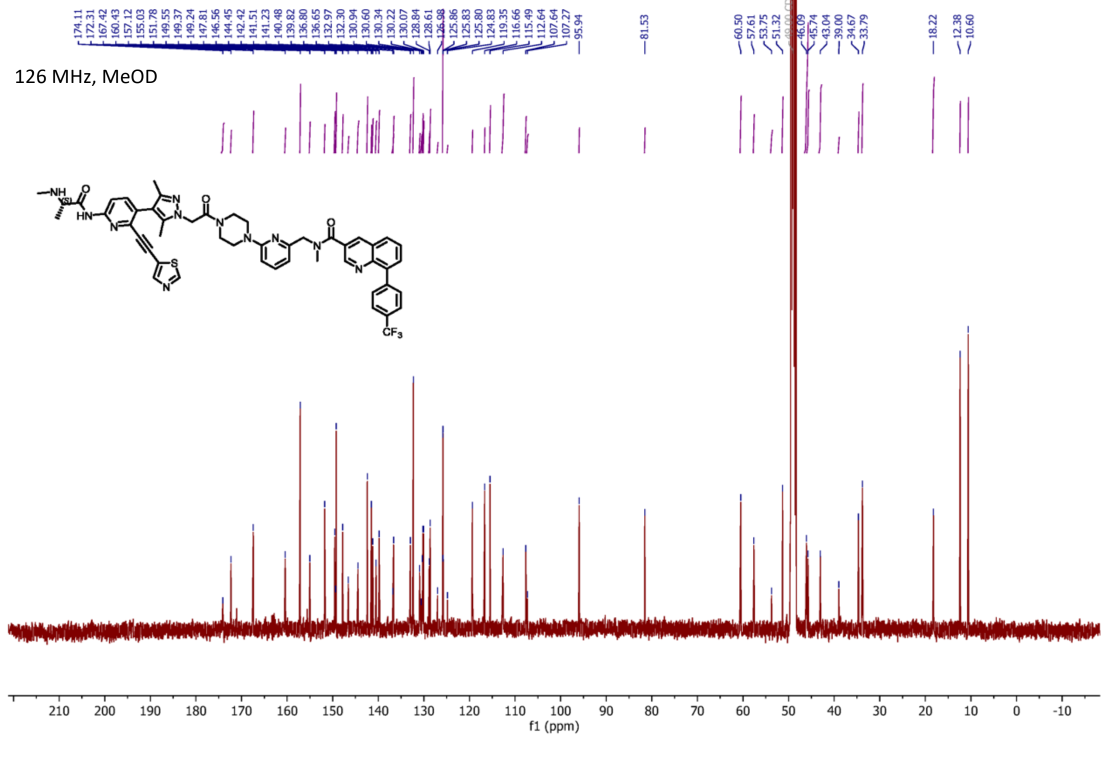

A423

c. ^{19}F-NMR

471 MHz, MeOD

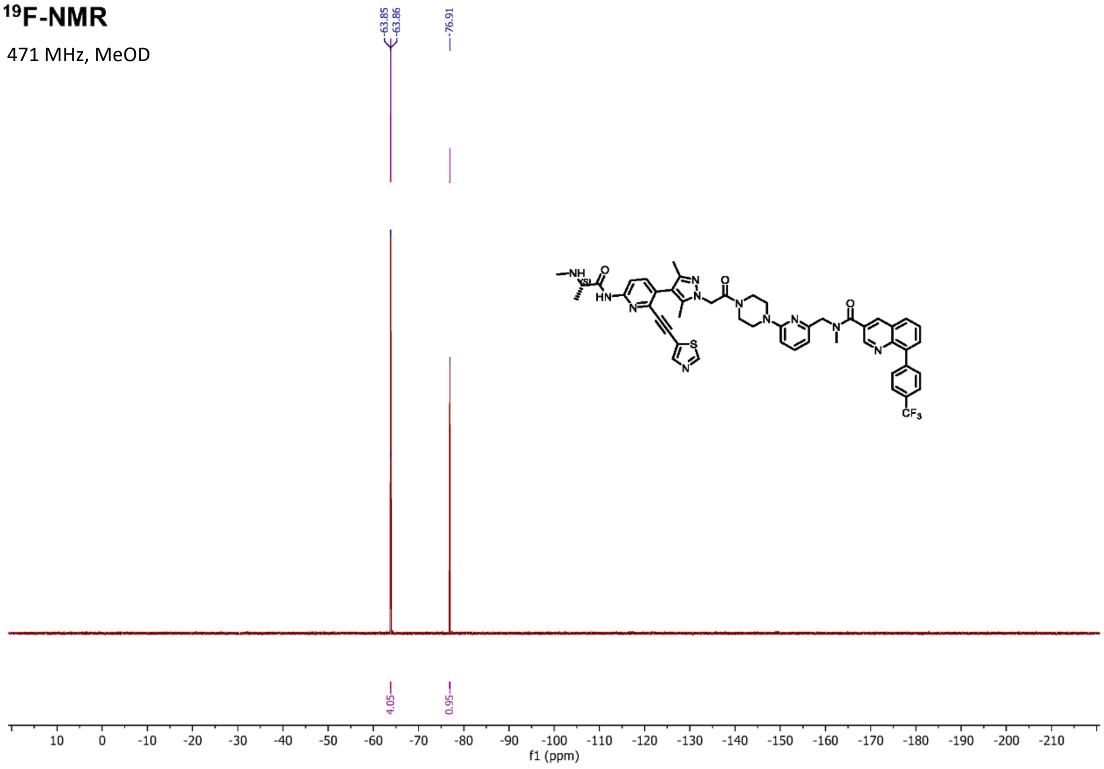

d. LCMS

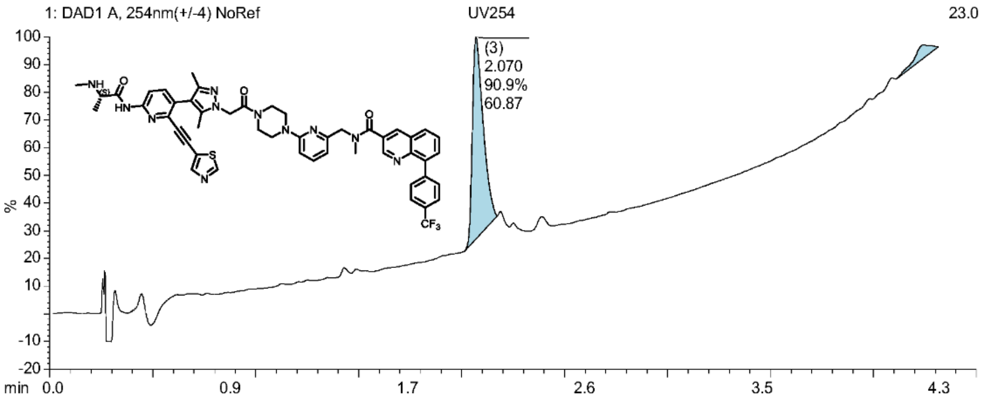

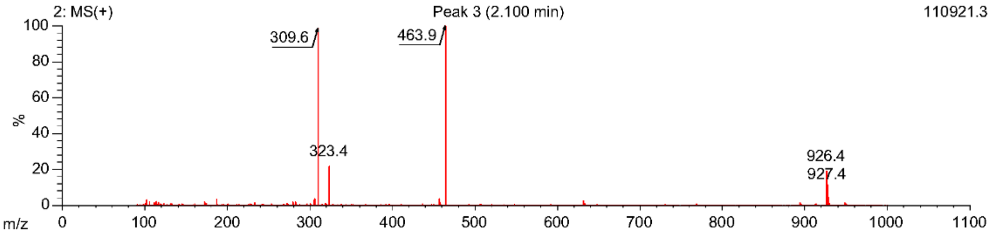

A423

e. HRMS

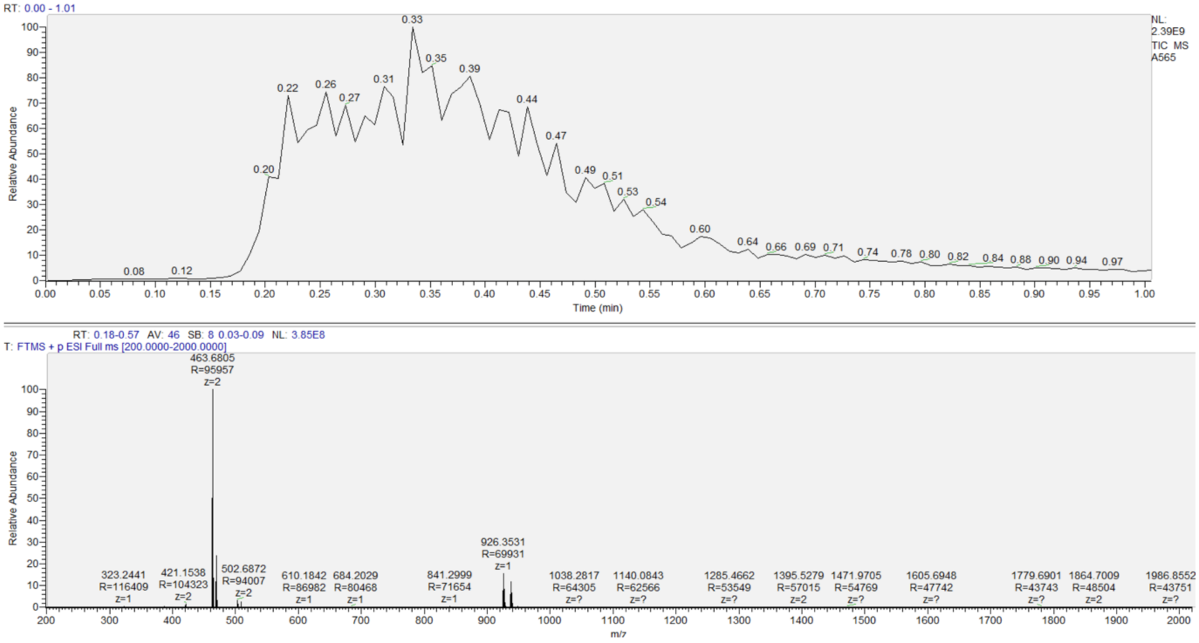

RT: 0.00 - 1.01
NL: 2.39E9
TIC MS A565
Relative Abundance
Time (min)
RT: 0.18-0.57 AV: 46 SB: 8 0.03-0.09 NL: 3.85E8
T: FTMS + p ESI Full ms [200.0000-2000.0000]
463.6805
R=95957
z=2
926.3531
R=69931
z=1
323.2441
R=116409
z=1
421.1538
R=104323
z=2
502.6872
R=94007
z=2
610.1842
R=86982
z=1
684.2029
R=80468
z=1
841.2999
R=71654
z=1
1038.2817
R=64305
z=?
1140.0843
R=62566
z=?
1285.4662
R=53549
z=?
1395.5279
R=57015
z=2
1471.9705
R=54769
z=?
1605.6948
R=47742
z=?
1779.6901
R=43743
z=?
1864.7009
R=48504
z=2
1986.8552
R=43751
z=?
m/z

A538

a. ^{1}H-NMR

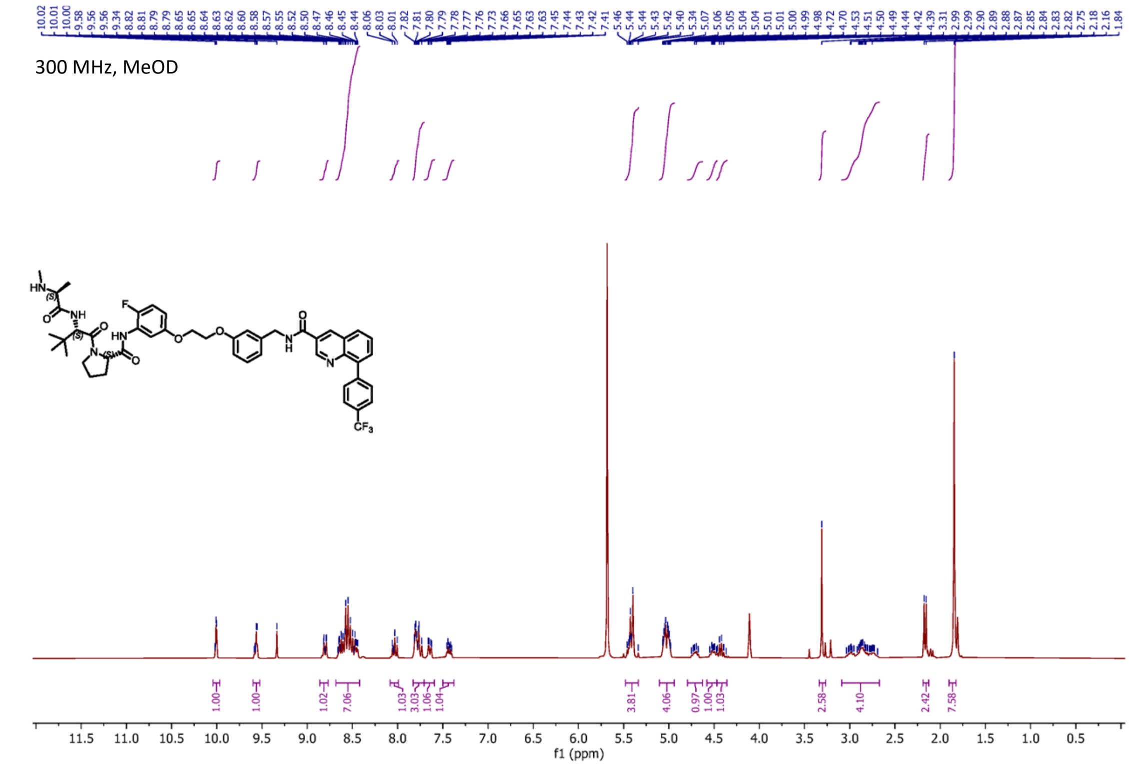

b. ^{13}C-NMR

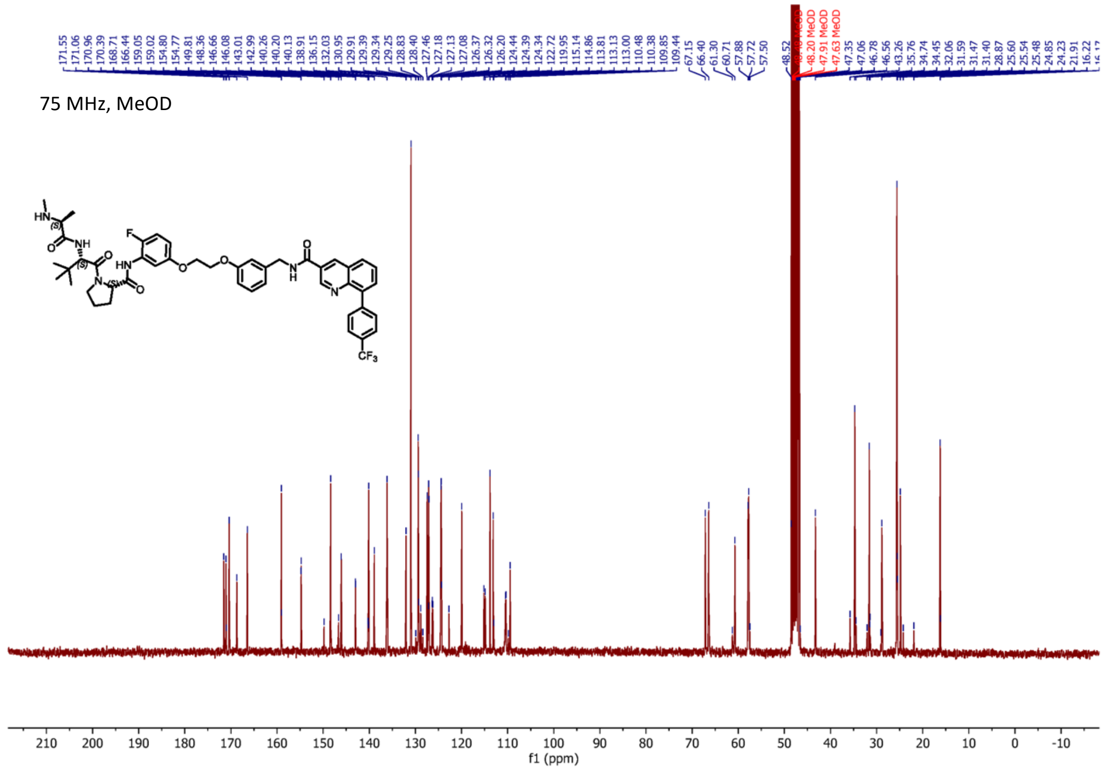

A538

c. ^{19}F-NMR

282 MHz, MeOD

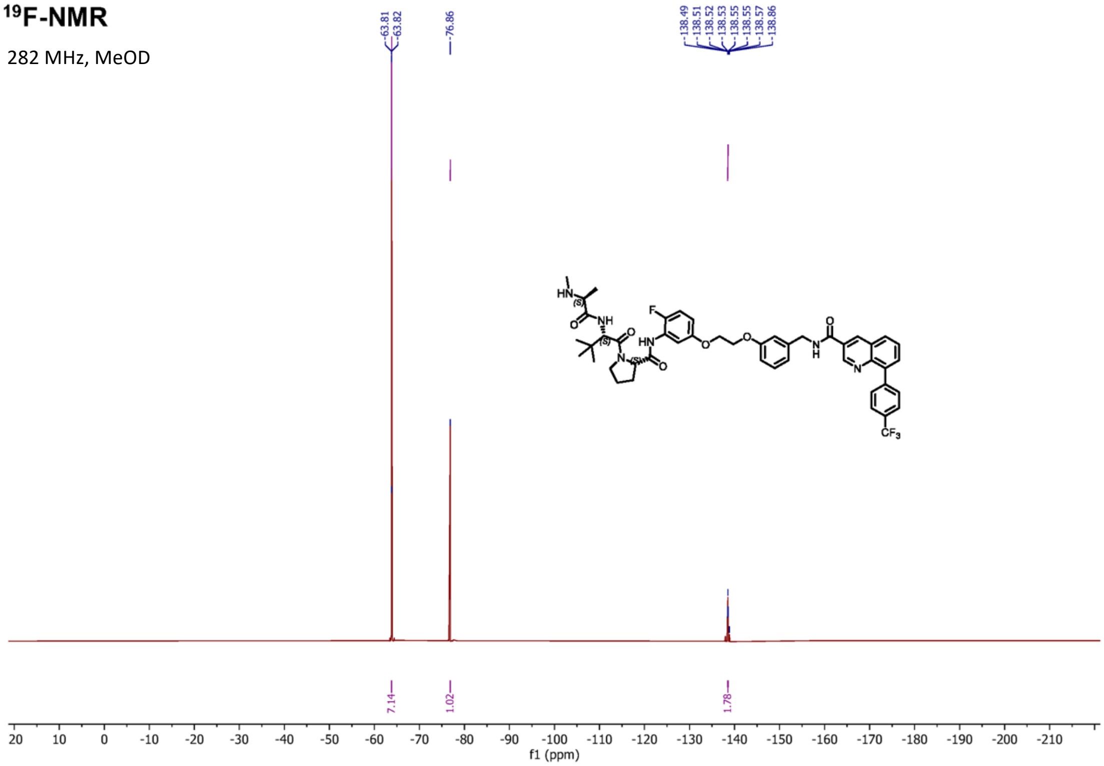

d. LCMS

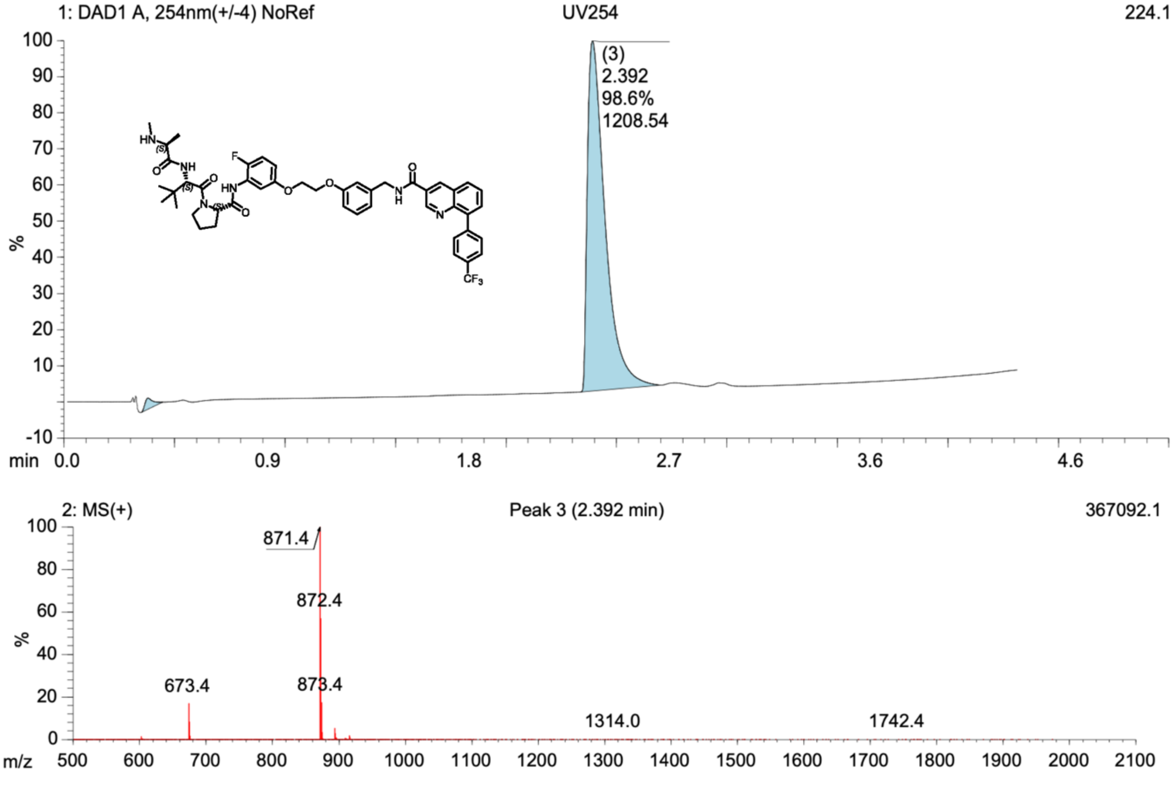

A538

e. HRMS

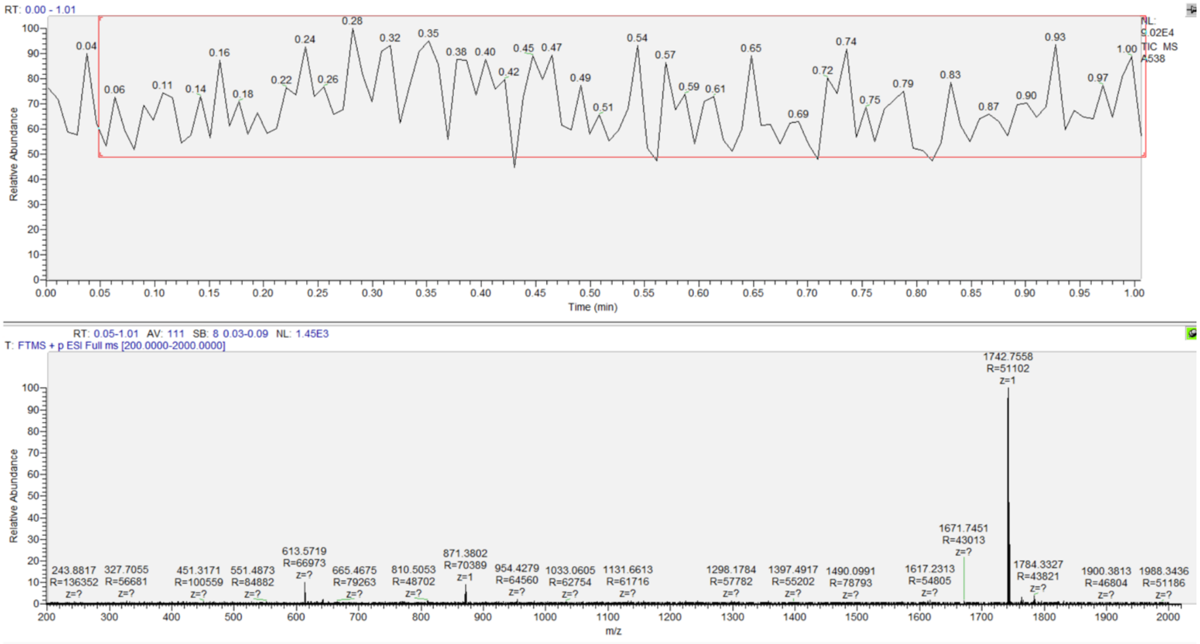

RT: 0.00 - 1.01
NL: 5.02E4 TIC MS A538
Relative Abundance
Time (min)
RT: 0.05-1.01 AV: 111 SB: 8 0.03-0.09 NL: 1.45E3
T: FTMS + p ESI Full ms [200.0000-2000.0000]
1742.7558 R=51102 z=1
1671.7451 R=43013 z=?
1784.3327 R=43821 z=?
871.3802 R=70389 z=1
613.5719 R=66973 z=?
m/z

A559

a. ^{1}H-NMR

300 MHz, MeOD

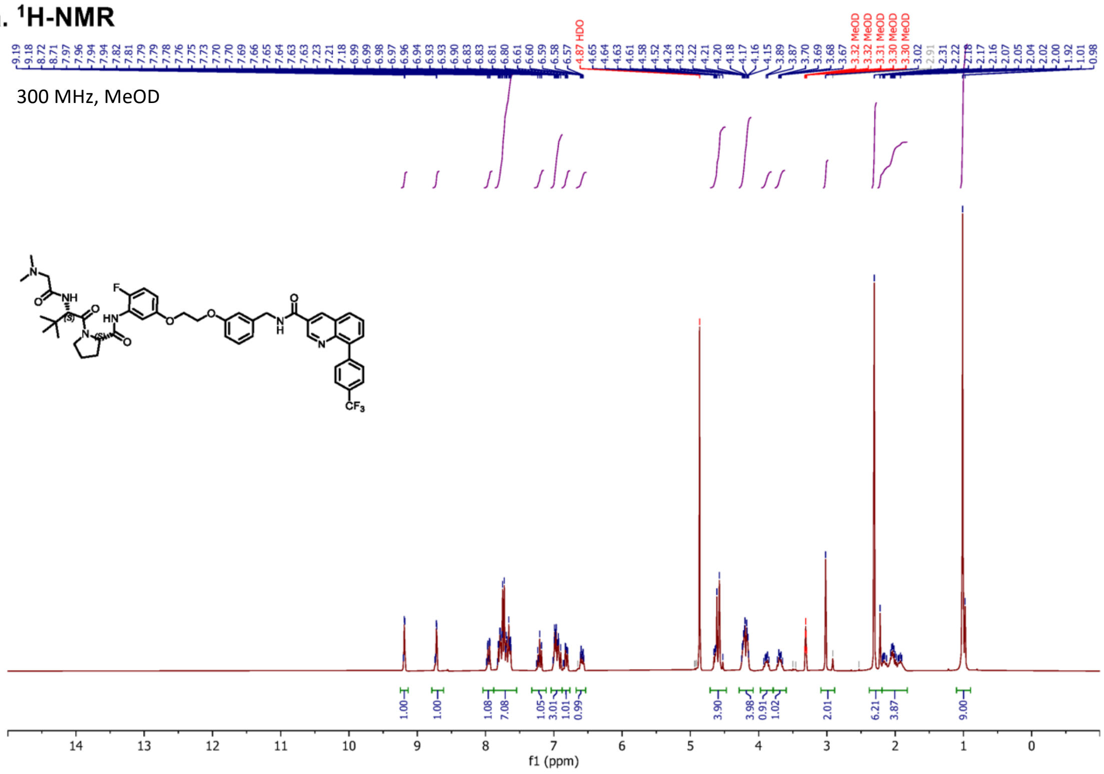

b. ^{13}C-NMR

75 MHz, MeOD

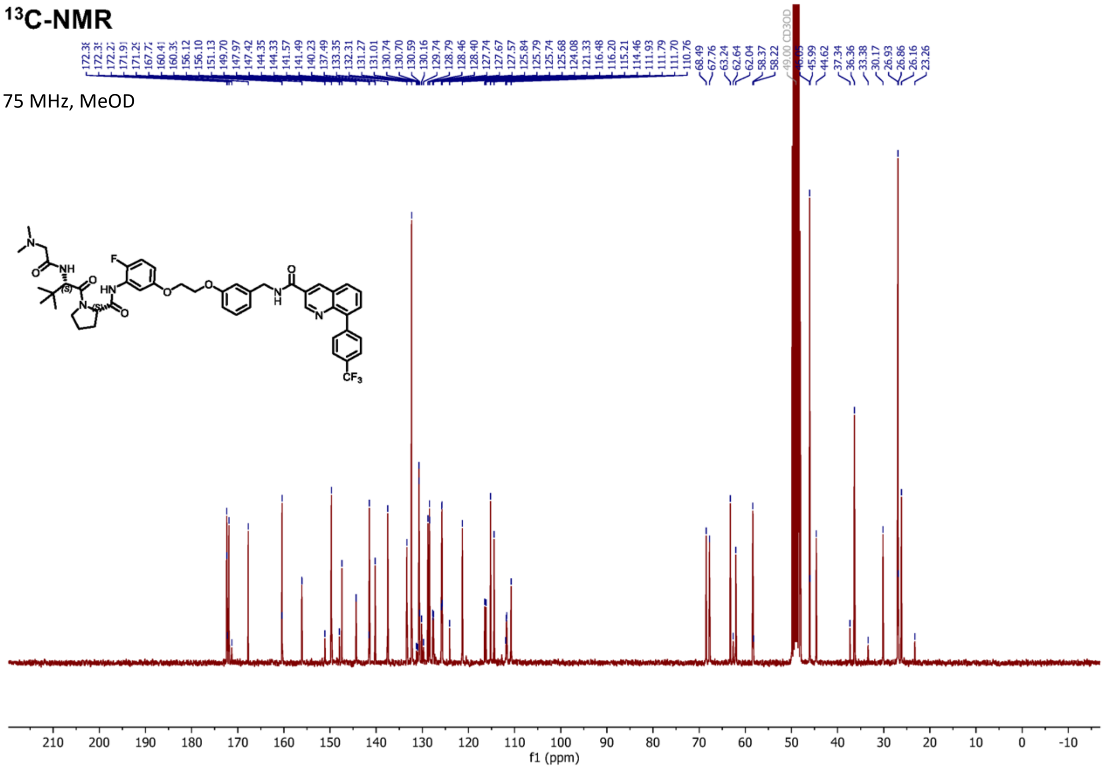

A559

c. ^{19}F-NMR

282 MHz, MeOD

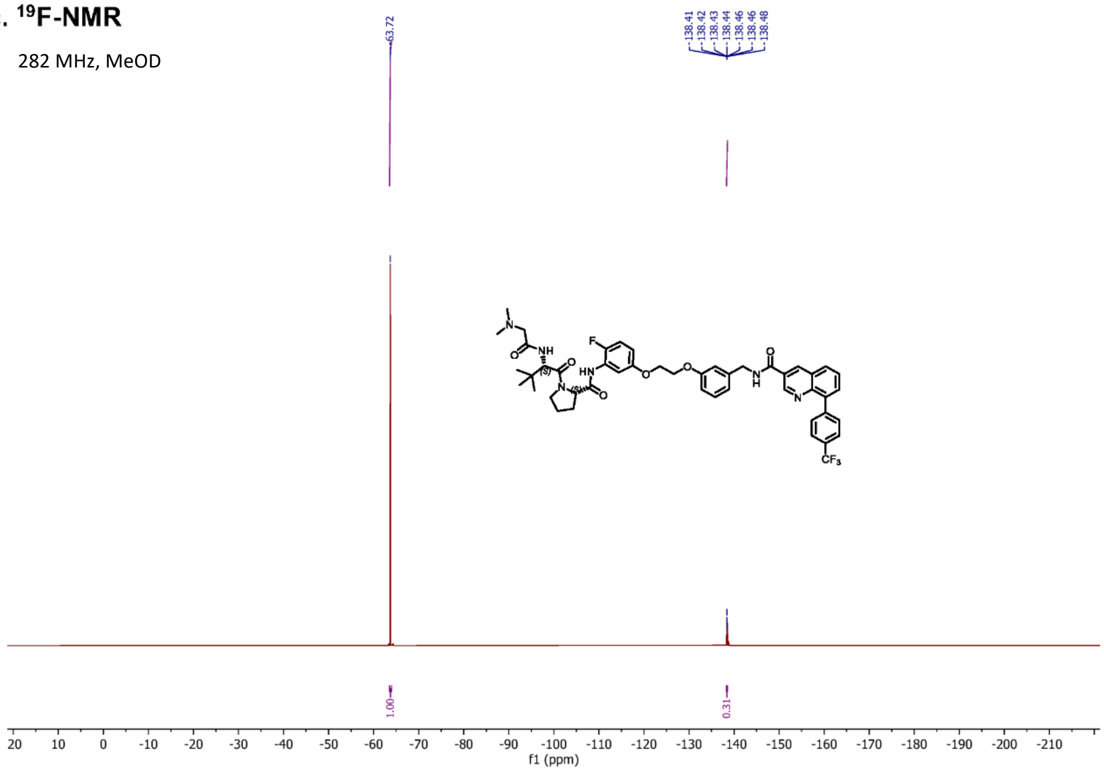

d. LCMS

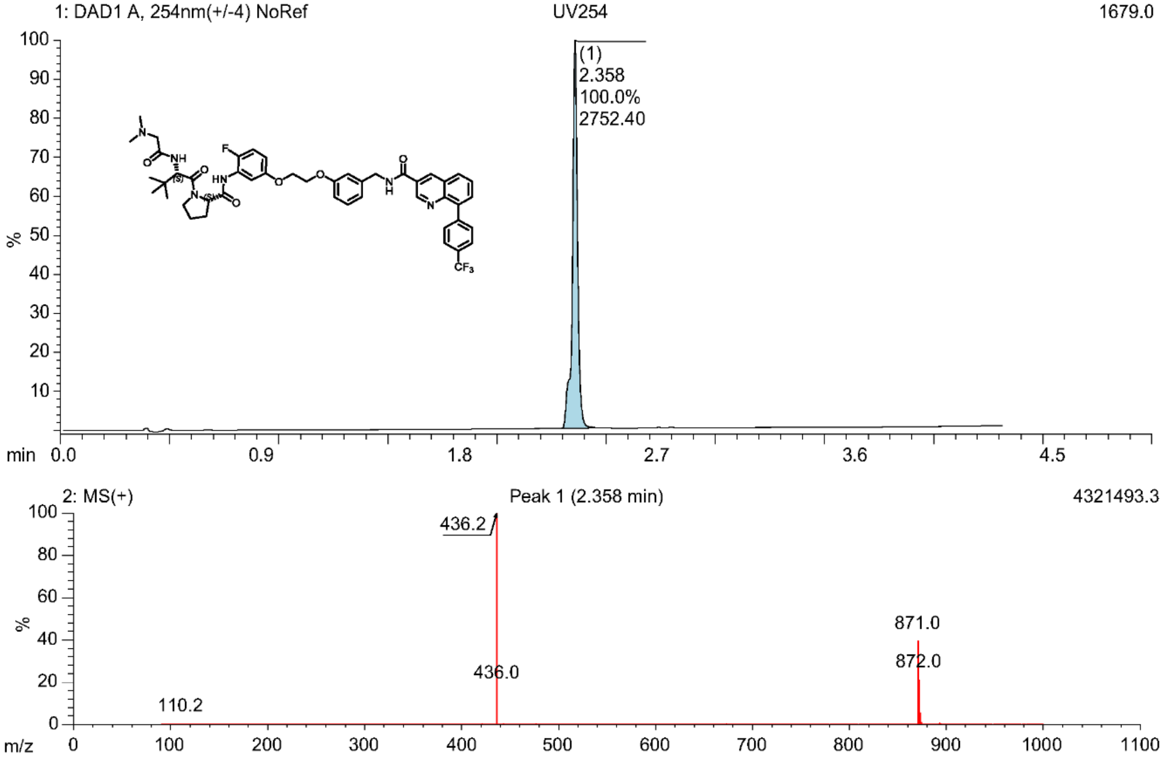

A559

e. HRMS

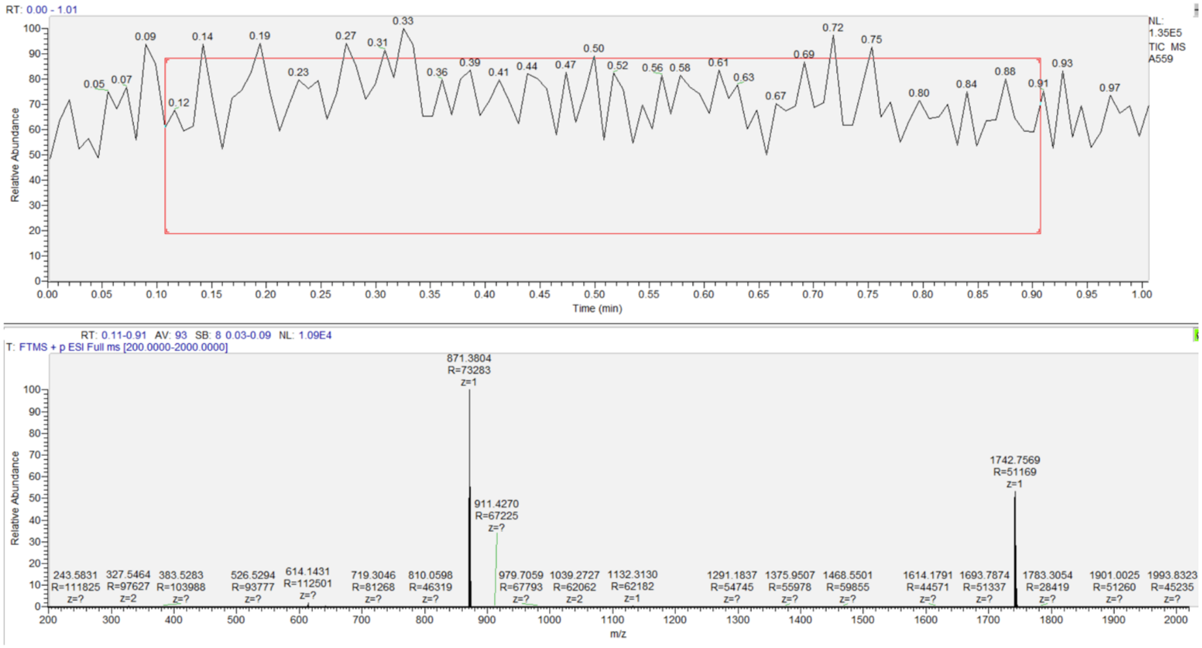

RT: 0.00 - 1.01
NL: 1.35E5 TIC MS A559
Relative Abundance
Time (min)
RT: 0.11-0.91 AV: 93 SB: 8 0.03-0.09 NL: 1.09E4
T: FTMS + p ESI Full ms [200.0000-2000.0000]
871.3804
R=73283
z=1
1742.7569
R=51169
z=1
911.4270
R=67225
z=?
m/z

A561

a. ^{1}H-NMR

300 MHz, MeOD

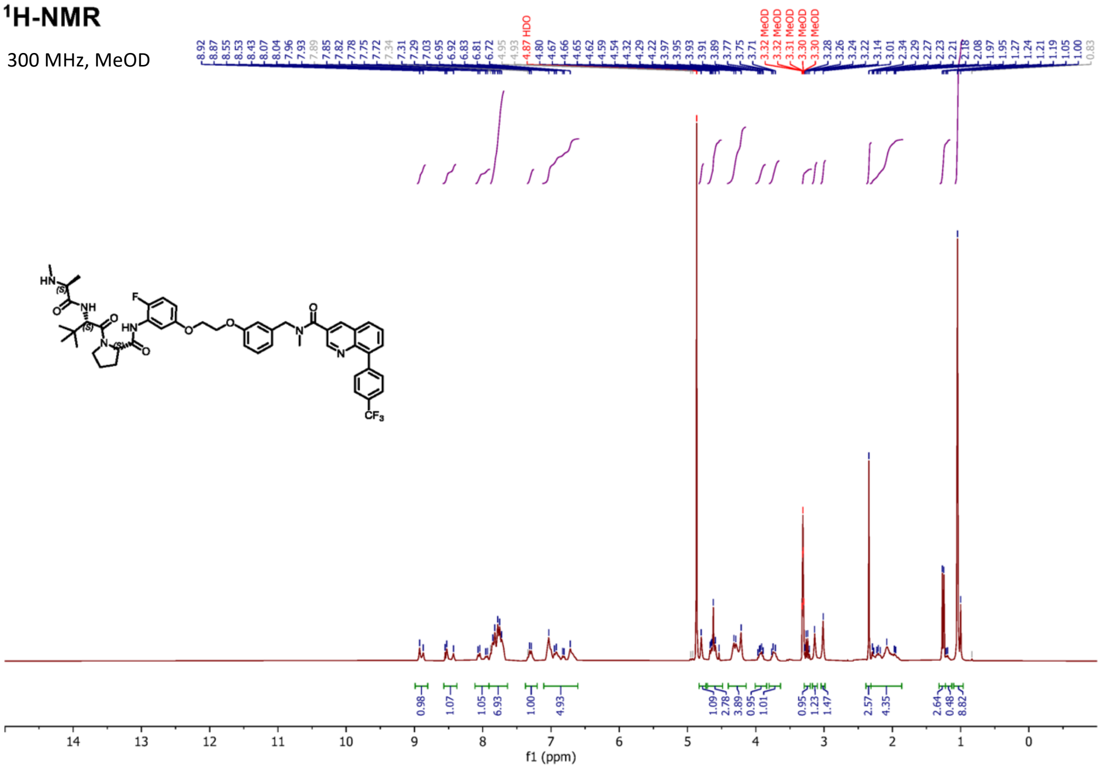

b. ^{13}C-NMR

126 MHz, MeOD

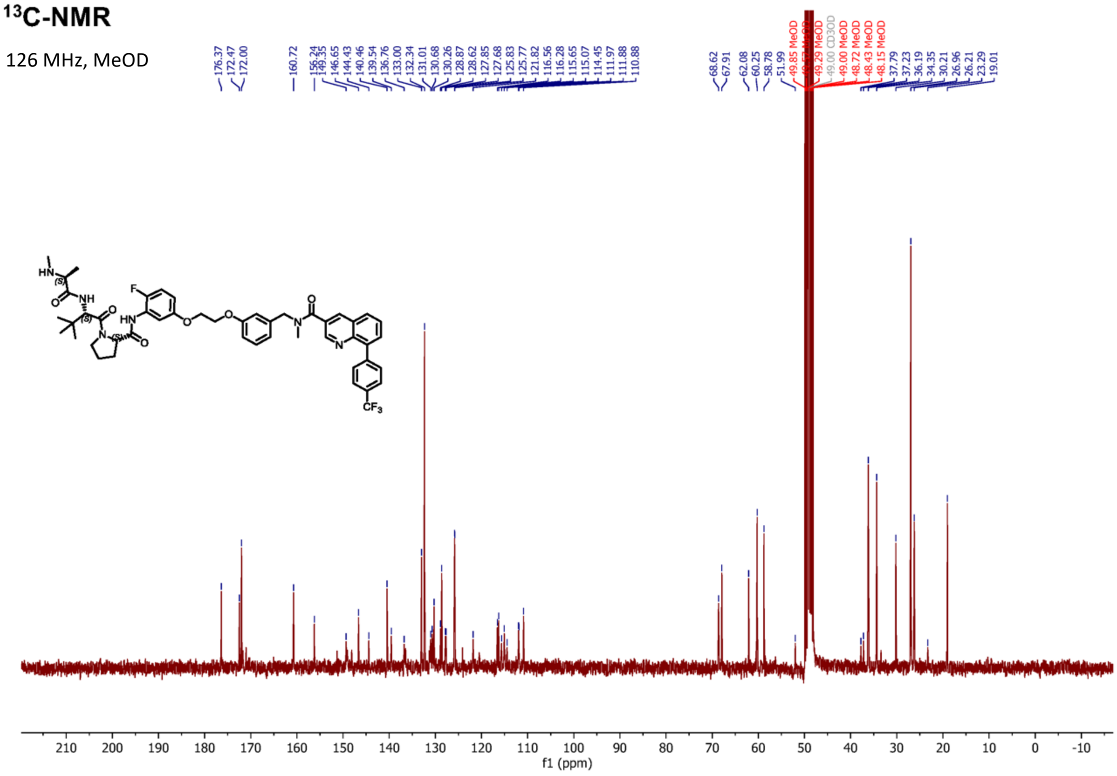

A561

c. ^{19}F-NMR

282 MHz, MeOD

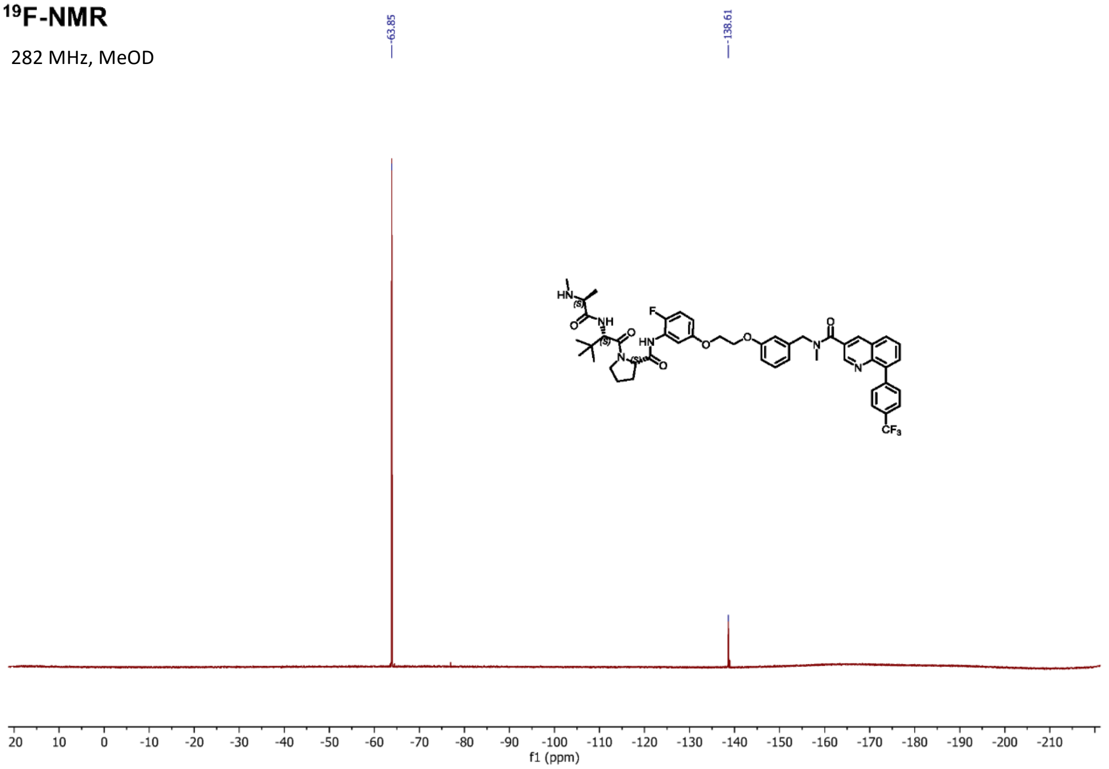

d. LCMS

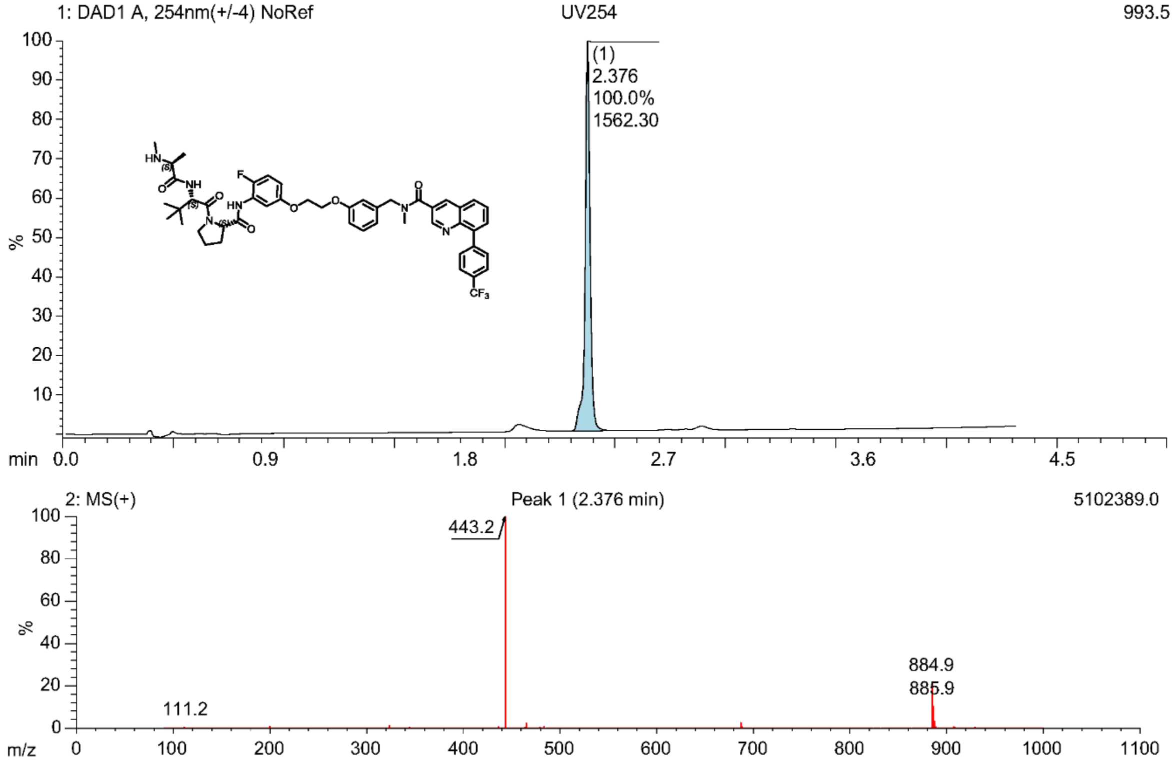

A561

e. HRMS

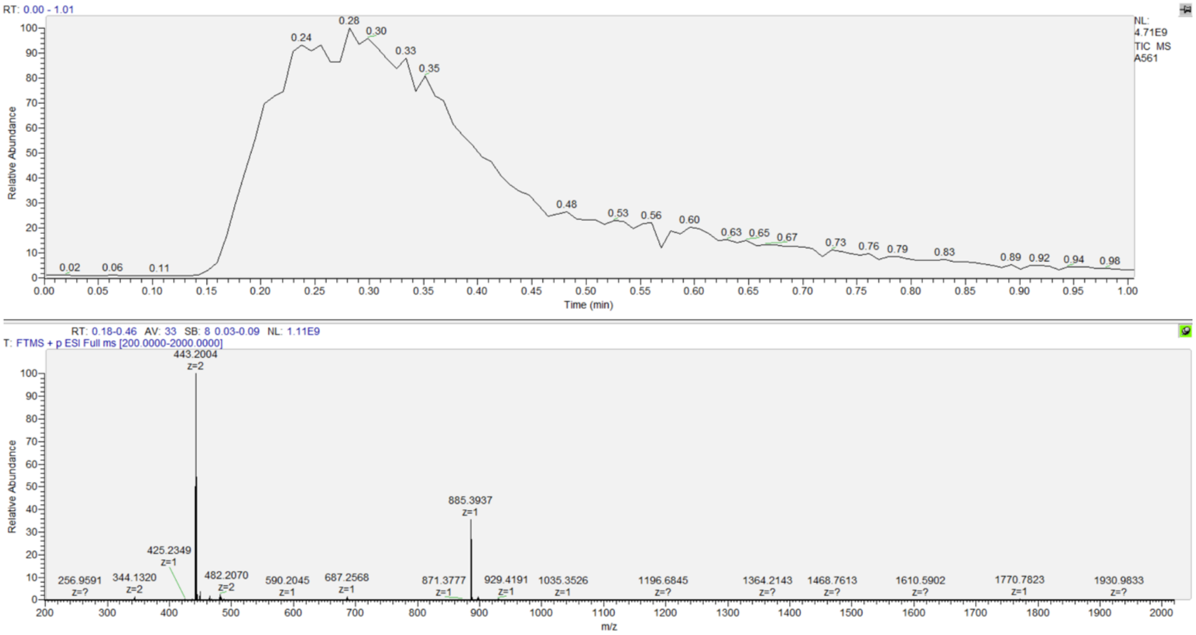

RT: 0.00 - 1.01
NL: 4.71E9 TIC MS A561
Relative Abundance
Time (min)
RT: 0.18-0.46 AV: 33 SB: 8 0.03-0.09 NL: 1.11E9
T: FTMS + p ESI Full ms [200.0000-2000.0000]
443.2004 z=2
885.3937 z=1
425.2349 z=1
482.2070 z=2
m/z

A536

a. ^{1}H-NMR

300 MHz, MeOD

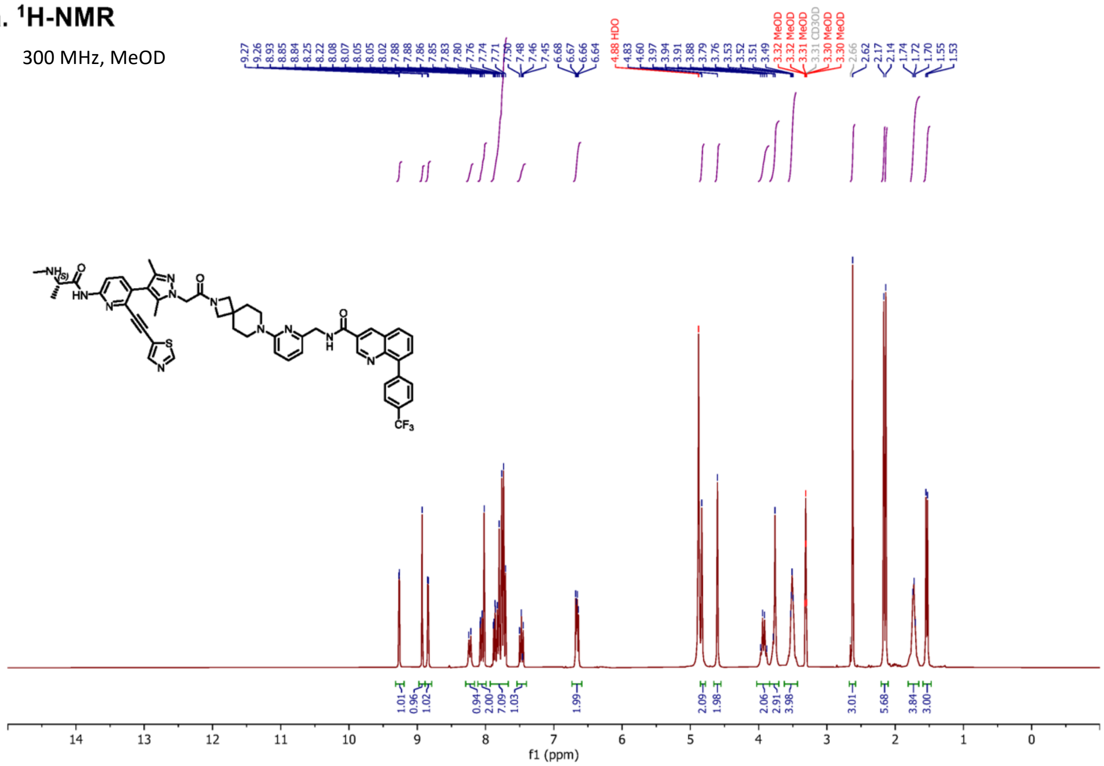

b. ^{13}C-NMR

75 MHz, MeOD

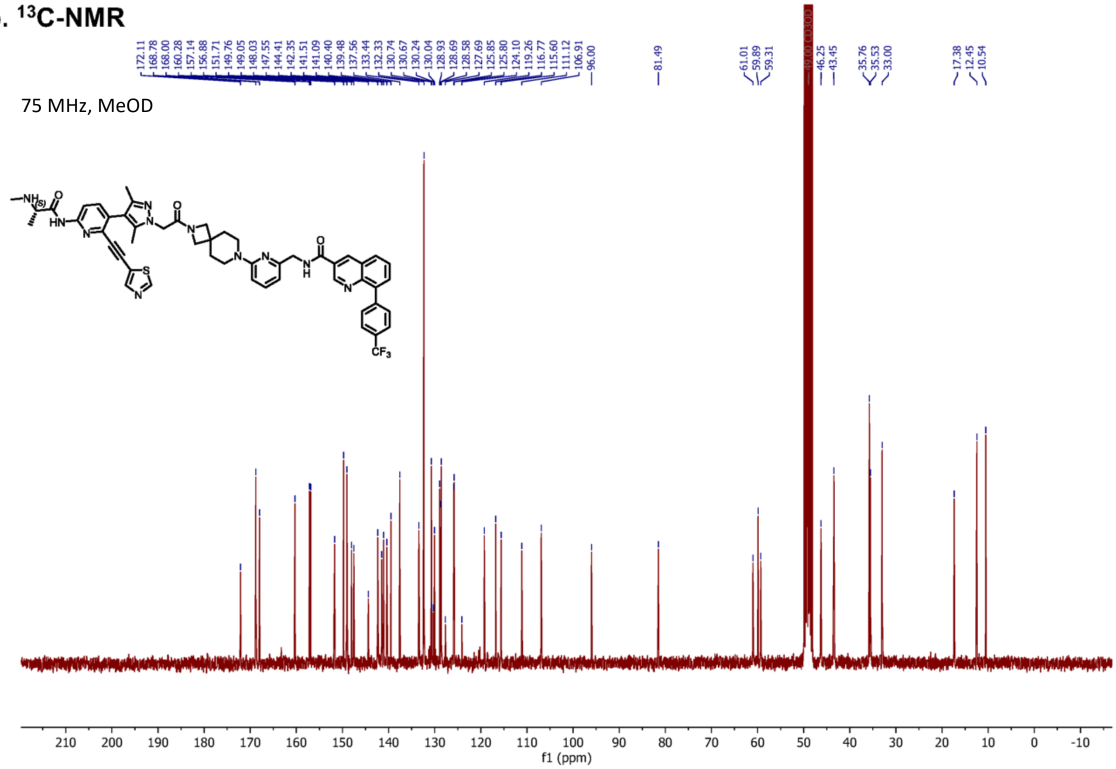

A536

c. ^{19}F-NMR

282 MHz, MeOD

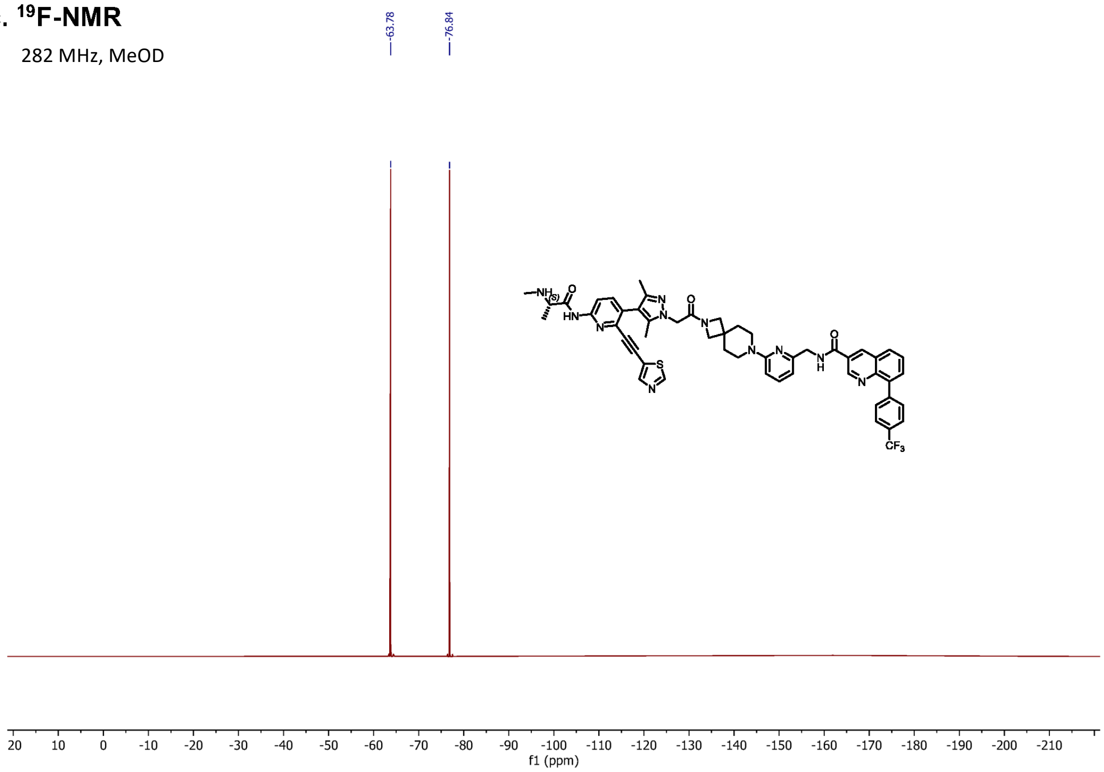

d. LCMS

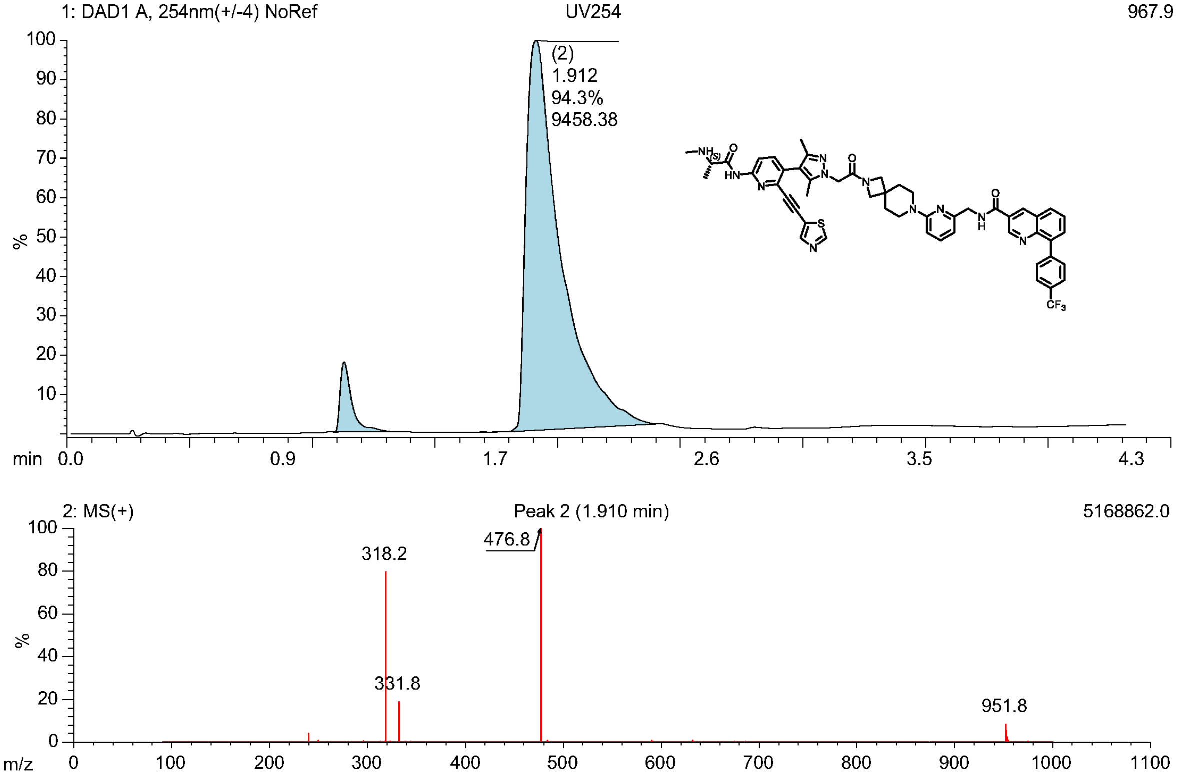

A536

e. HRMS

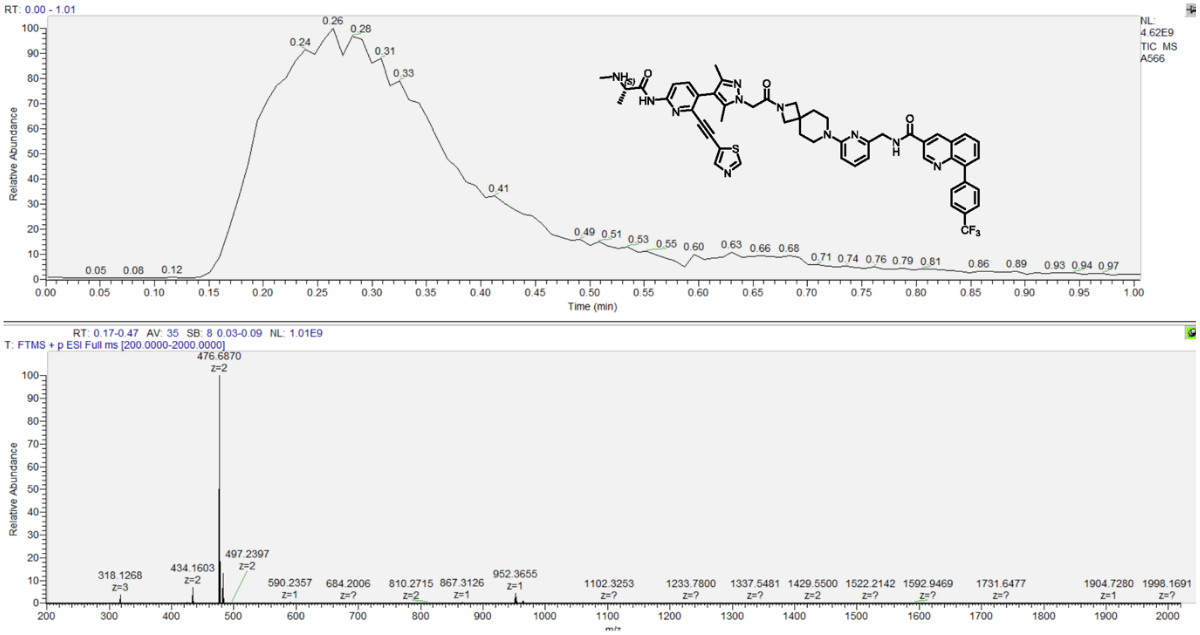

RT: 0.00 - 1.01
NL: 4.62E9 TIC MS A566
Relative Abundance
Time (min)
0.24 0.26 0.28 0.31 0.33 0.41 0.49 0.51 0.53 0.55 0.60 0.63 0.66 0.68 0.71 0.74 0.76 0.79 0.81 0.86 0.89 0.93 0.94 0.97 0.05 0.08 0.12
RT: 0.17-0.47 AV: 35 SB: 8 0.03-0.09 NL: 1.01E9
T: FTMS + p ESI Full ms [200.0000-2000.0000]
476.6870 z=2
497.2397 z=2
434.1603 z=2
318.1268 z=3
590.2357 z=1
684.2006 z=?
810.2715 z=2
867.3126 z=1
952.3655 z=1
1102.3253 z=?
1233.7800 z=?
1337.5481 z=?
1429.5500 z=2
1522.2142 z=?
1592.9469 z=?
1731.6477 z=?
1904.7280 z=1
1998.1691 z=?
m/z

A558

a. ¹H-NMR

300 MHz, MeOD

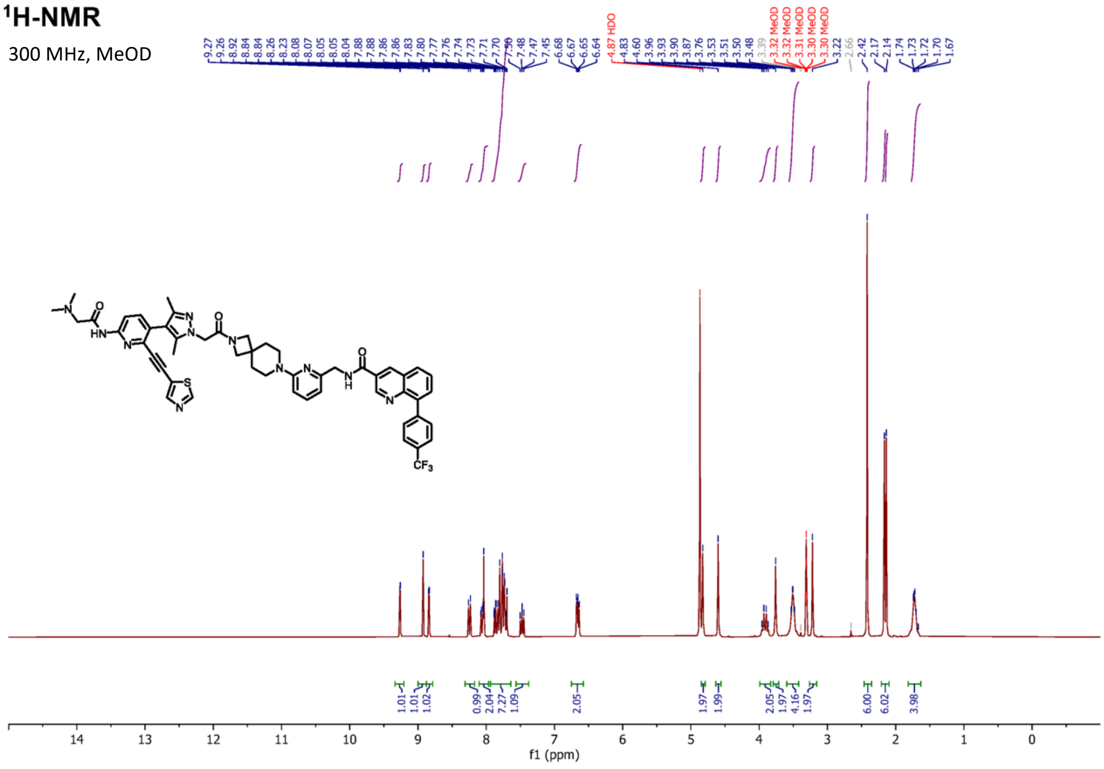

b. ¹³C-NMR

75 MHz, MeOD

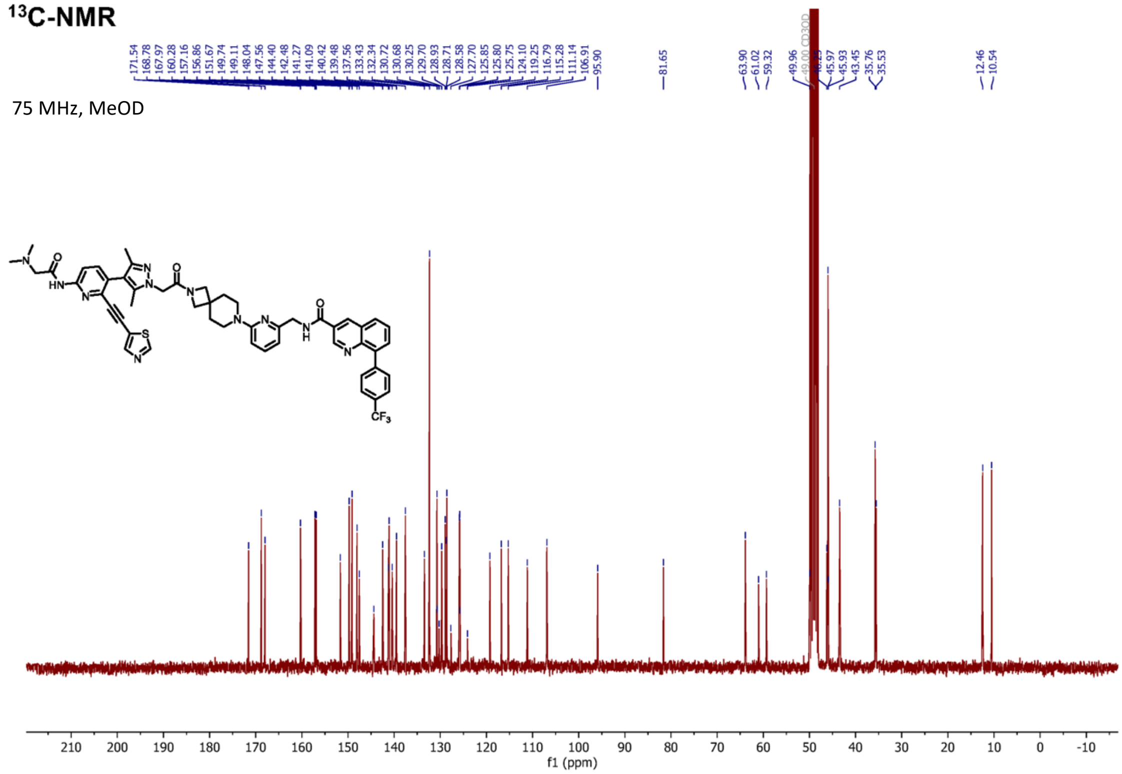

A558

c. ^{19}F-NMR

282 MHz, MeOD

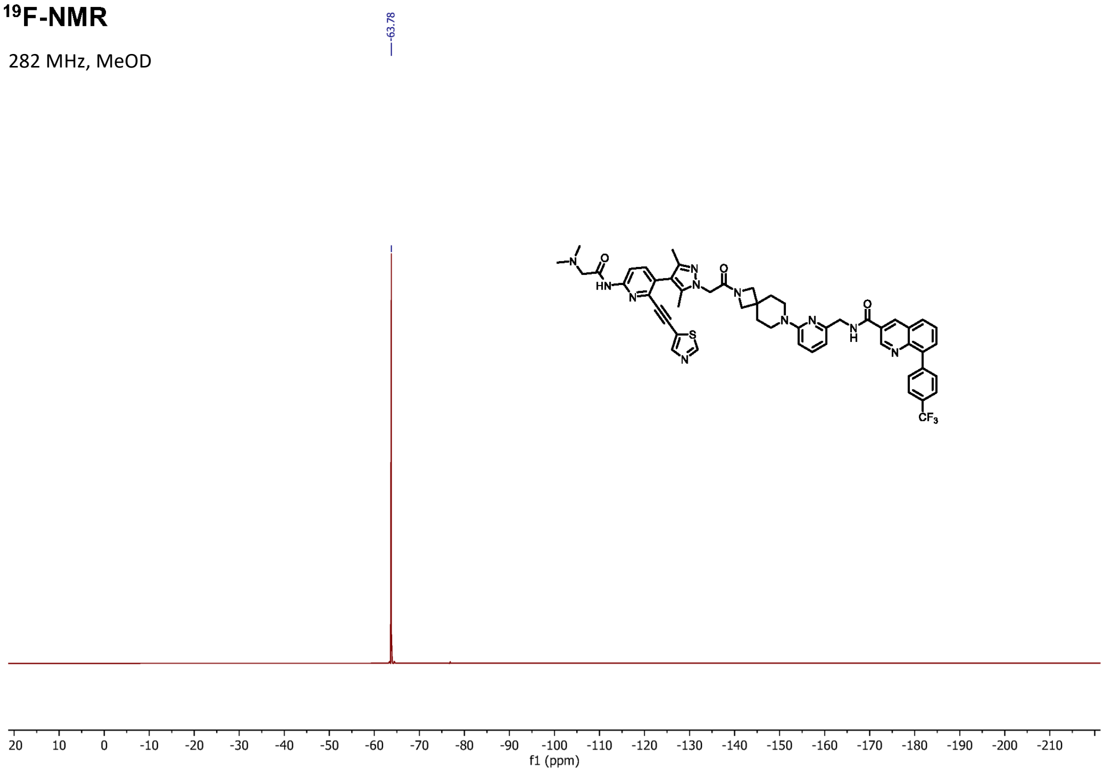

d. LCMS

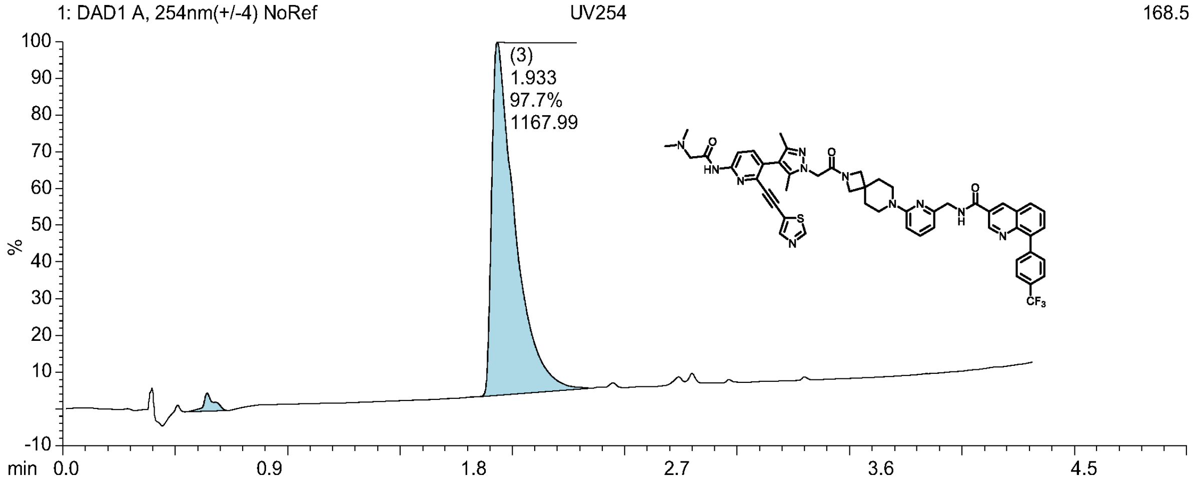

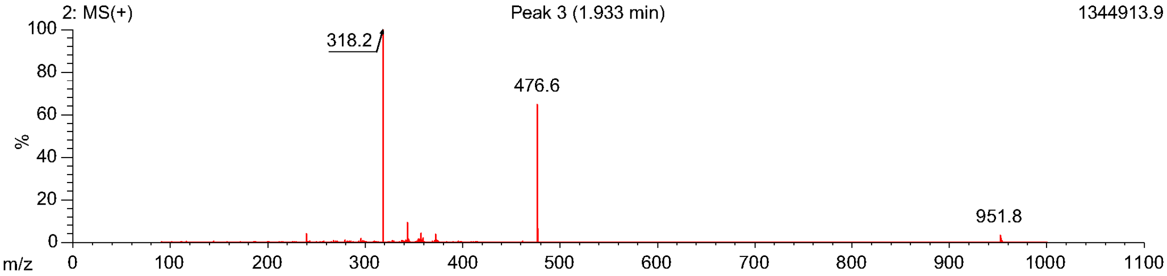

A558

e. HRMS

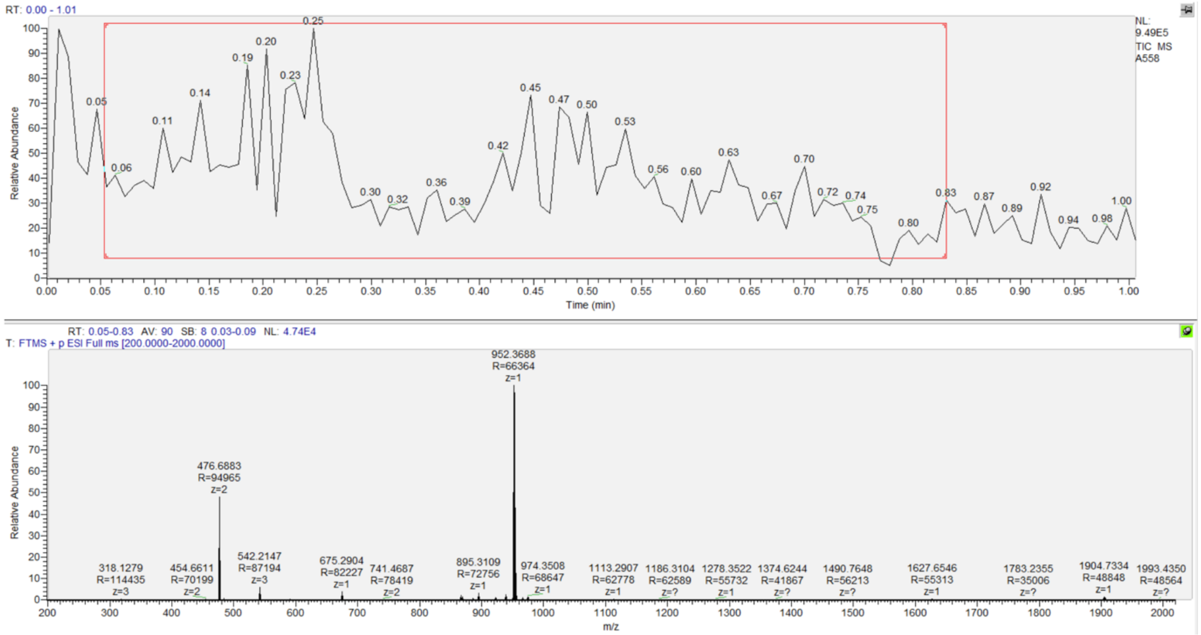
RT: 0.00 - 1.01
NL: 9.49E5 TIC MS A558
Relative Abundance
Time (min)
RT: 0.05-0.83 AV: 90 SB: 8 0.03-0.09 NL: 4.74E4
T: FTMS + p ESI Full ms [200.0000-2000.0000]
952.3688 R=66364 z=1
476.6883 R=94965 z=2
318.1279 R=114435 z=3
454.6611 R=70199 z=2
542.2147 R=87194 z=3
675.2904 R=82227 z=1
741.4687 R=78419 z=2
895.3109 R=72756 z=1
974.3508 R=68647 z=1
1113.2907 R=62778 z=1
1186.3104 R=62589 z=?
1278.3522 R=55732 z=1
1374.6244 R=41867 z=?
1490.7648 R=56213 z=?
1627.6546 R=55313 z=1
1783.2355 R=35006 z=?
1904.7334 R=48848 z=1
1993.4350 R=48564 z=?
m/z

A560

a. ^{1}H-NMR

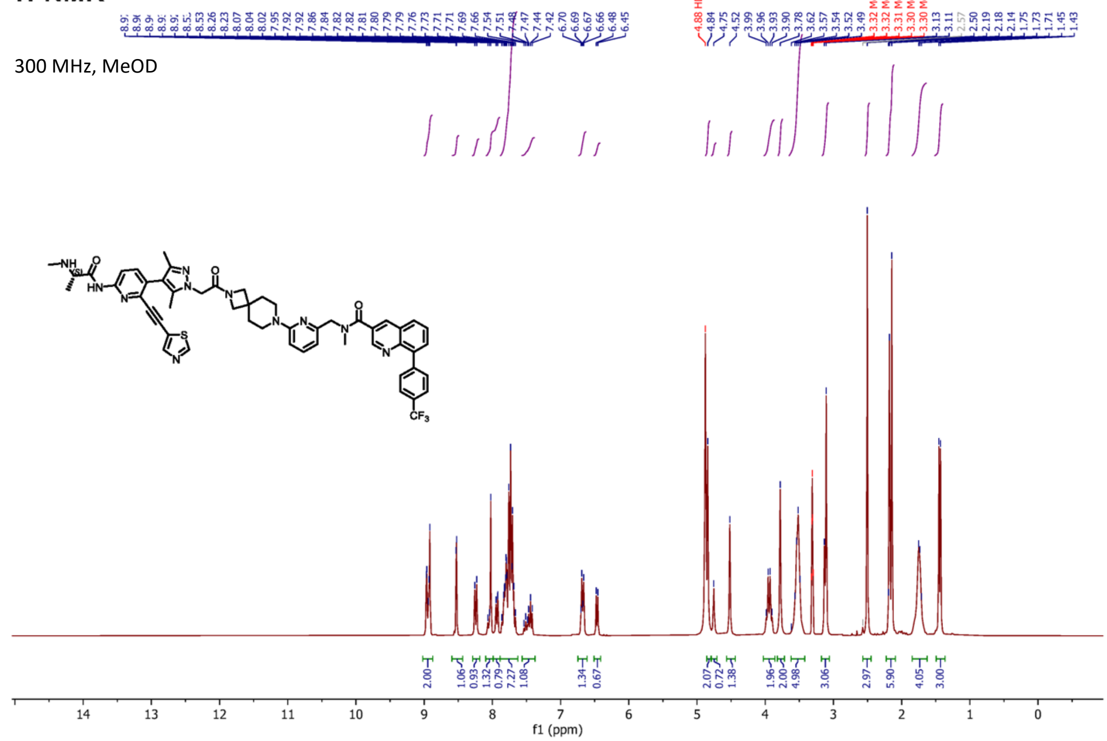

b. ^{13}C-NMR

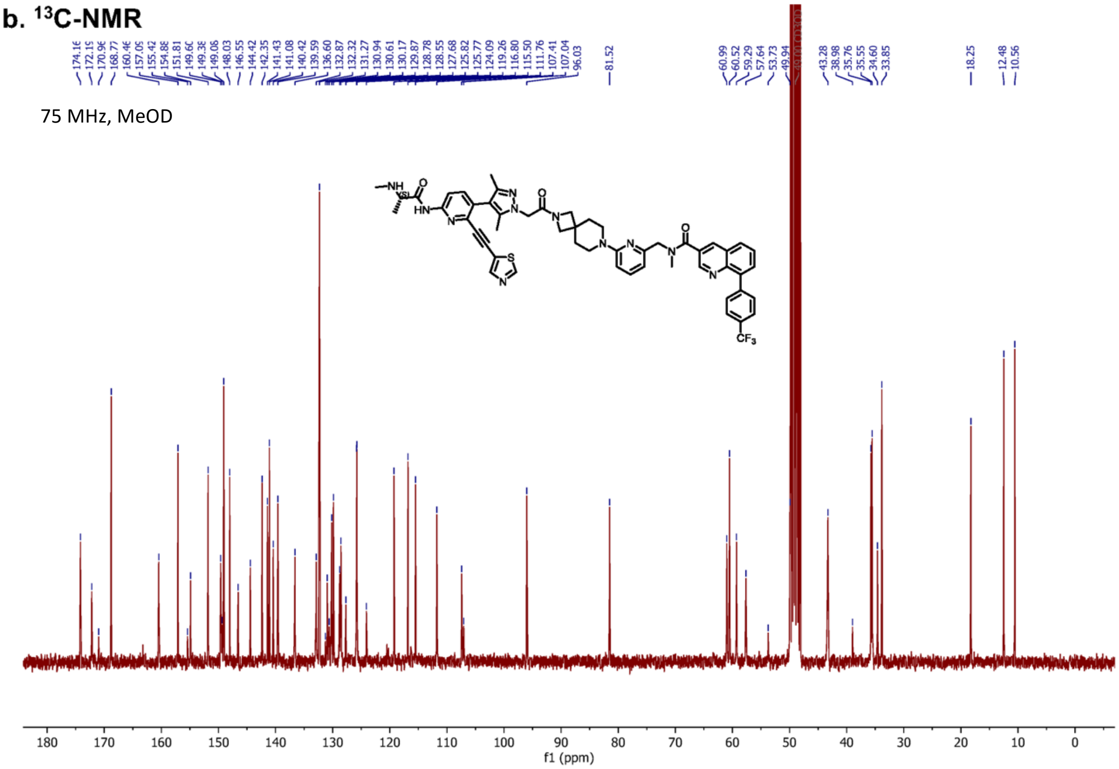

A560

c. ^{19}F-NMR

282 MHz, MeOD

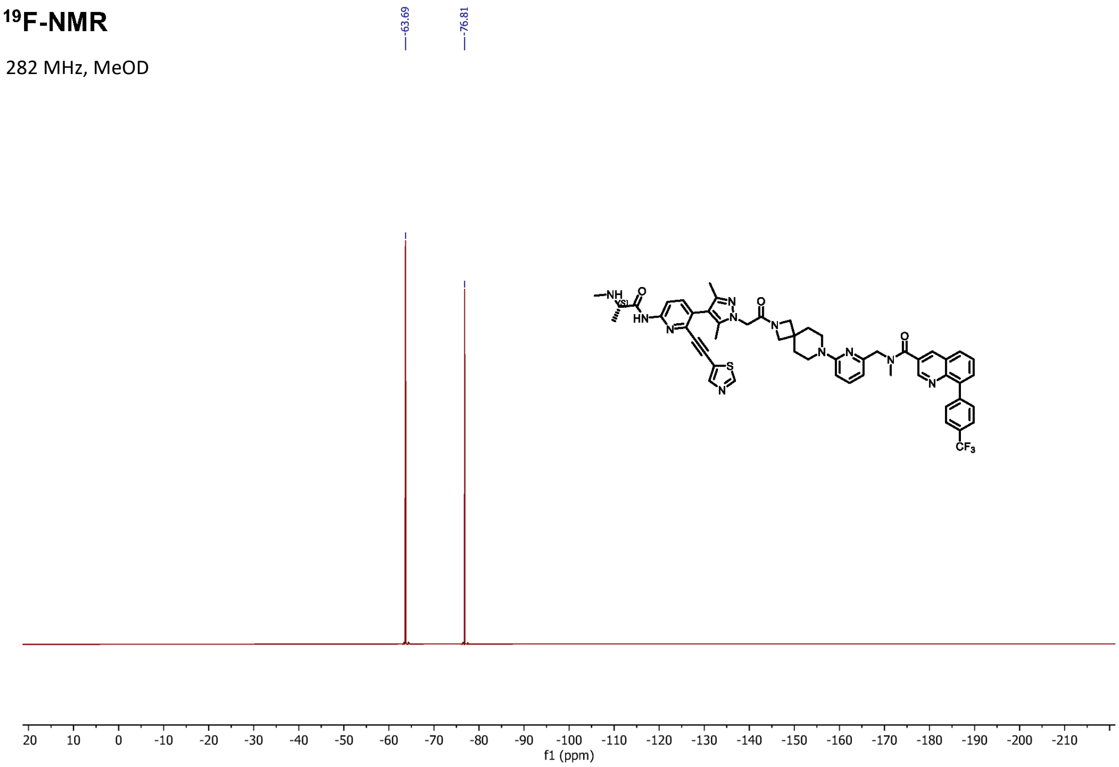

d. LCMS

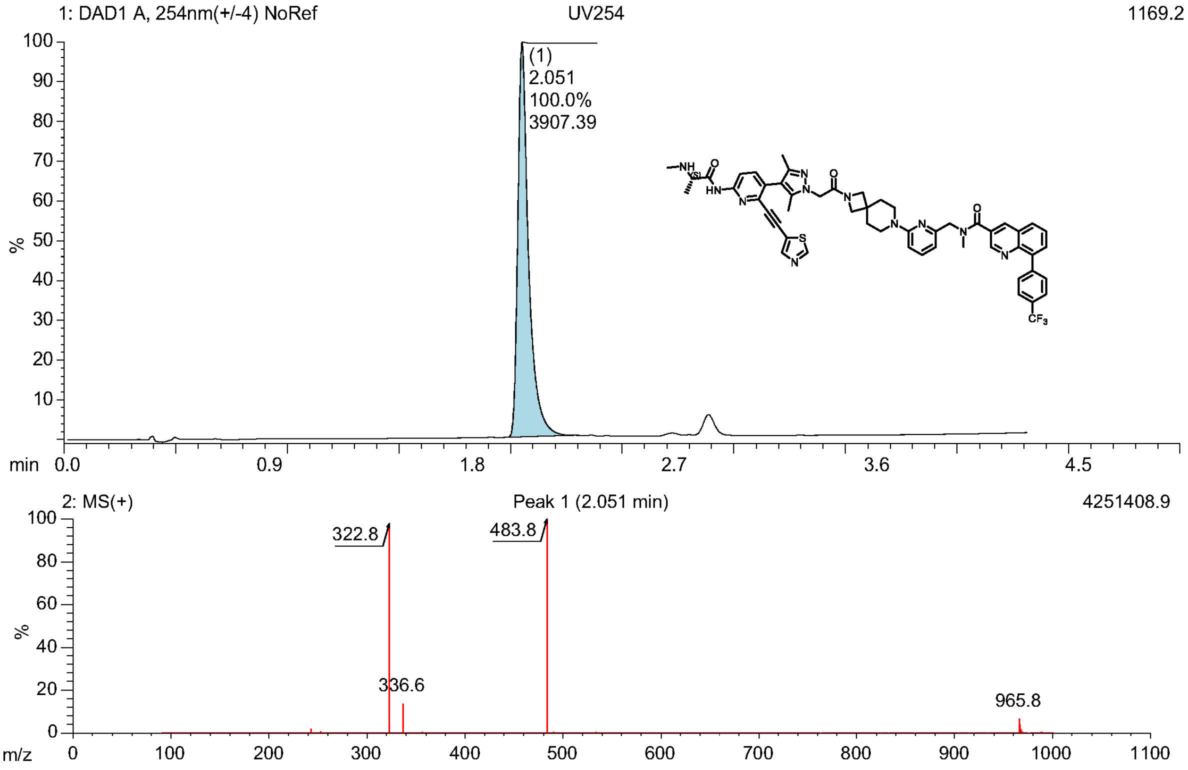

A560

e. HRMS

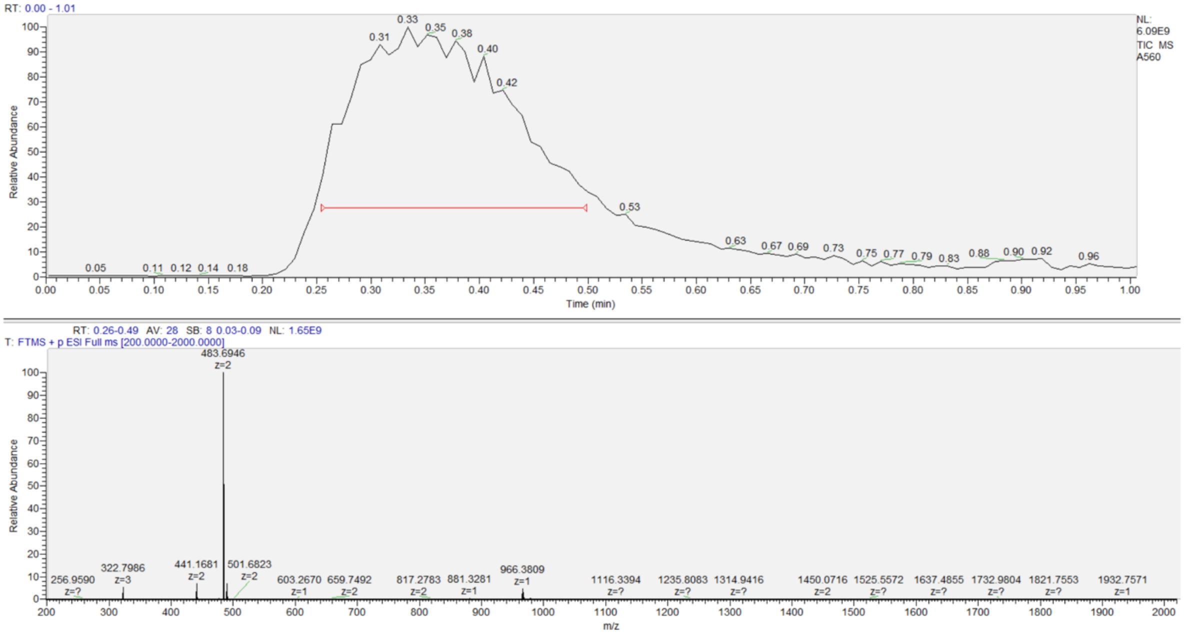

RT: 0.00 - 1.01
NL: 6.09E9
TIC MS
A560
Relative Abundance
Time (min)
RT: 0.26-0.49 AV: 28 SB: 8 0.03-0.09 NL: 1.65E9
T: FTMS + p ESI Full ms [200.0000-2000.0000]
483.6946
z=2
441.1681
z=2
501.6823
z=2
322.7986
z=3
256.9590
z=?
603.2670
z=1
659.7492
z=2
817.2783
z=2
881.3281
z=1
966.3809
z=1
1116.3394
z=?
1235.8083
z=?
1314.9416
z=?
1450.0716
z=2
1525.5572
z=?
1637.4855
z=?
1732.9804
z=?
1821.7553
z=?
1932.7571
z=1
m/z

A232

a. ^{1}H-NMR

300 MHz, MeOD

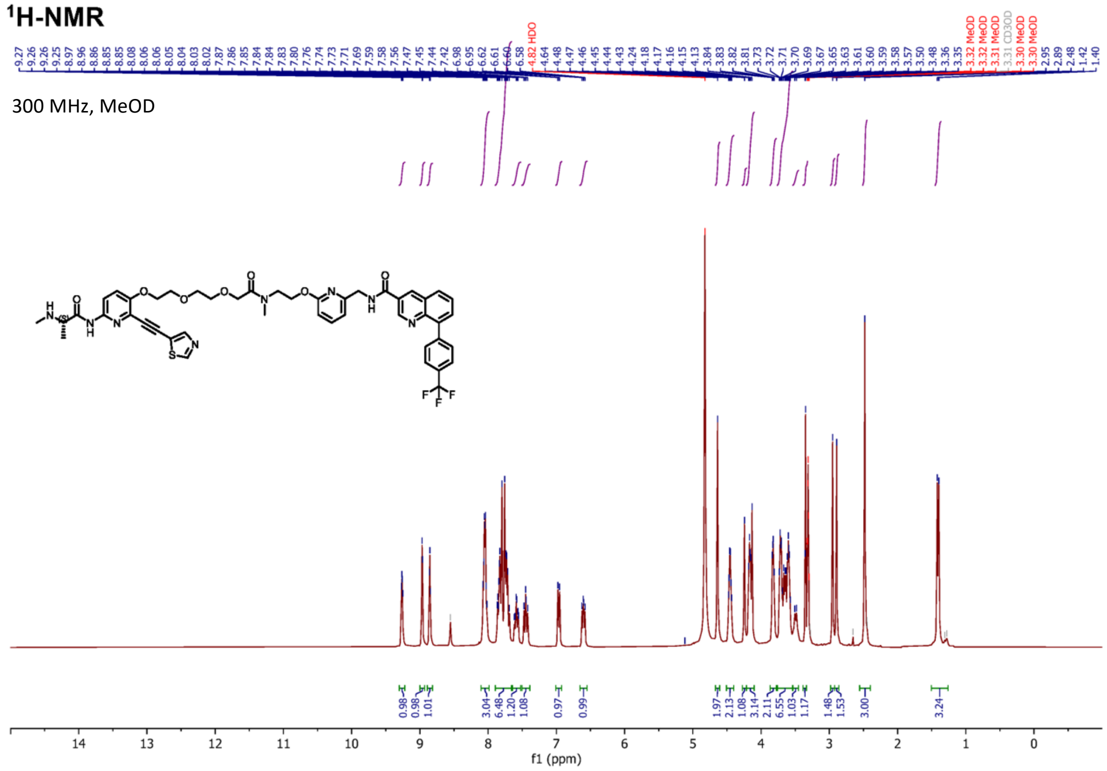

b. ^{13}C-NMR

75 MHz, MeOD

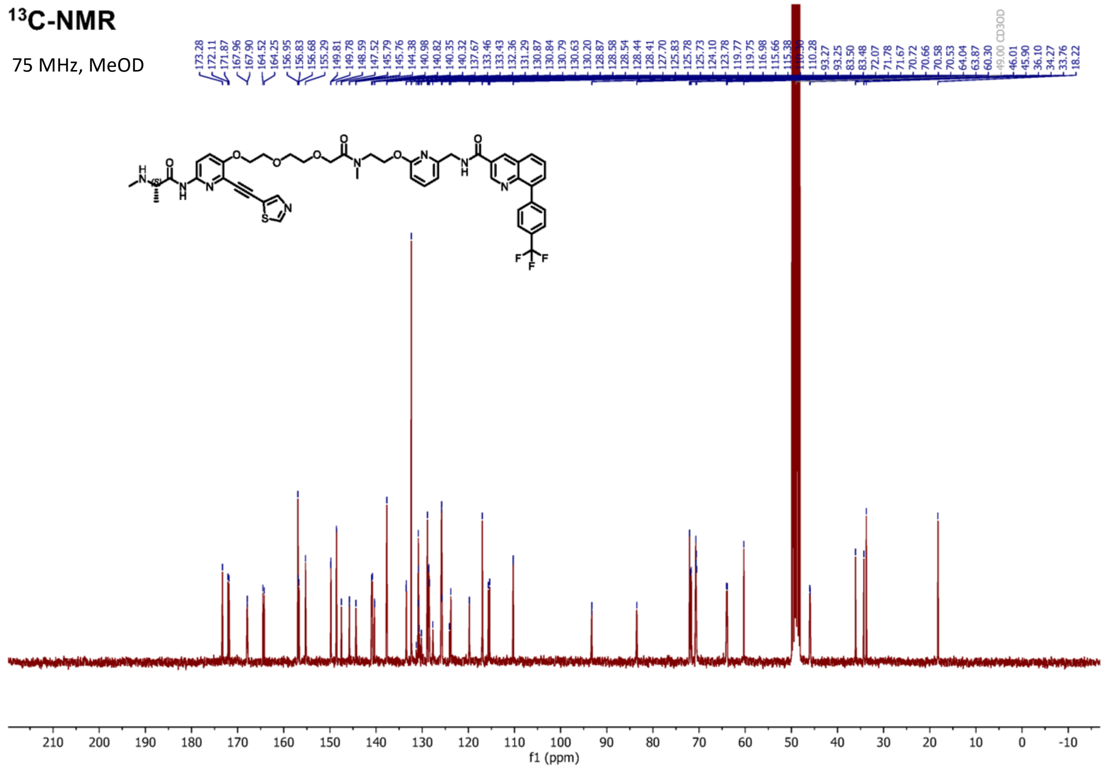

A232

c. ^{19}F-NMR

282 MHz, MeOD

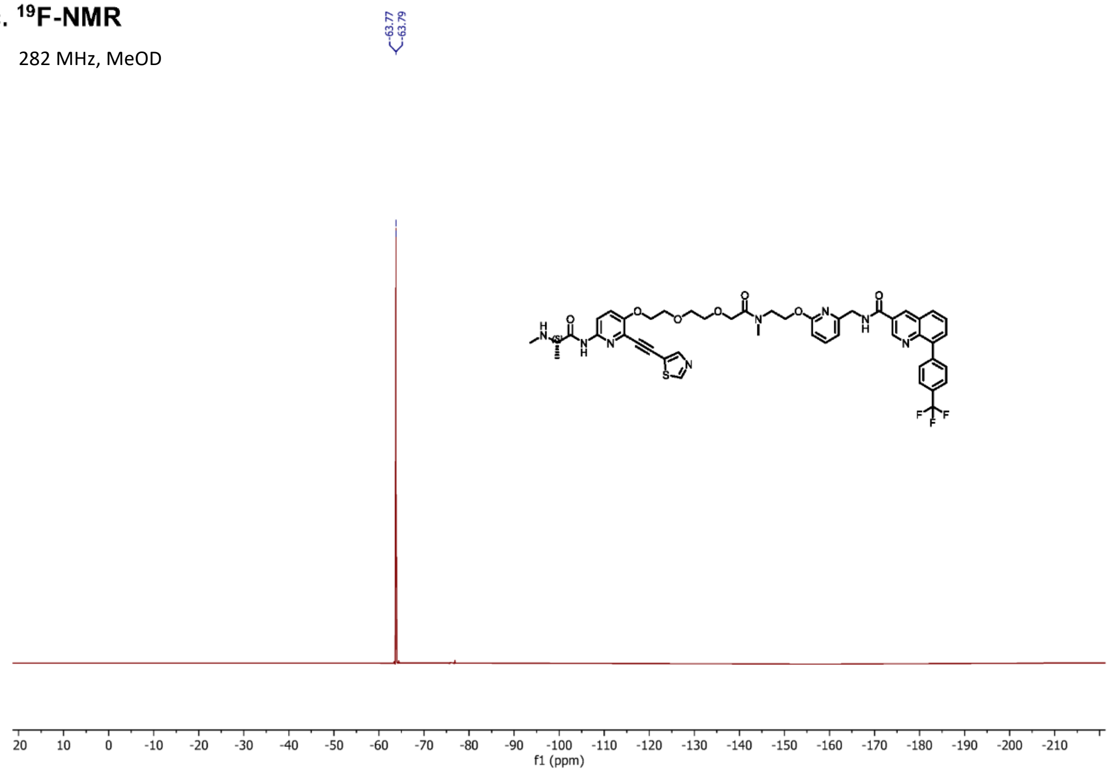

d. LCMS

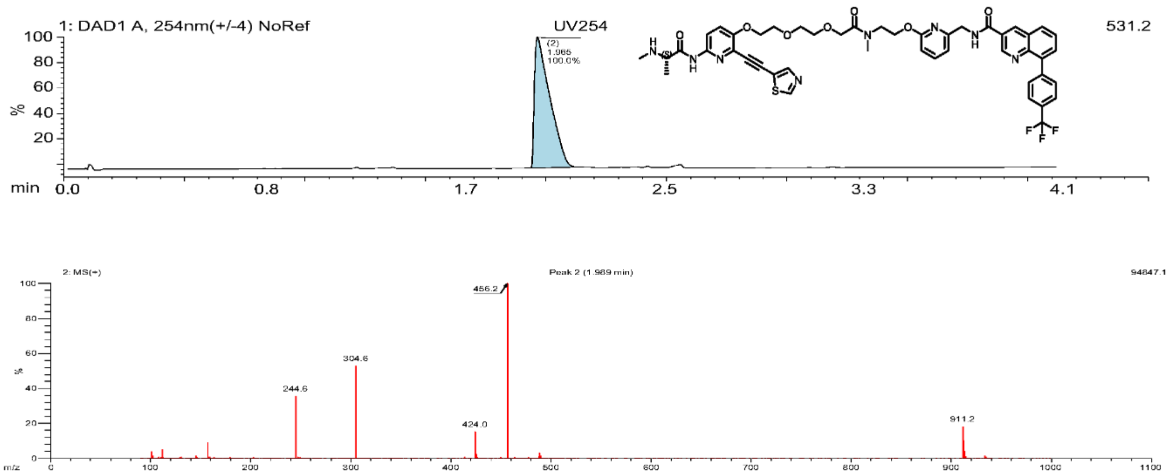

A232

e. HRMS

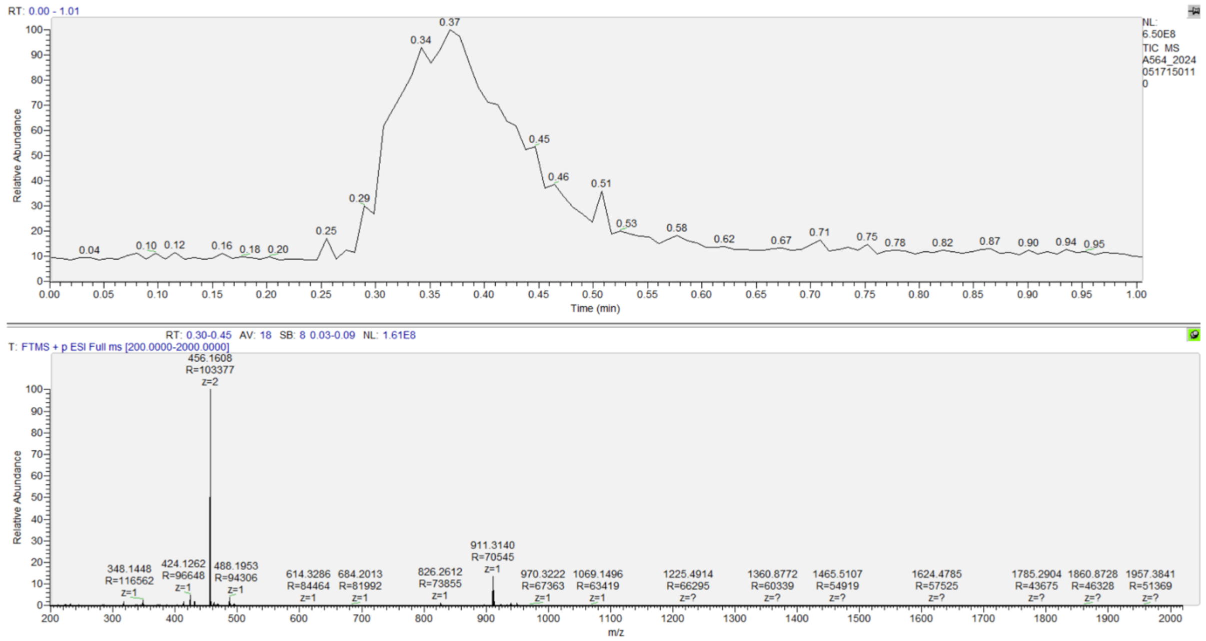

RT: 0.00 - 1.01
NL: 6.50E8 TIC MS A564_2024051715011 0
Relative Abundance
Time (min)
RT: 0.30-0.45 AV: 18 SB: 8 0.03-0.09 NL: 1.61E8
T: FTMS + p ESI Full ms [200.0000-2000.0000]
456.1608 R=103377 z=2
911.3140 R=70545 z=1
348.1448 R=116562 z=1
424.1262 R=96648 z=1
488.1953 R=94306 z=1
614.3286 R=84464 z=1
684.2013 R=81992 z=1
826.2612 R=73855 z=1
970.3222 R=67363 z=1
1069.1496 R=63419 z=1
1225.4914 R=66295 z=?
1360.8772 R=60339 z=?
1465.5107 R=54919 z=?
1624.4785 R=57525 z=?
1785.2904 R=43675 z=?
1860.8728 R=46328 z=?
1957.3841 R=51369 z=?
m/z

A230

a. ^{1}H-NMR

300 MHz, MeOD

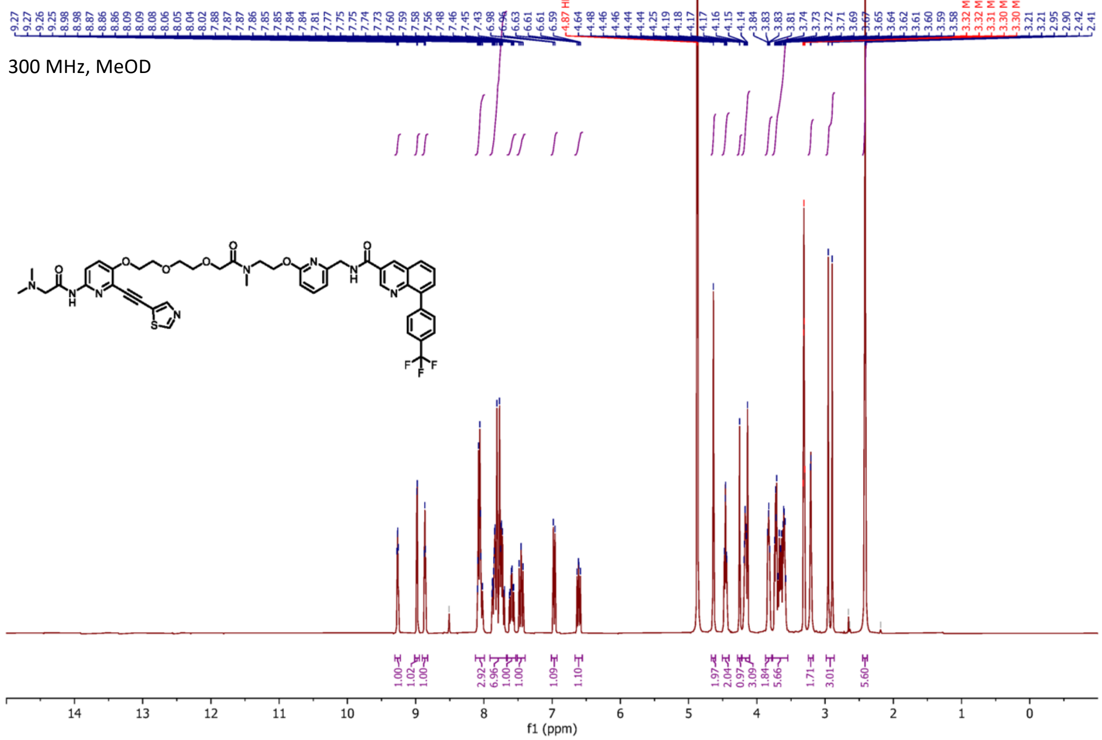

b. ^{13}C-NMR

75 MHz, MeOD

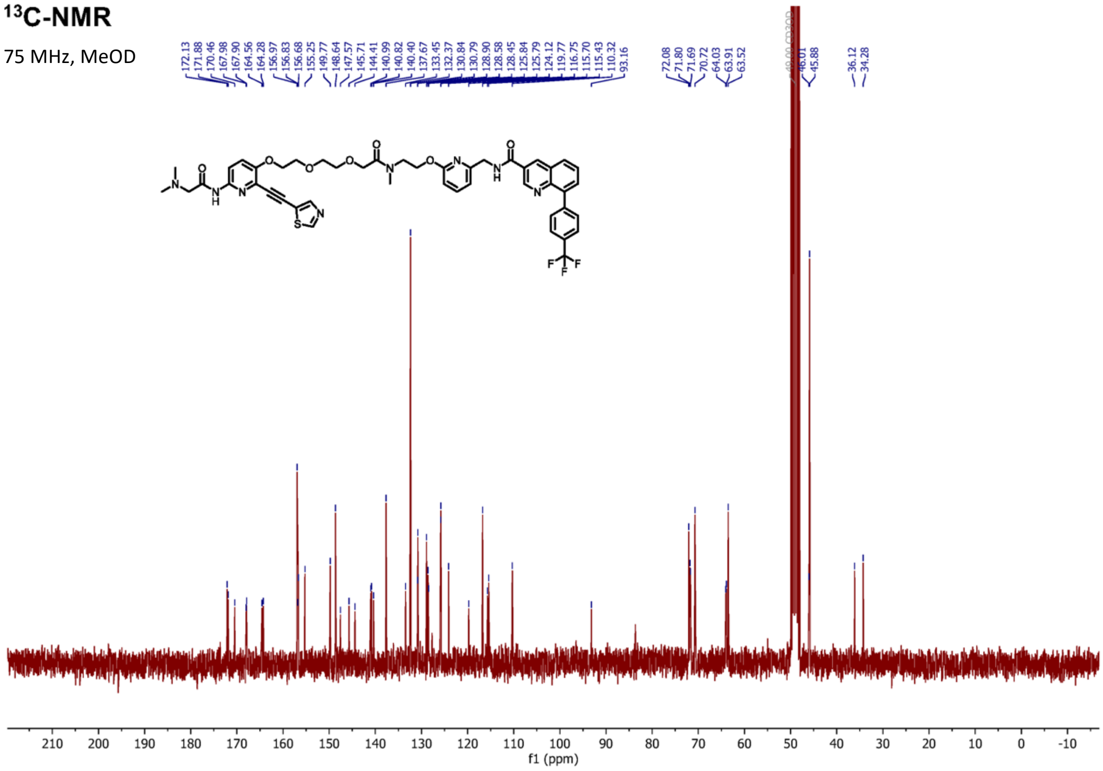

A230

c. ^{19}F-NMR

282 MHz, MeOD

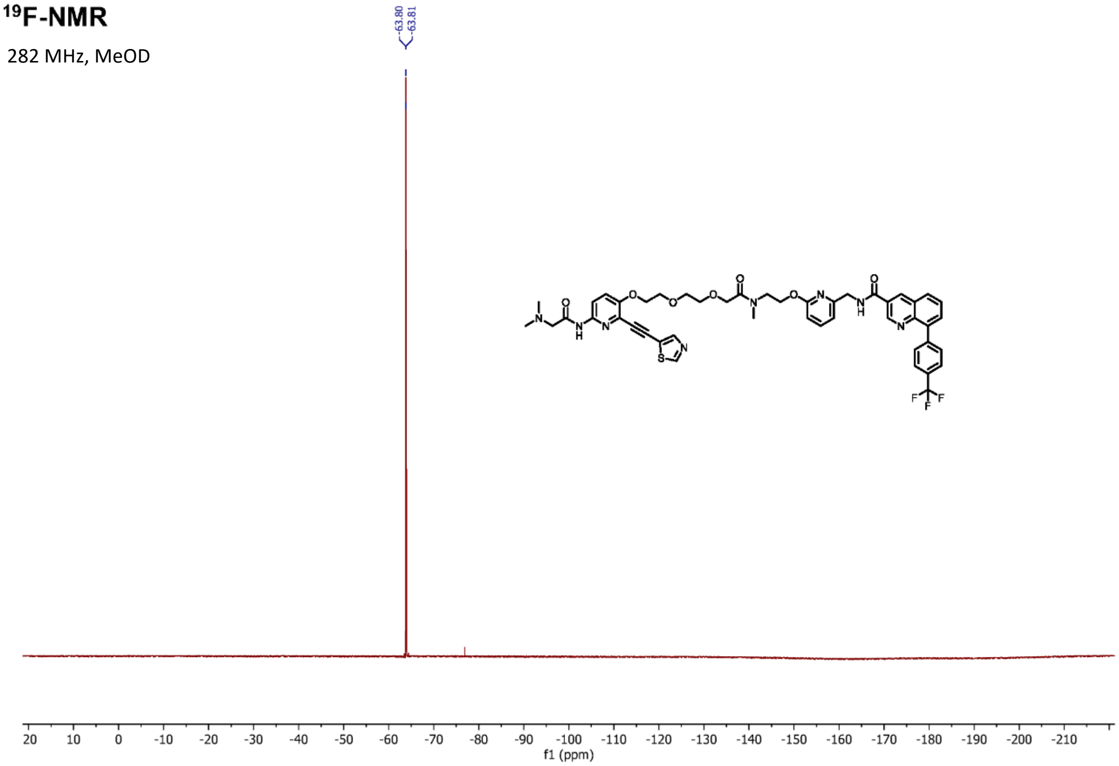

d. LCMS

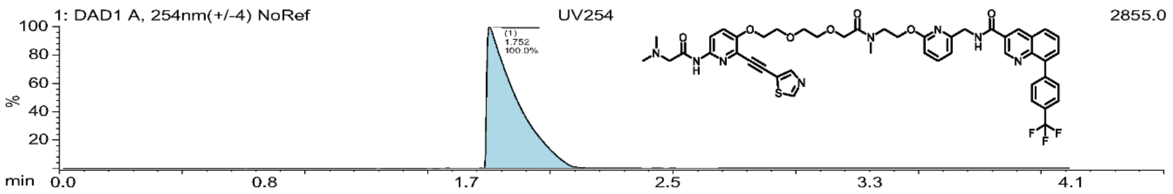

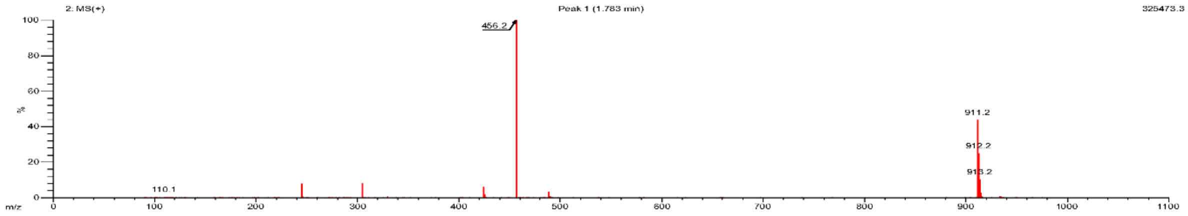

A230

e. HRMS

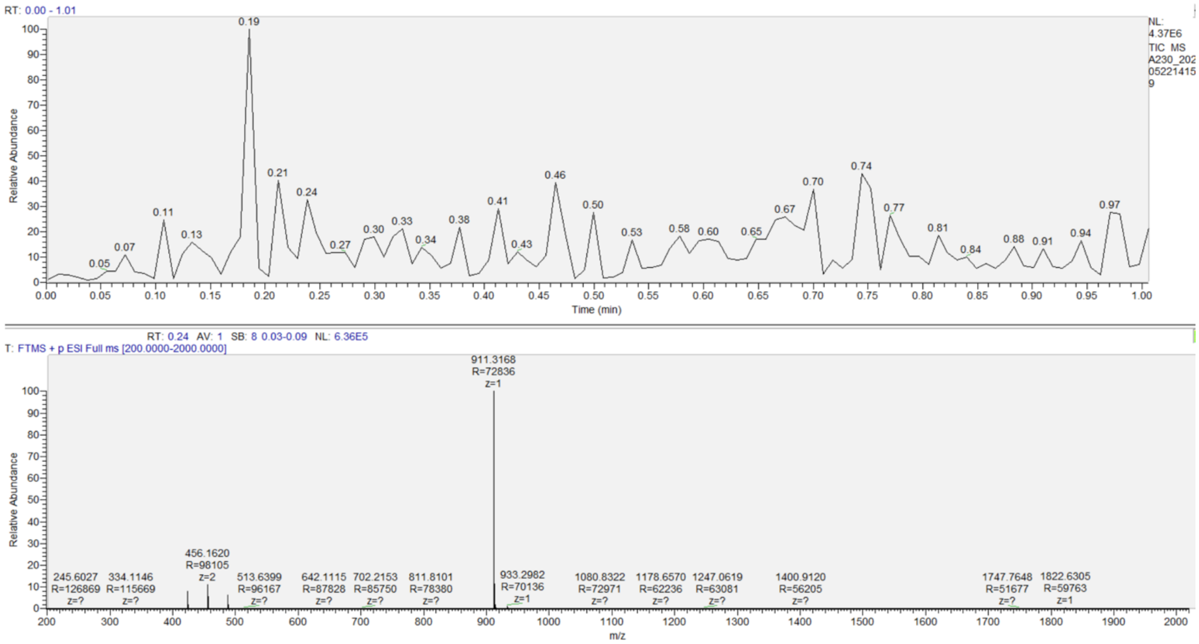
RT: 0.00 - 1.01
NL: 4.37E6 TIC MS A230_20205221415 9
Relative Abundance
Time (min)
RT: 0.24 AV: 1 SB: 8 0.03-0.09 NL: 6.36E5
T: FTMS + p ESI Full ms [200.0000-2000.0000]
911.3168 R=72836 z=1
456.1620 R=98105 z=2
933.2982 R=70136 z=1
1822.6305 R=59763 z=1
m/z

A231

a. ¹H-NMR

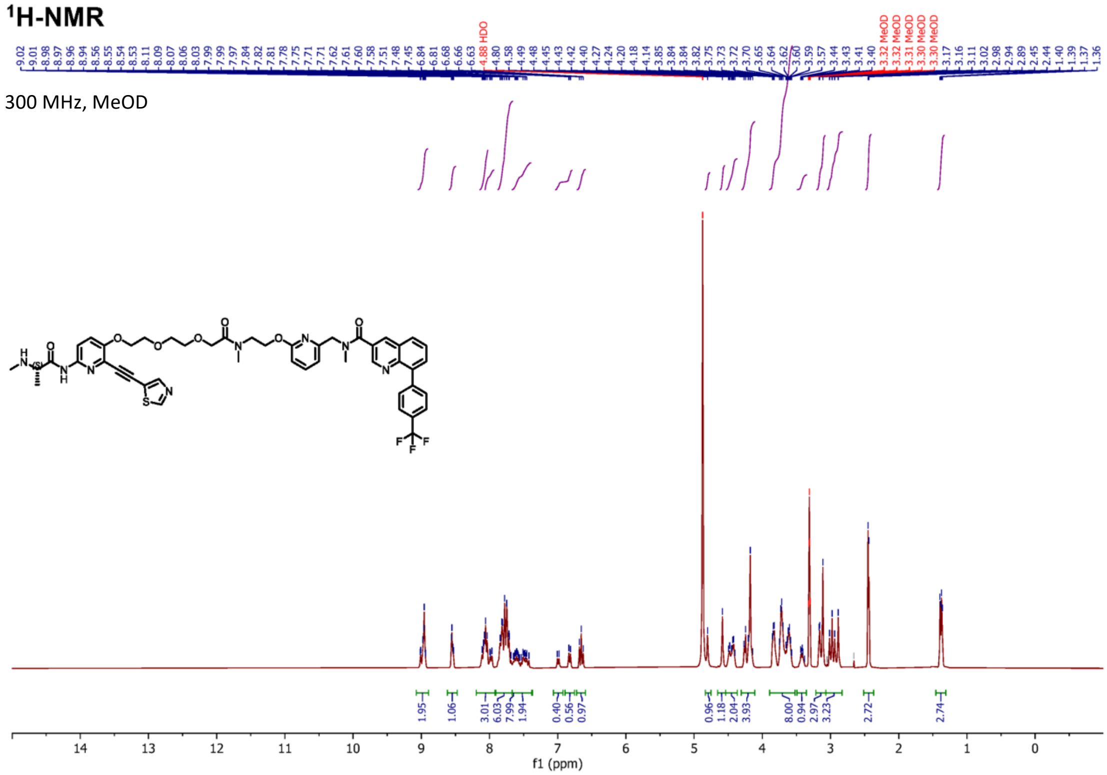

b. ¹³C-NMR

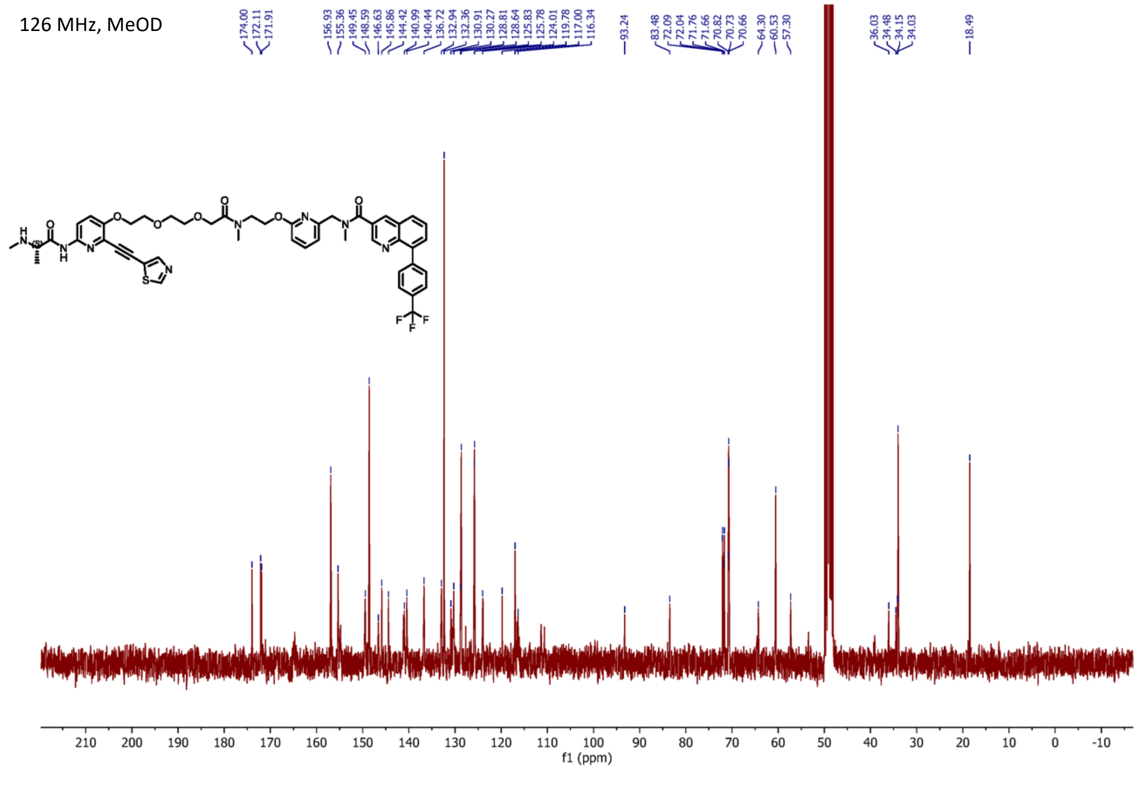

A231

c. ^{19}F-NMR

282 MHz, MeOD

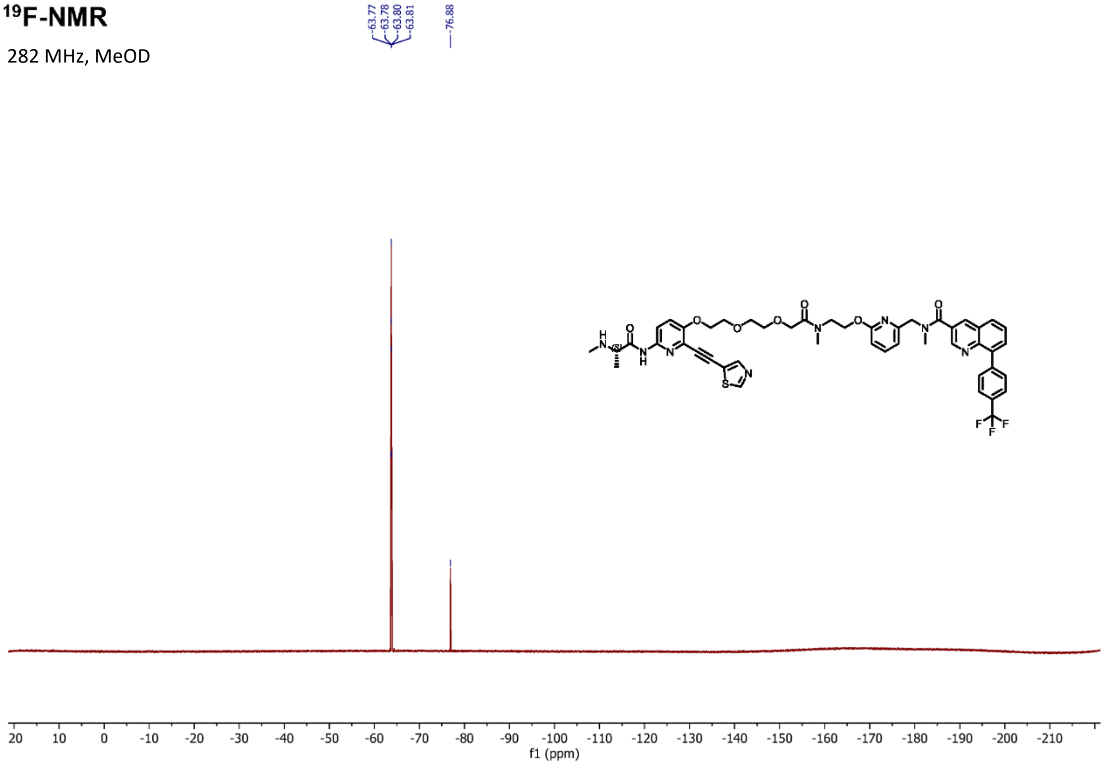

d. LCMS

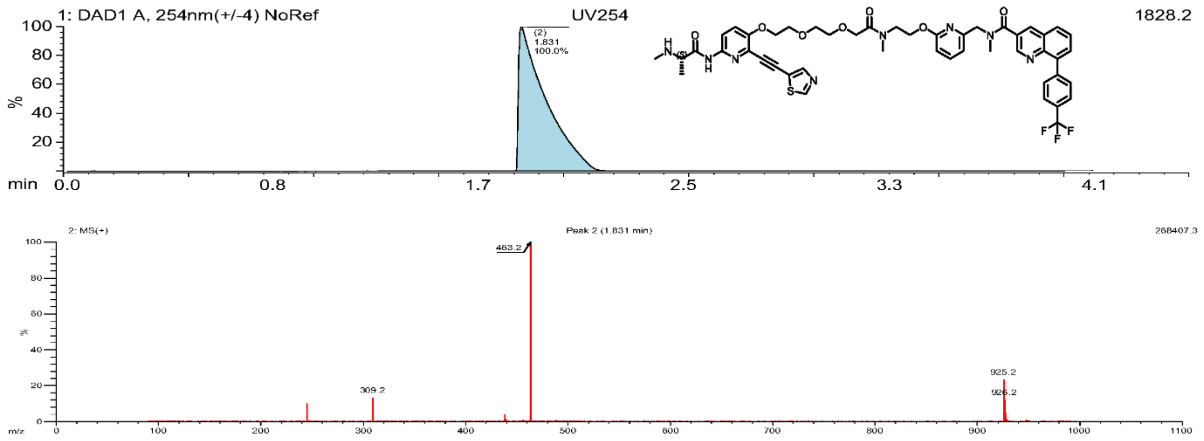

A231

e. HRMS

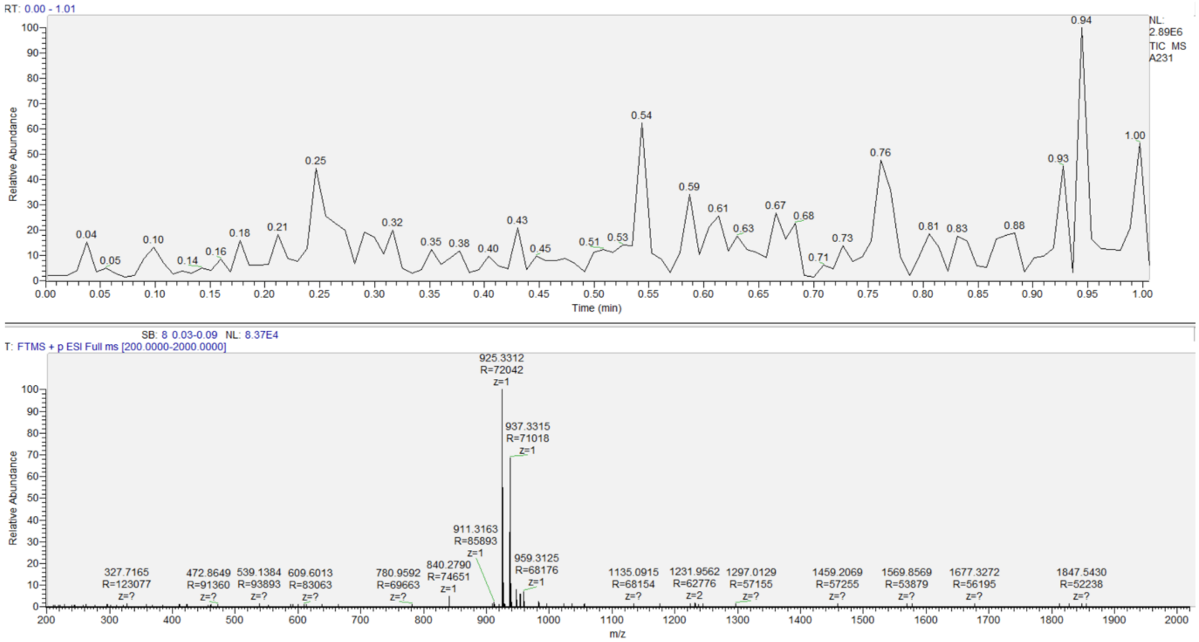

RT: 0.00 - 1.01
NL: 2.89E6 TIC MS A231
Relative Abundance
Time (min)
SB: 8 0.03-0.09 NL: 8.37E4
T: FTMS + p ESI Full ms [200.0000-2000.0000]
925.3312 R=72042 z=1
937.3315 R=71018 z=1
911.3163 R=85893 z=1
959.3125 R=68176 z=1
840.2790 R=74651 z=1
327.7165 R=123077 z=?
472.8649 R=91360 z=?
539.1384 R=93893 z=?
609.6013 R=83063 z=?
780.9592 R=69663 z=?
1135.0915 R=68154 z=?
1231.9562 R=62776 z=2
1297.0129 R=57155 z=?
1459.2069 R=57255 z=?
1569.8569 R=53879 z=?
1677.3272 R=56195 z=?
1847.5430 R=52238 z=?
m/z

A191

a. ¹H-NMR

300 MHz, $CDCl_3$

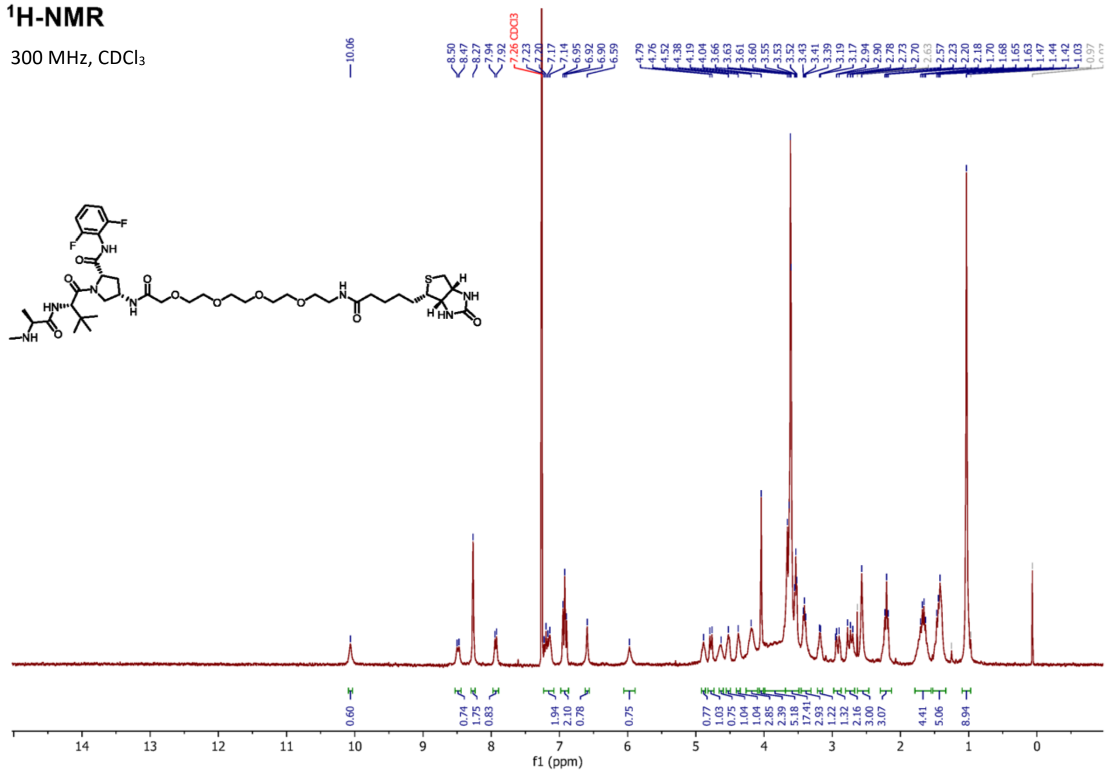

b. LCMS

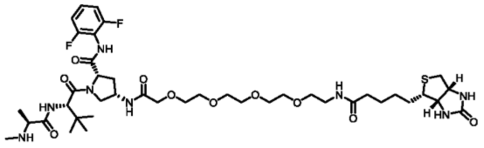

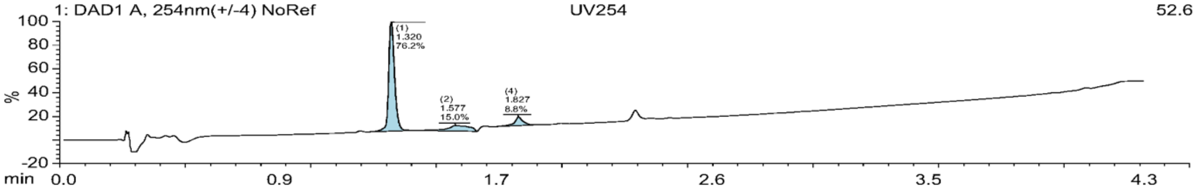

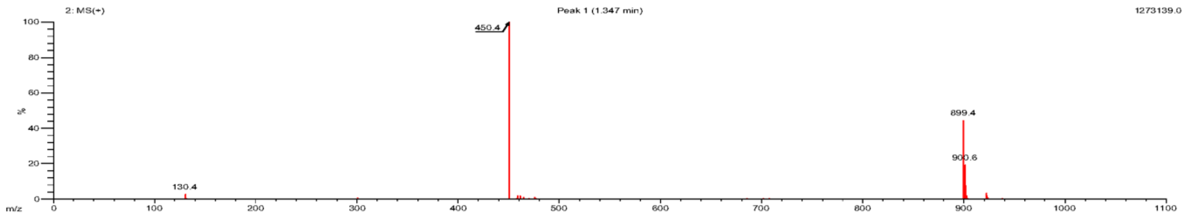

A191

c. HRMS

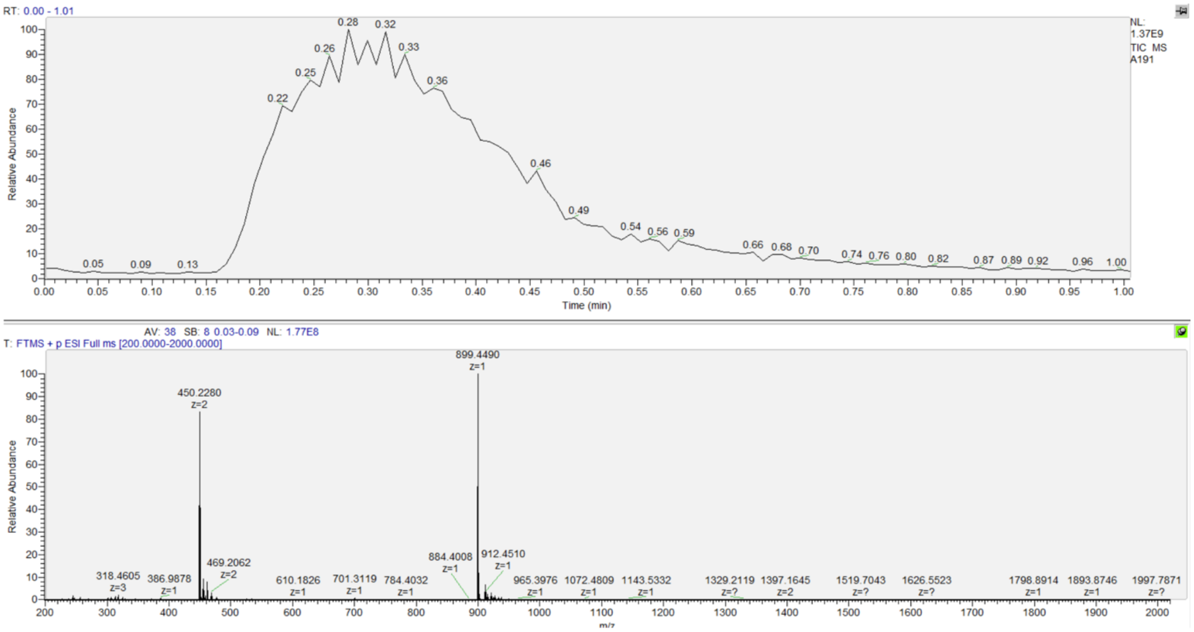
RT: 0.00 - 1.01
NL: 1.37E9 TIC MS A191
Relative Abundance
Time (min)
AV: 38 SB: 8 0.03-0.09 NL: 1.77E8
T: FTMS + p ESI Full ms [200.0000-2000.0000]
899.4490 z=1
450.2280 z=2
469.2062 z=2
884.4008 z=1
912.4510 z=1
318.4605 z=3
386.9878 z=1
610.1826 z=1
701.3119 z=1
784.4032 z=1
965.3976 z=1
1072.4809 z=1
1143.5332 z=1
1329.2119 z=?
1397.1645 z=2
1519.7043 z=?
1626.5523 z=?
1798.8914 z=1
1893.8746 z=1
1997.7871 z=?

B678

a. 1H-NMR

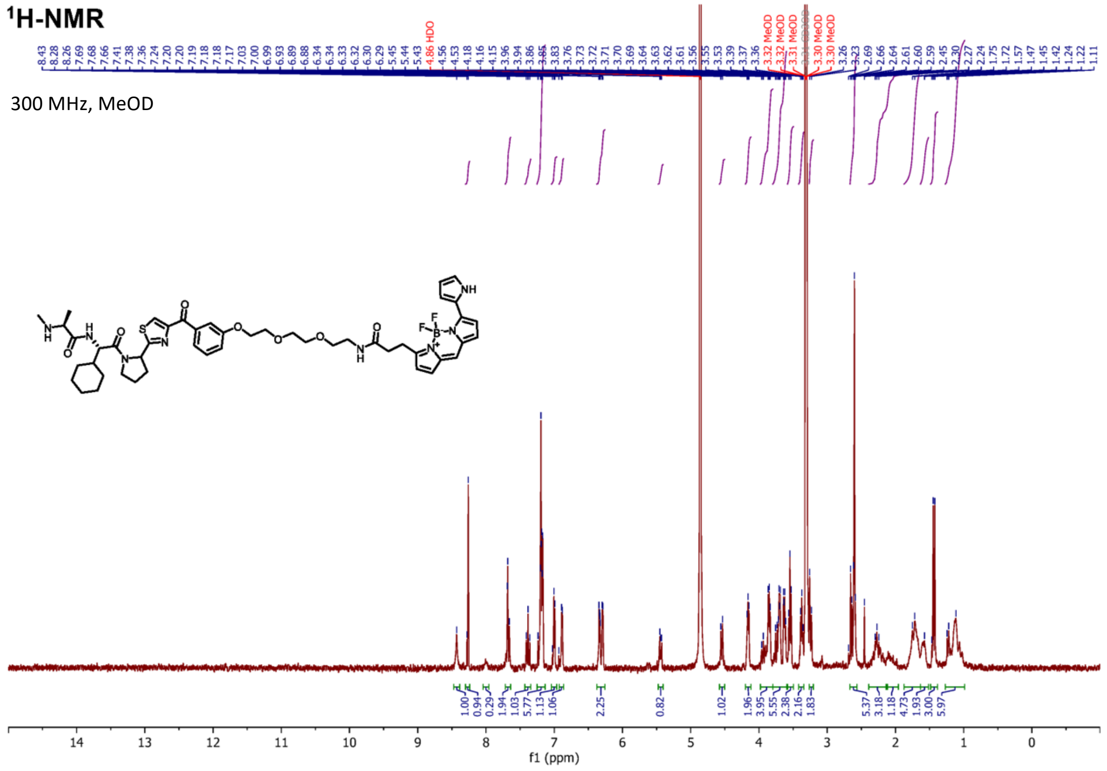

b. LCMS

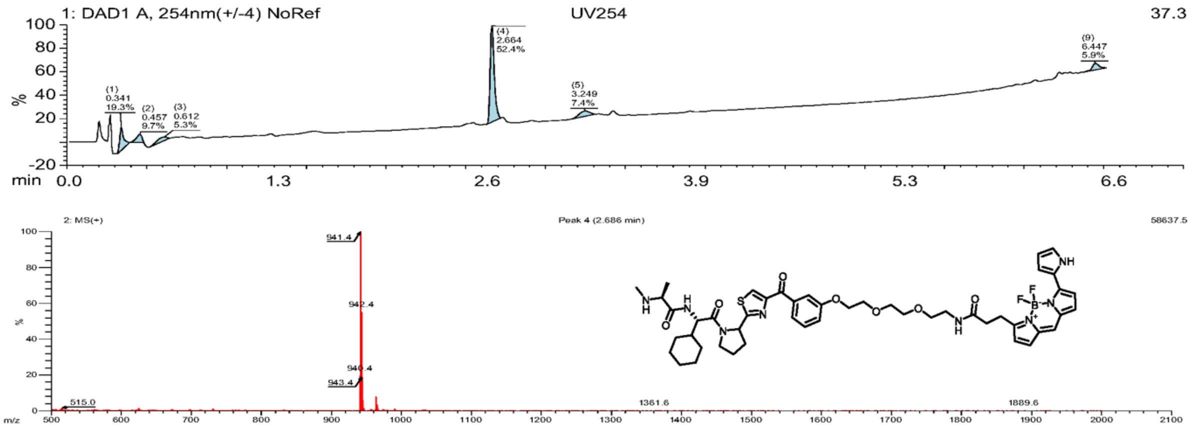

B678

c. HRMS

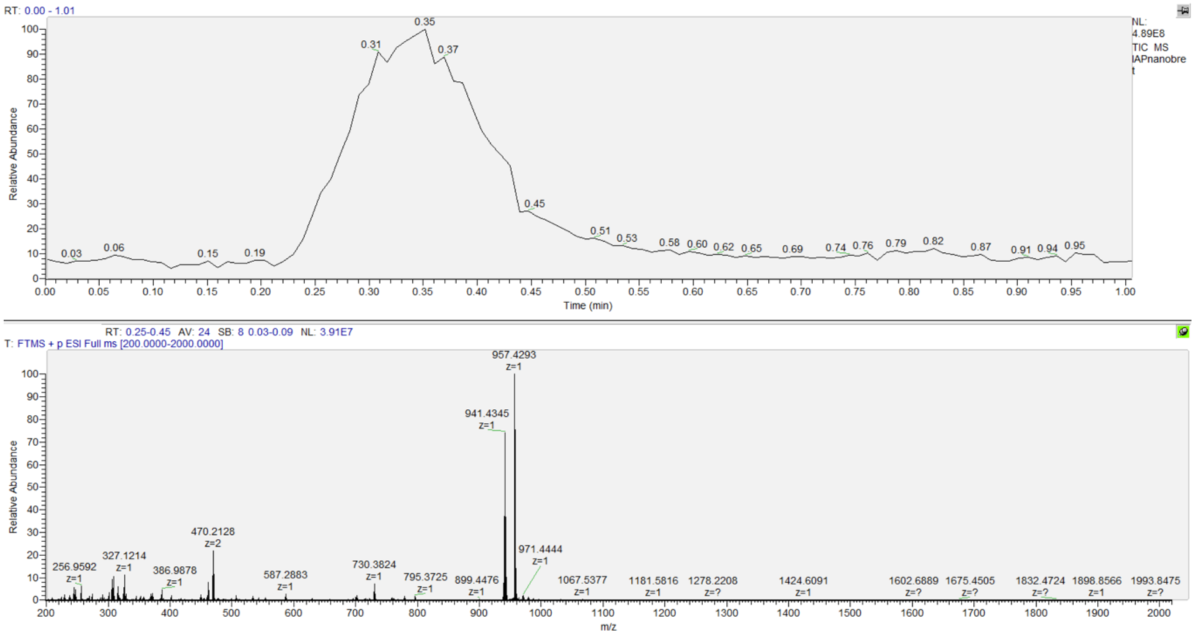

RT: 0.00 - 1.01
NL: 4.89E8 TIC MS IAPnanobret
Relative Abundance
Time (min)
RT: 0.25-0.45 AV: 24 SB: 8 0.03-0.09 NL: 3.91E7
T: FTMS + p ESI Full ms [200.0000-2000.0000]
957.4293 z=1
941.4345 z=1
971.4444 z=1
470.2128 z=2
327.1214 z=1
256.9592 z=1
386.9878 z=1
587.2883 z=1
730.3824 z=1
795.3725 z=1
899.4476 z=1
1067.5377 z=1
1181.5816 z=1
1278.2208 z=?
1424.6091 z=1
1602.6889 z=?
1675.4505 z=?
1832.4724 z=?
1898.8566 z=1
1993.8475 z=?
m/z

A472

a. ^{1}H-NMR

300 MHz, $CDCl_3$

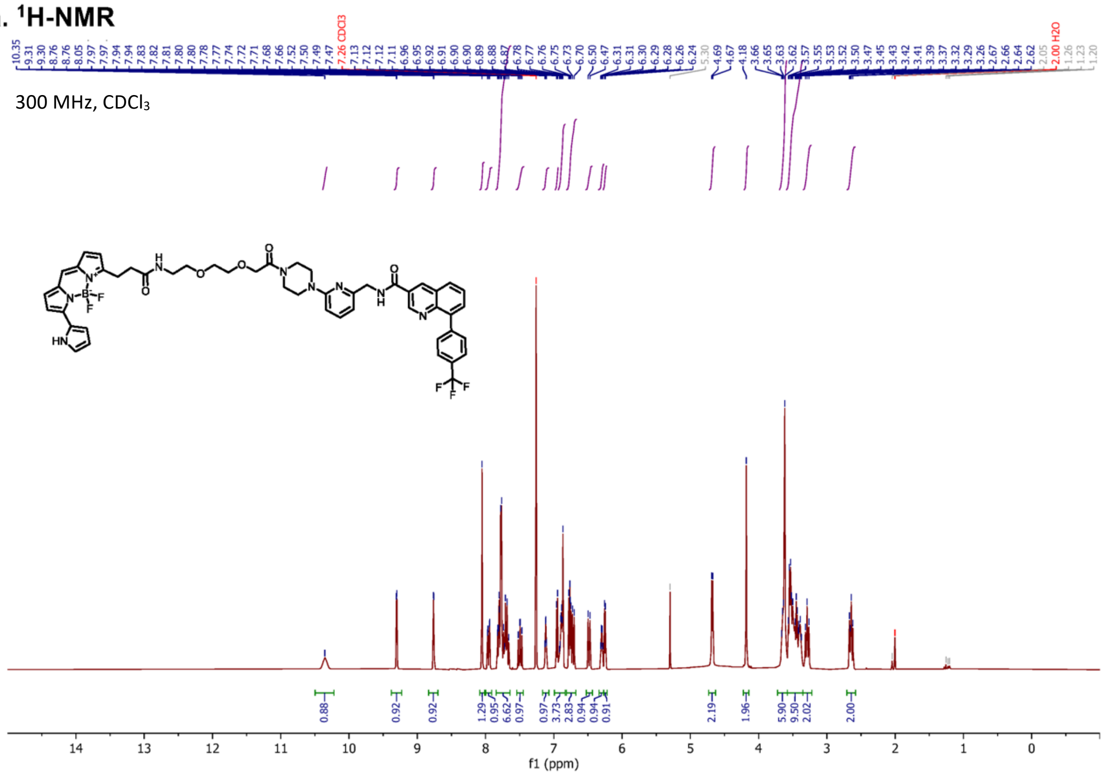

b. ^{19}F-NMR

282 MHz, $CDCl_3$

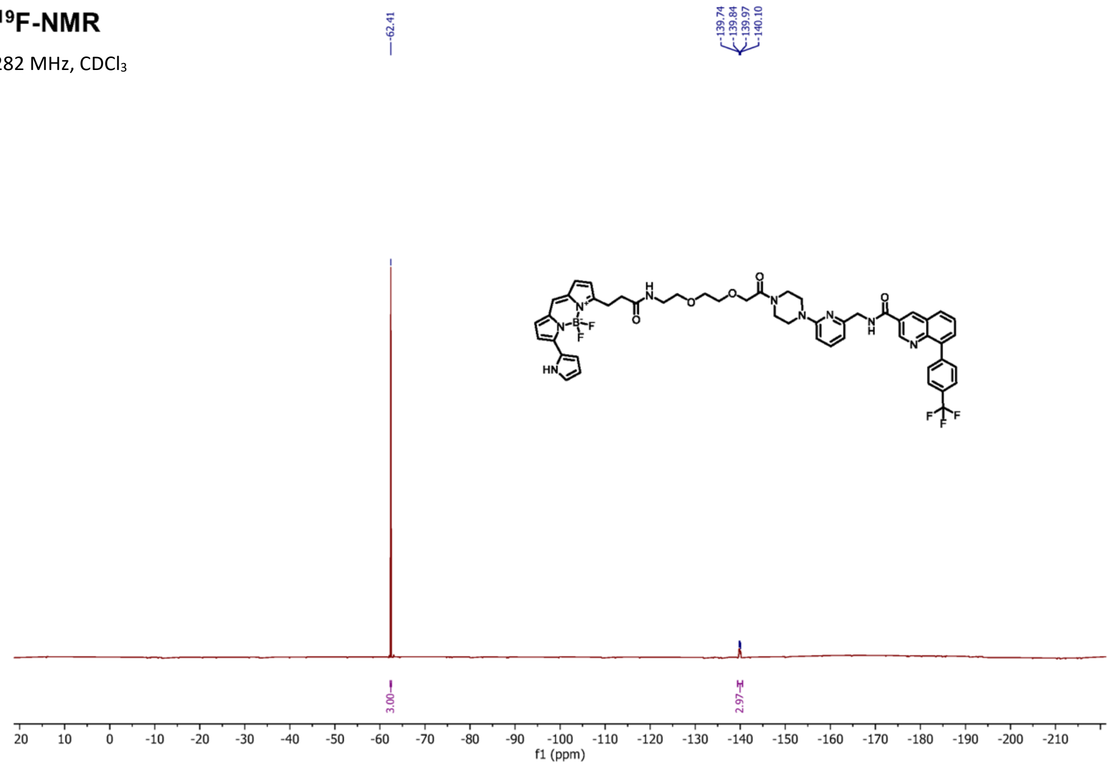

A472

c. LCMS

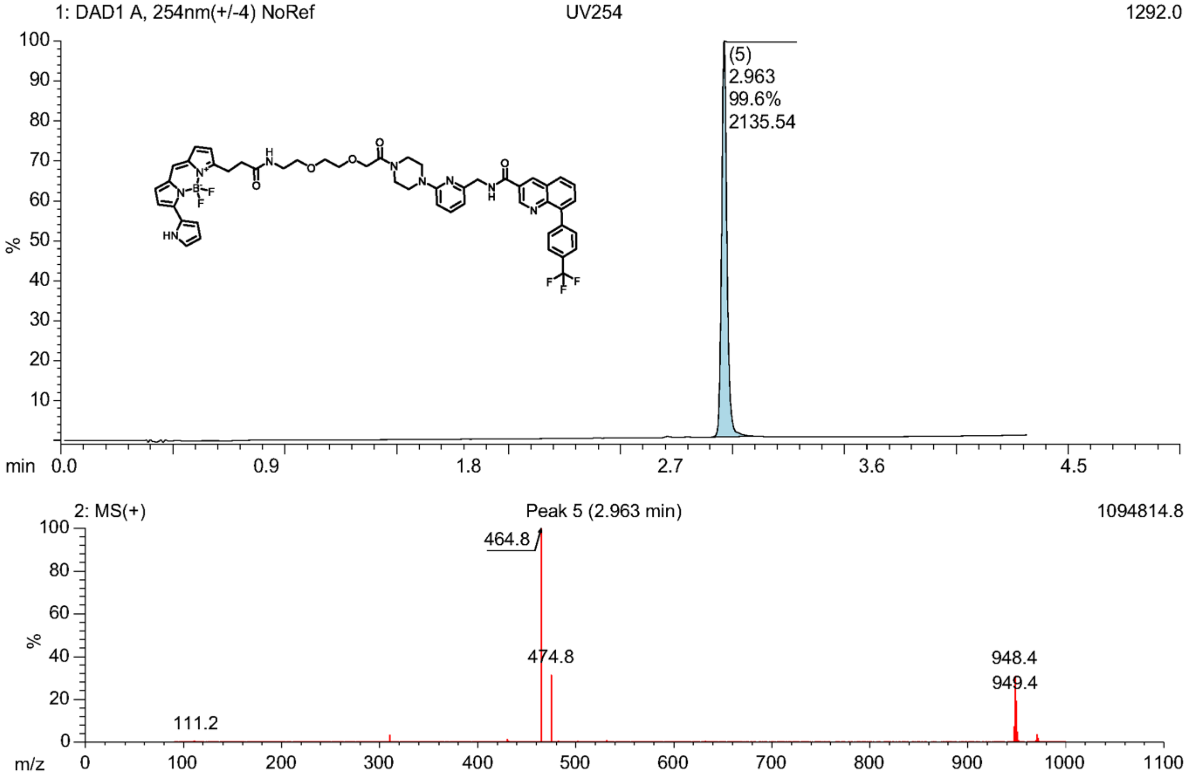

e. HRMS

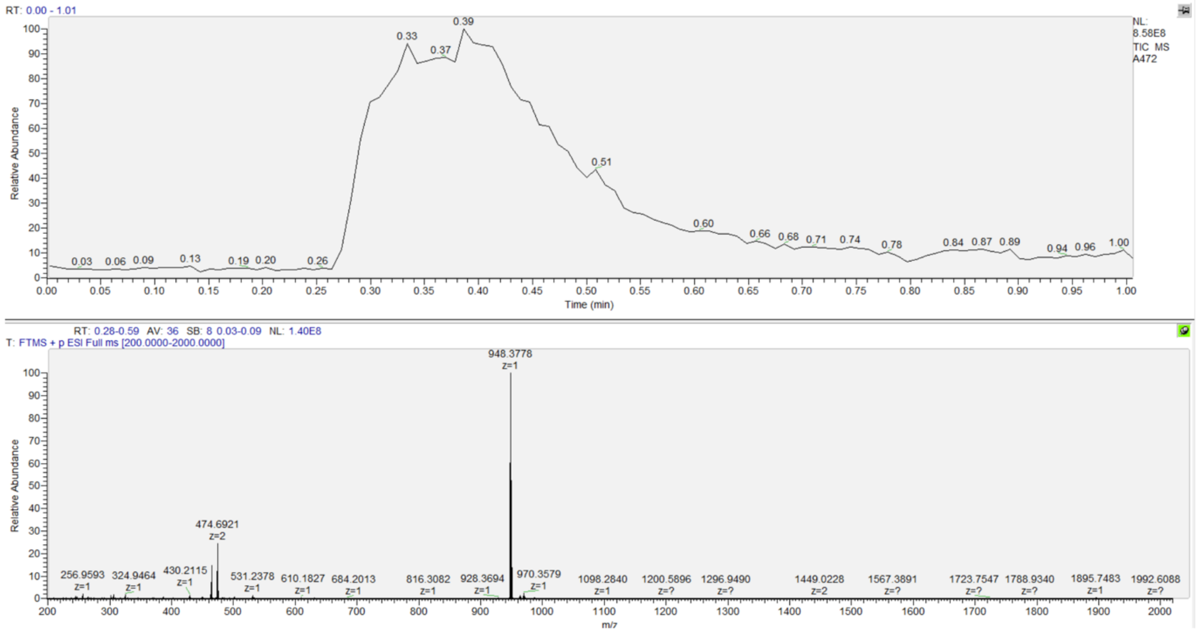

Abbreviations

Make

rt	room temperature
h	hours
min	minutes
d	days
RT	Retention Time
eq.	equivalents
prepLCMS	preparative LCMS
LCMS	liquid chromatography mass spectrometry
NMR	Nuclear Magnetic Resonance
DIPEA	*N*,*N*-Diisopropylethylamine
MeOH	Methanol
EtOAc	Ethyl acetate
MeCN	Acetonitrile
TFA	Trifluoroacetic acid
DCM	Dichloromethane
HRMS	High Resolution Mass Spectrometry
TLC	Thin layer chromatography
HPLC	High pressure liquid chromatography
THF	tetrahydrofuran
Boc	tert-butoxy carbonyl
HATU	Hexafluorophosphate Azabenzotriazole Tetramethyl Uronium
T3P	Propanephosphonic acid anhydride
DMF	*N*,*N*-dimethylformamide
Hept	*n*-Heptane
RP	Reverse Phase

SM	Starting material
Sat.	Saturated (in aqueous solution)
aq.	Aqueous
DCC	Dicyclohexylcarbodiimide
DCU	Dicyclohexylurea
dppf	1,1'-Bis(diphenylphosphino)ferrocene
dba	Dibenzylideneacetone
SCX	Strong Cation Exchange
approx.	approximately
NMP	*N*-methyl-2-pyrrolidone
BOP	benzotriazol-1-yloxytris(dimethylamino)phosphonium hexafluorophosphate
DIAD	Diisopropyl azodicarboxylate
Pet ether	Petroleum ethers
IAP	Inhibitors of Apoptosis
TEAD	transcriptional enhanced associate domain
ABC	Ammonium bicarbonate (as a solution)
DMA	*N*,*N*-dimethylacetamide
LiHMDS	Lithium bis(trimethylsilyl)amide

Supplementary References

1. Wu, G., *et al.* Structural basis of IAP recognition by Smac/DIABLO. *Nature* **408**, 1008-1012 (2000).

2. Pettersen, E. F., *et al.* UCSF ChimeraX: Structure visualization for researchers, educators, and developers. *Protein Sci.* **30**, 70-82 (2020).

3. Mace, P. D., *et al.* Structures of the cIAP2 RING Domain Reveal Conformational Changes Associated with Ubiquitin-conjugating Enzyme (E2) Recruitment. *J. Biol. Chem.* **283**, 31633-31640 (2008).

4. Feltham, R., *et al.* Smac Mimetics Activate the E3 Ligase Activity of cIAP1 Protein by Promoting RING Domain Dimerization. *J. Biol. Chem.* **286**, 17015-17028 (2011).

5. Dueber, E. C., *et al.* Antagonists Induce a Conformational Change in cIAP1 That Promotes Autoubiquitination. *Science* **334**, 376-380 (2011).

6. Nakatani, Y., Kleffmann T., Linke K., Condon S., Hinds M., Day C. Regulation of ubiquitin transfer by XIAP, a dimeric RING E3 ligase. *Biochem. J.* **450**, 629-638 (2013).

7. Polykretis, P., *et al.* Conformational characterization of full-length X-chromosome-linked inhibitor of apoptosis protein (XIAP) through an integrated approach. *IUCrJ* **6**, 948-957 (2019).

8. Tamanini, E., *et al.* Discovery of a Potent Nonpeptidomimetic, Small-Molecule Antagonist of Cellular Inhibitor of Apoptosis Protein 1 (cIAP1) and X-Linked Inhibitor of Apoptosis Protein (XIAP). *J. Med. Chem.* **60**, 4611-4625 (2017).

9. Tencer, A. H., *et al.* Molecular basis for nuclear accumulation and targeting of the inhibitor of apoptosis BIRC2. *Nat. Struct. Mol. Biol.* **30**, 1265-1274 (2023).
