# Supplementary material for: Expanding the toolbox to develop IAP-based degraders of TEAD transcription factors
Source: Commun Chem. 2026 Jan 19;9:69. doi: 10.1038/s42004-025-01871-x (PMC12873239; doi:10.1038/s42004-025-01871-x)
Supplement: Supplementary file 2 — Description of Additional Supplementary Files [file 42004_2025_1871_MOESM2_ESM.pdf]

Description of Supplementary Data Files

File name: *Supplementary Data 1*

Description – Source Data for Figures 1-7 and Supplementary Figures 1-6; Mycoplasma testing and compound numbering key for Source Data.

File name: *Supplementary Data 2*

Description –

Output tables (Worksheet 1) and sample key (Worksheet 2) from proteomics-based mass spectrometry analysis of NCI-H2052 cells treated with IPD **A538**, matched IAP-negative control **A559**, matched TEAD-negative control **A561**.

List (Worksheet 3) comparing union of all significantly downregulated proteins (FDR ≤ 0.05, Fold change: Negative 1.25) in NCI-H2052 cells treated with IPD **A538,** matched IAP-negative control **A559**, matched TEAD-negative control **A561**.

File Name: *Supplementary Data 3*

Description - List of all identified proteins (Worksheet 1) and significant proteins (FDR ≤ 0.05, Worksheet 2) from proteomics-based mass spectrometry analysis of NCI-H2052 cells treated with IPD **A538** versus DMSO.

File Name: *Supplementary Data 4*

Description - List of all identified proteins (Worksheet 1) and significant proteins (FDR ≤ 0.05, Worksheet 2) from proteomics-based mass spectrometry analysis of NCI-H2052 cells treated with IPD **A538** versus matched IAP-negative control **A559**.

File Name - *Supplementary Data 5*

Description - List of all identified proteins (Worksheet 1) and significant proteins (FDR ≤ 0.05, Worksheet 2) from proteomics-based mass spectrometry analysis of NCI-H2052 cells treated with IPD **A538** versus matched TEAD-negative control **A561**.
