# Supplementary material for: Expanding the toolbox to develop IAP-based degraders of TEAD transcription factors
Source: Commun Chem. 2026 Jan 19;9:69. doi: 10.1038/s42004-025-01871-x (PMC12873239; doi:10.1038/s42004-025-01871-x)
Supplement: Supplementary file 8 — Life Sciences Reporting Summary [file 42004_2025_1871_MOESM8_ESM.pdf]

nature portfolio

Corresponding author(s): Michael J. Roy; Nicola A. E. Chessum

Last updated by author(s): Dec 4, 2025

Reporting Summary

Nature Portfolio wishes to improve the reproducibility of the work that we publish. This form provides structure for consistency and transparency in reporting. For further information on Nature Portfolio policies, see our Editorial Policies and the Editorial Policy Checklist.

Statistics

For all statistical analyses, confirm that the following items are present in the figure legend, table legend, main text, or Methods section.

n/a	Confirmed	
☐	☒	The exact sample size (*n*) for each experimental group/condition, given as a discrete number and unit of measurement
☐	☒	A statement on whether measurements were taken from distinct samples or whether the same sample was measured repeatedly
☒	☐	The statistical test(s) used AND whether they are one- or two-sided<br>*Only common tests should be described solely by name; describe more complex techniques in the Methods section.*
☒	☐	A description of all covariates tested
☒	☐	A description of any assumptions or corrections, such as tests of normality and adjustment for multiple comparisons
☐	☒	A full description of the statistical parameters including central tendency (e.g. means) or other basic estimates (e.g. regression coefficient) AND variation (e.g. standard deviation) or associated estimates of uncertainty (e.g. confidence intervals)
☒	☐	For null hypothesis testing, the test statistic (e.g. *F*, *t*, *r*) with confidence intervals, effect sizes, degrees of freedom and *P* value noted<br>*Give P values as exact values whenever suitable.*
☒	☐	For Bayesian analysis, information on the choice of priors and Markov chain Monte Carlo settings
☒	☐	For hierarchical and complex designs, identification of the appropriate level for tests and full reporting of outcomes
☒	☐	Estimates of effect sizes (e.g. Cohen's *d*, Pearson's *r*), indicating how they were calculated

Our web collection on statistics for biologists contains articles on many of the points above.

Software and code

Policy information about availability of computer code

Data collection	Biacore 8K Control Software (Cytiva, v. 3.0.12.15655), CLARIOstarPlus plate reader control software (BMG Labtech, v 5.70 R3), EnVision Manager Software (PerkinElmer, v 1.14), Compass for Simple Western software (ProteinSimple, v 6.1.0), Image Lab Touch Software (Bio-Rad, v 2.4.0.03).<br>X-ray crystallography data collection was performed on the MX2 beamline at the Australian Synchrotron. The beamline/data collection were controlled using their standard in-house software (Blu-Ice, v. 5.0).
Data analysis	AIMLESS (v. 0.5.21), Biacore Insight Evaluation Software (Cytiva, v. 3.0.12.15655), COOT (v. 0.9.8.1), Fiji (ImageJ) software (v. 2.0.0), PHASER (v. 2.8.3), PHENIX (v. 1.21.1-5286), XSCALE (v. 20161205), XDS (v. 20161205), Pymol (Schrodinger, v. 2.5.4), ChimeraX (UCSF, v. 1.5), GraphPad Prism (v.10.3.1), InDesign (Adobe, v.18.2.1), Illustrator (Adobe, v.27.5), Affinity Photo 2 and Designer 2 (Affinity, v 2.5.7), Fiji (ImageJ) software (version: 2.0.0). Compass for Simple Western software (ProteinSimple; Version 6.1.0). Microsoft Excel (Microsoft, v 16.9.2). Spectronaut Software (Biognosys Inc, v. 20.0), R software (R-project, v. 4.4.2), limma R package (v. 3.62.2), ggplot2 R package (https://ggplot2.tidyverse.org), Harmonizome (version 3.0; https://maayanlab.cloud/Harmonizome/).

For manuscripts utilizing custom algorithms or software that are central to the research but not yet described in published literature, software must be made available to editors and reviewers. We strongly encourage code deposition in a community repository (e.g. GitHub). See the Nature Portfolio guidelines for submitting code & software for further information.

Data

Policy information about availability of data

All manuscripts must include a data availability statement. This statement should provide the following information, where applicable:

- Accession codes, unique identifiers, or web links for publicly available datasets
- A description of any restrictions on data availability
- For clinical datasets or third party data, please ensure that the statement adheres to our policy

Coordinates and structure factors for the X-ray crystal structures have been deposited in the PDB with accession codes 9N1R (XIAP-BIR3:A171), 9N21 (XIAP-BIR3:A250) and 9N23 (cIAP1-BIR3:A273).The mass spectrometry proteomics data have been deposited to the ProteomeXchange Consortium via the PRIDE partner repository with the dataset identifier PXD068528.

Research involving human participants, their data, or biological material

Policy information about studies with human participants or human data. See also policy information about sex, gender (identity/presentation), and sexual orientation and race, ethnicity and racism.

Reporting on sex and gender	N/A
Reporting on race, ethnicity, or other socially relevant groupings	N/A
Population characteristics	N/A
Recruitment	N/A
Ethics oversight	N/A

Note that full information on the approval of the study protocol must also be provided in the manuscript.

Field-specific reporting

Please select the one below that is the best fit for your research. If you are not sure, read the appropriate sections before making your selection.

☒ Life sciences ☐ Behavioural & social sciences ☐ Ecological, evolutionary & environmental sciences

For a reference copy of the document with all sections, see nature.com/documents/nr-reporting-summary-flat.pdf

Life sciences study design

All studies must disclose on these points even when the disclosure is negative.

Sample size	Statistical methods were not used to determine sample sizes.<br>For quantitation purposes, in general, unless otherwise stated a sample size of at least N =2 or N = 3 (two or three independent/biological repeats) was performed to enable assessment of the reproducibility and robustness of performed experiments. Sample sizes were selected based on similar studies in the field. For Mass Spectrometry Proteomics, 5 biological replicates were used per condition.
Data exclusions	No data were excluded from analyses unless otherwise noted.<br>For the SPR analysis, in some cases at the highest compound concentration sensorgrams exhibited some non-specific binding and these data points were typically excluded from fitting. For Jess endogenous degradation data and cellular luminescence and BRET assays, a small number of clear outlier values were excluded from fitting (arising due to compound solubility at high concentration, instrument variation or measurements outside dynamic range and inconsistent with other replicates). Excluded values are indicated in the data (e.g. Source data; Fig. 3b,c,e). For Mass Spectrometry Proteomics, only proteins identified using proteotypic peptides were retained for downstream analysis. To ensure data quality, protein groups that were present in at least 50% of replicates within at least one experimental condition were included for further analysis, resulting in a final set of 8,937 proteins.
Replication	To ensure reproducibility of experimental findings, biochemical/biophysical or cellular assays were repeated independently at least 2-3 times under equivalent conditions or using orthogonal techniques. Specific replicate information can be found in figure legends. For degradation studies, both HiBit tagged and endogenous proteins were compared to ensure reproducibility and specificity.<br>We confirm that all attempts at replication were successful.
Randomization	No experimental group allocation was carried out and so no randomization was performed.
Blinding	No experimental group allocation was carried out and so no blinding was performed.

Reporting for specific materials, systems and methods

We require information from authors about some types of materials, experimental systems and methods used in many studies. Here, indicate whether each material, system or method listed is relevant to your study. If you are not sure if a list item applies to your research, read the appropriate section before selecting a response.

Materials & experimental systems

n/a	Involved in the study
☐	☒ Antibodies
☐	☒ Eukaryotic cell lines
☒	☐ Palaeontology and archaeology
☒	☐ Animals and other organisms
☒	☐ Clinical data
☒	☐ Dual use research of concern
☒	☐ Plants

Methods

n/a	Involved in the study
☒	☐ ChIP-seq
☒	☐ Flow cytometry
☒	☐ MRI-based neuroimaging

Antibodies

Antibodies used

For cell based studies, the following antibodies (dilution, supplier, catalogue number) were obtained commercially:

Primary antibodies
rat anti-cIAP1 (1:500, Enzo, ALX-803-335)
mouse anti-XIAP(1:1000, MBL life science, M044-3)
rabbit anti-TEAD1 (1:50 for Jess, or otherwise 1:1000, Cell Signaling Technology, 12292S)
rabbit anti-Pan-TEAD (1:1000, Cell Signaling Technology, 13295S)
mouse anti-TEAD4 (1:1000, Abcam, ab58310)
rabbit anti GAPDH antibody (1:500 for Jess, Abcam, ab9485)
mouse anti-GAPDH (1:10,000 Sigma, G8795)
mouse anti-Hi-Bit (1:1000, Promega, N7200)
rat anti-Hsp90 (1:1000, Enzo life science, DI-SPA835)
rabbit anti-Lamin B1 (1:1000, Cell Signaling Technology,12586
rabbit anti-NFkb2 p100/p52 (1:1000, Cell Signaling Technology, 4882).

Secondary antibodies:
goat anti-Rat IgG HRP (1:5000, Southern Biotech, 3010-05)
goat Anti-Rabbit IgG HRP (1:5000, Southern Biotech, 4010-05)
goat anti-Mouse HRP (1:5000, Southern Biotech, 1010-05).

Validation

All the used antibodies are commercially available and have been validated by the manufacturer to be used in that species/ application and this information is provided on their websites and/or antibody datasheets. We have additionally validated TEAD1, TEAD4, cIAP1, XIAP antibodies with KO cell lines and data is provided as part of the paper.
We confirm that the antibodies used are fit for purpose.

rat anti-cIAP1 (1:500, Enzo, ALX-803-335)
See specific information from manufacturer:
https://www.enzo.com/product/c-iap1-monoclonal-antibody-1e1-1-10/

mouse anti-XIAP(1:1000, MBL life science, M044-3)
See specific information from manufacturer:
https://www.mblbio.com/bio/g/dtl/A/?pcd=M044-3

rabbit anti-TEAD1 (1:50 for Jess, or otherwise 1:1000, Cell Signaling Technology, 12292S)
See specific information from manufacturer:
https://www.cellsignal.com/products/primary-antibodies/tead1-d9x2l-rabbit-mab/12292

rabbit anti-Pan-TEAD (1:1000, Cell Signaling Technology, 13295S)
See specific information from manufacturer:
https://www.cellsignal.com/products/primary-antibodies/pan-tead-d3f7l-rabbit-mab/13295

mouse anti-TEAD4 (1:1000, Abcam, ab58310)
See specific information from manufacturer:
https://www.abcam.com/en-us/products/primary-antibodies/tead4-antibody-5h3-ab58310

rabbit anti GAPDH antibody (1:500 for Jess, Abcam, ab9485)
See specific information from manufacturer:
https://www.abcam.com/en-us/products/primary-antibodies/gapdh-antibody-loading-control-ab9485

mouse anti-GAPDH (1:10,000 Sigma, G8795)

See specific information from manufacturer:
https://www.sigmaaldrich.com/AU/en/product/sigma/g8795

mouse anti-Hi-Bit (1:1000, Promega, N7200)
See specific information from manufacturer:
https://www.promega.com.au/products/protein-detection/protein-quantification/anti-hibit-antibody/?catNum=N7200

rat anti-Hsp90 (1:1000, Enzo life science, ADI-SPA835)
See specific information from manufacturer:
https://www.enzo.com/product/hsp90-monoclonal-antibody-16f1/

rabbit anti-Lamin B1 (1:1000, Cell Signaling Technology, 12586)
See specific information from manufacturer:
https://www.cellsignal.com/products/primary-antibodies/lamin-b1-d4q4z-rabbit-mab/12586

rabbit anti-NFkb2 p100/p52 (1:1000, Cell Signaling Technology, 4882).
https://www.cellsignal.com/products/primary-antibodies/nf-kb2-p100-p52-antibody/4882

Eukaryotic cell lines

Policy information about cell lines and Sex and Gender in Research

Cell line source(s)	The human SCC-L cell lines NCI-H226, NCI-H2052, NCI-H520 and human HEK293T cell line were obtained from American Type Culture Collection (ATCC).<br>The human mesothelioma cell line ZL55 was provided by CellBank Australia.
Authentication	NCI-H226, NCI-H2052 and HEK293T (parental cell lines) as well as the NCI-H2052 Hibit cell lines were all authenticated using STR analysis.
Mycoplasma contamination	All cell lines used were routinely screened for mycoplasma contamination in a PCR-based assay and found negative.
Commonly misidentified lines<br>(See ICLAC register)	We have not used any commonly misidentified cell lines in this study.

Plants

Seed stocks	*Report on the source of all seed stocks or other plant material used. If applicable, state the seed stock centre and catalogue number. If plant specimens were collected from the field, describe the collection location, date and sampling procedures.*
Novel plant genotypes	*Describe the methods by which all novel plant genotypes were produced. This includes those generated by transgenic approaches, gene editing, chemical/radiation-based mutagenesis and hybridization. For transgenic lines, describe the transformation method, the number of independent lines analyzed and the generation upon which experiments were performed. For gene-edited lines, describe the editor used, the endogenous sequence targeted for editing, the targeting guide RNA sequence (if applicable) and how the editor was applied.*
Authentication	*Describe any authentication procedures for each seed stock used or novel genotype generated. Describe any experiments used to assess the effect of a mutation and, where applicable, how potential secondary effects (e.g. second site T-DNA insertions, mosiacism, off-target gene editing) were examined.*
